# TRIM21-mediated ubiquitination of PARP1 regulated by the PI3K/AKT-STAT5A axis suppresses small cell lung cancer

**DOI:** 10.1038/s41467-026-73271-1

**Published:** 2026-06-18

**Authors:** Guozhen Cao, Gongfeng Li, Xiaolin Wang, Tengfei Zhang, Xinhuang Yao, Peng Hou, Jiahui Zhang, Xiaopeng Guo, Jiarong Wang, Li Xiang, Hongbin Ji, Chenchen Guo, Wenchu Lin

**Affiliations:** 1 The Second Affiliated Hospital, School of Medicine, The Chinese University of Hong Kong, Shenzhen & Longgang District People’s Hospital of Shenzhen, Shenzhen, China; 2 University of Science and Technology of China, Hefei, China; 3 High Magnetic Field Laboratory, Chinese Academy of Sciences, Hefei, China; 4 Key Laboratory of Multi-Cell Systems, Shanghai Institute of Biochemistry and Cell Biology, Center for Excellence in Molecular Cell Science, Chinese Academy of Sciences, Shanghai, China; 5 University of Chinese Academy of Sciences, Beijing, China; 6 School of Life Science and Engineering, Lanzhou University of Technology, Lanzhou, China; 7 Westlake University School of Medicine, Hangzhou, China; 8 Shanghai Institute of Thoracic Oncology, Shanghai Chest Hospital, Shanghai Jiao Tong University School of Medicine, Shanghai, China; 9 Shanghai Key Laboratory of Thoracic Tumor Biotherapy, Shanghai Chest Hospital, Shanghai Jiao Tong University School of Medicine, Shanghai, China

**Keywords:** Small-cell lung cancer, Ubiquitylation

## Abstract

Abnormal accumulation of Poly(ADP-ribose) polymerase 1 (PARP1) promotes cancer progression, yet its stabilization mechanisms remain unclear. Here, we identify E3 ubiquitin ligase tripartite motif-containing 21 (TRIM21) as a PARP1-binding partner. PARP1 interacts directly with TRIM21 via its 662-908 domain, while the PRY-SPRY domain of TRIM21 is essential for this binding. TRIM21 facilitates PARP1 polyubiquitination at residue K654, leading to its degradation. In small cell lung cancer (SCLC), TRIM21 is significantly downregulated, and its tumor-suppressive function is partly mediated through the degradation of PARP1, supporting genomic stability. Additionally, the PI3K/AKT pathway transcriptionally suppresses TRIM21 via transcription factor STAT5A, thereby stabilizing PARP1. Importantly, combining the PI3K/AKT inhibitor PKI-587 with the PARP inhibitor BMN673 synergistically inhibits tumor growth across multiple SCLC models, including cell lines, patient-derived organoids, and xenograft models. Collectively, our findings define a “PI3K/AKT-STAT5A-TRIM21-PARP1” axis critical for SCLC progression and propose its dual inhibition as a promising therapeutic strategy.

## Abstract (web summary)

Aberrant accumulation of Poly(ADP-ribose) polymerase 1 (PARP1) contributes to cancer progression. Here the authors identify that the E3 ubiquitin ligase TRIM21 is transcriptionally downregulated in small cell lung cancer, therefore ubiquitination and degradation of PARP1 is inhibited.

## Introduction

Small cell lung cancer (SCLC) is a highly metastatic neuroendocrine lung cancer subtype with high lethality and poor prognosis^1^. Recent investigations into the genomic analysis of SCLC have shown extensive chromosomal rearrangements, high mutational burden, and abnormal expressions of epigenetic factors^2^. These alterations drive significant epigenetic variabilities and genomic instability, which are vital contributors to the development of SCLC^3^. However, the mechanisms through which these epigenetic abnormalities promote SCLC progression remain inadequately understood, posing challenges for the development of effective therapeutic strategies.

Poly (ADP-ribose) Polymerase-1 (PARP1), a protein sensing DNA strand breaks and orchestrating the subsequent repair process, plays a pivotal role in the cellular response to DNA damage and in the maintenance of genomic stability^4,5^. Elevated PARP1 expression, at both mRNA and protein levels, has been observed across various cancer types, including ovarian carcinoma^6^, lung cancer^7,8^, nasopharyngeal carcinoma^9^, breast cancer^10^, and oral cancer^11^. The stability of the PARP1 protein is often controlled by post-translational modifications (PTMs), such as ubiquitination on specific amino acids within the regulatory domains of PARP1. For instance, Ring Finger Protein 4 (RNF4), a SUMO-targeted ubiquitin E3 ligase, mediates heat-shock-inducible ubiquitination of PARP1 to maintain its stability^12^. In response to mitotic stress or DNA damage, the checkpoint with forkhead and ring finger domains (CHFR) catalyzes the synthesis of K48-linked polyubiquitination chains on PARP1, facilitating its degradation^13,14^. Additionally, Smurf2 has been shown to bind and ubiquitinate PARP1 under oxidative stress, leading to its subsequent degradation^15^. Despite these findings, the molecular mechanisms regulating PARP1 stability through ubiquitination under physiological conditions remain unclear.

To address these gaps, we conducted a screening of PARP1-interacting proteins and identified tripartite motif-containing 21 (TRIM21), a ring finger domain-containing E3 ubiquitin ligase, as a binding partner of PARP1. TRIM21 is well-established in various immunological processes, including antiviral response, inflammation modulation, and regulation of cytokine production^16^. As an E3 ubiquitin ligase, TRIM21 has been found to target several substrates, including VHL^17^, DDX41^18^, and RPA2^19^. Moreover, a wealth of recent studies has also indicated that TRIM21 is implicated in tumorigenesis, with evidence for both oncogenic and tumor-suppressive roles. For example, TRIM21 is downregulated in breast cancer^20^, B-cell lymphoma^21^, and renal carcinoma^22^, where it functions as a tumor suppressor. Conversely, TRIM21 has shown pro-oncogenic effects in gliomas^23^ and liver cancer through elevated expression^24^. However, the specific role of TRIM21 in SCLC is not fully understood. Moreover, while extensive research has focused on the downstream targets of TRIM21, the upstream regulation of TRIM21 expression is less well-understood.

In this study, we demonstrate that PARP1 interacts directly with TRIM21, with the 662-908 domain of PARP1 binding to the PRY-SPRY (PP) domain of TRIM21, an interaction essential for TRIM21-mediated ubiquitination-proteasomal degradation of PARP1. Further research indicates that TRIM21 is downregulated in SCLC and silencing *TRIM21* promotes SCLC cell survival and tumor growth, whereas overexpression of *TRIM21* exhibits the opposite effects. Notably, the tumor-suppressive function of TRIM21 in SCLC appears to depend on its role in mediating the ubiquitination-dependent degradation of PARP1. Furthermore, we reveal that both the downregulation of *TRIM21* transcription and the stabilization of PARP1 are regulated by STAT5A, which is modulated through the PI3K/AKT pathway. The combination of the PI3K/AKT inhibitor PKI-587 with the PARP inhibitor (PARPi) BMN673 demonstrates superior and durable anti-tumor effects in both in vitro and in vivo models of SCLC. Overall, our findings establish the “PI3K/AKT-STAT5A-TRIM21-PARP1” signaling axis as a critical pathway in SCLC tumor growth. Targeting this axis through dual inhibition with PI3K/AKT inhibitors and PARPi represents a promising therapeutic strategy for SCLC.

## Results

### PARP1 physically interacts with TRIM21

**Fig. 1:**
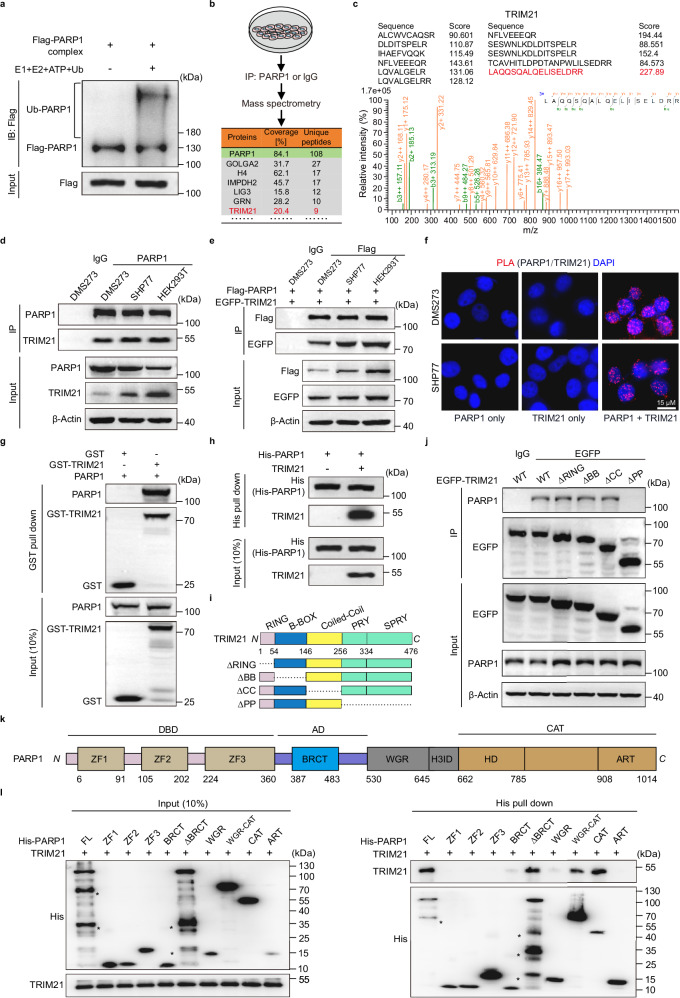
PARP1 directly interacts with TRIM21. **a** The PARP1-associated complex contains an E3 ligase. HEK293T cells were transfected with Flag-tagged PARP1 for 48 h, followed by immunoprecipitation (IP) using a Flag antibody to isolate the Flag-PARP1 complexes. An in vitro ubiquitylation assay was then performed with E1, E2, ubiquitin, ATP, and the anti-Flag immunoprecipitates. **b** Schematic representation of the experimental workflow for identifying PARP1-associated proteins. HEK293T cell lysates were subjected to IP with an anti-PARP1 antibody to enrich PARP1 and its interacting proteins. These complexes were then analyzed by liquid chromatography-tandem mass spectrometry (LC-MS/MS) (*n* = 1). **c** Representative MS/MS fragmentation spectrum identifying TRIM21 as a binding partner of PARP1. **d** Validation of the endogenous interaction between PARP1 and TRIM21 in DMS273, SHP77, and HEK293T cells using Co-IP assays. **e** Co-IP assays demonstrating the interaction between ectopically expressed Flag-*PARP1* and EGFP-*TRIM21* in DMS273, SHP77, and HEK293T cells. **f** Representative images of in situ PLA of the interaction between PARP1 and TRIM21 proteins in DMS273 and SHP77 cells. Red fluorescent signals indicate the interaction between PARP1 and TRIM21. Nuclei (blue) were stained with DAPI. **g**, **h** In vitro interaction between recombinant TRIM21 and PARP1 confirmed by both GST pull-down (**g**) and His pull-down (**h**) assays. **i** Schematic of EGFP-tagged TRIM21 and various deletion mutants used in this study. ∆RING, ∆BB, ∆CC, and ∆PP represent deletion mutants of the RING, B-BOX, Coiled-Coil, and PRY-SPRY domains, respectively. **j** Co-IP assays elucidating the interaction between PARP1 and various TRIM21 truncations. Full-length and truncated EGFP-tagged *TRIM21* constructs were transfected into HEK293T cells. 48 h post-transfection, EGFP-trap was utilized to isolate EGFP-tagged proteins, followed by immunoblot analysis with specific antibodies to probe the domains of PARP1-TRIM21 interactions. **k** Schematic diagram of PARP1 protein domains alongside deletion constructs. **l** His pull-down assays showing the interaction between TRIM21 and the functional domains of PARP1, including constructs such as His-PARP1, His-ZF1, His-ZF2, His-ZF3, His-BRCT, His-∆BRCT, His-WGR, His-WGR-CAT, His-CAT, and His-ART in vitro. Asterisks in (**l**) indicate non-specific bands. Source data are provided as a Source Data file.

To investigate the ubiquitination-mediated degradation of PARP1 under physiological conditions, we performed an in vitro ubiquitination assay using anti-Flag immunoprecipitates from HEK293T cells overexpressing Flag-*PARP1*, along with purified recombinant E1 and E2. PARP1 ubiquitylation was observed without the addition of a recombinant E3 ligase (Fig. 1a), indicating that an endogenous E3 enzyme was co-immunoprecipitated in the Flag-PARP1 complexes. Mass spectrometry (MS) analysis of these anti-PARP1 immunoprecipitates identified TRIM21, an E3 ubiquitin ligase, as one of the PARP1-binding proteins (Fig. 1b, c, and Supplementary Data 1), suggesting that TRIM21 could function as an E3 ligase for PARP1 ubiquitylation. To validate this interaction, we performed co-immunoprecipitation (Co-IP) assays and found that endogenous TRIM21 indeed bound to PARP1 in DMS273, SHP77, and HEK293T cells (Fig. 1d). Reverse Co-IP assays using an anti-Flag antibody further confirmed the interactions of exogenous PARP1 and TRIM21 in Flag-*PARP1* and EGFP-*TRIM21*-transfected cells (Fig. 1e). Consistent with these findings, immunofluorescence staining revealed that both TRIM21 and PARP1 were mainly located in the nucleus (Supplementary Fig. 1a). A proximity ligation assay (PLA), which visualizes protein interactions in situ, showed prominent red fluorescent dots representing endogenous TRIM21-PARP1 interactions (Fig. 1f). Together, these results indicate that TRIM21 binds to PARP1 across various cell types.

Next, we sought to clarify the molecular basis of the PARP1-TRIM21 interaction. Pull-down assays using recombinant PARP1 and TRIM21 in vitro demonstrated that PARP1 could physically interact with TRIM21 (Fig. 1g, h, and Supplementary Fig. 1b). Since TRIM21 contains four conserved domains, RING-finger domain (RING), B-box domain (BB), Coiled-coil domain (CC), and PRY-SPRY (PP) domain^25^ (Fig. 1i), we investigated which domain of TRIM21 was essential for binding PARP1. Full-length and various deletion forms of TRIM21 were transfected into HEK293T cells, followed by Co-IP assays. Western analysis showed that deletion of the PP domain in TRIM21 led to a near-complete loss of interaction with PARP1 (Fig. 1j), suggesting that the PP domain is primarily required for this interaction. It has been reported that residues W381 and W383 within the PP domain are central for interacting with IgG Fc as well as other substrates^26^. Interestingly, we found that the TRIM21-PARP1 interaction is independent of the W381/W383 residues in TRIM21 (Supplementary Fig. 1c). Furthermore, a ligase-deficient (LD) mutant (C16A, C31A, and H33W)^27^ of TRIM21 retained its binding to PARP1 (Supplementary Fig. 1c), indicating that the E3 ligase activity of TRIM21 is not required for the association with PARP1.

To identify the domain of PARP1 required for TRIM21 binding (Fig. 1k and Supplementary Fig. 1d), we conducted His pull-down assays and mapped the 662-908 domain of PARP1 as necessary and sufficient for the PARP1-TRIM21 interaction (Fig. 1l). The WGR and 662-908 domains of PARP1 include an additional 18-amino-acid segment, the H3ID region, which is known to be critical for binding with H3.3^28^. We further examined whether the H3ID domain is essential for TRIM21 binding using Co-IP assays, finding that loss of H3ID did not weaken the interaction between PARP1 and TRIM21 (Supplementary Fig. 1e). These results confirm that the PARP1-TRIM21 interaction primarily depends on the 662-908 domain of PARP1 and the PP domain of TRIM21.

### TRIM21 ubiquitinates PARP1 via K48 linkage

**Fig. 2:**
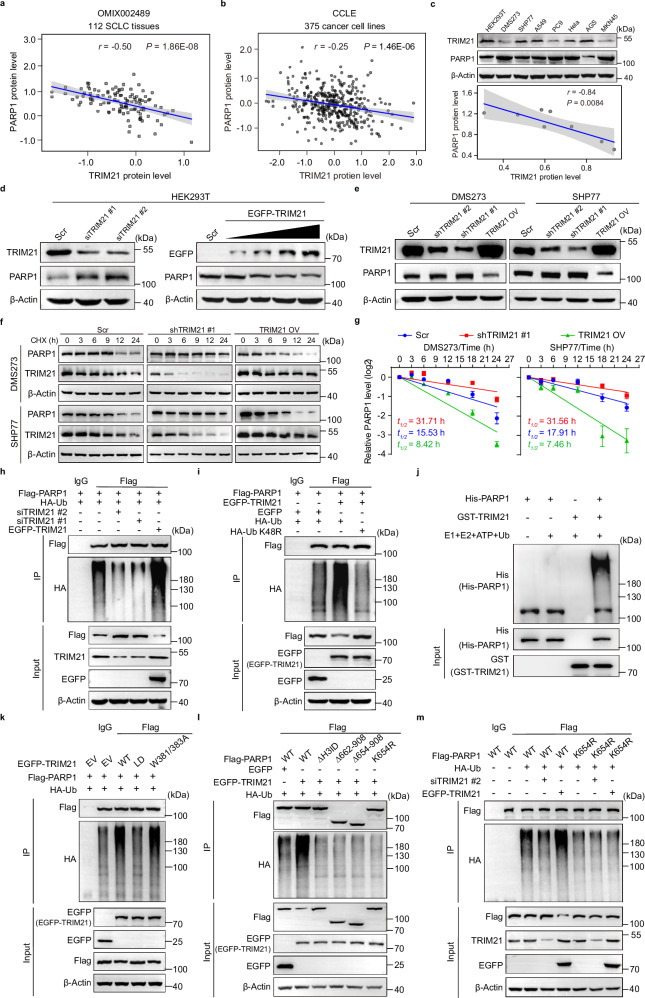
TRIM21 mediates the ubiquitin-dependent degradation of PARP1. **a**–**c** Correlation analysis of TRIM21 and PARP1 protein levels in SCLC tissues from the OMIX database (OMIX002489, *n* = 112; 95% confidence interval (CI), −0.63 to −0.35) (**a**), across 375 cancer cell lines (CCLE database; 95% CI, −0.34 to −0.14) (**b**), and in a panel of cell lines (*n* = 8; 95% CI, −0.97 to −0.34) (**c**). Two-tailed *P* values are reported in (**a**–**c**). **d** Effect of TRIM21 on PARP1 expression. Western blot analysis of TRIM21 and PARP1 protein levels in HEK293T cells with modulated *TRIM21* expression. **e** Assessment of PARP1 expression following *TRIM21* modulation. Western blot analyses were performed to assess PARP1 expression after knockdown and overexpression of *TRIM21* in DMS273 and SHP77 cells. **f** Effect of TRIM21 on PARP1 stability. DMS273 and SHP77 cells with *TRIM21* knockdown or overexpression were treated with CHX (100 μg/ml) for the indicated times, and PARP1 protein levels were analyzed by Western blot. **g** Quantification of PARP1 protein levels relative to β-Actin based on the results obtained from (**f**), and data from three independent experiments are shown as mean ± SEM. **h** The ubiquitination of PARP1. HEK293T cells were co-transfected with Flag-*PARP1*, HA-Ub, and either si*TRIM21* or EGFP-*TRIM21*, followed by analysis of Flag-PARP1 ubiquitination. **i** Assessment of specific ubiquitination pathways. The procedure was performed as described in (**h**). **j** In vitro ubiquitination assays of PARP1. The reaction system included purified recombinant His-PARP1 and GST-TRIM21, along with ubiquitin (Ub), E1 enzyme (UbE1), and E2 enzyme (UbcH5c). After 3 h of incubation at 37 °C, the reaction was terminated, and PARP1 ubiquitination was analyzed by immunoblotting. **k** Detection of TRIM21-mediated PARP1 polyubiquitination. HEK293T cells were co-transfected with Flag-*PARP1*, HA-Ub, and either EGFP-*TRIM21* WT, EGFP-*TRIM21* LD (C16A, C31A, and H33W) mutant, or EGFP-*TRIM21* W381/383 A mutant. The ubiquitination levels of Flag-PARP1 were evaluated by IP-Western blot. **l** Lysine 654 (K654) was identified as the polyubiquitination site on PARP1 mediated by TRIM21, using a procedure analogous to (**k**). **m** The K654 ubiquitination site was further validated following a similar methodology as in (**k**). Source data are provided as a Source Data file.

To explore the regulatory impact of TRIM21 on PARP1, we conducted Pearson’s correlation analysis between TRIM21 and PARP1 protein expression levels. A significant negative correlation was observed in both primary human SCLC samples (Fig. 2a) and in a panel of 375 cell lines across 38 cancer types (Fig. 2b), and this inverse relationship was further confirmed in a separate set of cell lines (Fig. 2c). Together, these data indicate that TRIM21 negatively correlates with PARP1 protein expression across various cancer contexts.

To determine whether TRIM21 specifically affects PARP1 protein levels, we conducted a series of knockdown and overexpression experiments. Western blot analysis demonstrated that *TRIM21* knockdown markedly led to PARP1 accumulation, while *TRIM21* overexpression significantly reduced PARP1 protein levels in DMS273, SHP77, and HEK293T cells (Fig. 2d, e, and Supplementary Fig. 2a). Notably, *PARP1* mRNA levels remained unchanged under these conditions (Supplementary Fig. 2b–d), suggesting that TRIM21 regulates PARP1 post-transcriptionally.

Next, we evaluated whether *TRIM21* influences PARP1 protein stability. CHX chase assays revealed that *TRIM21* knockdown significantly increased PARP1 stability, while *TRIM21* overexpression decreased its half-life (Fig. 2f, g). These results imply that TRIM21 promotes PARP1 degradation. To confirm that TRIM21-mediated PARP1 degradation occurs via the ubiquitin-proteasome pathway, we examined PARP1 ubiquitination in cells with altered *TRIM21* expression. Overexpression of *TRIM21* notably enhanced PARP1 ubiquitination, while *TRIM21* knockdown reduced it (Supplementary Fig. 2e). Similar effects were also observed in HEK293T cells (Fig. 2h), indicating that TRIM21-mediated regulation of PARP1 stability is not restricted to SCLC cells. Importantly, expression of the HA-Ub K48R mutant, which blocks K48-linked polyubiquitination, resulted in a substantial reduction of PARP1 ubiquitination compared with wild-type ubiquitin (Fig. 2i), indicating that TRIM21 ubiquitinates PARP1 primarily through K48 linkages. Moreover, we further confirmed TRIM21’s role as an E3 ligase for PARP1 by performing in vitro ubiquitination assays with purified recombinant His-PARP1 and GST-TRIM21 in the presence of ubiquitin (Ub), E1 (UbE1), and E2 (UbcH5c) enzymes. Polyubiquitination of PARP1 was observed in the presence of GST-TRIM21 (Fig. 2j). Notably, both the TRIM21 LD mutant and PP domain deletion mutant showed significantly reduced PARP1 ubiquitination compared to wild-type TRIM21 (Fig. 2k and Supplementary Fig. 2f), indicating that both TRIM21’s E3 ligase activity and its PP domain are essential for ubiquitinating PARP1. Interestingly, deletion of the 662-908 domain in PARP1 nearly abolished TRIM21-mediated ubiquitination (Supplementary Fig. 2g), underscoring the necessity of the TRIM21-PARP1 interaction for efficient ubiquitination.

Furthermore, we have identified K654 on PARP1, located within the H3ID region adjacent to the 662-908 domain, as a key ubiquitination site^28^. Ubiquitylation assays with full-length PARP1 and various mutants revealed that the K654R mutation dramatically reduced PARP1 polyubiquitination, similar to the deletion of the H3ID region (Fig. 2l), demonstrating that K654 is a critical site for TRIM21-mediated ubiquitination of PARP1. The K654R mutation did not impair TRIM21 binding to PARP1 (Supplementary Fig. 1e). Notably, in cells transfected with Flag-*PARP1* K654R, TRIM21 expression does not affect PARP1 ubiquitination levels (Fig. 2m), further supporting the essential role of K654 in TRIM21-mediated PARP1 degradation.

Our studies also demonstrate that oxidative stress (H_2_O_2_ or etoposide (EP) treatment) reduces PARP1 ubiquitination and consequently increases PARP1 accumulation (Supplementary Fig. 2h, i), suggesting cellular attenuation of PARP1 degradation to preserve DNA repair capacity under genotoxic stress. Importantly, while oxidative stress moderately diminishes TRIM21-mediated PARP1 ubiquitination, TRIM21 consistently promotes PARP1 degradation under both normal and stress conditions (Supplementary Fig. 2h). These findings establish that TRIM21 maintains its role in facilitating ubiquitin-dependent PARP1 degradation across physiological and pathological contexts, underscoring its critical function in regulating PARP1 protein homeostasis regardless of cellular stress status.

### TRIM21 suppresses SCLC progression

**Fig. 3:**
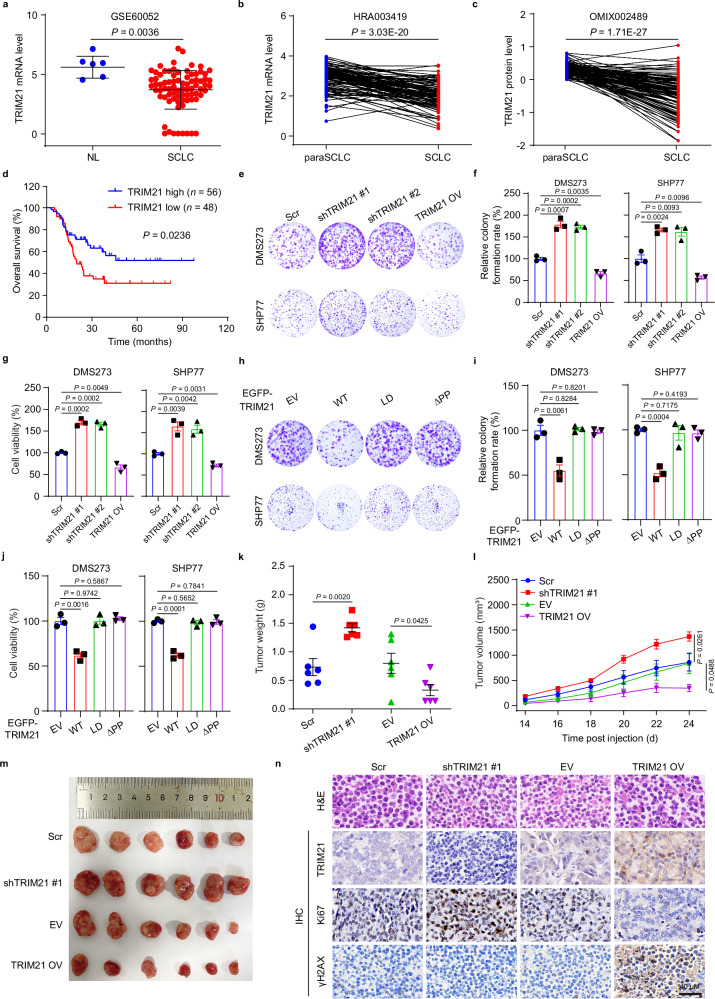
TRIM21 inhibits the progression of SCLC. **a** Scatter plot of *TRIM21* expression in the GSE60052 dataset showing significantly lower levels in SCLC (*n* = 79) versus normal lung (NL) tissues (*n* = 6). **b** Analysis of *TRIM21* mRNA expression in 107 paired SCLC and adjacent normal tissues (paraSCLC) from the GSA database (HRA003419). **c** Protein expression of TRIM21 in 112 paired SCLC and paraSCLC from the OMIX database (OMIX002489). **d** Kaplan-Meier survival analysis of SCLC patients stratified by TRIM21 expression levels (OMIX002489, log-rank test). **e** Colony formation assays performed upon *TRIM21* knockdown (sh*TRIM21* #1 and #2) or overexpression (*TRIM21* OV) in DMS273 and SHP77 cells. **f** Quantitative analysis of clonogenic survival was conducted using ImageJ software based on the data from (**e**). **g** Cell viability was measured using the CellTiter-Glo luminescent assay in the same cells as in (**e**). **h** Effect of TRIM21 and its mutants on SCLC cell survival. Clonogenic assays were performed in DMS273 and SHP77 cells transfected with EGFP-*TRIM21* WT, EGFP-*TRIM21* LD, or EGFP-*TRIM21* ∆PP. **i** Quantification of clonogenic survival from (**h**), analyzed using ImageJ software. **j** Parallel assessment of cell viability using the CellTiter-Glo luminescent assay under the same transfection conditions as in (**h**). **k** Scatter plot displaying tumor weights from DMS273 xenograft mice subjected to scramble control (Scr), *TRIM21* knockdown (sh*TRIM21* #1), or overexpression (*TRIM21* OV) (*n* = 6 for each group). **l** Tumor volume curves of DMS273 xenograft mice under the same conditions as in (**k**). **m** Representative images of xenograft tumors from the groups in (**k**). **n** Representative H&E staining and immunohistochemistry for TRIM21, Ki67, and γH2AX in tumor sections. Scale bars, 40 μm. Data in (**a**) are shown as mean ± SD. Data are from three independent experiments in (**f**, **g**, **i**, **j**) or 6 mice per group in (**k,** **l**) and are presented as mean ± SEM. Statistical analysis was performed using two-tailed unpaired Student’s *t*-tests for (**a**, **f**, **g**, **i**–**l**), and two-tailed paired Student’s *t*-tests for (**b**, **c**). Source data are provided as a Source Data file.

Subsequently, we investigated the functional significance of TRIM21 in SCLC. The results indicated that, in the CCLE database, the mRNA levels of *TRIM21* were significantly lower in SCLC cell lines compared to those from normal tissues (Supplementary Fig. 3a, b). Similarly, *TRIM21* mRNA levels were markedly reduced in SCLC tissues compared to normal or adjacent non-cancerous tissues (Fig. 3a, b, and Supplementary Fig. 3c–f). Consistent with mRNA data, protein levels of TRIM21 were also significantly downregulated in SCLC tissues relative to adjacent tissues (Fig. 3c). Kaplan-Meier survival analysis revealed that lower TRIM21 expression was significantly associated with shorter overall survival in SCLC patients (Fig. 3d). Collectively, these data suggest that low TRIM21 expression in SCLC is indicative of poor prognosis.

To investigate the role of TRIM21 in SCLC tumor growth, we conducted cell viability and clonogenic assays. Depletion of *TRIM21* significantly promoted cell viability and clonogenicity of SCLC cells, whereas *TRIM21* overexpression notably inhibited these phenotypes (Fig. 3e–g). Recent studies have suggested that TRIM21’s E3 ubiquitin ligase activity plays a crucial role in various cancers^29^. We speculated that the inhibitory effect of TRIM21 on SCLC cell activity might depend on both its E3 ubiquitin ligase activity and the PP domain necessary for PARP1 binding. Supporting this, cell viability and clonogenic assays showed that overexpression of wild-type *TRIM21* significantly inhibited the viability of SCLC, whereas TRIM21 mutants lacking the PP domain or E3 ligase activity did not affect SCLC survival (Fig. 3h–j). These results suggest that TRIM21’s impact on SCLC survival likely requires its enzymatic function and its interaction with PARP1. Similarly, we observed consistent effects in other SCLC subtype cell lines, including SCLC-*P* (H526) and SCLC-*Y* (H196) (Supplementary Fig. 3g–i), indicating that the tumor-suppressive role of TRIM21 is independent of SCLC molecular subtypes.

Consistently, in vivo experiments corroborated these findings, showing that *TRIM21* silencing significantly accelerated tumor growth, while ectopic expression of *TRIM21* delayed tumor progression (Fig. 3k–m). Histological examination of SCLC specimens through H&E staining is illustrated in Fig. 3n. Immunohistochemical analysis demonstrated that *TRIM21* knockdown substantially increased the expression of Ki67, a well-known marker of cell proliferation, whereas *TRIM21* overexpression reduced Ki67 levels. Additionally, γH2AX, a marker of DNA damage, was markedly elevated in the *TRIM21* overexpression group (Fig. 3n). Collectively, these findings underscore that TRIM21 is a key determinant of SCLC tumorigenic potential and suggest its expression level may be critical for modulating cancer progression.

### TRIM21 inhibits SCLC cell survival by destabilizing PARP1

**Fig. 4:**
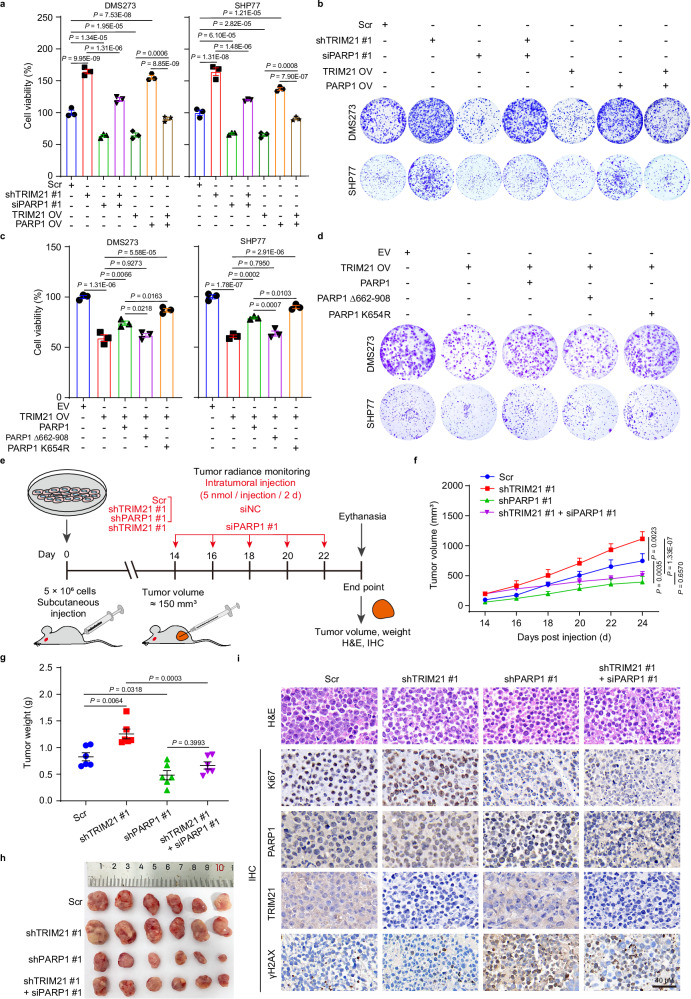
TRIM21 inhibits SCLC cell survival by limiting the accumulation of PARP1. **a** Evaluation of the compensatory effect of PARP1 on *TRIM21* knockdown or overexpression. DMS273 and SHP77 cells with *TRIM21* knockdown or overexpression were transfected with si*PARP1* or Flag-*PARP1*. Subsequent cell viability was measured using the CellTiter-Glo luminescent assay. **b** Cell survival was measured via colony formation assays in cells described in (**a**). **c** Assessment of the compensatory effects of full-length PARP1 and its mutants on *TRIM21*-overexpressing cells. DMS273 and SHP77 cells with *TRIM21* overexpression were transfected with Flag-tagged *PARP1*, *PARP1* Δ662-908, or *PARP1* K654R. Subsequent cell viability was measured using the CellTiter-Glo luminescent assay. **d** Cell survival was measured via colony formation assays in cells described in (**c**). **e** Schematic representation of the experimental workflow of the xenograft study. Arrows indicate the times when different events occurred. **f** Tumor volume curves of xenograft mice in vivo (*n* = 6 for each group). ‘Scr’ denotes the control group, ‘sh*TRIM21* #1’ represents *TRIM21* knockdown, and ‘sh*PARP1* #1’ indicates *PARP1* knockdown. Meanwhile, ‘sh*TRIM21* #1 + si*PARP1* #1’ refers to *TRIM21* knockdown tumors that received intratumoral injection of si*PARP1* #1. **g** Scatter plot depicting tumor weights from xenograft mice at the end points of the experiments. **h** Representative images of xenograft tumors were displayed as described in (**f**). **i** Representative images of H&E staining and immunohistochemistry for Ki67, PARP1, TRIM21, and γH2AX within tumor sections. Scale bars represent 40 μm. Data in (**a,** **c**) are from three independent experiments, and data in (**f**, **g**) are from 6 mice. All data are presented as mean ± SEM. Statistical analysis was performed using two-tailed one-way ANOVA. Source data are provided as a Source Data file.

Given TRIM21’s role in promoting ubiquitination-mediated degradation of PARP1, we hypothesized that TRIM21’s inhibitory effect on SCLC might be linked to PARP1 protein stability. To test this, we modulated *PARP1* expression in DMS273 and SHP77 cells with either *TRIM21* knockdown or overexpression (Supplementary Fig. 4a), followed by cell viability and clonogenic assays. The results showed that *PARP1* knockdown or overexpression partially rescued the effects of *TRIM21* modulation on SCLC cell viability (Fig. 4a, b, and Supplementary Fig. 4b), indicating that TRIM21’s impact on cell viability is, at least in part, dependent on PARP1. The partial nature of this rescue may be attributable to TRIM21’s additional roles as an E3 ligase, potentially targeting other substrates beyond PARP1.

Building on these findings, we proposed that TRIM21’s inhibition of SCLC cell survival might be associated with the ubiquitination and degradation of PARP1. To further investigate, we transfected *TRIM21*-overexpressing cells with full-length *PARP1*, ∆662-908 mutant, or K654R mutant (Supplementary Fig. 4c), and assessed cell survival via CellTiter-Glo and clonogenic assays. The results showed that full-length *PARP1* partially reversed the inhibitory effect of *TRIM21* overexpression on SCLC cell survival. Notably, the K654R mutant exhibited an even greater compensatory effect than full-length *PARP1* (Fig. 4c, d, and Supplementary Fig. 4d), likely due to the enhanced stability of PARP1 conferred by the K654R mutation. These results indicate that TRIM21 impacts cell survival, to some extent, by destabilizing PARP1 in SCLC cells.

Consistent with these observations, in vivo experiments further demonstrated that *TRIM21* silencing significantly enhanced the tumorigenic potential of SCLC cells, while *PARP1* silencing markedly curtailed tumor progression (Fig. 4e–h). Importantly, additional *PARP1* silencing via intratumoral injection of si*PARP1* in the *TRIM21* knockdown tumors yielded a more profound inhibition in tumor growth (Fig. 4f–h). Immunohistochemical analysis further confirmed that *TRIM21* depletion led to significant accumulation of PARP1 protein and elevated Ki67 expression (Fig. 4i). However, once *PARP1* was additionally silenced in *TRIM21*-knockdown cells, Ki67 expression markedly decreased (Fig. 4i). Together, these results reinforce the conclusion that TRIM21’s inhibitory effect on SCLC tumorigenicity is closely linked to its regulation of PARP1 protein stability.

### TRIM21 loss enhances DNA damage response by restricting PARP1 degradation

**Fig. 5:**
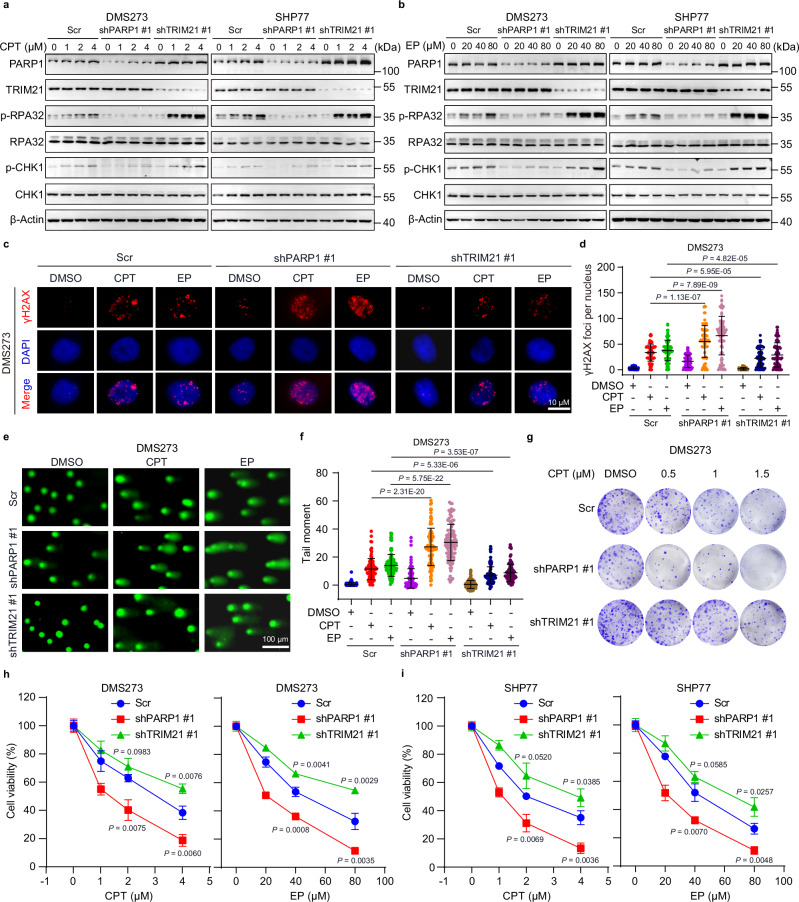
TRIM21 deficiency amplifies DNA damage responses by preventing PARP1 degradation. **a**, **b** Western blot analysis showing the activation of DNA damage repair signal post-DNA damage in SCLC cells upon *PARP1* or *TRIM21* knockdown. DMS273 and SHP77 cells were treated for 1 h with increasing amounts of camptothecin (CPT) (DMSO, 1, 2, 4 μM) or etoposide (EP) (DMSO, 20, 40, 80 μM), followed by washing and incubation in fresh medium for 4 h. PARP1, TRIM21, CHK1, p-CHK1 (Ser345), RPA32, and p-RPA32 (S4/S8) proteins were analyzed by immunoblotting. **c** Immunofluorescent staining depicting the change of DNA damage-induced γH2AX foci in SCLC cells upon *PARP1* or *TRIM21* knockdown. Immunostaining analysis of γH2AX foci formation after 1 h of treatment with 2 μM CPT or 40 μM EP, followed by 4 h of recovery in *PARP1* or *TRIM21* stably knockdown DMS273 cells. Scale bars, 10 μm. **d** Quantification of the γH2AX foci in (**c**). **e** Comet assays depicting the effect of *PARP1* or *TRIM21* knockdown on DNA damage in DMS273 cells following the same treatment conditions as described in (**c**). Scale bars, 100 μm. **f** Quantification of the tail moment of comet assays in (**e**). **g** Colony formation assays measuring the clonogenic capacity of the DMS273 cells treated by CPT with indicated concentrations upon *PARP1* or *TRIM21* knockdown. **h**, **i** Cell viability assays depicting cellular survival following DNA damage upon *PARP1* or *TRIM21* knockdown. Cells were treated as described in (**a**, **b**), and cell viability was assessed using the CellTiter-Glo luminescent assay after 48 h. Data in (**d**) (*n* = 80 cells) and (**f**) (*n* = 100 cells) are presented as mean ± SD. In (**h**, **i**), data from three independent experiments are shown as mean ± SEM. Statistical analysis was performed by two-tailed unpaired *t*-tests in (**d**, **f**, **h**, **i**). Source data are provided as a Source Data file.

Given that TRIM21 mediates the proteolytic ubiquitination of PARP1, which is a decisive factor in the cellular response to DNA damage, we hypothesized that TRIM21 might influence cellular sensitivity to DNA damage by modulating PARP1 stability. To test this, we assessed DNA damage response (DDR) signaling in cells exposed to genotoxic stress following knockdown of either *PARP1* or *TRIM21*. After treatment with the topoisomerase-I inhibitor camptothecin (CPT) or the topoisomerase-II inhibitor EP to induce DNA damage, the kinetics of CHK1 and RPA32 phosphorylation were examined via Western blotting. The results showed enhanced phosphorylation of these DDR proteins in *TRIM21*-depleted cells, a pattern opposite to that observed in *PARP1*-knockdown cells, suggesting that *TRIM21* loss promotes DNA repair by stabilizing PARP1 (Fig. 5a, b).

*TRIM21* depletion significantly reduced γH2AX levels, whereas SCLC cells lacking *PARP1* exhibited increased γH2AX levels following CPT or EP treatment in both DMS273 and SHP77 cells (Fig. 5c, d, and Supplementary Fig. 5a, b). Similarly, in H526 and H196 cell lines, *TRIM21* knockdown markedly attenuated CPT-induced γH2AX foci formation, while *TRIM21* overexpression further elevated γH2AX levels under the same genotoxic conditions (Supplementary Fig. 5c–f). Notably, overexpression of the *TRIM21*-LD mutant did not alter γH2AX levels in H526 and H196 cells (Supplementary Fig. 5c–f). Moreover, *PARP1* silencing partially rescued the *TRIM21* knockdown-mediated reduction in γH2AX signals (Fig. 4i). Comet assays further confirmed that *PARP1*-depleted cells exhibited significantly increased tail moments compared to controls, while *TRIM21*-deficient cells showed reduced DNA fragmentation after treatment (Fig. 5e, f, and Supplementary Fig. 5g, h). Together, these results demonstrate that *TRIM21* loss enhances DNA repair efficiency in SCLC cells by stabilizing PARP1, thereby limiting DNA damage accumulation.

Further analysis of cellular susceptibility to DNA-damaging agents via colony-forming assays revealed that *PARP1* knockdown increased cellular sensitivity to CPT and EP, while *TRIM21* knockdown conferred resistance to these agents (Fig. 5g and Supplementary Fig. 5i–o). This pattern correlated with reduced survival in *PARP1*-depleted cells and increased survival in *TRIM21*-knockdown cells (Fig. 5h, i). In summary, our findings reveal that *TRIM21* depletion strengthens the DNA damage response, promoting DNA repair and SCLC cell survival, likely due to the increased stability and accumulation of PARP1.

### PI3K/AKT promotes PARP1 stabilization through downregulation of TRIM21

**Fig. 6:**
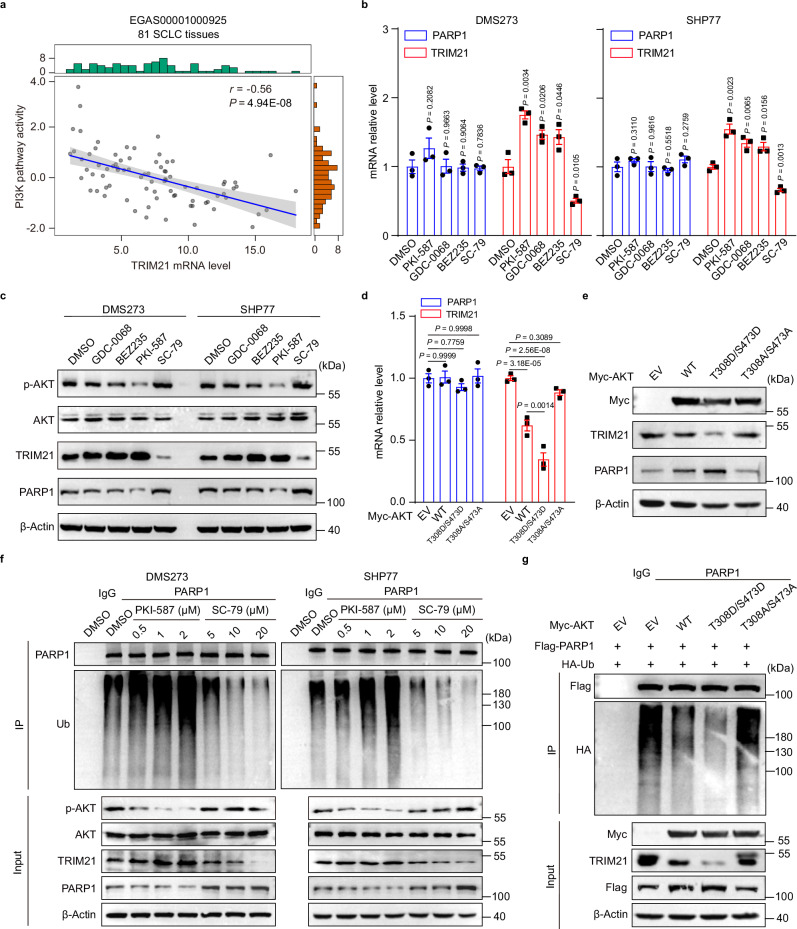
PI3K/AKT promotes PARP1 stabilization through downregulation of TRIM21. **a** Scatter plot depicting the correlations between the PI3K/AKT pathway activity and *TRIM21* mRNA levels in SCLC tissues (*n* = 81; 95% confidence interval, −0.69 to −0.39). Two-tailed *P* value is reported. **b** RT-qPCR analysis demonstrating that the PI3K/AKT signaling pathway suppresses *TRIM21* transcription. DMS273 and SHP77 cells were treated with PI3K/AKT inhibitors PKI-587 (1 µM), GDC-0068 (10 µM), BEZ235 (40 µM), or a PI3K/AKT activator SC-79 (10 µM) for 24 h, followed by measurement of *TRIM21* and *PARP1* mRNA levels. **c** Western blot analysis of p-AKT, AKT, TRIM21, and PARP1 after treatment with PI3K/AKT inhibitors and activators. Cells were treated as shown in panel (**b**), followed by measurement of TRIM21 and PARP1 protein levels via Western blot analysis. **d**, **e** RT-qPCR and Western blot analyses of *TRIM21* and *PARP1* following ectopic expression of AKT and its mutants. HEK293T cells were transfected with AKT-WT, AKT-T308D/S473D, or AKT-T308A/S473A plasmids. Following transfection, the mRNA (**d**) and protein (**e**) levels of TRIM21 and PARP1 were measured. **f** Ubiquitination analysis showing ubiquitination of PARP1 following the treatment with a PI3K/AKT inhibitor or activator. DMS273 and SHP77 cells were treated with different concentrations of PKI-587 or SC-79 for 24 h. Following treatment, the ubiquitination levels of the PARP1 protein were assessed using immunoprecipitation followed by Western blot. **g** Ubiquitination analysis demonstrating PARP1 ubiquitination following ectopic expression of AKT and its mutants. HEK293T cells were treated similarly to (**d**), followed by ubiquitination assays as described in (**f**). In (**b**, **d**), data from three independent experiments are shown as mean ± SEM. Statistical analysis was performed using two-tailed unpaired *t*-tests. Source data are provided as a Source Data file.

To explore the upstream regulation of TRIM21 expression, we analyzed the correlation between signaling pathways and *TRIM21* mRNA levels in human primary SCLC tissues using two independent SCLC RNA-seq datasets. The analysis revealed a significant negative correlation between the PI3K/AKT pathway activity and *TRIM21* mRNA levels (Fig. 6a and Supplementary Fig. 6a–c). Inhibition of the PI3K/AKT pathway using specific inhibitors (PKI-587, GDC-0068, or BEZ235) in SCLC cells increased TRIM21 expression while reducing PARP1 protein levels; conversely, PI3K/AKT activation by SC-79 markedly decreased TRIM21 at both transcriptional and protein levels and promoted PARP1 accumulation (Fig. 6b, c and Supplementary Fig. 6d, e), suggesting that TRIM21 is regulated through a PI3K/AKT-dependent mechanism. Intriguingly, overexpression of AKT and its constitutively activated form (AKT-T308D/S473D), but not its inactivated form (AKT-T308A/S473A), inhibited TRIM21 expression and increased PARP1 protein levels (Fig. 6d, e). These results suggest that PI3K/AKT signaling suppresses TRIM21 expression, thereby promoting PARP1 accumulation.

To further verify that the PI3K/AKT pathway promotes PARP1 accumulation by inhibiting *TRIM21* transcription and thus suppressing PARP1 ubiquitination and degradation, we assessed PARP1 ubiquitination levels following treatment with PI3K/AKT inhibitor (PKI-587) and activator (SC-79). Western blot analysis showed that PI3K/AKT inhibition significantly enhanced PARP1 ubiquitination, whereas PI3K/AKT activation suppressed PARP1 ubiquitination (Fig. 6f). Consistently, overexpression of AKT and its activating mutant, but not its inactive form, significantly inhibited PARP1 ubiquitination (Fig. 6g). These results collectively indicate that the PI3K/AKT pathway facilitates PARP1 accumulation by reducing its ubiquitination and degradation via downregulation of *TRIM21* transcription.

### Inhibition of *TRIM21* transcription is mediated by the PI3K/AKT/STAT5A axis

**Fig. 7:**
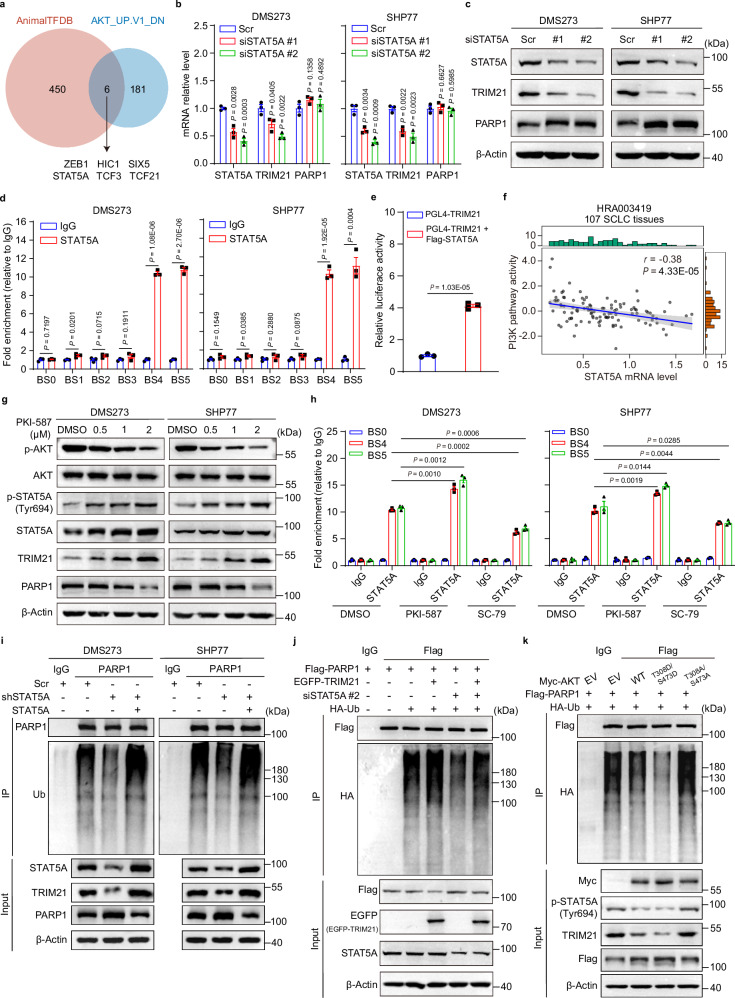
The transcriptional inhibition of TRIM21 is mediated by the PI3K/AKT/STAT5A axis. **a** Venn diagram showing the overlap of 6 candidate transcription factors (TFs) that may regulate *TRIM21*. These TFs represent the intersection between 456 *TRIM21* promoter-binding TFs (AnimalTFDB database) and genes suppressed by the PI3K/AKT pathway (GSEA database). **b, c** RT-qPCR (**b**) and Western blot (**c**) analysis of the levels of STAT5A, TRIM21, and PARP1 after *STAT5A* knockdown using si*STAT5A*. **d** ChIP-qPCR analysis of STAT5A binding to the *TRIM21* promoter in DMS273 and SHP77 cells. **e** Dual-luciferase reporter assay demonstrating STAT5A binding to the *TRIM21* promoter. HEK293T cells were co-transfected with a PGL4-*TRIM21* promoter construct and Flag-*STAT5A*. Luciferase activity was measured 48 h post-transfection (Firefly/Renilla ratio). **f** Correlations between the PI3K/AKT pathway activity score and *STAT5A* mRNA levels in SCLC tissues (HRA003419 dataset, *n* = 107; 95% confidence interval, -0.54 to -0.21). Two-tailed *P* value is reported. **g** Western blot analyses of the key proteins involved in the PI3K/AKT signaling pathway, STAT5A, TRIM21, and PARP1 in DMS273 and SHP77 cells after 24 h treatment with the indicated concentrations of PKI-587. **h** STAT5A binding to the *TRIM21* promoter assessed by ChIP-qPCR in DMS273 and SHP77 cells treated for 24 h with PKI-587 or SC-79. **i** IP-Western blot analysis of PARP1 accumulation and polyubiquitination in DMS273 and SHP77 cells stably expressing sh*STAT5A*, followed by *STAT5A* overexpression rescue (STAT5A). **j** IP-Western analysis of key proteins and the ubiquitination of PARP1. HEK293T cells were co-transfected with HA-Ub, Flag-*PARP1*, EGFP-*TRIM21*, and si*STAT5A*. After 48 h, Flag-IP followed by anti-HA immunoblotting was performed to assess the polyubiquitination of PARP1. **k** PARP1 ubiquitination modulated by AKT and its mutants. HEK293T cells were co-transfected with HA-Ub, Flag-*PARP1*, along with either AKT-WT, AKT-T308D/S473D, or AKT-T308A/S473A plasmids. Following transfection, the ubiquitination of Flag-PARP1 was measured. Data in (**b**, **d**, **e**, **h**) are from three independent experiments and are presented as mean ± SEM. Statistical significance was determined using two-tailed unpaired Student’s *t*-tests. Source data are provided as a Source Data file.

Since the PI3K/AKT pathway suppresses *TRIM21* transcription, we hypothesized that this effect may be mediated through specific transcription factors (TFs). To identify the TF involved in regulating *TRIM21* expression, we overlapped 456 TFs from the AnimalTFDB database predicted to bind to the *TRIM21* promoter with genes downregulated by PI3K/AKT signaling. Six candidate TFs were identified (Fig. 7a). Correlation analysis using an SCLC RNA-seq dataset next revealed significant positive correlations between *TRIM21* and three of these TFs: *STAT5A*, *TCF21*, and *SIX5* (Supplementary Fig. 7a). Subsequent knockdown experiments demonstrated that only *STAT5A* deficiency significantly reduced *TRIM21* mRNA levels without affecting *PARP1* expression (Fig. 7b and Supplementary Fig. 7b). Western blot analysis confirmed that *STAT5A* knockdown decreased TRIM21 protein levels and led to an accumulation of PARP1 (Fig. 7c), suggesting that STAT5A is a key regulator of *TRIM21* transcription.

To further investigate STAT5A’s role in *TRIM21* regulation, we analyzed chromatin immunoprecipitation (ChIP)-seq data from the ChIP-Atlas database, which indicated potential STAT5A binding sites within the *TRIM21* promoter (Supplementary Fig. 7c). Based on STAT5A binding motif (Supplementary Fig. 7d), five putative STAT5A binding sites (BSs) within the *TRIM21* promoter were predicted using the Eukaryotic Promoter Database (EPD) and AnimalTFDB 4.0 (Supplementary Fig. 7e). ChIP followed by qPCR analysis revealed that STAT5A directly binds to BS4 and BS5 in the *TRIM21* promoter region (Fig. 7d). Based on these experimental findings, STAT5A likely binds to an ~300 bp regulatory region flanking the *TRIM21* transcription start site (TSS), spanning specifically from −200 bp upstream to +100 bp downstream of the TSS. Accordingly, we constructed a STAT5A-specific dual-luciferase reporter system and performed corresponding assays, which further confirmed that STAT5A indeed binds to the *TRIM21* promoter region (Fig. 7e).

To elucidate the mechanism through which the PI3K/AKT pathway modulates STAT5A’s transcriptional activity on *TRIM21*, we performed correlation and functional validation experiments. Pearson correlation analysis indicated a negative correlation between PI3K/AKT activity and *STAT5A* mRNA levels (Fig. 7f and Supplementary Fig. 7f), while STAT5A and TRIM21 exhibited a positive correlation at both mRNA and protein levels (Supplementary Fig. 7g, h). Furthermore, PI3K/AKT inhibition using PKI-587 significantly increased both STAT5A transcription and phosphorylation (Fig. 7g and Supplementary Fig. 7i), confirming that PI3K/AKT signaling suppresses STAT5A signaling. Notably, ChIP-PCR showed that PI3K/AKT inhibition enhanced STAT5A binding to the *TRIM21* promoter, whereas PI3K/AKT activation reduced STAT5A binding (Fig. 7h), suggesting that PI3K/AKT signaling controls *TRIM21* transcription by modulating STAT5A’s promoter occupancy.

Following stable *STAT5A* knockdown, TRIM21 expression was significantly reduced at both transcriptional and protein levels, accompanied by decreased polyubiquitination and subsequent accumulation of PARP1 protein. These effects were markedly reversed upon *STAT5A* reconstitution (Fig. 7i and Supplementary Fig. 7j). *STAT5A* knockdown substantially impaired TRIM21-mediated polyubiquitination of PARP1 (Fig. 7j). Similarly, overexpression of AKT, particularly its constitutively active form, but not the inactive variant, suppressed STAT5A phosphorylation, resulting in downregulation of TRIM21 expression and stabilization of PARP1 through reduced ubiquitination (Fig. 7k). In addition, treatment with Stafia-1, a specific inhibitor of STAT5A phosphorylation, markedly suppressed STAT5A activation, thereby reducing TRIM21 expression and ultimately leading to PARP1 accumulation (Supplementary Fig. 7k). Collectively, these results indicate that the PI3K/AKT pathway suppresses STAT5A activity, thereby attenuating TRIM21-mediated polyubiquitination of PARP1 and ultimately promoting its accumulation.

STAT5 consists of two isoforms, STAT5A and STAT5B, which play similar yet non-redundant roles in human cancers^30,31^. We therefore also investigated the role of STAT5B, and the results showed that, similar to STAT5A, PI3K/AKT pathway activity suppresses both transcriptional and protein expression levels of STAT5B in SCLC cells (Supplementary Fig. 8a, b). This is consistent with previous studies demonstrating that treatment with the PI3K/AKT pathway inhibitor PIK-75 upregulates the transcription of both *STAT5A* and *STAT5B* in SCLC^32^ (Supplementary Fig. 8c). Furthermore, *STAT5B* mRNA levels were significantly negatively correlated with PI3K/AKT pathway activity (Supplementary Fig. 8d), indicating that the pathway also transcriptionally represses *STAT5B*. However, unlike *STAT5A*, *STAT5B* knockdown did not alter TRIM21 expression at either the mRNA or protein level (Supplementary Fig. 8e, f), and no correlation was observed between them (Supplementary Fig. 8g). Analysis of ChIP-seq data from the ChIP-Atlas database revealed minimal binding of STAT5B to the *TRIM21* promoter (Supplementary Fig. 8h), which was further confirmed by ChIP-PCR experiments showing no significant binding of STAT5B to this region (Supplementary Fig. 8i). These results collectively indicate that, although *STAT5B* is transcriptionally repressed by the PI3K/AKT pathway, it does not act as a TF for *TRIM21*. Thus, integrating our initial findings on STAT5A-mediated regulation of *TRIM21* transcription, we conclude that while PI3K/AKT suppresses the transcription of both *STAT5A* and *STAT5B*, only STAT5A functions as a TF for *TRIM21*.

### Inhibiting PI3K/AKT signaling and PARPi are synergistic in vitro and in vivo

**Fig. 8:**
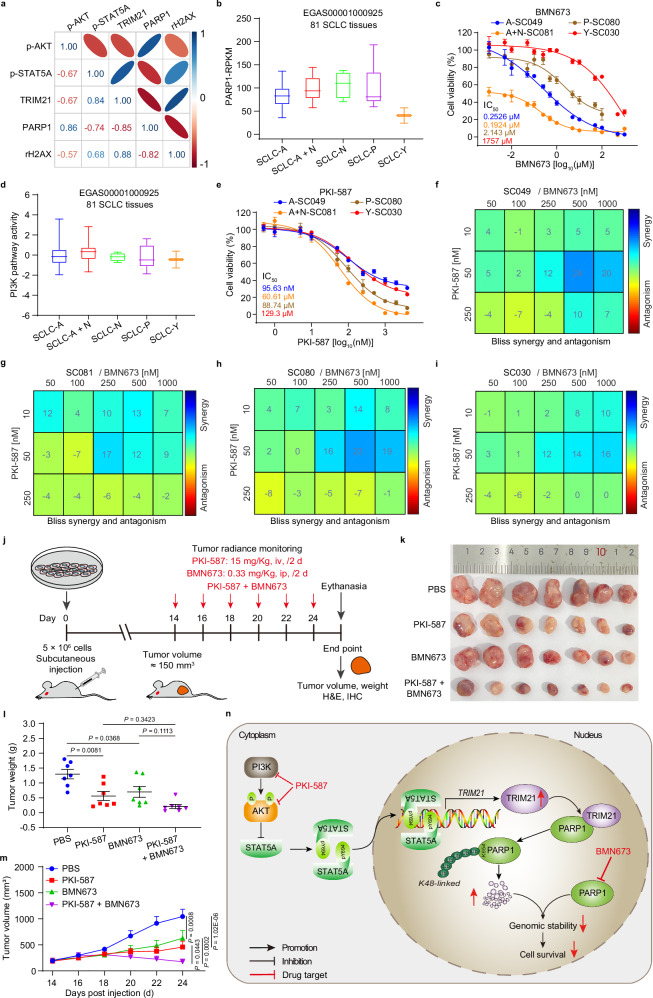
Inhibition of PI3K/AKT potentiates the effect of PARPi. **a** Correlation analysis of key biomarkers in SCLC patient-derived xenograft (PDX) tissues. The expression levels of p-AKT, p-STAT5A, TRIM21, PARP1, and γH2AX were quantified by IHC in 13 SCLC PDX models (as shown in Supplementary Fig. 8j), followed by pairwise correlation analysis. **b** Systematic evaluation of PARP1 expression levels across molecularly defined SCLC subtypes (*A*, *A* + *N*, *N*, *P*, and *Y*) using the EGAS00001000925 dataset. **c** Quantitative determination of BMN673 IC_50_ values through sigmoidal curve fitting of dose-response relationships in subtype-representative SCLC organoid models (*A*: SC049; *A* + *N*: SC081; *P*: SC080; *Y*: SC030). **d** Comparative analysis of PI3K pathway activation scores across SCLC subtypes derived from the EGAS00001000925 dataset. **e** The IC_50_ values of PKI-587 were determined from the sigmoidal dose-response curves in distinct subtypes of SCLC organoid (subtype designations identical to (**c**)). **f**–**i** Bliss synergy analysis of the BMN673 and PKI-587 combination treatment in four molecularly defined SCLC subtypes (*A*: SC049, **f**; *A* + *N*: SC081, **g**; *P*: SC080, **h**; *Y*: SC030, **i**). **j** Schematic representation of the experimental workflow of the xenograft study. When tumors reached approximately 150 mm³, the mice were treated with PKI-587, BMN673, or a combination of both. Each experimental group consisted of 7 mice. **k** Representative images of xenograft tumors. **l** Scatter plot depicting tumor weights from xenograft mice at the end points of the experiments. **m** Tumor volume curves of xenograft mice. Data in (**b**, **d**) are presented as box plots showing the median (center line), the 25th and 75th percentiles (box bounds), and whiskers extending to the most extreme data points within 1.5 × interquartile range (IQR) from the box. Data in (**c**, **e**) are from three independent experiments, and data in (**l**, **m**) are from 7 mice per group. All data are presented as mean ± SEM in (**c**, **e**, **l**, **m**). Statistical analysis was performed using two-tailed unpaired Student’s *t*-tests in (**l**, **m**). **n** A proposed model depicting the “PI3K/AKT-STAT5A-TRIM21-PARP1” signaling axis. Source data are provided as a Source Data file.

To validate the mechanism we identified, we performed additional IHC analyses on 13 SCLC PDX models. The results demonstrated consistently high expression levels of p-AKT and PARP1, while p-STAT5A, TRIM21, and γH2AX generally showed low expression patterns (Supplementary Fig. 8j). Furthermore, negative correlations were observed between p-AKT and p-STAT5A, as well as between p-AKT and TRIM21. In contrast, a positive correlation was found between p-STAT5A and TRIM21, whereas TRIM21 and PARP1 were negatively correlated (Fig. 8a). These findings strongly support our proposed mechanistic model wherein PI3K/AKT signaling suppresses *TRIM21* transcription through the TF STAT5A, thereby reducing TRIM21-mediated PARP1 ubiquitination and degradation. This process leads to PARP1 accumulation and subsequently maintains genomic stability.

PI3K/AKT pathway activation has been linked to reduced sensitivity to the PARPi Talazoparib (BMN673)^33^. We hypothesized that inhibiting PI3K/AKT could enhance PARPi cytotoxicity in SCLC via the STAT5A-TRIM21 axis. Based on the regulation of PARP1 by PI3K/AKT, we investigated combined inhibition of the PI3K/AKT pathway and PARP to maximize therapeutic efficacy in SCLC. We first determined the half-maximal inhibitory concentration (IC_50_) of both PARP inhibitor BMN673 and the PI3K/AKT inhibitor PKI-587 in different subtypes of SCLC cell lines, including *N*-subtype (DMS273), *A*-subtype (SHP77), *P*-subtype (H526), and *Y*-subtype (DMS114) (Supplementary Fig. 9a, b). Importantly, combined treatment with PKI-587 and BMN673 resulted in significantly enhanced therapeutic effects compared to BMN673 alone (Supplementary Fig. 9c–f). Bliss independence analysis further demonstrated that the combination produced strong synergistic effects at optimized doses in DMS273, SHP77, H526, and DMS114 cells in vitro (Supplementary Fig. 9g–n). These findings indicate that the synergistic interaction between these two drug classes occurs independently of SCLC subtypes when appropriately dosed.

We further evaluated the effects of BMN673 and PKI-587 using patient-derived organoid models representing four distinct SCLC subtypes: *A* subtype (SC049), *A* + *N* subtype (SC081), *P* subtype (SC080), and *Y* subtype (SC030). PARP1 expression was significantly higher in *A* and *A* + *N* subtypes compared to the *P* subtype, with the lowest expression observed in the *Y* subtype (Fig. 8b). Correspondingly, sensitivity to BMN673 was highest in *A* and *A* + *N* subtypes, intermediate in the *P* subtype, and lowest in the *Y* subtype (Fig. 8c), establishing a clear correlation between PARP1 expression levels and therapeutic response to BMN673 across molecular subtypes. In contrast, PI3K signaling activity remained consistent across all subtypes, with no significant differences in basal pathway activation or response to PKI-587 (Fig. 8d, e), suggesting that sensitivity to PI3K/AKT inhibition is independent of SCLC molecular classification. Most importantly, therapeutic synergy between BMN673 and PKI-587 was consistently observed across all SCLC subtypes in patient-derived organoid models (Fig. 8f–i and Supplementary Fig. 10a–d), supporting the broad clinical applicability of this combination strategy regardless of tumor subtype.

Encouraged by these in vitro and organoid results, we assessed the therapeutic efficacy of PKI-587 and BMN673 in an SCLC xenograft model (Fig. 8j). The combination therapy significantly inhibited tumor size and volume, and slowed the rate of tumor growth, with tumor regression observed exclusively in the combination group (Fig. 8k–m). None of the treatments caused significant body weight loss (Supplementary Fig. 10e), indicating minimal systemic toxicity of PKI-587, BMN673, or their combination in vivo. IHC analysis revealed decreased Ki67, p-AKT, and PARP1-positive cells in the combination group, indicating reduced proliferation and PI3K/AKT activity. Concurrent increases in p-STAT5A, TRIM21, and γH2AX-positive cells highlighted enhanced activation of the STAT5A-TRIM21 axis and DNA damage (Supplementary Fig. 10f). Collectively, these findings underscore the prolonged synergistic anti-tumor effects of combining PKI-587 with BMN673 in SCLC xenografts, supporting its therapeutic potential for SCLC treatment.

## Discussion

A comprehensive understanding of the mechanisms underlying PARP1 stability offers valuable insights for developing combination therapies targeting PARP1^34,35^. The study identifies TRIM21, an E3 ubiquitin ligase, as a critical regulator of PARP1 stability, and it interacts directly with PARP1 via the TRIM21-PP domain and the PARP1 662-908 domain. This interaction facilitates PARP1 ubiquitination and subsequent degradation. Downregulation of TRIM21 in SCLC enhances cell survival and tumor growth in vitro and in vivo, suggesting that TRIM21’s role in PARP1 ubiquitination and degradation is functionally significant for tumor suppression. Furthermore, TRIM21 expression is negatively regulated by the PI3K/AKT pathway, which represses STAT5A, a positive TF of *TRIM21* (Fig. 8n). In summary, the “PI3K/AKT-STAT5A-TRIM21-PARP1” signaling axis is implicated in promoting tumor growth in SCLC, and targeting this axis may potentially maximize the therapeutic effects of PARPi against SCLC.

PTMs are essential for modulating protein stability, activity, or interactions^36,37^. Among these, ubiquitination is pivotal in numerous physiological and pathological processes^38,39^. In this study, we identified TRIM21 as an E3 ligase specific for PARP1. Unlike other E3 ubiquitin ligases identified for PARP1^40,41^, TRIM21 facilitates PARP1 polyubiquitination and degradation under both physiological and genotoxic conditions. TRIM21 interacts with PARP1 through a unique region, distinct from the domain targeted by other known E3 ligases. For example, RNF4^40^, Smurf2^15^, and WWP2^41,42^ interact with the BRCT domain of PARP1, CHFR^13^ targets the auto-modification (AD) domain, and HECTD3^43^ associates with the DNA binding domain (DBD). Our findings indicate that TRIM21 specifically interacts with the 662-908 region of PARP1, underscoring that distinct E3 ligases target specific PARP1 domains, thereby modulating its polyubiquitination and subsequent degradation. PARP1 has multiple polyubiquitination sites^44^, and our study reveals that TRIM21 mediates polyubiquitination of PARP1 at the K654 site, located near the 662-908 region. This site differs from those previously identified for WWP2 and other E3 ligases^41,42,45^, marking K654 as a ubiquitination site for PARP1. This suggests that diverse E3 ligases may differentially regulate PARP1’s polyubiquitination in response to varying cellular stress and non-stress conditions. Further investigation is needed to determine whether additional E3 ligases and unidentified ubiquitination sites might contribute to PARP1’s degradation, broadening our understanding of PARP1 regulation in cellular responses.

The PP domain of TRIM21 is recognized for its specific binding to the Fc region of antibodies, a mechanism that is crucial for innate immune activation during viral infections^25^. In our study, we discovered that PARP1, a newly identified substrate of TRIM21, also interacts with TRIM21’s PP region. Emphasizing the functional importance of the PP region in TRIM21-substrate interactions. Notably, two hot-spot residues in TRIM21 (W381 and W383) within the PP domain are essential for Fc binding and for interacting with substrates such as p62; mutation of either residue disrupts these interactions^25^. Intriguingly, our findings indicate that these residues are not required for PARP1 binding, suggesting that while most TRIM21 substrates bind to the PP region, the critical amino acids involved may vary depending on the substrates. This leaves the key residues critical for TRIM21-PARP1 binding to be further defined. TRIM21 functions as an E3 ubiquitin ligase, targeting various antibody-coated pathogenic substrates for degradation^46,47^. Its RING domain confers this enzymatic activity, with residues C16, C31, and H33 forming the core group necessary for its catalytic function^48^. We confirmed that TRIM21-mediated polyubiquitination of PARP1 is dependent on these catalytic residues. Beyond K48-linked polyubiquitination, TRIM21 can also mediate K63-linked polyubiquitination, which serves to modulate substrate functions rather than drive proteasomal degradation. For instance, TRIM21 mediates K63-linked ubiquitination of p62, which prevents p62 dimerization and negatively regulates the Keap1-Nrf2 redox axis^26^. Additionally, TRIM21 suppresses CHK1 activation by preferentially targeting CLASPIN for K63-linked ubiquitination, leading to replication fork instability and promoting tumorigenesis^49^. These findings underscore TRIM21’s dual ability to induce substrate degradation and modulate substrate functions. Whether TRIM21 can mediate additional ubiquitination types for PARP1, such as K63-linked polyubiquitination, warrants further investigation, as such modifications could have distinct functional impacts on PARP1. Given TRIM21’s significant roles in both innate and tumor immunity^50,51^, TRIM21-mediated ubiquitination and degradation of PARP1 are likely to contribute to maintaining immune homeostasis. This points to a possible mechanism by which dysfunction in TRIM21-mediated pathways could enable immune evasion in tumors.

Emerging research highlights TRIM21’s complex role in tumor development and prognosis^52^. In breast cancer^20^ and renal carcinoma^22^, TRIM21 appears to function as a tumor suppressor, as its expression is often reduced. Conversely, TRIM21 is upregulated in glioma^23^ and liver cancer^24^, where it has been associated with oncogenic activity. Our study reveals that TRIM21 is markedly downregulated in SCLC, where it appears to inhibit SCLC cell viability by promoting PARP1 ubiquitin-mediated proteolysis. This aligns with other findings where TRIM21 suppresses tumor progression by ubiquitinating G6PD^53^. Thus, TRIM21’s function as a tumor suppressor or oncogene may depend on various factors, including tumor types and specific substrate interactions.

While the PI3K/AKT pathway’s role in suppressing TRIM21 expression at the transcriptional level has been established^53^, the exact mechanism was previously unclear. Our results show a negative correlation between TRIM21 expression and PI3K pathway activity in SCLC. Furthermore, we identified the TF STAT5A as a key downstream mediator of PI3K/AKT-mediated suppression of TRIM21 transcription. While prior studies have established STAT5 as an upstream activator of PI3K/AKT signaling by interacting with the p85 subunit of PI3K and enhancing AKT transcription in breast cancer^54,55^, our findings reveal that STAT5A also operates downstream of PI3K/AKT, with its transcriptional activity being negatively regulated by PI3K/AKT pathway activation in SCLC. These collective findings suggest the existence of a sophisticated and potentially bidirectional regulatory crosstalk between STAT5 and PI3K/AKT signaling, which may involve previously unrecognized feedback mechanisms. Furthermore, the interplay between STAT5 and PI3K/AKT pathways may exhibit tumor-specific characteristics. This understanding elucidates the upstream regulation of TRIM21-mediated PARP1 degradation, defining a “PI3K/AKT-STAT5A-TRIM21-PARP1” signaling axis that may serve as a therapeutic target in SCLC (Fig. 8n).

STAT5A and its closely related paralog STAT5B are key components of the Janus kinase (JAK)/STAT signaling pathway, which can be activated by cytokines and growth factors^56,57^. Upon activation, STAT5A undergoes phosphorylation on tyrosine residues by upstream kinases, leading to conformational changes that allow it to form active parallel dimers^30,58^. These dimers then translocate to the nucleus, where STAT5A binds to promoters and regulates the expression of target genes^59^. Our study reveals that the PI3K/AKT pathway negatively regulates STAT5A activation, suggesting that STAT5A acts as a mediator integrating multiple signaling pathways. Interestingly, although STAT5B transcription is also suppressed by the PI3K/AKT pathway, it does not participate in the transcriptional regulation of *TRIM21*. This indicates that the PI3K/AKT-mediated suppression of *TRIM21* transcription is specifically dependent on STAT5A homodimers, rather than STAT5A-STAT5B heterodimers. The PI3K/AKT signaling network exerts diverse downstream effects, primarily through the direct regulation of kinase activity or modulation of TFs^59^. Several TFs regulated by the PI3K/AKT pathway have been identified, including FOXO, ATF4, and MYC^60^. Our findings highlight STAT5A as a downstream TF within this pathway, furthering our understanding of the key effectors involved.

PARP inhibition has emerged as a promising therapeutic strategy in SCLC^7,61^. The mechanism of PARPi in SCLC appears to differ from its action in ovarian and breast cancers, where sensitivity is primarily driven by BRCA mutations^62^. In SCLC, BRCA mutations are rare, occurring in only 3–4% of cases^63^, and most patients do not experience significant benefit from PARPi as a monotherapy. Ongoing clinical trials are investigating combinations of PARPi with other small molecules in SCLC patients, although these have yet to demonstrate substantial improvements in median overall survival^64^. Therefore, there is an urgent need to explore combination strategies to enhance the efficacy of PARPi in SCLC. One potential approach involves combining PARPi with PI3K/AKT pathway inhibitors (PI3K/AKTi)^33,65–67^. However, the underlying mechanisms remain poorly understood. Our study preliminarily confirms the feasibility of sensitizing SCLC to PARPi through targeted inhibition of the PI3K/AKT pathway, using the combination of the PARPi BMN673 and the PI3K/AKTi PKI-587. We propose that the enhanced efficacy of this combination may be attributed to modulating the activation status of “PI3K/AKT-STAT5A-TRIM21-PARP1” signaling axis, a mechanism that provides theoretical support for the combined use of PI3K/AKTi and PARPi in cancer therapy. Previous studies in breast cancer have demonstrated that TRIM21 enhances PARPi sensitivity by promoting BRCA1 degradation^68^. Together, these studies highlight TRIM21 as a potentially crucial regulator of PARPi sensitivity across multiple cancer types. Furthermore, TRIM21 may exert anti-tumor effects by targeting substrate protein degradation^69^. PKI-587 treatment could transcriptionally upregulate TRIM21, thereby promoting PARP1 degradation and suppressing its oncogenic functions. Thus, modulation of the TRIM21-PARP1 regulatory axis contributes to, at least in part, the observed synergistic effect of BMN673 and PKI-587. Inhibiting the PI3K/AKT pathway may thus offer a promising strategy to overcome resistance and enhance the anti-tumor activity of PARPi in SCLC and potentially other cancers.

This study has some limitations. For instance, while PARP1 and other PARP family proteins (such as PARP2 or PARP3) share a similar *C*-terminus^70,71^, it remains unclear whether TRIM21 is a universal binding protein required for the stabilization of other PARP family proteins beyond PARP1. Furthermore, as an important E3 ligase, TRIM21 ubiquitinates multiple substrates through different ubiquitination modes, thereby exerting diverse biological functions, including the ubiquitin-mediated degradation of VDAC2^72 ^and SAMHD1^73^, autophagic degradation of c-Myc^74^ and elimination of stress granules^75^, and K63-linked ubiquitination of p62 to inhibit its dimerization^26^, among others. Our findings that PARP1 only partially rescues TRIM21-mediated suppression of SCLC further indicate that TRIM21 likely targets additional substrates beyond PARP1. These results underscore the necessity for future studies to systematically dissect the network of multi‑substrate ubiquitination by TRIM21 in SCLC.

In conclusion, our study elucidates the crucial molecular mechanism of the “PI3K/AKT-STAT5A-TRIM21-PARP1” signaling axis and its significant role in SCLC progression. Dual inhibition based on this signaling axis may potentially benefit SCLC treatment. Moreover, this approach could be particularly beneficial in overcoming resistance to PARPi, which is a current challenge in cancer therapy.

## Methods

### Ethical statement

This research complies with all relevant ethical regulations. All animal experiments were conducted in accordance with a protocol approved by the Institutional Animal Care and Use Committee of Longgang District People’s Hospital of Shenzhen (No. 2024003DW). Written informed consent was obtained from all patients whose SCLC tissues were used in this study, and the study was approved by the Medical Ethics Committee of Shanghai Pulmonary Hospital, Tongji University Medical School Cancer Institute (ethical approval Nos. K19-159 and K21-019).

### Cell lines, antibodies, and reagents

The SCLC cell lines DMS273 and SHP77 were kindly provided by Dr. Matthew Meyerson (Dana-Farber Cancer Institute, USA). The SCLC cell lines H196, H526, and DMS114 were kindly provided by Dr. Hongbin Ji (Key Laboratory of Multi-Cell Systems, Shanghai Institute of Biochemistry and Cell Biology, Center for Excellence in Molecular Cell Science, Chinese Academy of Sciences). HeLa and HEK293T cells were generously provided by Dr. Ge Shan (University of Science and Technology of China). All other cell lines, including A549, PC9, AGS, and MKN45, were obtained from and maintained in our laboratory repository. DMS273, SHP77, H196, H526, DMS114, A549, PC9, AGS, and MKN45 cells were routinely maintained in RPMI 1640 medium (Gibco, #11875093). HeLa and HEK293T cells were cultured in DMEM medium (Gibco, #11995065). All cell lines were supplemented with 10% fetal bovine serum (FBS) (VivaCell, #C04002) and 1% penicillin/streptomycin (PS) (Servicebio, #G4003), and incubated at 37 °C in a humidified atmosphere with 5% CO_2_. All cell lines were regularly tested for mycoplasma contamination using a mycoplasma detection kit (Beyotime, #C0301S) and authenticated by short tandem repeat (STR) profiling. All antibodies and chemical compounds used in this study are listed in Supplementary Data 2.

### Chemical preparation and cell treatments

Cycloheximide (CHX) was dissolved in DMSO to prepare a 100 mg/ml stock solution and used at 100 μg/ml. EP, CPT, GDC-0068, and BEZ235 were dissolved in DMSO to prepare stock solutions of 40 mM, 1 mM, 10 mM, and 200 μM, respectively, and used at final concentrations of 40 μM, 1 μM, 10 μM, and 200 nM. Talazoparib (BMN673), PKI-587, SC-79, and Stafia-1 were dissolved in DMSO to prepare 10 mM, 1 mM, 10 mM, and 10 mM stock solutions and used at various gradient concentrations. Freshly diluted 30% H_2_O_2_ in 1× PBS (2 mM) was added directly to cells at a final concentration of 2 μM.

### Plasmid constructs

For ectopic expression experiments, human *TRIM21* cDNA was cloned into the pcDNA3.1-EGFP vector (Addgene, #129020) to generate an EGFP-*TRIM21* fusion plasmid. Similarly, human *STAT5A* cDNA was inserted into the pCMV-*N*-3× Flag vector (Beyotime, #D2722) to generate a Flag-tagged *STAT5A* expression construct. pCMV-*PARP1*-3x Flag-WT was purchased from Addgene (#111575). The following three classes of mutation plasmids: 1) the truncated mutation plasmids pcDNA3.1-EGFP-*TRIM21* ΔRING (1-53), ΔBB (54-145), ΔCC (146-255) and ΔPRY-SPRY (256-475); 2) the truncated mutation plasmids of pCMV-*PARP1* Δ662-908, pCMV-*PARP1* ΔH3ID, and pCMV-*PARP1* Δ645-908; 3) the point mutation plasmids of pcDNA3.1-EGFP-*TRIM21* LD (C16A, C31A and H33W), pcDNA3.1-EGFP-*TRIM21* W381A/W383A, and pCMV-*PARP1* K654R were constructed using QuickMutation™ Site-Directed Mutagenesis Kit (Beyotime, #D0206M) according to the manufacturer’s instructions. pCMV-HA-Ub, pCMV-HA-Ub (K48R), and pCMV-HA-Ub (K63R) plasmids were purchased from HedgehogBio Science and Tecgnology Ltd.

For prokaryotic expression vectors, cDNA expressing full-length *TRIM21* was cloned into pEXG-4T-1 (Addgene, #27-4580-01) to construct GST-tagged *TRIM21*-expressing plasmids in *E.coli*. pET-28a-*PARP1*(FL) and truncated mutants (ZF1, ZF2, ZF3, BRCT, ∆BRCT, WGR, WGR-CAT, CAT, and ART) plasmids were a gift from Caihong Yun (Peking University). An *N*-terminal 6x His-tag followed by a tobacco etch virus (TEV) protease cleavage site (ENLYFQG) was added to the *N*-terminus of the constructs to facilitate protein purification. All expression vectors were confirmed by DNA sequencing before use.

### Virus package and construction of stable cell lines

To establish stable overexpression cell lines, the *TRIM21* and *STAT5A* coding sequences were amplified by PCR and cloned into the pCDH-UbC-MCS-EF1-Hygro (Addgene, #129436) and pCDH-CMV-MCS-EF1-copGFP-T2A-Puro (General Biology Co., Ltd.) lentiviral vectors, respectively. The short hairpin RNA (shRNA) sequence was subcloned into the pLKO.1-puro (Addgene, #8453, sh*TRIM21* and sh*PARP1*) or pLKO.1-Hygro (Addgene, #24150, sh*STAT5A*) lentiviral vector. The target sequences for all shRNAs used in this study are provided in Supplementary Data 3. For viral particle generation, cloned DNAs, psPAX2 (Addgene, #12260), and pMD2.G (Addgene, #12259), were transfected into HEK293T cells using the Effectene Transfection Reagent (QIAGEN, #301425) according to the manufacturer’s instructions. During all virus production, the media was changed 8 h post-transfection, and the virus was harvested after 48 h and filtered. For viral infection, 1.5 × 10^5^ cells were added to each well of a 6-well plate and incubated overnight. The viral supernatant was added to the plated cells in the presence of 8 μg/mL polybrene, and the selection of resistant colonies was initiated 48 h later with 200 μg/mL hygromycin or 2 μg/mL puromycin for 48 h. The resulting cell clones were subsequently validated by Western blot analysis.

### RNA Isolation and quantitative RT-PCR

Total RNA was extracted using the RNeasy Mini Kit (TransGen Biotech, #ER101) and subsequently synthesized into single-stranded cDNA using Evo M-MLV RT Mix Kit (Accurate Biology, #AG11728) in accordance with the manufacturer’s instructions. Quantitative real-time PCR reactions were performed in triplicate using the SYBR Green Premix Pro Taq HS qPCR Kit (Accurate Biology, #AG11718). Relative gene expression levels were normalized to β-Actin expression. At least three independent biological replicates were included in each qPCR. Primer sequences used are summarized in Supplementary Data 3.

### Cell viability assays

For cell viability assays, cells were seeded into 96-well plates at 3000 cells per well. After 24 h, cells were treated with corresponding drugs or controls for 72 h. Cell viability was determined using the CellTiter-Glo luminescence kit (Promega, #G7570). The ATP level in untreated cells was defined as 100%. The percentage of cell viability at each concentration was calculated against the respective control. The clonogenic assay was also performed to assess long-term cell survival. In this assay, 1000–2000 cells were seeded per well in a 12-well plate and cultured in complete medium for 7–10 days until visible colonies formed. The cells were then washed twice with PBS, fixed with methanol, and stained with 1% crystal violet solution for visualization. Quantification of clonogenicity was performed^76^. In brief, to assess clonogenic self-renewal capacity, the results were expressed as relative colony formation rate compared to the control group. For evaluating cytotoxic drug treatments, the data were presented as clonogenic survival relative to the control.

### Western blotting

Total protein extracts were prepared using a lysis buffer composed of 150 mM NaCl, 50 mM Tris-HCl (pH 8.0), 1% Triton X-100, and 1 mM EDTA, supplemented with a cocktail of protease and phosphatase inhibitors (Roche). After clarification by centrifugation, the concentration of the total protein was quantified using a bicinchoninic acid assay kit (Servicebio, #G2026). An aliquot of 20 μg of protein was subjected to separation by SDS-PAGE and subsequently transferred onto an Immobilon-P PVDF membrane (Millipore, #IPVH00010). The membranes were incubated with primary antibodies, followed by incubation with HRP-conjugated anti-mouse or anti-rabbit IgG secondary antibodies. After 15 min of washing in TBST, signal detection was carried out using the Ncm ECL Ultra kit (New Cell & Molecular, #P10300) and visualized with a chemiluminescence photodocumentation system. The following antibodies were used in this study: anti-PARP1 (Active Motif, 39559, 1:2000), anti-TRIM21 (Abcam, ab207728, 1:1000), anti-AKT (CST, 9272, 1:1000), anti-p-AKT (Ser473) (CST, 4060, 1:1000), anti-STAT5A (Abcam, ab32043, 1:1000), anti-p-STAT5A (Tyr694) (Abcam, ab106095, 1:1000), anti-GFP-Tag (Abcam, ab290, 1:1000), anti-Myc-Tag (CST, 2276S, 1:1000), anti-GST-Tag (Affinity, T0007, 1:1000), anti-β-Actin (Affinity, T0022, 1:2000), anti-HA-Tag (Affinity, T0008, 1:1000), anti-His-Tag (Affinity, T0009, 1:1000), anti-Ubiquitin (CST, 3936S, 1:1000), anti-RPA32 (CST, 35869S, 1:1000), anti-p-RPA32 (CST, 83745S, 1:1000), anti-CHK1 (CST, 2360S, 1:1000), anti-p-CHK1 (CST, 12302S, 1:1000), anti-STAT5B (Abcam, ab178941, 1:1000).

### Co-Immunoprecipitation (Co-IP) assay

Cells were lysed in lysis buffer (20 mM Tris-HCl, pH 7.5, 150 mM NaCl, 1% Triton X-100, 1 mM EDTA, supplemented with a cocktail of protease and phosphatase inhibitors (Roche)), and clarified by centrifugation at 12,000 × *g* for 10 min at 4 °C. For each IP experiment, 500 μg of total protein was used, and primary antibodies were added, followed by incubation overnight at 4 °C on a rotator. Protein A/G magnetic beads (Thermo Fisher Scientific, #80105G) were added and incubated at 4 °C for 4 h. The beads were then washed three times with 0.5 mL of lysis buffer. For the resulting IP complexes, if the primary antibody used was a Flag antibody, competitive elution was performed using 100 μL of 3× Flag peptide elution solution (150 μg/mL), with incubation on ice and gentle shaking for 1 h. The mixture was then centrifuged at 6000 × *g* for 30 s at 4 °C, and the supernatant, containing the eluted target protein complex, was collected. For IP experiments conducted with other primary antibodies, the beads were directly boiled in 1× SDS loading buffer, followed by detection via Western blotting.

### Mass spectrometry (MS) analysis

Protein identification by MS was conducted at Shanghai Bioprofile Technology Co., Ltd., following previously described methods with minor modifications^77,78^. Briefly, HEK293T cells were subjected to IP using both a PARP1 antibody (Active Motif, 39559) or a control IgG antibody (GeneTex, GTX26702). The IP proteins were eluted via SDS-PAGE gel and visualized by Coomassie staining. Lysates were boiled for 5 min in SDT buffer (4% SDS, 100 mM DTT, 100 mM Tris-HCl), cooled, and centrifuged to collect the supernatant. UA buffer (8 M urea, 150 mM Tris-HCl, pH 8.0) was added, and the mixture was transferred to 10 kDa molecular filters and centrifuged. The filters were washed again with UA buffer, alkylated with 50 mM iodoacetamide (in UA buffer), centrifuged, and subsequently washed twice with UA buffer followed by two washes with NH₄HCO₃ buffer. Trypsin digestion (6 µg in 40 µL NH₄HCO₃ buffer) was carried out at 37 °C for 16–18 h. The resulting peptides were collected, desalted, and dried. The dried peptides were reconstituted in 0.1% formic acid. For LC-MS/MS analysis, the digested peptide mixtures were separated using a nano-flow Easy nLC 1200 system (Thermo Fisher Scientific) with mobile phases consisting of solvent A (0.1% formic acid in water) and solvent B (0.1% formic acid in 80% acetonitrile/20% water). Samples were loaded onto a Trap Column (100 µm × 20 mm, C18) and then separated on an analytical column (75 µm × 150 mm, C18) using a gradient elution at a constant flow rate of 300 nL/minute. Peptide separation was followed by analysis on a Q-Exactive HF-X mass spectrometer (Thermo Fisher Scientific). The full scan range was set from 350 to 1800 m/z with a resolution of 120,000 @ m/z 200, an AGC target of 3e6, and a maximum injection time of 50 ms for MS1. For MS2 acquisition, the top 20 most intense precursor ions were selected for HCD fragmentation with an isolation window of 1.6 m/z and a normalized collision energy of 28. MS2 scans were acquired with a resolution of 15,000 @ m/z 200, an AGC target of 1e5, and a maximum injection time of 50 ms. Raw data were processed using MaxQuant 1.6.17.0 against the UniProt database (*Homo sapiens*) with the following parameters: enzyme: trypsin; maximum missed cleavages, 2; fixed modification: carbamidomethyl (C); variable modifications: oxidation (M) and acetyl (protein *N*-terminus); MS/MS tolerance: 20 ppm; database pattern: reverse; and peptide/protein/site FDR ≤ 0.01. For the identification of PARP1-interacting proteins, biological replicates were not performed.

### Immunostaining and confocal microscopy

An immunofluorescence assay was performed^79^. Briefly, cells were seeded on the glass bottom of a cell culture dish. After 11–12 h, cells were treated, washed once with ice-cold PBS, and fixed with 4% paraformaldehyde solution for 20 min at RT. After being washed with PBS three times, cells were permeabilized with 0.1% Triton X-100 for 10 min. The coverslips were washed three times with PBS, blocked with 5% BSA for 1 h, then incubated with primary antibodies (TRIM21, 1:400; PARP1, 1:400; γH2AX, 1:500) at 4 °C overnight. Cells were rinsed with PBS four times and incubated with secondary antibodies (diluted 1:1,000 in 5% BSA solution) for 1 h in the dark. The coverslips were then rinsed with PBS four times, taken out, fixed onto slides with ProLong Diamond antifade mountant containing DAPI (Thermo Fisher Scientific, #P36966), and observed with laser scanning confocal microscopes. Quantifications were performed using ImageJ software.

### In situ PLA assay

In situ proximity ligation assays (PLA) were performed using the Duolink in situ PLA assay reagent (Sigma-Aldrich, #DUO92008), following the manufacturer’s instructions. Briefly, cells cultured on confocal plates were fixed with 4% PFA in PBS for 15 min. After three washes with PBS, the cells were permeabilized with 0.5% Triton X-100 in PBS for 15 min and blocked with 3% BSA in PBS for 1 h at room temperature (RT). Cells were then double-stained with primary antibodies against PARP1 and TRIM21, and incubated overnight at 4 °C. Afterward, the slides were washed twice for 5 min each in wash buffer A (0.01 M Tris, 0.15 M NaCl, 0.05% Tween-20, pH 7.4) on a laboratory shaker. Cells were incubated with Duolink in situ PLA Probe Anti-Rabbit PLUS (Sigma-Aldrich, #DUO92002) and Duolink in situ PLA Probe Anti-Mouse MINUS (Sigma-Aldrich, #DUO92004) for 1 h at 37 °C in a humidity chamber. After washing, ligation was performed with ligase solution for 30 min at 37 °C to allow the hybridization of oligonucleotide-tagged probes. Following two brief washes, cells were incubated with the amplification polymerase solution for 120 min at 37 °C to amplify the hybridized oligonucleotides and label the amplification products with fluorescence. The slides were then washed twice for 10 min in wash buffer B (0.2 M Tris, 0.1 M NaCl, pH 7.5), allowed to dry, and mounted with ProLong Diamond antifade mountant containing DAPI (Thermo Fisher Scientific, #P36966). Imaging was conducted using a confocal microscope.

### Protein stability assay

Cells were seeded in a 6-well plate one day before the experiments. After 24 h, fresh medium with 100 μg/ml CHX was added to the cells, which were incubated and harvested for 0, 3, 6, 12, 18, and 24 h. Cell pellets were stored at -80 °C until all samples were collected. One well of cells immediately before adding CHX was harvested as a control. Cell pellets were lysed in RIPA buffer supplemented with protease and phosphatase inhibitors (Roche) and subjected to standard SDS-PAGE and Western blot analysis with the indicated antibodies.

### Chromatin immunoprecipitation (ChIP) assays

Each ChIP experiment utilized approximately 1.0 to 4.0 × 10^6^ cells. Cells were cross-linked with 1% formaldehyde for 10 min at RT. The formaldehyde cross-linking was then quenched by adding glycine to a final concentration of 125 mM. Cells were resuspended in ChIP Lysis Buffer (50 mM HEPES-KOH pH 7.5, 140 mM NaCl, 1 mM EDTA pH 8.0, 1% Triton X-100, 0.1% Sodium Deoxycholate, 0.5% SDS, Protease Inhibitors) and incubated on ice for 10 min. Chromatin was sheared to an average DNA size of approximately 200 bp using an Ultrasonic Homogenizer JY92-IIN (SCIENTZ). The chromatin solution was then diluted 10-fold in ChIP Dilution Buffer and incubated with 50 μL of protein A/G beads (Thermo Fisher Scientific, #80105 G) pre-loaded with 2 μg of antibody overnight on a rotator at 4 °C. The beads were subjected to the following washes: once with Low Salt Wash Buffer (0.1% SDS, 1% Triton X-100, 2 mM EDTA, 20 mM Tris-HCl pH 8.0, 150 mM NaCl), once with High Salt Wash Buffer (0.1% SDS, 1% Triton X-100, 2 mM EDTA, 20 mM Tris-HCl pH 8.0, 500 mM NaCl), and once with LiCl Wash Buffer (0.25 M LiCl, 1% NP-40, 1% Sodium Deoxycholate, 1 mM EDTA, 10 mM Tris-HCl pH 8.0). The immunoprecipitated protein-DNA complexes were eluted from the beads, and cross-links were reversed by adding 5 M NaCl and Proteinase K, followed by incubation for 2 h at 65 °C. The DNA was then purified using a SteadyPure PCR Purification Kit (Accurate Biology, #AG21003). Primer sequences are listed in Supplementary Data 3.

### Protein expression and purification

For expression and purification of protein in a prokaryotic system^80,81^, human *TRIM21*, *PARP1* FL, and truncation variants were expressed in *E. coli* BL21(DE3) to gain recombinant proteins. Cells were grown at 37 °C in LB medium until reaching an OD_600_ of 0.6 to 0.8. Expression was then induced with 0.5 mM isopropyl-d-1-thiogalactopyranoside (IPTG) (GlpBio, #GC30002) at 18 to 20 °C for 12 to 16 h. Following the expression, bacterial cells were harvested by centrifugation and snap-frozen in liquid nitrogen. GST-TRIM21 fusion proteins were purified using a commercially available kit of BeyoGold™ GST-tag Purification Resin (Beyotime, #P2253) according to the manufacturer’s instructions. Briefly, for fresh bacterial deposits, add lysis buffer at a ratio of 2 to 5 ml per gram of wet weight of bacteria to fully resuspend bacteria, and then add lysozyme until the final concentration is 1 mg/mL on ice for 30 min. Ultrasonic lytic bacteria on ice, with an ultrasonic power of 200–300 W, and each treatment was 10 s/interval of 10 s, for a total of 6 treatments, followed by centrifuging at 10,000 × g at 4 °C for 20–30 min, the supernatant of bacterial lysate was collected and placed on ice. Take an appropriate amount of BeyoGold™ GST-Tag Resin, which is evenly mixed, and centrifuge it at 4 °C (1000 × g, 10 s) to discard the storage solution. BeyoGold™ GST-tag Purification Resin was mixed with 4 ml supernatant of bacterial lysis solution in a ratio of 1:8 per 0.5 ml gel, and shaken slowly on a side or horizontal shaker for 60 min at 4 °C; the mixture of the lysate and BeyoGold™ GST-Tag Purification Resin was filled into the appropriate empty column tube, and make the liquid in the column flow out under the action of gravity, and the column was washed 5 times, and each time 1–2 column volume of lysis buffer was added. Elute the target protein 6–10 times, each time using a column volume of eluting buffer. The eluent collected is the purified GST label protein sample. To purify PARP1-FL and truncated mutants, clarified lysates were applied to Ni^2+^-agarose, depending on the tag system, using a gravity column for 1 to 2 h on a rotary shaker at 4 °C. The assays were performed using a commercially available His-tag Protein Purification Kit (Beyotime, #P2229S) according to the manufacturer’s instructions. The specific method was similar to the above purification method of the GST fusion protein. To remove tags, protein samples were dialyzed against a buffer (25 mM Tris-HCl (pH 7.6), 150 mM NaCl, and 5 mM BME) containing TEV or thrombin protease overnight at 4 °C. The proteins of interest were separated from the affinity tags or the remaining uncleaved proteins by applying the dialyzed and cleaved samples onto the same resin and collecting the flow-through. Protein concentrations were quantified using a bicinchoninic acid assay kit (Servicebio, #G2026) according to the description provided by the manufacturer.

### GST or His pull-down assay

25 μg Recombinant GST-tagged TRIM21 and its mutant variants were incubated with BeyoGold™ GST-tag Purification Resin (Beyotime, #P2253) in protein binding buffer (Tris-HCl 50 mM, NaCl 200 mM, EDTA 1 mM, NP-40 1% (v/v), DTT 1 mM, MgCl_2_ 10 mM, pH 8.0), and then, co-incubated with 25 μg recombinant PARP1 protein a total volume of 300 μL. The protein mixture was incubated for 2 h at 4 °C on rolls to allow for protein-protein interaction. The samples were washed again with elution buffer (Tris-HCl 50 mM; NaCl 400 mM; GSH 50 mM; EDTA 1 mM; DTT 1 mM; pH 8.0), boiled in 2x loading buffer, resolved on standard SDS-PAGE, and subsequently subjected to Western blotting. The method of His pull-down assay was similar to the process of the GST pull-down assay.

### Ubiquitylation assays

The ubiquitination assay was performed under denaturing conditions^82^. Briefly, HEK293T or SCLC cells transfected with the indicated plasmids or siRNAs were washed twice with cold PBS and lysed on ice with lysis buffer (20 mM Tris-HCl, pH 7.5, 150 mM NaCl, 1% Triton X-100, 1 mM EDTA) supplemented with protease and phosphatase inhibitors (Roche) for 30 min. The lysates were sonicated twice at 30% power for 5 s each, then centrifuged at 13,000 × *g* for 15 min. After collecting the supernatants, SDS was added to a final concentration of 1%, and samples were boiled at 95 °C for 5 min to denature. The samples were then diluted 1:10 in 1% Triton X-100 (final SDS concentration 0.1%). For the detection of endogenous ubiquitination, lysates were incubated with an anti-PARP1 antibody for 16 h at 4 °C. Protein A/G magnetic beads (Thermo Fisher Scientific, #80105 G) were then added and incubated for an additional 4 h at 4 °C. The beads were washed three times with dilution buffer and then directly boiled in 1× SDS loading buffer and analyzed by immunoblotting. For the detection of exogenous ubiquitination, lysates were incubated with BeyoMag™ Anti-Flag Magnetic Beads (Beyotime, #P2115) for 16 h at 4 °C. The beads were collected using a magnetic stand, washed three times with dilution buffer, and eluted with 100 μL of 3× Flag peptide elution solution (150 μg/mL) for 1 h at 4 °C with gentle shaking. The mixture was centrifuged at 6000 × *g* for 30 s at 4 °C, and the supernatant was collected. PARP1 ubiquitination was analyzed by immunoblotting using anti-HA or anti-Ub antibodies. To avoid interference from antibody heavy chains, a light chain-specific secondary antibody, IPKine™ HRP IgG LCS (Abbkine), was used for Western blot detection.

### In vitro ubiquitination assays

The first part is the in vitro ubiquitination of the Flag-PARP1 complex. Flag-*PARP1* was expressed in HEK293T cells. After the Flag pull-down, the Flag-PARP1 complex was eluted with elution buffer supplemented with Flag peptide. For the in vitro ubiquitination assay, we used a Ubiquitinylation kit (Enzo, #BML-UW9920) with the following specific protocol: 5 μg Flag-PARP1 complex was incubated in a 50 μl reaction mixture containing 5 μl 10× ubiquitinylation buffer, 10 μl IPP (100 U/ml), 1 μl DTT (50 mM), 2.5 μl Mg-ATP, 2.5 μl 20× ubiquitin, 2.5 μl 20× E1, 5 μl 10× E2. After incubation for 3 h at 37 °C, the reaction was stopped by adding 5× loading buffer, boiled for 10 min, and subjected to immunoblotting to measure the levels of PARP1 ubiquitination. In the second part, the in vitro ubiquitination of PARP1 by TRIM21 was performed, and the specific method was the same as the ubiquitination of the above Flag-PARP1 complex, where purified recombinant TRIM21 and PARP1 usage was 5 μg each.

### Comet assays

Alkaline comet assays were performed using the Comet Assay Kit (R&D Systems, #4250-050) according to the manufacturer’s protocol. Briefly, the lysis solution was prepared and chilled at 4 °C for at least 20 min before use. Agarose was melted in a boiling water bath for 5 min and then cooled in a 37 °C water bath for at least 20 min. Cells (1 × 10^5^ /mL) were mixed with molten agarose at a 1:10 (v/v) ratio, and 50 μL of the mixture was placed onto comet slides. The slides were refrigerated at 4 °C for 10 min and then immersed in a 4 °C lysis solution for 30–60 min. Next, the slides were immersed in an alkaline unwinding solution (NaOH, 0.4 g; 200 mM EDTA, pH 10, 250 μL; deionized water 49.75 mL) for 20 min at RT, and then subjected to electrophoresis in alkaline electrophoresis solution (NaOH, 8 g; 500 mM EDTA, 2 mL; deionized water 1 L) at 21 volts for 30 min. Afterward, the slides were gently immersed twice in deionized water for 5 min each, followed by 70% ethanol for 5 min. The samples were then dried at 37 °C for 10 min, stained with SYBR Green for 10 min, and imaged using epifluorescence microscopy. Tail moments were analyzed with the Comet Assay Software Project (CASP).

### Dual-Luciferase reporter assay

The luciferase reporter assay was performed as previously described^83^. Briefly, the *TRIM21* promoter sequence was cloned into the PGL4 vector (referred to as PGL4-*TRIM21*). HEK293T cells were co-transfected with PGL4-*TRIM21*, Flag-*STAT5A*, and a Renilla luciferase plasmid as an internal control. Cells were harvested and lysed 48 h post-transfection, and luciferase activity was measured using a dual-luciferase assay kit (Vazyme, #DL101). Firefly luciferase signals were normalized to the corresponding Renilla luciferase values for each sample.

### Drug synergy assay

Drug synergy was assessed using Bliss independence modeling implemented in the Combenefit software (CRUK Cambridge Institute)^82,84^. Briefly, cells representing different SCLC subtypes, including subtype *A* (SHP77), subtype *N* (DMS273), subtype *P* (H526), and subtype *Y* (DMS114), were treated with gradient concentrations of BMN673 and PKI-587, either as single agents or in combination, for 48 h. Cell viability was then measured using the CellTiter-Glo luminescent assay. Sigmoidal dose-response curves were generated, and synergy scores were calculated via Bliss synergy analysis within the Combenefit software. A similar analytical approach was applied to evaluate drug synergy in four SCLC organoid models: subtype *A* (SC049), subtype *A* + *N* (SC081), subtype *P* (SC080), and subtype *Y* (SC030).

### In vivo tumor growth and therapeutic study

Female nu/nu athymic mice (BALB/c), aged 4–6 weeks, were purchased from the Guangdong Medical Laboratory Animal Center (Guangzhou, China). The mice were randomly assigned to experimental groups, and xenograft experiments were initiated 7–10 days after their arrival. All mice were maintained in an enriched environment under controlled standard conditions: a temperature of 23–25 °C, humidity of 40–60%, and a 12-h light/dark cycle (lights on from 08:00 to 20:00). All animal experiments were conducted in accordance with a protocol approved by the Institutional Animal Care and Use Committee of Longgang District People’s Hospital of Shenzhen (2024003DW). Athymic nude mice were subcutaneously injected in the dorsal flank with a 100 μL suspension containing 2 × 10⁶ cells mixed in an equal volume of Matrigel (Corning, #354230). In the first part of the study, stable DMS273 cell lines with *TRIM21* knockdown (sh*TRIM21* #1) or overexpression (*TRIM21* OV), along with their respective controls (shControl/Scr and Empty Vector/EV), were subcutaneously implanted into nude mice, forming four experimental groups. In the second part of the experiment, intratumoral injection of siRNA was performed^85,86^. Briefly, stable DMS273 cell lines with *TRIM21* knockdown (sh*TRIM21* #1) or *PARP1* knockdown (sh*PARP1* #1) were similarly implanted. Once tumors reached approximately 150 mm³, the sh*TRIM21* #1 group was randomly divided into two subgroups: one continued without further treatment, while the other received intratumoral injections of si*PARP1* #1 to establish the “sh*TRIM21* #1 + si*PARP1* #1” cohort. The intratumoral injection of siRNAs was performed using in vivo-jetPEI® (Polyplus, #101000030) according to the manufacturer’s guidelines. 10 μg si*PARP1* #1 diluted in sterile 5% glucose solution was complexed with in vivo-jetPEI® at a ratio of 0.16 μL reagent per 1 μg siRNA. The formulated siRNA was administered intratumorally every two days. siRNA sequences are detailed in Supplementary Data 3. In the third part of the experiment, when the tumors became palpable (approximately 150 mm³), tumor-bearing mice were randomly divided into four groups for treatment. The dosing regimen for PKI-587 was 7.5 mg/kg, administered via tail vein injection every two days. BMN673 was administered at a dose of 0.33 mg/kg via intraperitoneal injection every two days. Mice in the combination treatment group received both PKI-587 and BMN673, following the same dosing regimen and administration routes as in the single-drug groups. The control group received an equivalent volume of PBS. All tumor sizes and mouse body weights were measured beginning when the tumor volume reached ~150 mm³, with measurements taken every two days. Tumor volume was calculated using the formula: tumor volume (mm³) = (tumor length × tumor width²) / 2. Tumor volumes were not allowed to exceed 1500 mm³, as authorized by the Committees on Animal Research and Ethics, and this limit was not exceeded during the experiments. At the end of the experiment, mice were euthanized at the appropriate time points, and tumors were excised, photographed, weighed, and embedded in paraffin for further pathological analysis.

### Immunohistochemical staining (IHC)

For IHC, tissue specimens were fixed in 10% formalin and subsequently embedded in paraffin. Sections with a thickness of 4 μm were prepared and deparaffinized in xylene. IHC staining was conducted using the EnVision Detection Systems HRP, Rabbit/Mouse (DAB + ) kit (Agilent), in adherence to the provided manufacturer’s guidelines. Endogenous peroxidase activity was mitigated by treating the sections with 0.3% hydrogen peroxide for 15 min. Antigen retrieval was achieved by heating the slides in 10 mM citrate buffer (pH 6.0) in a water bath for 20 min. The sections were then washed with PBS containing 0.05% Tween and blocked for 30 min using 5% bovine serum albumin (BSA). They were incubated overnight at 4 °C with the following primary antibodies (Supplementary Data 2): anti-TRIM21 (1:300), anti-PARP1 (1:300), p-AKT (1:300), p-STAT5A (1:300), γH2AX (1:300), and anti-Ki-67 (1:500). This was succeeded by 1 h RT incubation with a Labelled Polymer-HRP. For negative controls, sections were processed analogously, albeit without the inclusion of primary antibodies. The sections were subsequently treated with DAB^+^ Chromogen, and counterstaining was performed with hematoxylin. Following mounting, the slides were examined under a microscope. After DAB^+^ chromogen development, the sections were counterstained with hematoxylin, mounted, and examined under a microscope. The expression level of each IHC marker is expressed as the Integrated Optical Density (IOD) divided by the area of the region of interest (IOD/Area)^87,88^. Here, IOD represents the cumulative optical density within positively stained regions, and area refers to the entire defined region of interest; both parameters were measured using Image-Pro Plus software.

### SCLC PDX models and organoid-based therapy

The 13 SCLC PDX models used in this study were derived from previously published work^89^, with detailed establishment methods described in references^89,90^. IHC was performed to evaluate the expression levels of p-AKT, p-STAT5A, PARP1, TRIM21, and γH2AX, followed by correlation analysis.

For the establishment of SCLC organoids, 4 SCLC PDX models were first generated as described previously^89,90^. Briefly, transbronchoscopic lung biopsies collected from SCLC patients were embedded in Matrigel (Corning, #354230) and subsequently injected into the right flank of male C.B-17 severe combined immunodeficiency (SCID) mice, aged 4–6 weeks, which were obtained from Shanghai SLAC Laboratory Animal Co., Ltd. (Shanghai, China). Written informed consent was obtained from all patients, and the study was approved by the Medical Ethics Committee of Shanghai Pulmonary Hospital, Tongji University Medical School Cancer Institute (ethical approval Nos. K19-159 and K21-019). PDX tissues were then harvested. Subsequently, SCLC organoids were obtained as described in ref.^91^. In brief, SCLC PDX tumors were dissected and enzymatically digested in a buffer consisting of Advanced DMEM/F12 supplemented with 5 mg/mL Collagenase Type II, 1× penicillin/streptomycin, 1 mg/mL primocin, and 10 mM Y-27632 for 45 min at 37 °C. The resulting cell suspension was collected, resuspended in organoid culture medium, mixed 1:1 with Basement Membrane Extract (Corning, #356231), and seeded into 24-well plates at a density of 5000–10,000 cells per well. For organoid-based drug evaluation, four representative models corresponding to distinct molecular subtypes were selected: SC049 (subtype *A*), SC081 (subtype *A* + *N*), SC080 (subtype *P*), and SC030 (subtype *Y*). The therapeutic effects of BMN673 and PKI-587, both as single agents and in combination, were subsequently examined in these organoid systems.

### Statistical analysis

All cellular and biochemical experiments were performed with at least three independent repeats. The symbol “*n*” denotes the number of experimental replicates, quantified cells, animals, or PDX specimens used in each analysis. Image processing and quantification were carried out using ImageJ or Image-Pro Plus software, as specified in the respective methods. Statistical analyses were conducted with GraphPad Prism 8. Comparisons between two independent groups were made using an unpaired, two-tailed Student’s *t*-test, while paired samples were analyzed with a paired Student’s *t*-test. One-way ANOVA with Tukey’s test was used to compare multiple groups of data. Pearson’s correlation coefficient was calculated to assess the relationship between variables. Overall survival was depicted using Kaplan-Meier survival curves, with significance determined by the log-rank test. The differences were considered statistically significant when *P* values were below 0.05.

### Reporting summary

Further information on research design is available in the Nature Portfolio Reporting Summary linked to this article.

## Supplementary information

Supplementary Information

Description of Additional Supplementary Files

Supplementary Data 1

Supplementary Data 2

Supplementary Data 3

Reporting Summary

Transparent Peer Review file

## Source data

Source Data

## Data Availability

All the mass spectrometry proteomics data have been deposited to the ProteomeXchange Consortium (http://proteomecentral.proteomexchange.org) via the iProX partner repository with the dataset identifier PXD068168. The mass spectrometry analysis was performed once (*n* = 1, no biological replicates), and the potential PARP1-associated proteins are listed in Supplementary Data 1. The RNA-seq data for 1,019 cancer cell lines and the proteomic profiles of 375 cancer cell lines were retrieved from the Cancer Cell Line Encyclopedia (CCLE) via https://portals.broadinstitute.org/ccle/data. The RNA-seq datasets were downloaded from the NCBI Gene Expression Omnibus under accessions GSE40564, GSE60052, GSE30219, GDS4794, GSE149507, and GSE40564; obtained from the Genome Sequence Archive (http://bigd.big.ac.cn/gsa-human) under accession HRA003419; and obtained from the European Genome-phenome Archive under accession EGAS00001000925. The proteomic data were secured from the OMIX database under accession OMIX002489. All ChIP-seq data were sourced from the ChIP-Atlas database under accessions SRX831872, SRX041292, SRX18444036, SRX9681372, SRX9932045, and SRX190177. The information on compounds and antibodies is provided in Supplementary Data 2. All the sequences of oligos used in this study are presented in Supplementary Data 3. The original photographs of immunoblots are provided in the source data file. The remaining data are available within the Article, Supplementary Information, or Source Data file. Source data are provided with this paper.
