# Supplementary material for: TRIM21-mediated ubiquitination of PARP1 regulated by the PI3K/AKT-STAT5A axis suppresses small cell lung cancer
Source: Nat Commun. 2026 Jun 18;17:7704. doi: 10.1038/s41467-026-73271-1 (PMC13434705; doi:10.1038/s41467-026-73271-1)

Supplementary Information for

TRIM21-mediated ubiquitination of PARP1 regulated by the PI3K/AKT-STAT5A axis suppresses small cell lung cancer

Guozhen Cao[1, 2, 3, #], Gongfeng Li[1, 2, 3, #], Xiaolin Wang[2], Tengfei Zhang[4, 5], Xinhuang Yao[1], Peng Hou[1, 2, 3], Jiahui Zhang[1, 2, 3], Xiaopeng Guo[6], Jiarong Wang[3], Li Xiang[1], Hongbin Ji[4, 7], Chenchen Guo[4, 8, 9, *], Wenchu Lin[1, *]

[1] The Second Affiliated Hospital, School of Medicine, The Chinese University of Hong Kong, Shenzhen & Longgang District People's Hospital of Shenzhen, Shenzhen 518172, China

[2] University of Science and Technology of China, Hefei 230036, Anhui, China

[3] High Magnetic Field Laboratory, Chinese Academy of Sciences, Hefei 230031, Anhui, China

[4] Key Laboratory of Multi-Cell Systems, Shanghai Institute of Biochemistry and Cell Biology, Center for Excellence in Molecular Cell Science, Chinese Academy of Sciences, Shanghai 200031, China

[5] University of Chinese Academy of Sciences, Beijing 100049, China

[6] School of Life Science and Engineering, Lanzhou University of Technology, Lanzhou 730050, Gansu, China

[7] Westlake University School of Medicine, Hangzhou 310030, Zhejiang, China

[8] Shanghai Institute of Thoracic Oncology, Shanghai Chest Hospital, Shanghai Jiao Tong University School of Medicine, Shanghai 200030, China

[9] Shanghai Key Laboratory of Thoracic Tumor Biotherapy, Shanghai Chest Hospital, Shanghai Jiao Tong University School of Medicine, Shanghai 200030, China

Running Title: PI3K/AKT-STAT5A-TRIM21-PARP1 axis Drives SCLC

[#]These authors contributed equally: Guozhen Cao, Gongfeng Li.

*These authors jointly supervised this work. Correspondence to Chenchen Guo, Wenchu Lin.

Chenchen Guo, Ph. D., Shanghai Institute of Thoracic Oncology, Shanghai Chest Hospital, Shanghai Jiao Tong University School of Medicine, Shanghai 200030, China. E-mail: guochenchen2014@sibcb.ac.cn;

Wenchu Lin, Ph. D., The Second Affiliated Hospital, School of Medicine, The Chinese University of Hong Kong, Shenzhen & Longgang District People's Hospital of Shenzhen, Shenzhen 518172. China. E-mail: linwenchu@ustc.edu.cn.

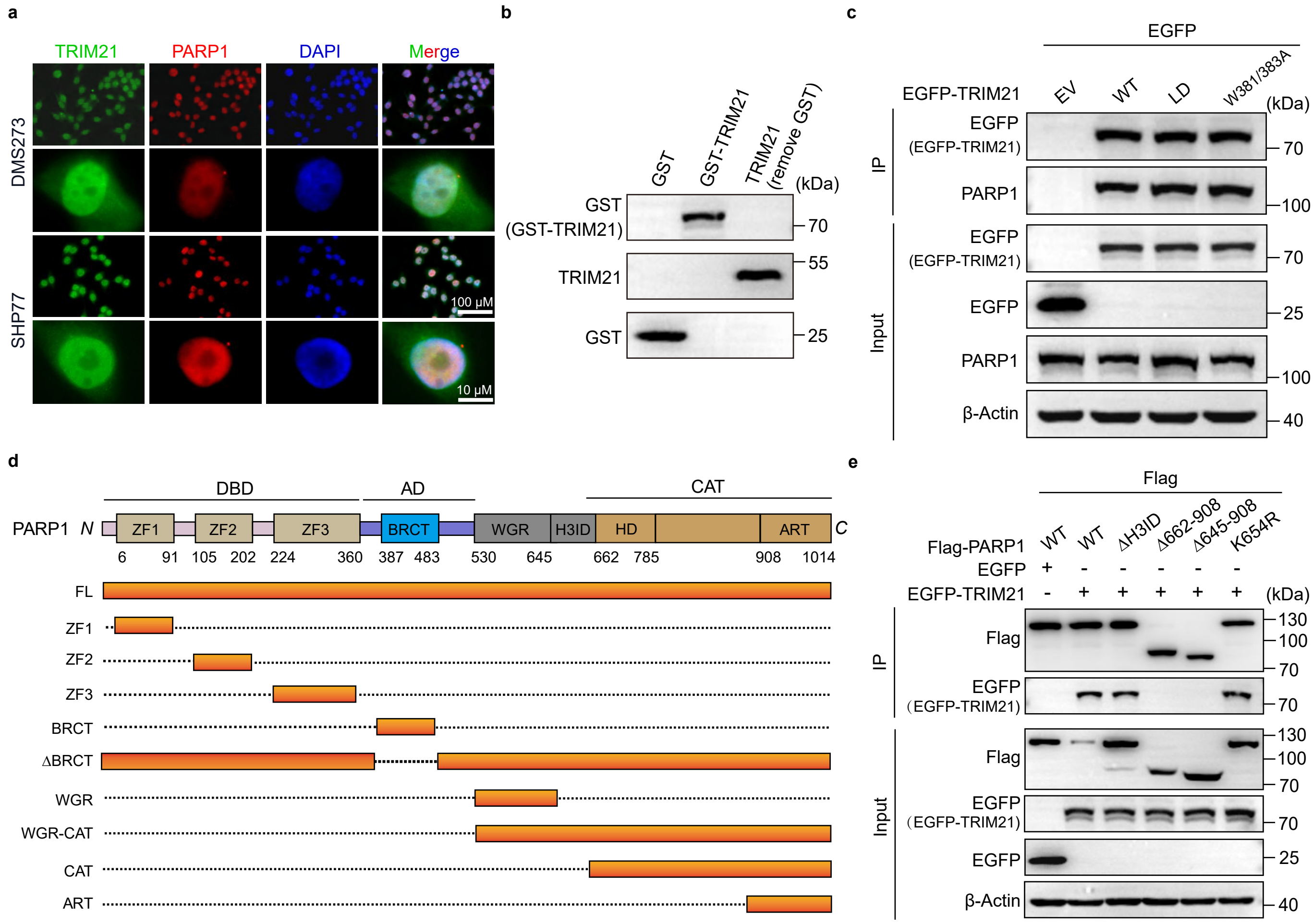

Supplementary Fig. 1 PARP1 binds with TRIM21. a Immunofluorescence assay demonstrating the cellular localization of TRIM21 and PARP1 in DMS273 and SHP77 cells. **b** Western blot analysis of TRIM21 after removal of the GST tag from GST-TRIM21 recombinant protein. **c** Immunoprecipitation (IP)-Western analysis of the interaction between PARP1 and TRIM21. HEK293T cells were transfected with either EGFP-empty, EGFP-*TRIM21* WT, EGFP-*TRIM21* LD (C16A, C31A, and H33W), or EGFP-*TRIM21* W381/383A mutant. The interaction between TRIM21 and PARP1 was detected through IP followed by Western blot analysis. **d** Schematic diagram illustrating the domains of the PARP1 protein alongside deletion constructs. **e** IP-Western analysis demonstrating the domain of PARP1 required for its interaction with TRIM21. Flag-*PARP1* WT, Flag-*PARP1* Δ HI3D, Flag-*PARP1* Δ 662-908, Flag-*PARP1* Δ 645-908, or Flag-*PARP1* K654R was co-transfected with EGFP-*TRIM21* or EGFP-empty in HEK293T cells, and then the interaction between TRIM21 and PARP1 was evaluated by IP and immunoblotting. Source data are provided as a Source Data file.

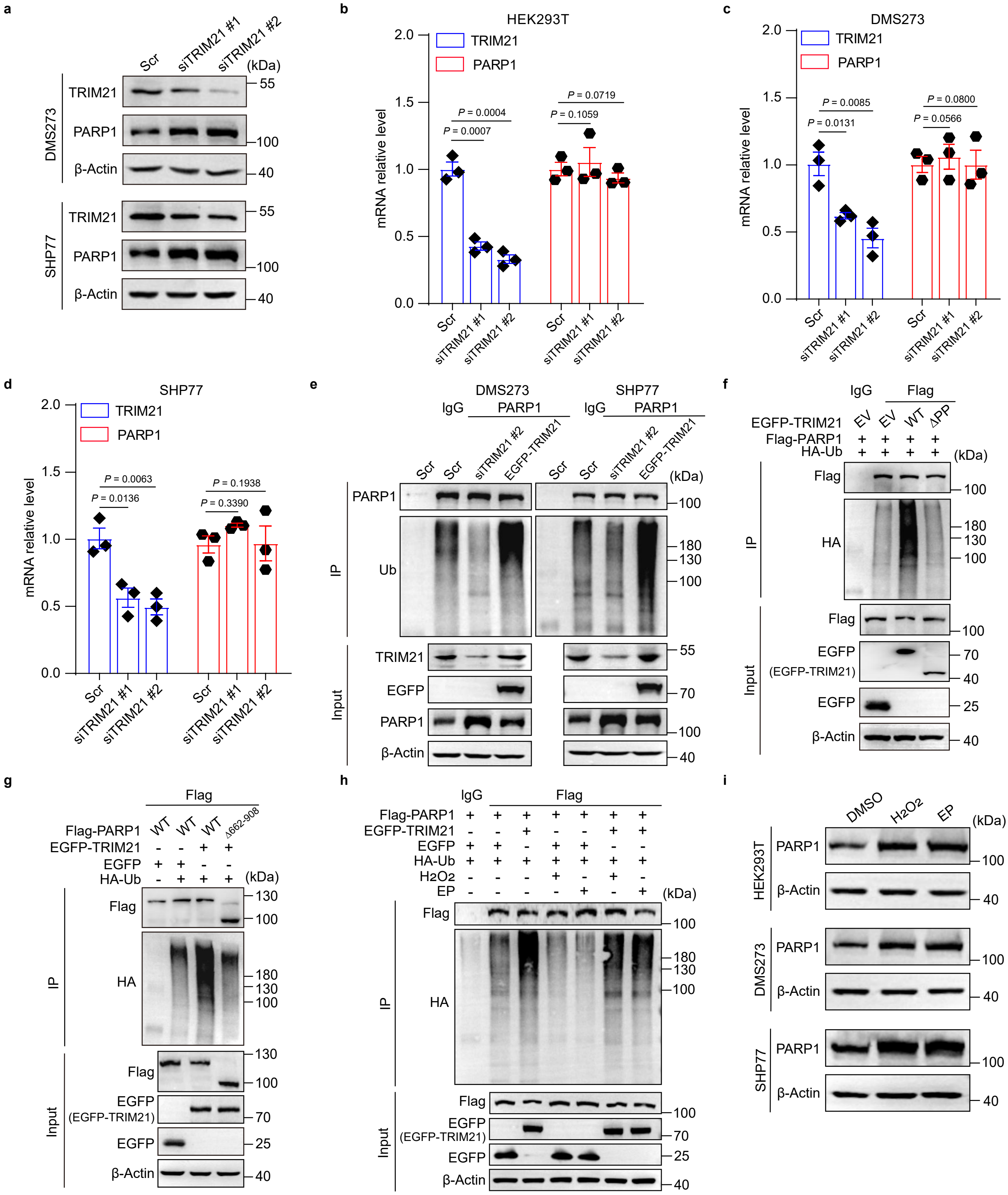

a
Scr
siTRIM21 #1
siTRIM21 #2
(kDa)
DMS273
TRIM21
PARP1
β-Actin
55
100
40
SHP77
TRIM21
PARP1
β-Actin
55
100
40
b
HEK293T
TRIM21
PARP1
mRNA relative level
P = 0.0004
P = 0.0007
P = 0.0719
P = 0.1059
Scr
siTRIM21 #1
siTRIM21 #2
c
DMS273
TRIM21
PARP1
mRNA relative level
P = 0.0085
P = 0.0131
P = 0.0800
P = 0.0566
Scr
siTRIM21 #1
siTRIM21 #2
d
SHP77
TRIM21
PARP1
mRNA relative level
P = 0.0063
P = 0.0136
P = 0.1938
P = 0.3390
Scr
siTRIM21 #1
siTRIM21 #2
e
DMS273
SHP77
IgG
PARP1
Scr
siTRIM21 #2
EGFP-TRIM21
(kDa)
IP
PARP1
Ub
Input
TRIM21
EGFP
PARP1
β-Actin
100
180
130
55
70
40
f
IgG
Flag
EGFP-TRIM21
EV
WT
ΔPP
Flag-PARP1
HA-Ub
(kDa)
IP
Flag
HA
Input
Flag
EGFP
(EGFP-TRIM21)
EGFP
β-Actin
100
180
130
70
40
25
g
Flag
Flag-PARP1
WT
Δ662-908
EGFP-TRIM21
EGFP
HA-Ub
(kDa)
IP
Flag
HA
Input
Flag
EGFP
(EGFP-TRIM21)
EGFP
β-Actin
130
100
180
70
25
40
h
IgG
Flag
Flag-PARP1
EGFP-TRIM21
EGFP
HA-Ub
H2O2
EP
(kDa)
IP
Flag
HA
Input
Flag
EGFP
(EGFP-TRIM21)
EGFP
β-Actin
100
180
130
70
25
40
i
DMSO
H2O2
EP
(kDa)
HEK293T
PARP1
β-Actin
DMS273
PARP1
β-Actin
SHP77
PARP1
β-Actin
100
40

Supplementary Fig. 2 TRIM21 facilitates the ubiquitination and degradation of PARP1. **a** Western blot analysis of PARP1 following *TRIM21* knockdown using siRNAs targeting *TRIM21*. **b**–**d** RT-qPCR analysis of *PARP1* expression in response to *TRIM21* knockdown by siRNAs in HEK293T (**b**), DMS273 (**c**), and SHP77 (**d**) cells, normalized to β-Actin. **e** *In vivo* ubiquitination of PARP1. Immunoprecipitation (IP) followed by Western blot was employed to detect total ubiquitinated PARP1 in both *TRIM21* knockdown and overexpressed DMS273 and SHP77 cells. **f**, **g** IP-Western analysis showing the requirement of the TRIM21-PARP1 interaction for TRIM21-mediated ubiquitination and degradation of PARP1. HEK293T cells were co-transfected with Flag-*PARP1*, HA-Ub, and either EGFP-*TRIM21* or EGFP-*TRIM21* ∆PP (**f**), or co-transfected with HA-Ub, EGFP-*TRIM21*, and either Flag-*PARP1* or Flag-*PARP1* ∆662-908 (**g**). PARP1 ubiquitination levels were assessed via IP-Western analysis. **h** Assessment of PARP1 ubiquitination levels under DNA damage-inducing conditions. HEK293T cells were co-transfected with Flag-*PARP1*, HA-Ub, and either EGFP-*TRIM21* or EGFP-empty vector. After treatment with etoposide (EP, 40 μM, 24 hours) or H_2O_2 (2 μM, 5 min), the ubiquitination and protein levels of PARP1 were evaluated by IP followed by Western blotting. **i** Analysis of PARP1 protein accumulation under DNA damage-inducing conditions. HEK293T, DMS273, and SHP77 cells were treated with EP (40 μM, 24 hours) or H_2O_2 (2 μM, 5 min), and PARP1 accumulation was analyzed by Western blotting. Data from three independent experiments are shown as mean ± SEM in (**b**–**d**). Statistical analysis was performed using two-tailed unpaired Student's *t*-tests. Source data are provided as a Source Data file.

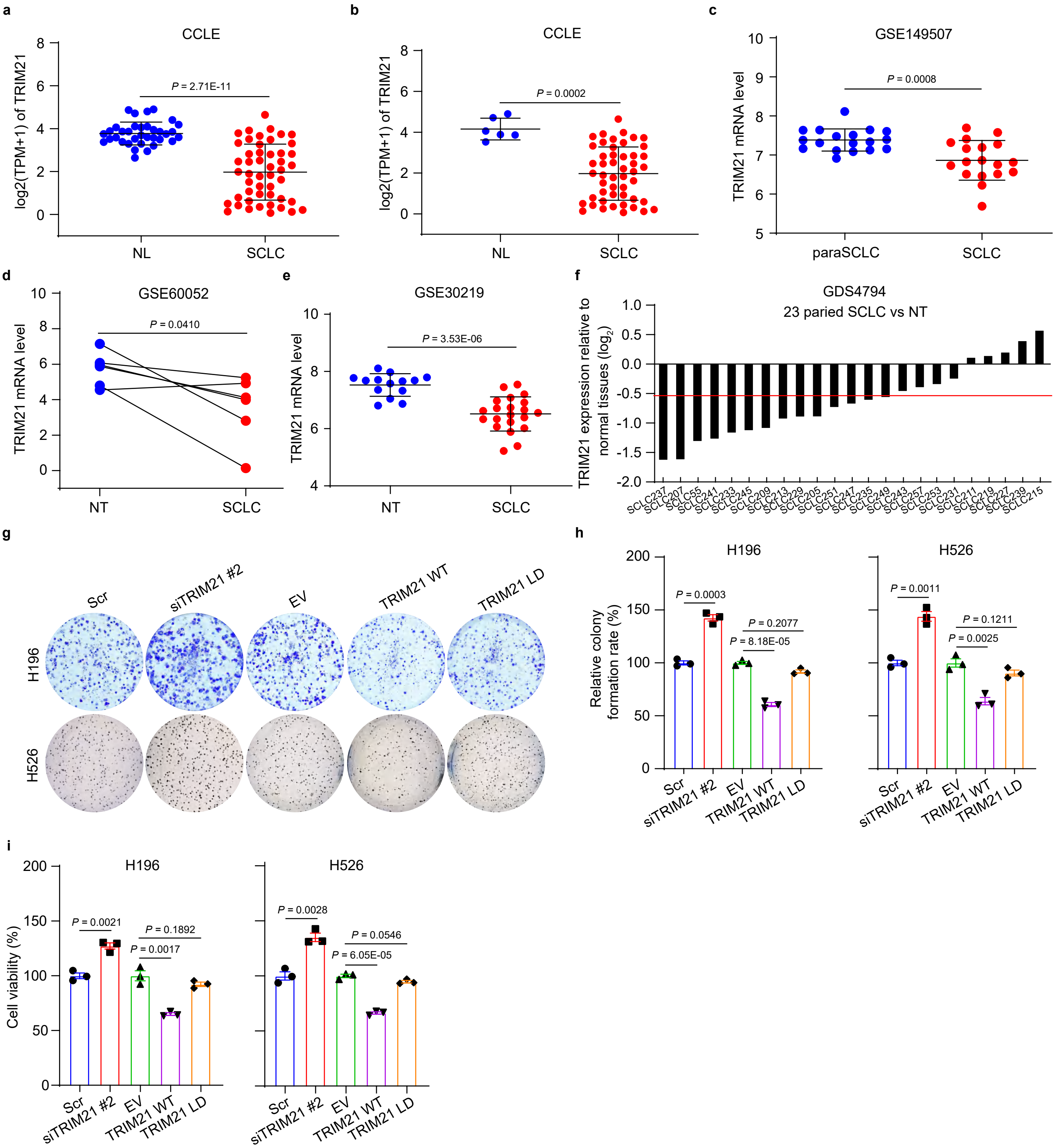
a
CCLE
log2(TPM+1) of TRIM21
P = 2.71E-11
NL
SCLC
b
CCLE
log2(TPM+1) of TRIM21
P = 0.0002
NL
SCLC
c
GSE149507
TRIM21 mRNA level
P = 0.0008
paraSCLC
SCLC
d
GSE60052
TRIM21 mRNA level
P = 0.0410
NT
SCLC
e
GSE30219
TRIM21 mRNA level
P = 3.53E-06
NT
SCLC
f
GDS4794
23 paried SCLC vs NT
TRIM21 expression relative to normal tissues (log2)
g
Scr
siTRIM21 #2
EV
TRIM21 WT
TRIM21 LD
H196
H526
h
H196
H526
Relative colony formation rate (%)
P = 0.0003
P = 0.2077
P = 8.18E-05
P = 0.0011
P = 0.1211
P = 0.0025
Scr
siTRIM21 #2
EV
TRIM21 WT
TRIM21 LD
i
H196
H526
Cell viability (%)
P = 0.0021
P = 0.1892
P = 0.0017
P = 0.0028
P = 0.0546
P = 6.05E-05
Scr
siTRIM21 #2
EV
TRIM21 WT
TRIM21 LD

Supplementary Fig. 3 TRIM21 is downregulated in SCLC. **a**, **b** Scatter plot analysis of the CCLE dataset showing significantly reduced expression of *TRIM21* in SCLC cells (*n* = 49) compared to normal tissue cell lines (NL) (*n* = 35) (**a**), and normal lung cell lines (NL) (*n* = 6) (**b**). **c** Analysis of *TRIM21* mRNA expression in SCLC and adjacent normal tissues (paraSCLC) from the GSE149507 dataset (*n* = 18). **d** Scatter plot comparing *TRIM21* expression in SCLC tissues versus paired normal lung tissues (NT) from the GSE60052 dataset (*n* = 6). **e** Analysis of *TRIM21* mRNA expression levels in 21 SCLC tissues and 14 normal tissues (NT) from the GSE30219 dataset. **f** Relative *TRIM21* expression in human SCLC tissues compared to the normal lung tissues (NT) from the GDS4794 dataset (*n* = 23, the red line means more than a 1.5-fold increase). **g** Colony formation assays in H526 and H196 cells following either *TRIM21* knockdown (si*TRIM21*) or overexpression of wild-type (WT) or ligase-deficient mutant (LD, C16A, C31A, and H33W) *TRIM21*. For the suspension cell line H526 in soft agar colony formation assays. **h** Quantification of clonogenic survival from (**g**) using ImageJ software. **i** Cell viability measured by CellTiter-Glo assay in H526/H196 cells with si*TRIM21*, *TRIM21* WT, or *TRIM21* LD expression. Data are shown as mean ± SD in (**a**–**c**, **e**). Data from three independent experiments are shown as mean ± SEM in (**h**, **i**). Statistical analysis was performed using two-tailed unpaired Student's *t*-tests in (**a**–**c**, **e**, **h**, **i**); data in (**d**) were analyzed by two-tailed paired Student's *t*-tests. Source data are provided as a Source Data file.

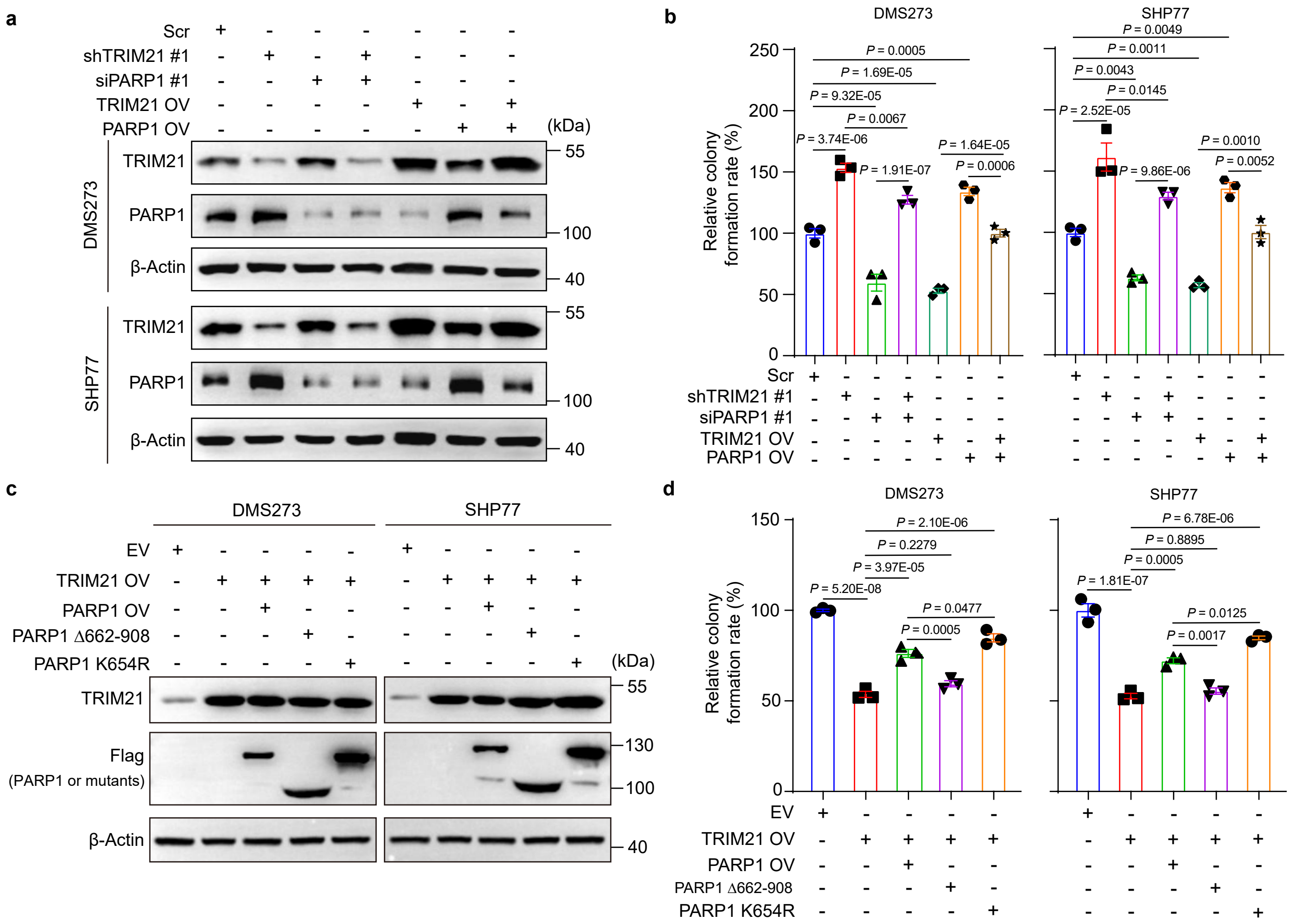

Supplementary Fig. 4 TRIM21 suppresses the survival of SCLC cells by decreasing PARP1 accumulation. **a** Western blot analysis investigating the compensatory effects of *PARP1* on altered *TRIM21* expression. PARP1 and TRIM21 protein levels were assessed following the treatment described in Fig. **4a**. **b** Quantitative analysis of colony formation shown in Fig. **4b**. **c** Western blot analysis of TRIM21, full-length PARP1, and its mutants in *TRIM21*-overexpressing cells. The protein levels of TRIM21, PARP1, and its mutants were analyzed following the treatment described in Fig. **4c**. **d** Quantitative analysis of colony formation is shown in Fig. **4d.** Data from three independent experiments are shown as mean ± SEM in (**b**, **d**). Statistical analysis was performed using two-tailed one-way ANOVA. Source data are provided as a Source Data file.

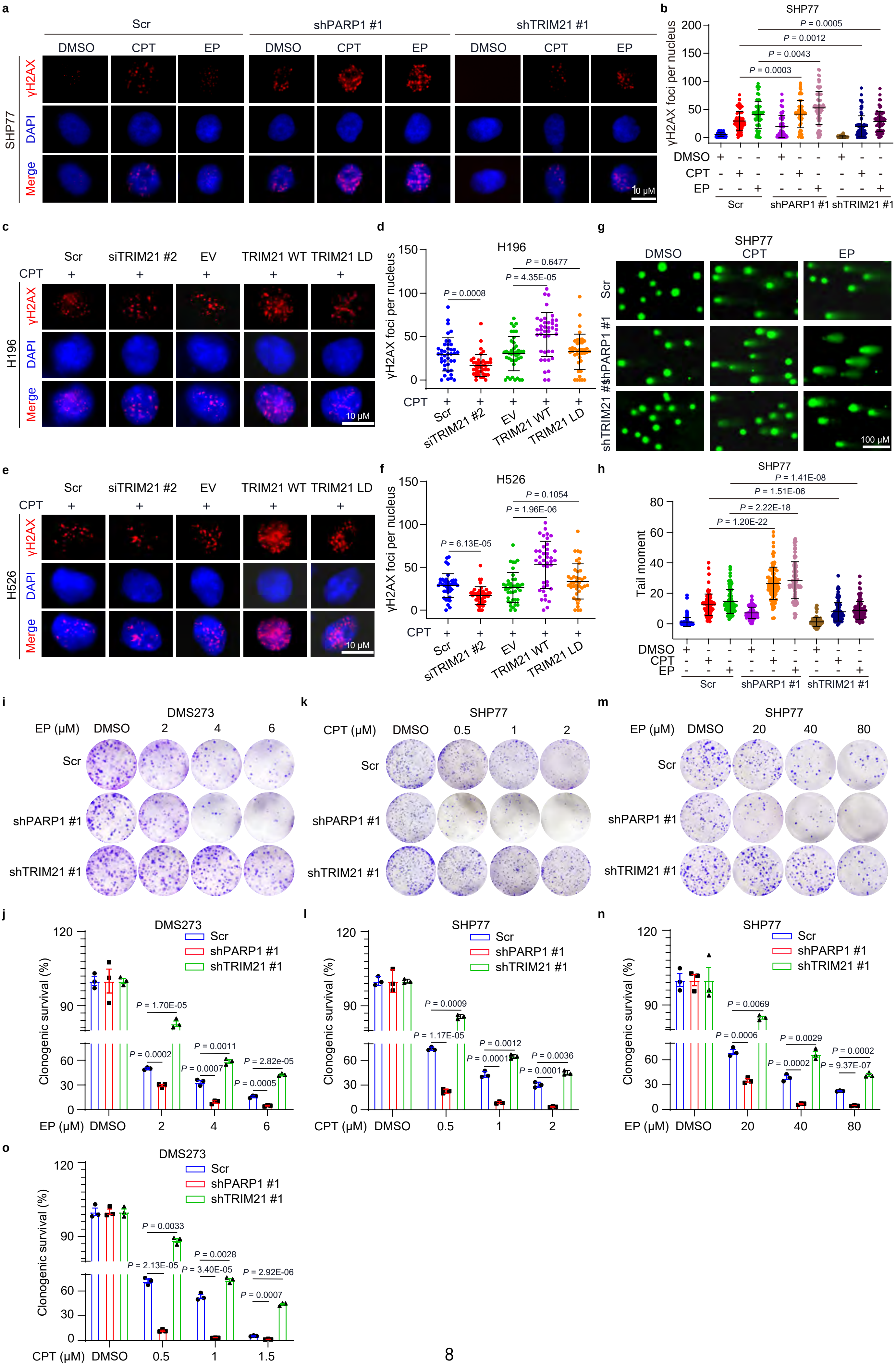
a
Scr
shPARP1 #1
shTRIM21 #1
DMSO
CPT
EP
SHP77
γH2AX
DAPI
Merge
10 μM
b
SHP77
γH2AX foci per nucleus
P = 0.0005
P = 0.0012
P = 0.0043
P = 0.0003
DMSO
CPT
EP
Scr
shPARP1 #1
shTRIM21 #1
c
Scr
siTRIM21 #2
EV
TRIM21 WT
TRIM21 LD
CPT
H196
10 μM
d
H196
P = 0.6477
P = 4.35E-05
P = 0.0008
e
H526
f
H526
P = 0.1054
P = 1.96E-06
P = 6.13E-05
g
SHP77
100 μM
h
SHP77
Tail moment
P = 1.41E-08
P = 1.51E-06
P = 2.22E-18
P = 1.20E-22
i
DMS273
EP (μM)
k
SHP77
CPT (μM)
m
SHP77
EP (μM)
j
DMS273
Clonogenic survival (%)
P = 1.70E-05
P = 0.0002
P = 0.0011
P = 0.0007
P = 2.82e-05
P = 0.0005
l
SHP77
P = 0.0009
P = 1.17E-05
P = 0.0012
P = 0.0001
P = 0.0036
P = 0.0001
n
SHP77
P = 0.0069
P = 0.0006
P = 0.0029
P = 0.0002
P = 0.0002
P = 9.37E-07
o
DMS273
P = 0.0033
P = 2.13E-05
P = 0.0028
P = 3.40E-05
P = 2.92E-06
P = 0.0007

Supplementary Fig. 5 TRIM21 deficiency enhances DNA damage responses by induction of PARP1 accumulation. a Immunostaining showing the formation of γH2AX foci in SHP77 cells following the protocol described in Fig. **5c**. Scale bars, 10 μm. **b** Quantification of the γH2AX foci formation in (**a**). **c**, **e** Immunofluorescence analysis of γH2AX foci in H196 (**c**) and H526 (**e**) cells treated with 2 μM Camptothecin (CPT, 1 hour) followed by 4 hours recovery under si*TRIM21*, overexpression of wild-type (WT) *TRIM21*, or ligase-deficient mutant (LD, C16A, C31A, and H33W) *TRIM21* conditions. Scale bars, 10 μm. **d**, **f** γH2AX foci quantification from (**c**, **e**). **g** Comet assays depicting the effect of *PARP1* or *TRIM21* knockdown on DNA damage in SHP77 cells. Comet assays were performed in SHP77 cells, following a protocol similar to that described in Fig. **5e**. Scale bars, 100 μm. **h** Quantification of the tail moment of the comet assay in (**g**). **i**, **k**, **m** Colony formation assays measuring the clonogenic capacity of the DMS273 or SHP77 cells treated by CPT or EP with indicated concentrations. **j**, **l**, **n** Quantitative analysis of colony formation shown in (**i**, **k**, **m**). **o** Quantitative analysis of colony formation shown in Fig. **5g**. Data in (**b**) (*n* = 80 cells), (**d**, **f**) (*n* = 40 cells), and (**h**) (*n* = 100 cells) are presented as mean ± SD. In (**j**, **l**, **n**, **o**), data from three independent experiments are shown as mean ± SEM. Statistical analysis was performed using two-tailed unpaired Student′s *t*-tests. Source data are provided as a Source Data file.

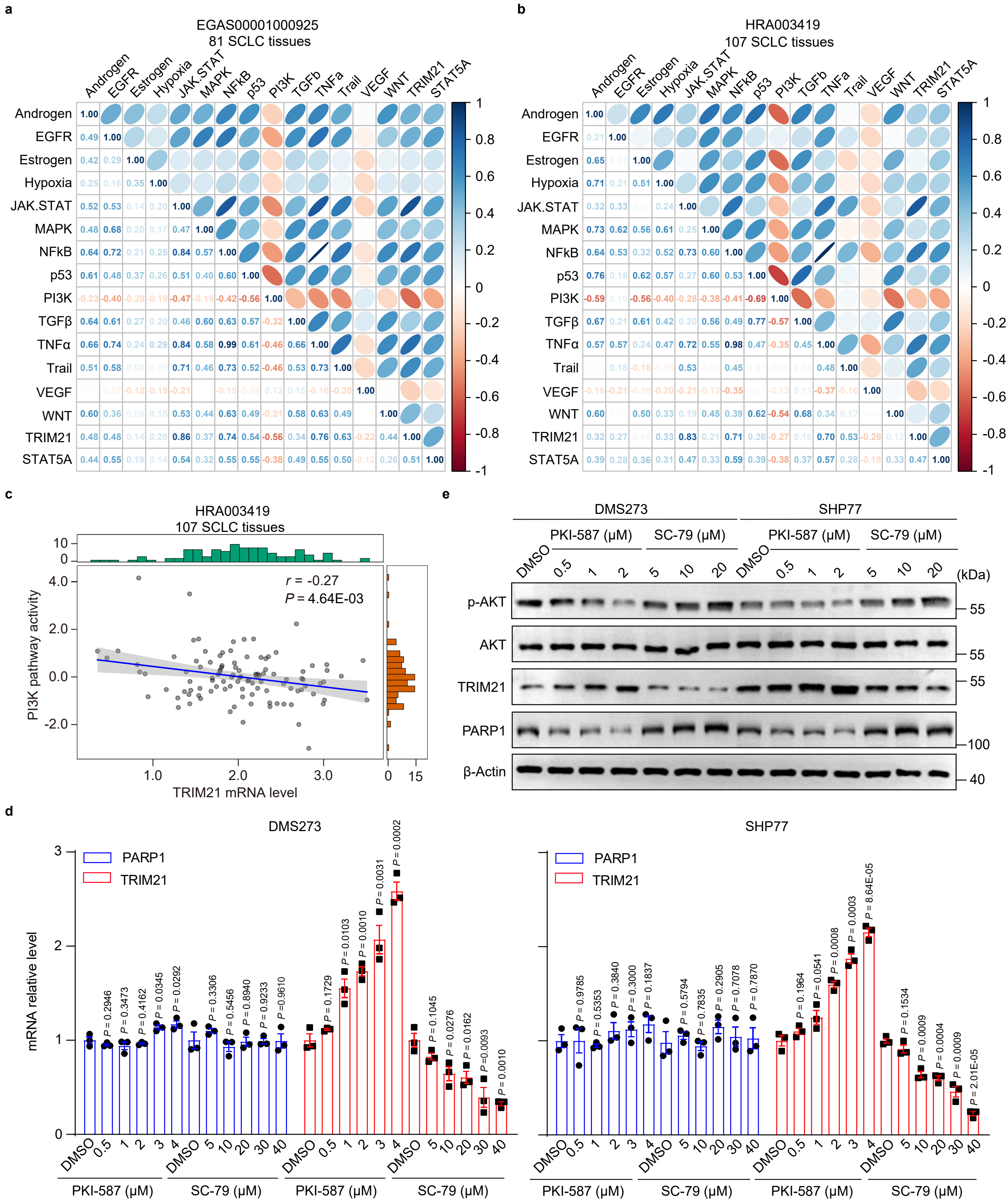
a
EGAS00001000925
81 SCLC tissues
Androgen
EGFR
Estrogen
Hypoxia
JAK.STAT
MAPK
NFkB
p53
PI3K
TGFb
TNFa
Trail
VEGF
WNT
TRIM21
STAT5A
TGFβ
TNFα
b
HRA003419
107 SCLC tissues
c
HRA003419
107 SCLC tissues
r = -0.27
P = 4.64E-03
PI3K pathway activity
TRIM21 mRNA level
e
DMS273
SHP77
DMSO
PKI-587 (μM)
SC-79 (μM)
(kDa)
p-AKT
AKT
TRIM21
PARP1
β-Actin
d
DMS273
SHP77
PARP1
TRIM21
mRNA relative level

Supplementary Fig. 6 PI3K/AKT facilitates PARP1 stabilization by blocking TRIM21 expression. a Correlation analysis of *TRIM21* and *STAT5A* mRNA levels with the activity of various signaling pathways in 81 SCLC tissues from the European Genome-phenome Archive database (EGAS00001000925). **b** A similar correlation analysis to (**a**) in 107 SCLC tissues from the Genome Sequence Archive (GSA) database (HRA003419). **c** Analysis of the correlation between PI3K/AKT pathway activity and *TRIM21* mRNA levels in SCLC tissues from the GSA database (HRA003419, n = 107; 95% confidence interval, -0.44 to -0.09). Two-tailed P value is reported. **d** RT-qPCR analysis demonstrating the effects of PKI-587 or SC-79 on the expression of *TRIM21* and *PARP1* in SCLC cells. DMS273 and SHP77 cells were treated with a gradient of different concentrations of PKI-587 or SC-79 for 24 hours, followed by measurement of *TRIM21* and *PARP1* mRNA levels. **e** Dose-response analysis of protein levels of AKT, p-AKT, TRIM21, and PARP1 in DMS273 and SHP77 cells treated with a gradient of PKI-587 or SC-79 concentrations for 24 hours, and these protein levels were subsequently assessed by Western blot. In (**d**), data from three independent experiments are shown as mean ± SEM. Statistical analysis was performed using two-tailed unpaired Student's *t*-tests. Source data are provided as a Source Data file.

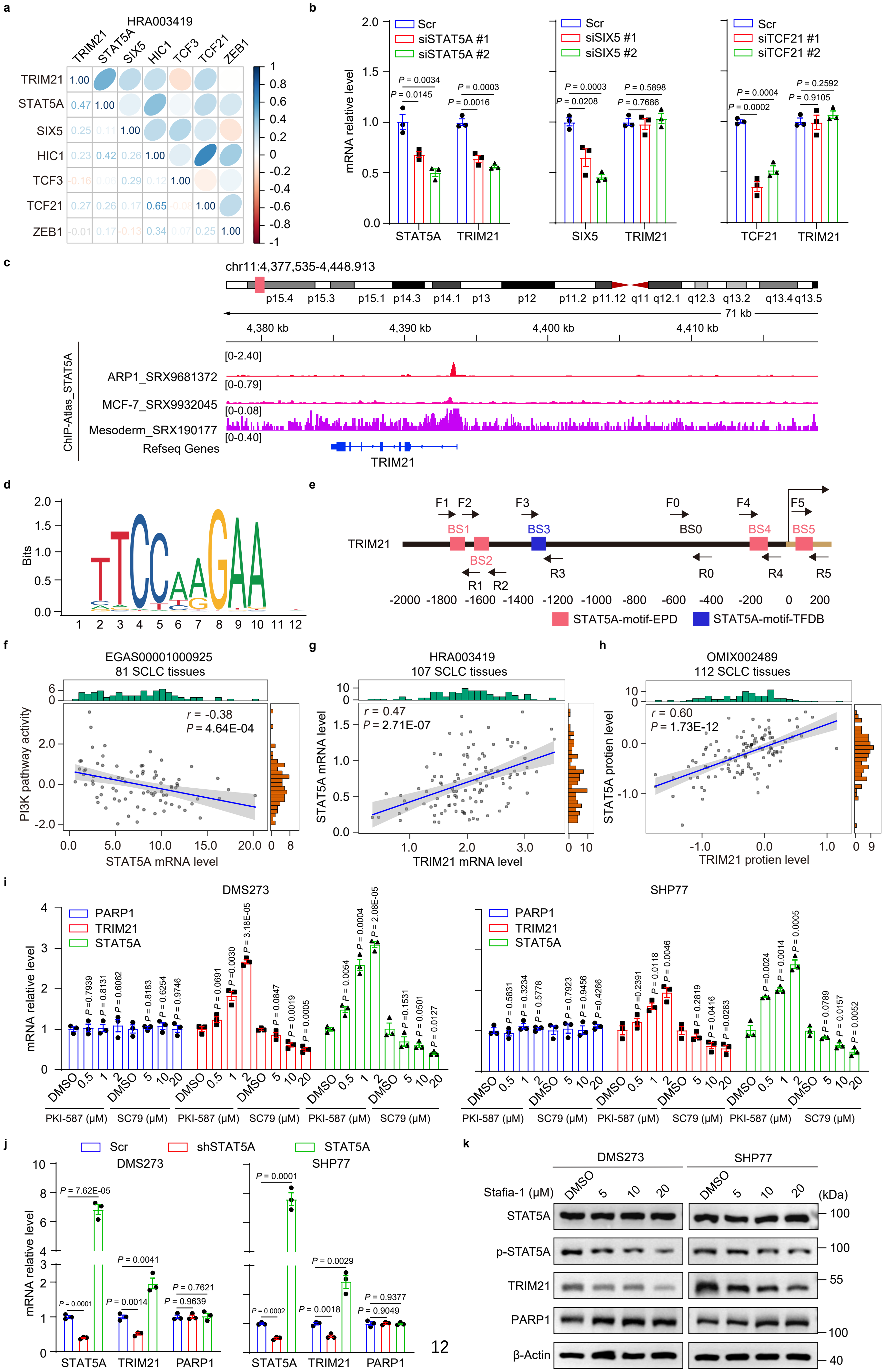
a
HRA003419
TRIM21
STAT5A
SIX5
HIC1
TCF3
TCF21
ZEB1
1.00
0.47 1.00
0.25 0.11 1.00
0.23 0.42 0.26 1.00
-0.16 0.08 0.29 0.12 1.00
0.27 0.26 0.17 0.65 -0.08 1.00
-0.01 0.17 -0.13 0.34 0.07 0.25 1.00
b
Scr
siSTAT5A #1
siSTAT5A #2
siSIX5 #1
siSIX5 #2
siTCF21 #1
siTCF21 #2
mRNA relative level
P = 0.0034
P = 0.0145
P = 0.0003
P = 0.0016
P = 0.0003
P = 0.0208
P = 0.5898
P = 0.7686
P = 0.0004
P = 0.0002
P = 0.2592
P = 0.9105
STAT5A
TRIM21
SIX5
TCF21
c
chr11:4,377,535-4,448.913
p15.4 p15.3 p15.1 p14.3 p14.1 p13 p12 p11.2 p11.12 q11 q12.1 q12.3 q13.2 q13.4 q13.5
71 kb
4,380 kb
4,390 kb
4,400 kb
4,410 kb
ChIP-Atlas_STAT5A
[0-2.40]
ARP1_SRX9681372
[0-0.79]
MCF-7_SRX9932045
[0-0.08]
Mesoderm_SRX190177
[0-0.40]
Refseq Genes
TRIM21
d
Bits
e
TRIM21
F1 F2 F3 F0 F4 F5
BS1 BS2 BS3 BS0 BS4 BS5
R1 R2 R3 R0 R4 R5
-2000 -1800 -1600 -1400 -1200 -1000 -800 -600 -400 -200 0 200
STAT5A-motif-EPD
STAT5A-motif-TFDB
f
EGAS00001000925
81 SCLC tissues
r = -0.38
P = 4.64E-04
PI3K pathway activity
STAT5A mRNA level
g
HRA003419
107 SCLC tissues
r = 0.47
P = 2.71E-07
STAT5A mRNA level
TRIM21 mRNA level
h
OMIX002489
112 SCLC tissues
r = 0.60
P = 1.73E-12
STAT5A protien level
TRIM21 protien level
i
DMS273
SHP77
PARP1
TRIM21
STAT5A
mRNA relative level
P =0.7939
P = 0.8131
P = 0.6062
P = 0.8183
P = 0.6254
P = 0.9746
P = 0.0691
P = 0.0030
P = 3.18E-05
P = 0.0847
P = 0.0019
P = 0.0005
P = 0.0054
P = 0.0004
P = 2.08E-05
P = 0.1531
P = 0.0501
P = 0.0127
P = 0.5831
P = 0.3234
P = 0.5778
P = 0.7923
P = 0.9456
P = 0.4266
P = 0.2391
P = 0.0118
P = 0.0046
P = 0.2819
P = 0.0416
P = 0.0263
P =0.0024
P = 0.0014
P = 0.0005
P = 0.0789
P = 0.0157
P = 0.0052
DMSO 0.5 1 2 DMSO 5 10 20
PKI-587 (μM)
SC79 (μM)
j
Scr
shSTAT5A
STAT5A
DMS273
SHP77
mRNA relative level
P = 7.62E-05
P = 0.0001
P = 0.0041
P = 0.0014
P = 0.7621
P = 0.9639
P = 0.0001
P = 0.0002
P = 0.0029
P = 0.0018
P = 0.9377
P = 0.9049
STAT5A
TRIM21
PARP1
k
DMS273
SHP77
Stafia-1 (μM)
DMSO 5 10 20
(kDa)
STAT5A
p-STAT5A
TRIM21
PARP1
β-Actin
100
100
55
100
40

Supplementary Fig. 7 STAT5A acts as a transcription factor inhibiting *TRIM21* via the PI3K/AKT pathway. a Correlations between the *TRIM21* mRNA levels and the expression of potential transcription factors (TFs) ZEB1, HIC1, SIX5, STAT5A, TCF3, and TCF21 in 107 SCLC tissues from the HRA003419 dataset. **b** RT-qPCR analysis of *TRIM21*, *STAT5A*, *SIX5*, and *TCF21* expression after knocking down *STAT5A*, *SIX5*, or *TCF21* in HEK293T cells. **c** Identification of STAT5A peaks in the promoter of the *TRIM21* gene from the ChIP-Atlas database. **d** The consensus DNA-binding motif of STAT5A derived from the JASPAR database. **e** Schematic diagram of primers design strategy for STAT5A binding sites (BSs) in the *TRIM21* promoter. Five putative STAT5A BSs were predicted using the Eukaryotic Promoter Database (EPD) and AnimalTFDB 4.0 (TFDB). BS0 serves as a randomly selected negative control. **f** Correlation between PI3K/AKT pathway activity and *STAT5A* mRNA levels in SCLC tissues from the European Genome-phenome Archive database (EGAS00001000925, n = 81; 95% confidence interval (CI), -0.55 to -0.18). **g** Correlation between *TRIM21* and *STAT5A* mRNA levels in SCLC tumor tissues from the GSA database (HRA003419, n = 107; 95% CI, 0.31 to 0.61). **h** Correlation between TRIM21 and STAT5A protein levels in SCLC tumor tissues from the OMIX database (OMIX002489, n = 112; 95% CI, 0.47 to 0.71). Two-tailed P values are reported in (**a**, **f**–**h**). **i** Dose-response of *STAT5A*, *TRIM21*, and *PARP1* mRNA levels in SCLC cells. DMS273 and SHP77 cells were treated with the indicated concentrations of PKI-587 or SC-79 for 24 hours, followed by RT-qPCR analysis of the respective mRNA. **j** *STAT5A*, *TRIM21*, and *PARP1* mRNA expression after *STAT5A* knockdown and rescue. mRNA levels were assessed by RT-qPCR in DMS273 and SHP77 cells stably expressing sh*STAT5*A, followed by *STAT5A* overexpression. **k** Western blot analysis of STAT5A, p-STAT5A, TRIM21, and PARP1 in DMS273 and SHP77 cells treated with increasing concentrations of Stafia-1 for 24 hours. Data in (**b**, **i**, **j**) are from three independent experiments and are presented as mean ± SEM. Statistical significance was determined using two-tailed unpaired Student's *t*-tests. Source data are provided as a Source Data file.

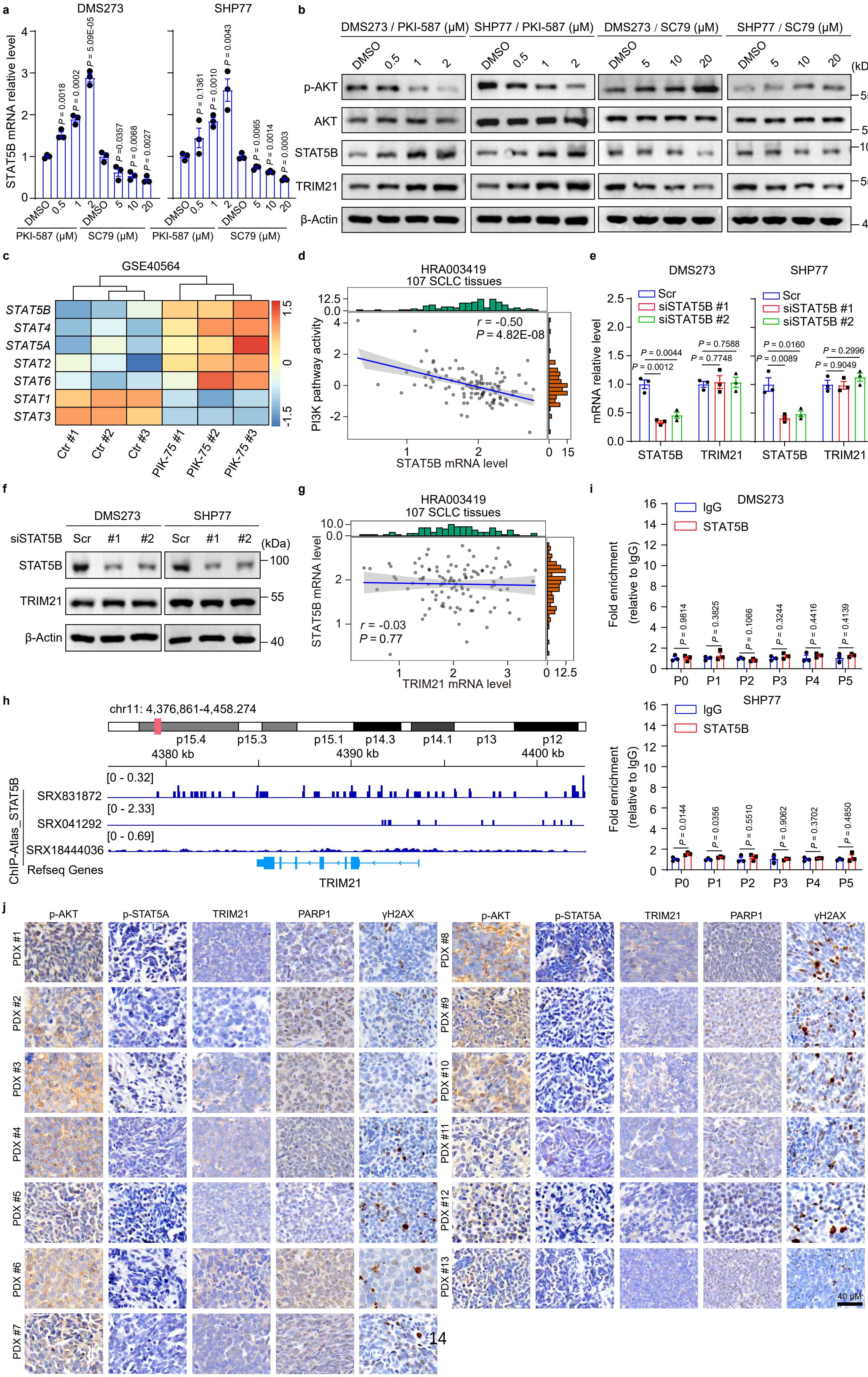
a
DMS273
SHP77
STAT5B mRNA relative level
P = 0.0018
P = 0.0002
P = 5.09E-05
P = 0.0357
P = 0.0068
P = 0.0027
P = 0.1361
P = 0.0010
P = 0.0043
P = 0.0065
P = 0.0014
P = 0.0003
DMSO 0.5 1 2 DMSO 5 10 20
PKI-587 (μM)
SC79 (μM)
b
DMS273 / PKI-587 (μM)
SHP77 / PKI-587 (μM)
DMS273 / SC79 (μM)
SHP77 / SC79 (μM)
DMSO 0.5 1 2
DMSO 5 10 20
(kDa)
p-AKT
AKT
STAT5B
TRIM21
β-Actin
55
55
100
55
40
c
GSE40564
STAT5B
STAT4
STAT5A
STAT2
STAT6
STAT1
STAT3
Ctr #1
Ctr #2
Ctr #3
PIK-75 #1
PIK-75 #2
PIK-75 #3
1.5
0
-1.5
d
HRA003419
107 SCLC tissues
r = -0.50
P = 4.82E-08
PI3K pathway activity
STAT5B mRNA level
e
DMS273
SHP77
Scr
siSTAT5B #1
siSTAT5B #2
mRNA relative level
P = 0.0044
P = 0.0012
P = 0.7588
P = 0.7748
P = 0.0160
P = 0.0089
P = 0.2996
P = 0.9049
STAT5B
TRIM21
f
DMS273
SHP77
siSTAT5B Scr #1 #2 Scr #1 #2
(kDa)
STAT5B
TRIM21
β-Actin
100
55
40
g
HRA003419
107 SCLC tissues
STAT5B mRNA level
r = -0.03
P = 0.77
TRIM21 mRNA level
h
chr11: 4,376,861-4,458.274
p15.4 p15.3 p15.1 p14.3 p14.1 p13 p12
4380 kb
4390 kb
4400 kb
[0 - 0.32]
SRX831872
[0 - 2.33]
SRX041292
[0 - 0.69]
SRX18444036
ChIP-Atlas_STAT5B
Refseq Genes
TRIM21
i
DMS273
IgG
STAT5B
Fold enrichment
(relative to IgG)
P = 0.9814
P = 0.3825
P = 0.1066
P = 0.3244
P = 0.4416
P = 0.4139
P0 P1 P2 P3 P4 P5
SHP77
IgG
STAT5B
Fold enrichment
(relative to IgG)
P = 0.0144
P = 0.0356
P = 0.5510
P = 0.9062
P = 0.3702
P = 0.4850
P0 P1 P2 P3 P4 P5
j
p-AKT
p-STAT5A
TRIM21
PARP1
γH2AX
PDX #1
PDX #2
PDX #3
PDX #4
PDX #5
PDX #6
PDX #7
PDX #8
PDX #9
PDX #10
PDX #11
PDX #12
PDX #13
40 μM

Supplementary Fig. 8 STAT5B is not a transcriptional regulator of TRIM21. a RT-qPCR analysis of *STAT5B* expression in DMS273 and SHP77 cells treated with a concentration gradient of PKI-587 or SC-79 for 24 hours. **b** Western blot analysis of p-AKT, AKT, STAT5B, and TRIM21 in DMS273 and SHP77 cells following treatment with PKI-587 or SC-79 for 24 hours. **c** Heatmap of STAT family members expression (*STAT1*, *STAT2*, *STAT3*, *STAT4*, *STAT5A/B*, and *STAT6*) from RNA-seq data (GSE40564 dataset) in SCLC cells treated with the PI3K/AKT inhibitor PIK-75. **d** Correlation analysis between PI3K/AKT pathway activity scores and *STAT5B* mRNA levels in SCLC tissues (HRA003419, *n* = 107; 95% confidence interval (CI), -0.63 to -0.34). **e**, **f** RT-qPCR and Western blot analyses of STAT5B and TRIM21 expression following *STAT5B* knockdown (si*STAT5B*). The mRNA (**e**) and protein (**f**) levels of STAT5B and TRIM21 were measured. **g** Correlation analysis between PI3K/AKT pathway activity scores and *STAT5B* mRNA levels in SCLC tissues (HRA003419, *n* = 107; 95% CI, -0.22 to 0.16). **h** Identification of STAT5B peaks in the promoter of the *TRIM21* gene from the ChIP-Atlas database. **i** STAT5B did not bind to the *TRIM21* promoter assessed by ChIP-qPCR in DMS273 and SHP77 cells. **j** IHC profiling of SCLC patient-derived xenograft (PDX) models. 13 PDX models (PDX #1–#13) were analyzed by IHC for key biomarkers: p-AKT, p-STAT5A, TRIM21, PARP1, and γH2AX. Scale bars, 40 μm. Data in (**a**, **e**, **i**) are from three independent experiments and are presented as mean ± SEM. Statistical significance was determined using two-tailed unpaired Student's *t*-tests. Two-tailed *P* values are reported in (**d**, **g**). Source data are provided as a Source Data file.

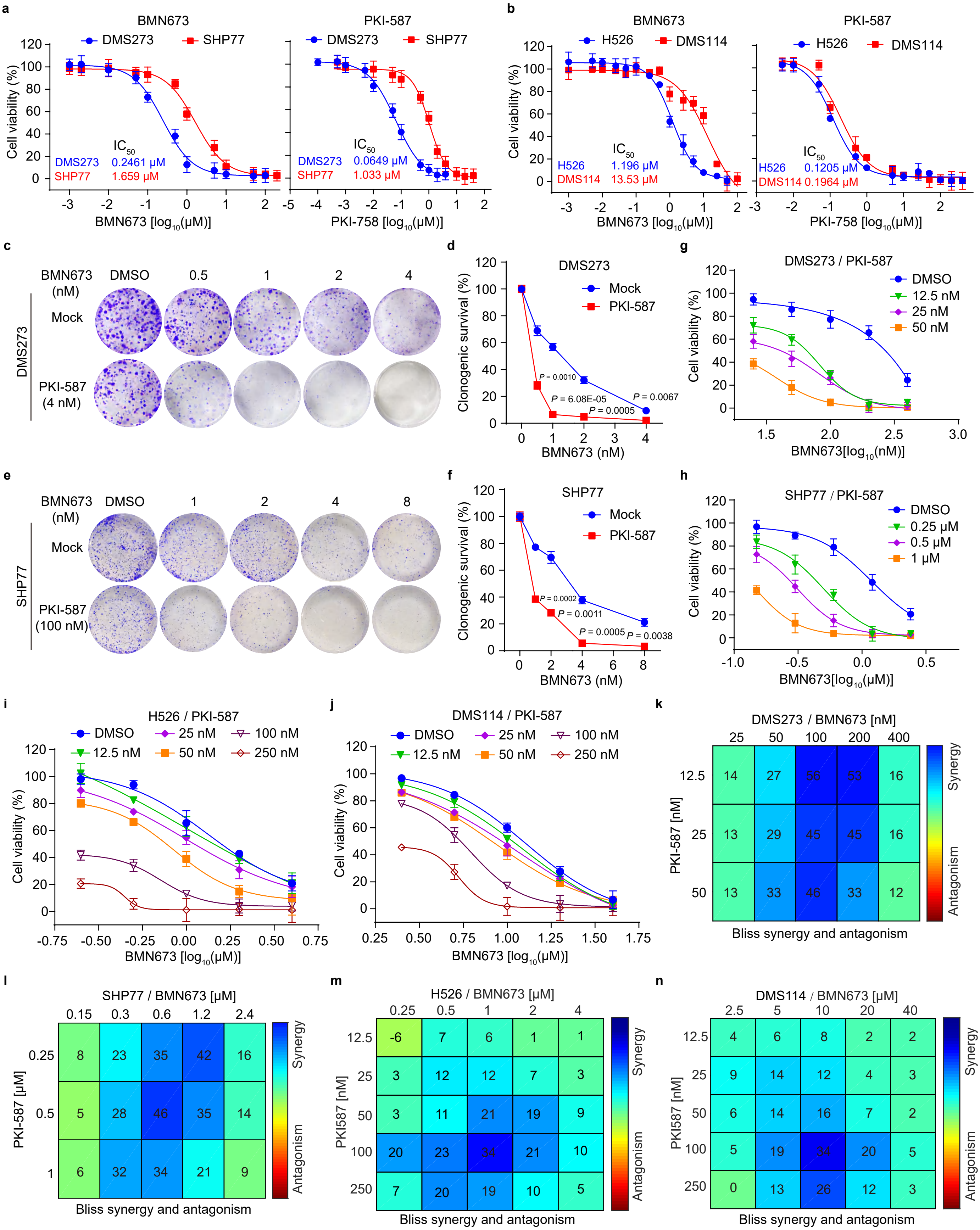
a
BMN673
DMS273
SHP77
Cell viability (%)
IC50
DMS273 0.2461 µM
SHP77 1.659 µM
BMN673 [log10(µM)]
PKI-587
DMS273
SHP77
IC50
DMS273 0.0649 µM
SHP77 1.033 µM
PKI-758 [log10(µM)]
b
BMN673
H526
DMS114
Cell viability (%)
IC50
H526 1.196 µM
DMS114 13.53 µM
BMN673 [log10(µM)]
PKI-587
H526
DMS114
IC50
H526 0.1205 µM
DMS114 0.1964 µM
PKI-758 [log10(µM)]
c
BMN673 (nM)
DMSO
0.5
1
2
4
DMS273
Mock
PKI-587 (4 nM)
d
DMS273
Mock
PKI-587
Clonogenic survival (%)
P = 0.0010
P = 6.08E-05
P = 0.0005
P = 0.0067
BMN673 (nM)
g
DMS273 / PKI-587
DMSO
12.5 nM
25 nM
50 nM
Cell viability (%)
BMN673[log10(nM)]
e
BMN673 (nM)
DMSO
1
2
4
8
SHP77
Mock
PKI-587 (100 nM)
f
SHP77
Mock
PKI-587
Clonogenic survival (%)
P = 0.0002
P = 0.0011
P = 0.0005
P = 0.0038
BMN673 (nM)
h
SHP77 / PKI-587
DMSO
0.25 µM
0.5 µM
1 µM
Cell viability (%)
BMN673[log10(µM)]
i
H526 / PKI-587
DMSO
12.5 nM
25 nM
50 nM
100 nM
250 nM
Cell viability (%)
BMN673 [log10(µM)]
j
DMS114 / PKI-587
DMSO
12.5 nM
25 nM
50 nM
100 nM
250 nM
Cell viability (%)
BMN673 [log10(µM)]
k
DMS273 / BMN673 [nM]
25 50 100 200 400
PKI-587 [nM]
12.5: 14 27 56 53 16
25: 13 29 45 45 16
50: 13 33 46 33 12
Synergy
Antagonism
Bliss synergy and antagonism
l
SHP77 / BMN673 [µM]
0.15 0.3 0.6 1.2 2.4
PKI-587 [µM]
0.25: 8 23 35 42 16
0.5: 5 28 46 35 14
1: 6 32 34 21 9
Synergy
Antagonism
Bliss synergy and antagonism
m
H526 / BMN673 [µM]
0.25 0.5 1 2 4
PKI587 [nM]
12.5: -6 7 6 1 1
25: 3 12 12 7 3
50: 3 11 21 19 9
100: 20 23 34 21 10
250: 7 20 19 10 5
Synergy
Antagonism
Bliss synergy and antagonism
n
DMS114 / BMN673 [µM]
2.5 5 10 20 40
PKI587 [nM]
12.5: 4 6 8 2 2
25: 9 14 12 4 3
50: 6 14 16 7 2
100: 5 19 34 20 5
250: 0 13 26 12 3
Synergy
Antagonism
Bliss synergy and antagonism

Supplementary Fig. 9 Inhibition of PI3K/AKT synergizes with PARPi in SCLC cells. a, **b** Growth inhibition curves of BMN673 and PKI-587 in DMS273, SHP77, H526, and DMS114 cell lines. The cells were treated with different concentrations of BMN673 or PKI-587 for 72 hours. The cell viability was then assessed by the CellTiter-Glo assay. The Half-lethal dose (IC_{50}) values were determined from the sigmoidal dose-response curves. **c**, **e** Colony formation assays demonstrating the effect of PKI-587 on the cytotoxicity of PARPi in SCLC cells. DMS273 (**c**) and SHP77 (**e**) cells were exposed to BMN673 in the presence of PKI-587 for a period of 7–10 days. **d**, **f** Drug-response curves for the colony formation assays presented in (**c**) and (**e**). **g**–**j** Dose-response profiles of BMN673 combined with PKI-587 in DMS273 (**g**), SHP77 (**h**), H526 (**i**), and DMS114 (**j**) cells. Cells were treated with increasing concentrations of BMN673 and PKI-587 for 72 hours, followed by cell viability measurement using CellTiter-Glo luminescent assay. **k**–**n** Bliss synergy analysis of the combination of BMN673 and PKI-587 in DMS273 (**k**), SHP77 (**l**), H526 (**m**), and DMS114 (**n**) cells. Synergy indices were calculated using Combenefit software (CRUK Cambridge Institute) based on the dose-response data from (**g**–**j**). Data in (**a**, **b**, **d**, **f**, **g**–**j**), derived from three independent experiments, are presented as mean ± SEM. Source data are provided as a Source Data file.

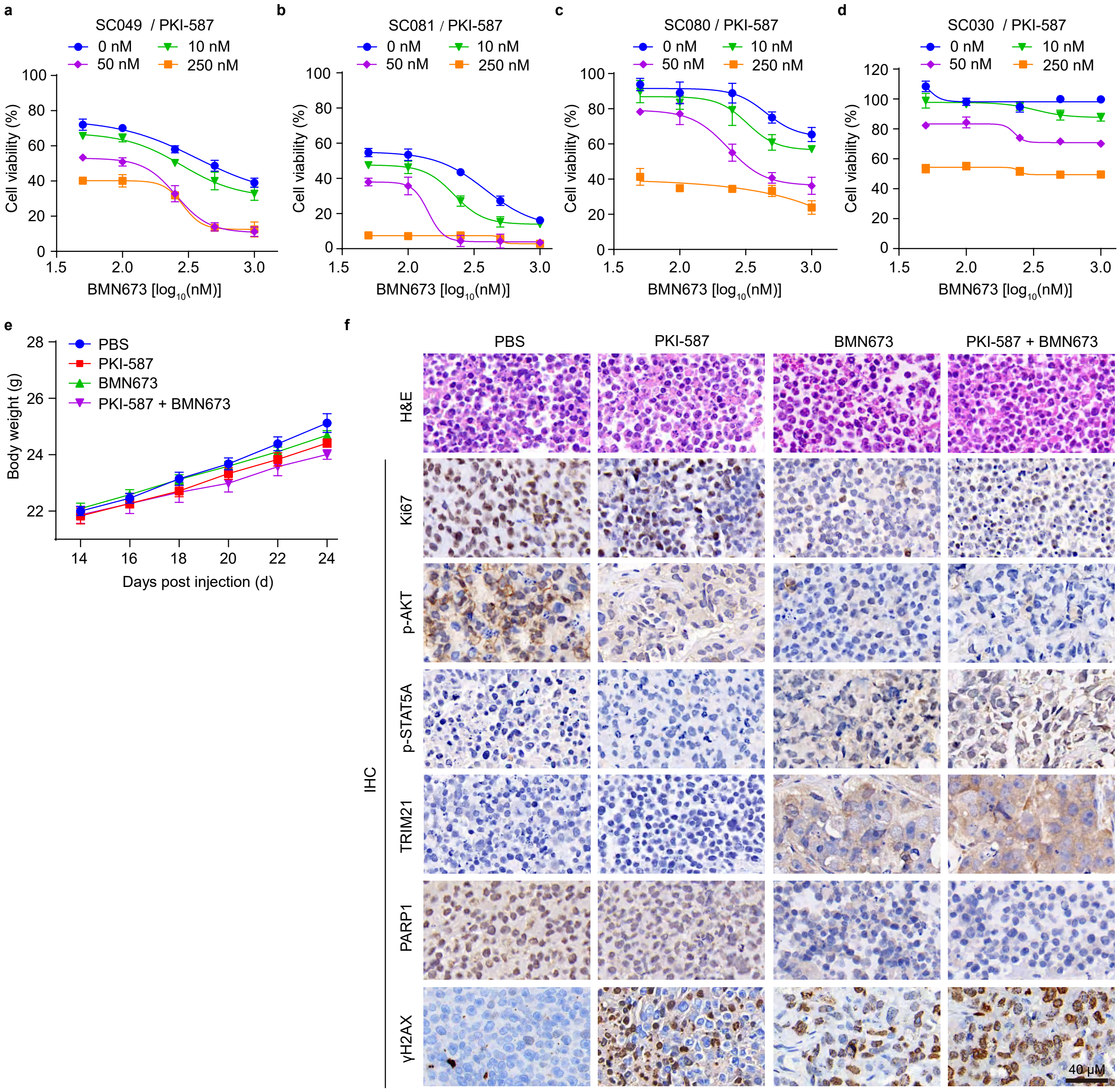

Supplementary Fig. 10 Co-inhibition of PI3K/AKT and PARP results in synergy *in vivo*. **a**–**d** Combinatorial dose-response profiles illustrating the effects of BMN673 and PKI-587 co-treatment across all four subtypes (*A*: SC049, **a**; *A* + *N*: SC081, **b**; *P*: SC080, **c**; *Y*: SC030, **d**) following 72 hours of exposure, measured by CellTiter-Glo viability assays. **e** Body weight curves of xenograft mice during treatment with PKI-587, BMN673, or a combination of PKI-587 and BMN673. **f** Representative images of HE staining and immunohistochemistry for Ki67, p-AKT, p-STAT5A, TRIM21, PARP1, and γH2AX within tumor sections. Scale bars represent 40 μm. Data in (**a**–**d**) are from three independent experiments, and data in (**e**) are from 7 mice per group. All data are presented as mean ± SEM. Source data are provided as a Source Data file.

Uncropped scans of Supplementary Figure 1

Supplementary Fig. 1b

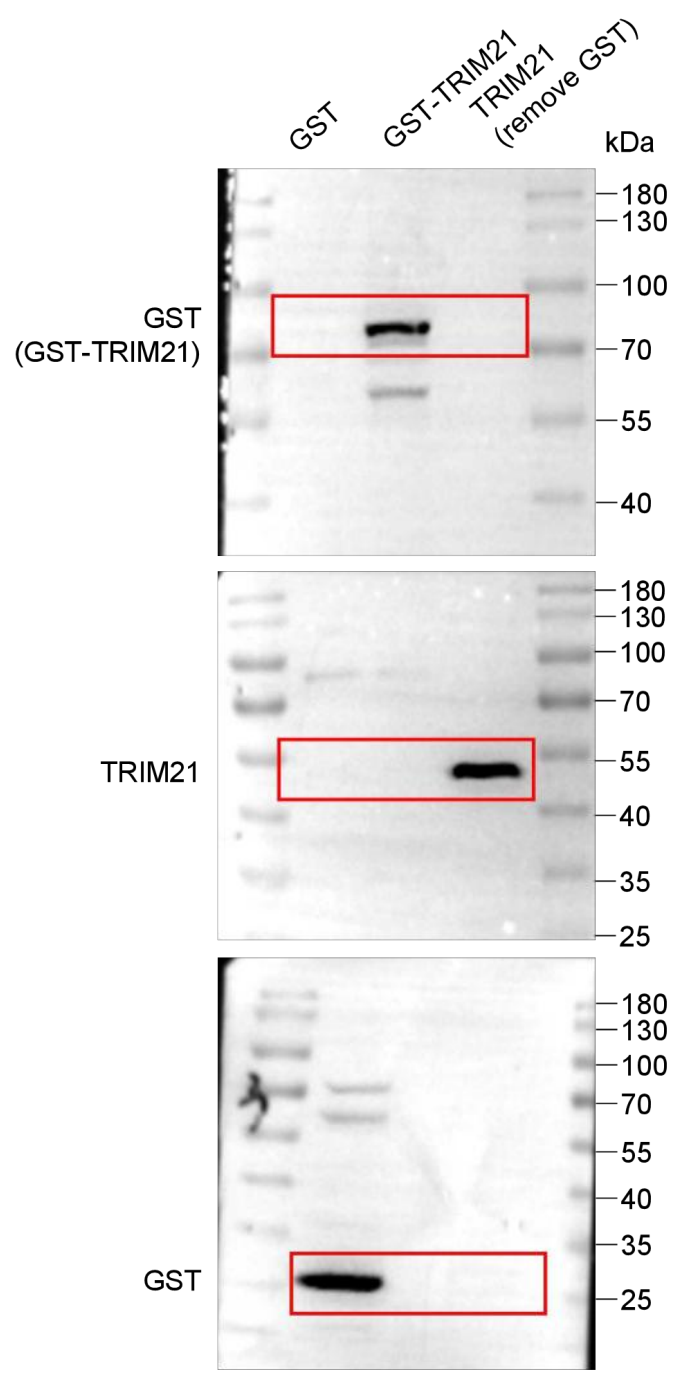

Supplementary Fig. 1c

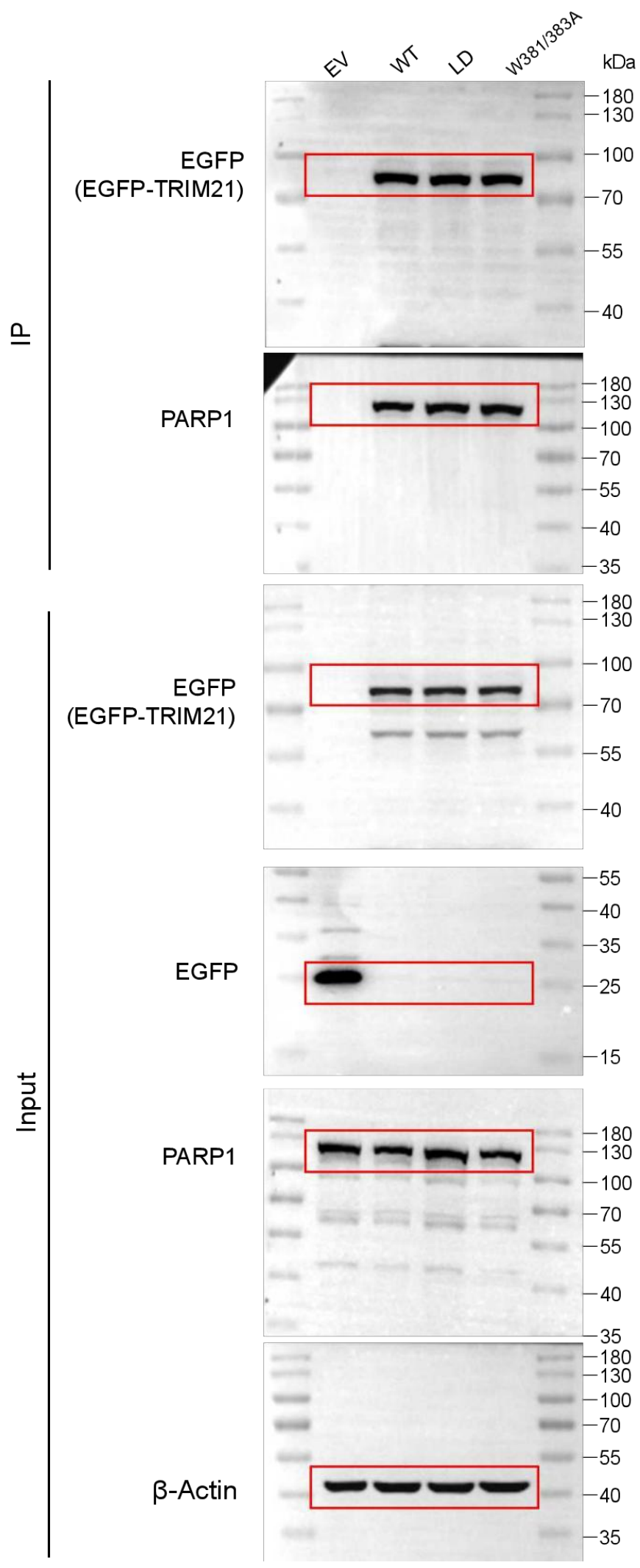

Supplementary Fig. 1e

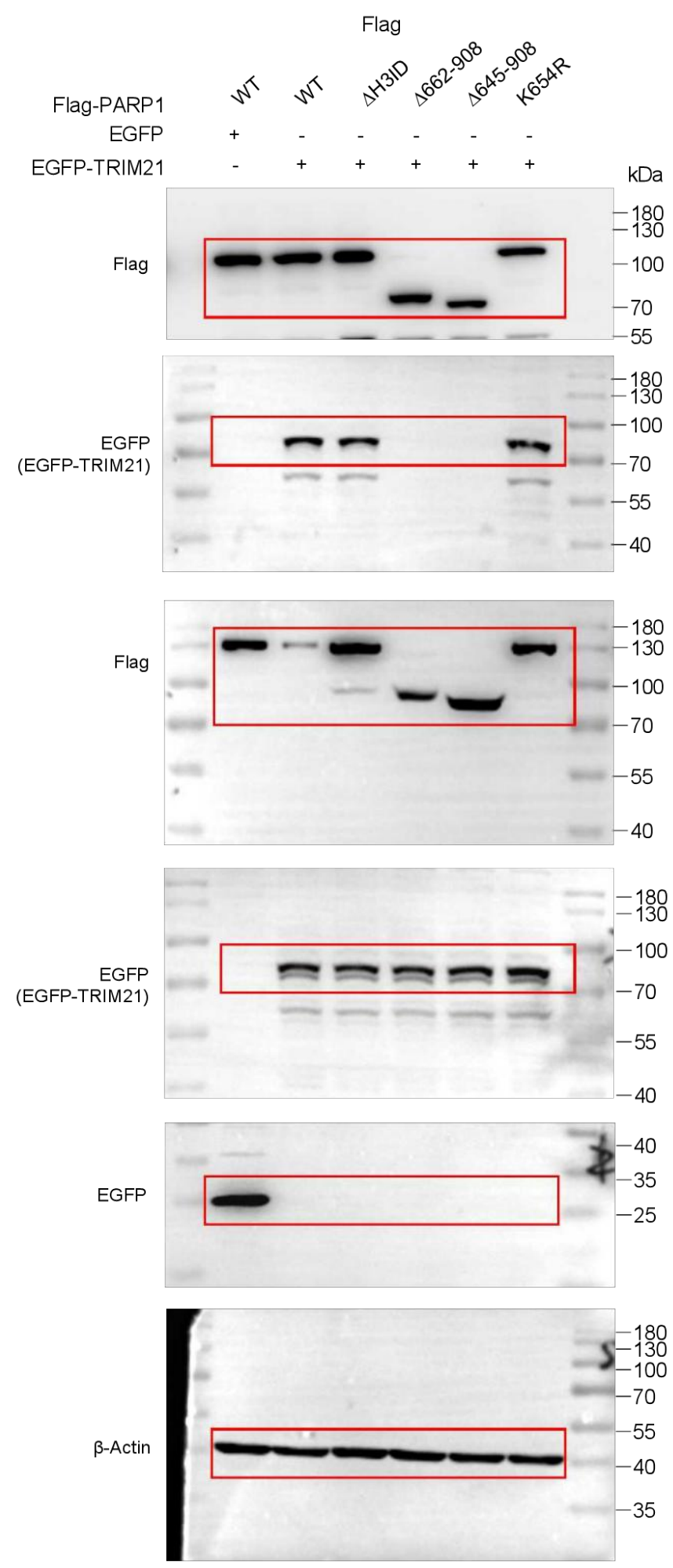

Uncropped scans of Supplementary Figure 2

Supplementary Fig. 2a

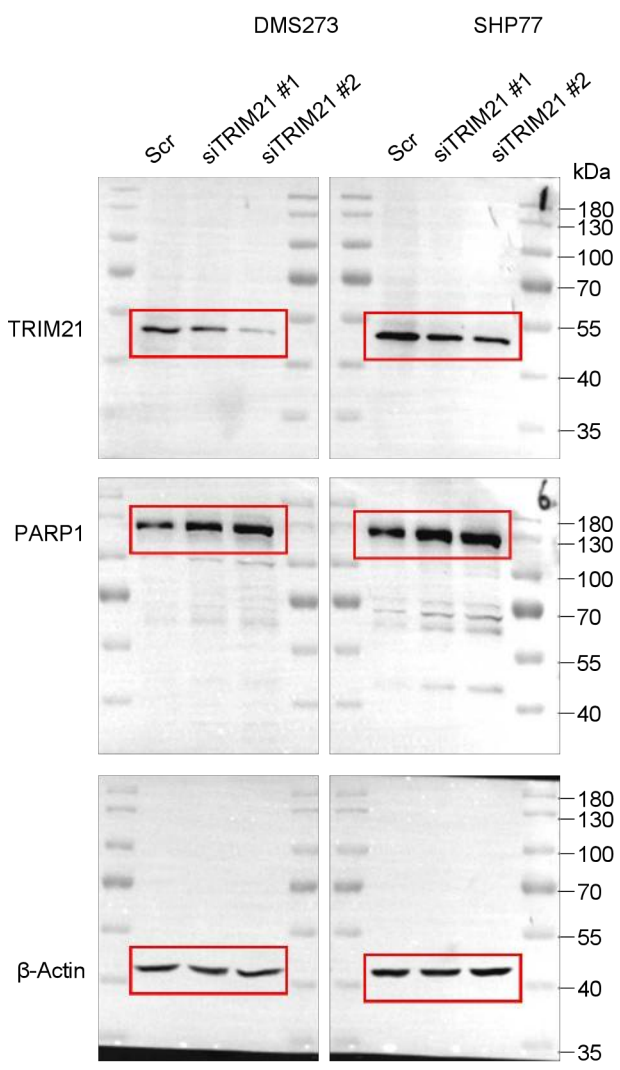

Supplementary Fig. 2e

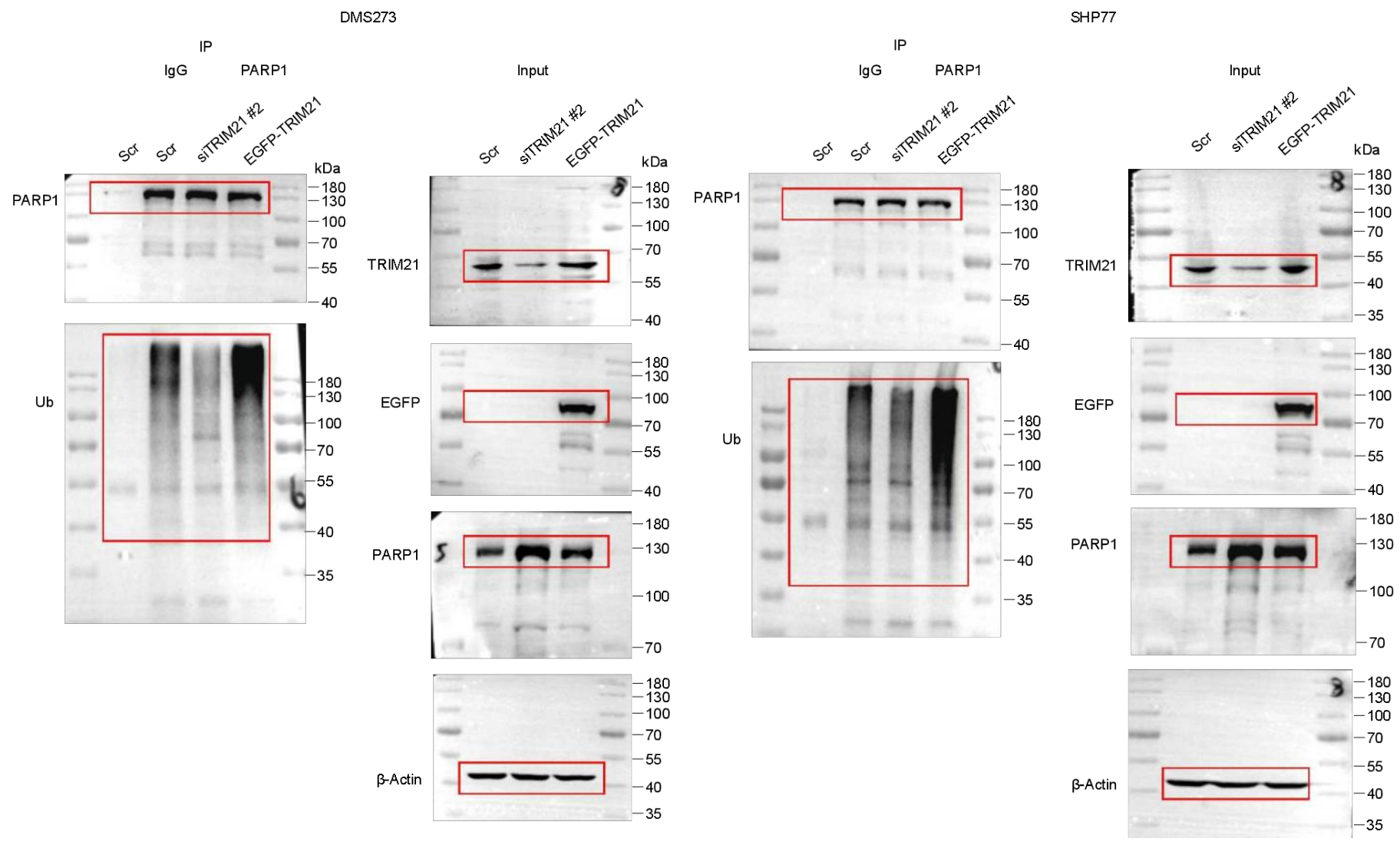

Supplementary Fig. 2f

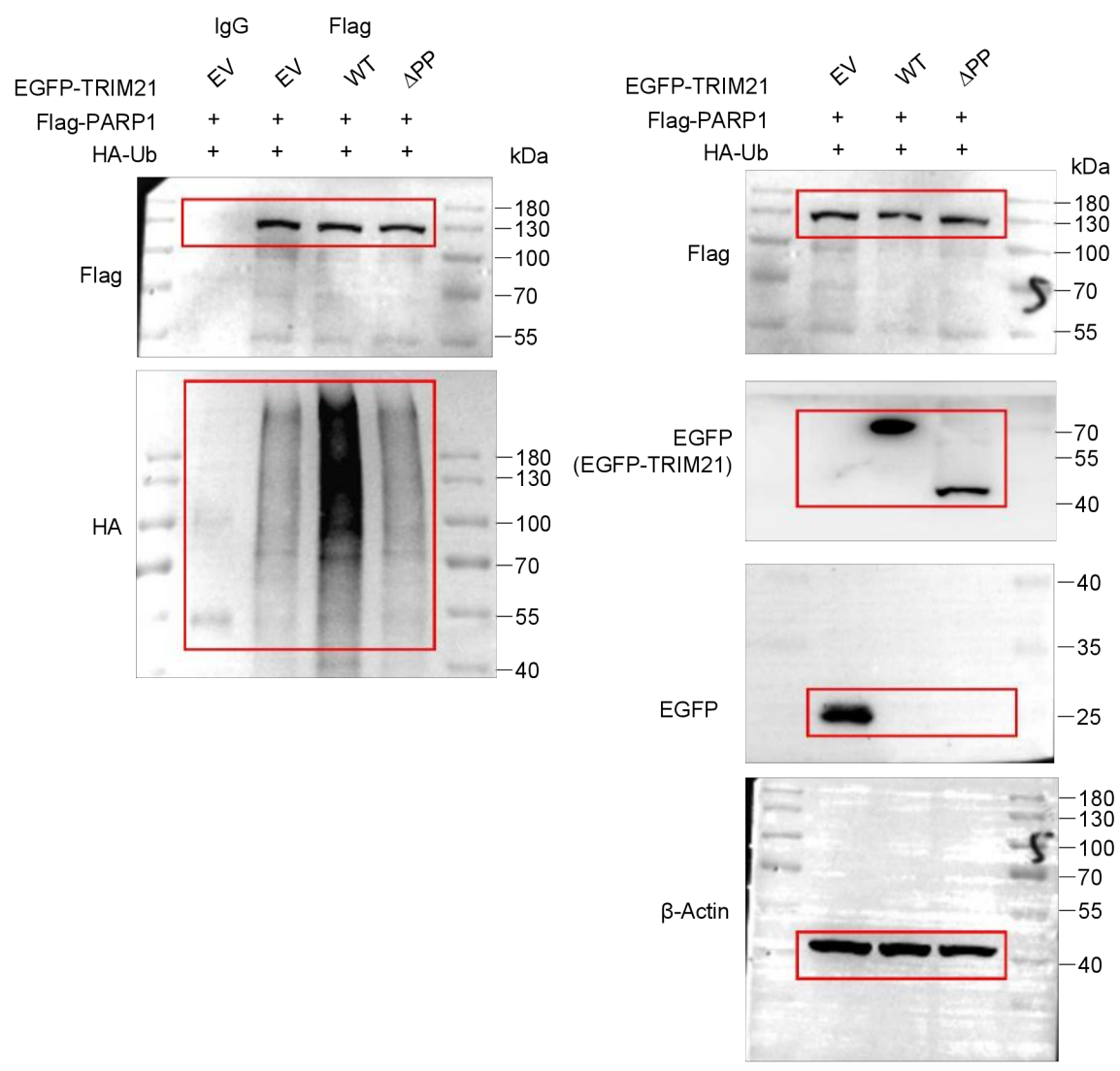

Supplementary Fig. 2g

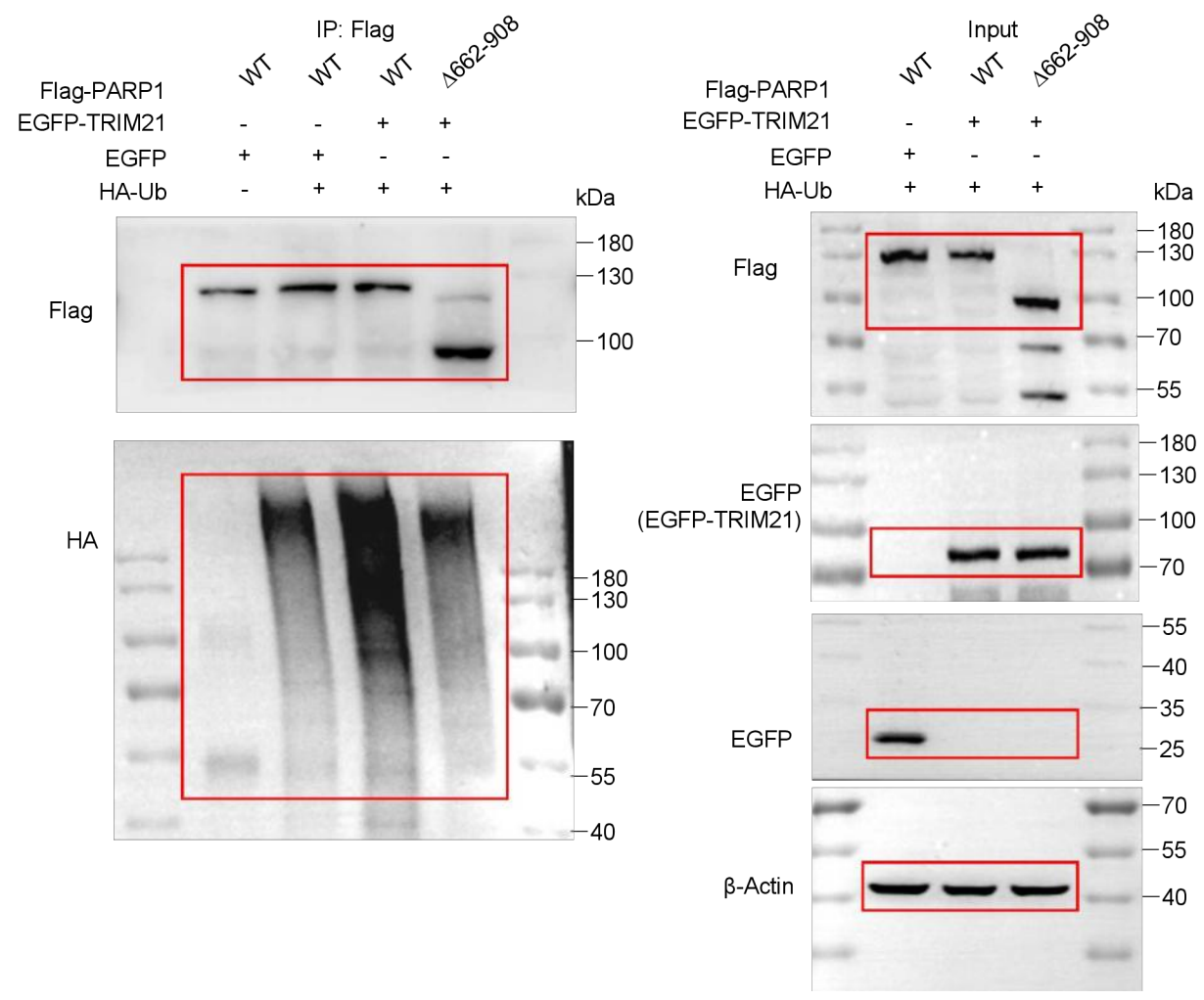

Supplementary Fig. 2h

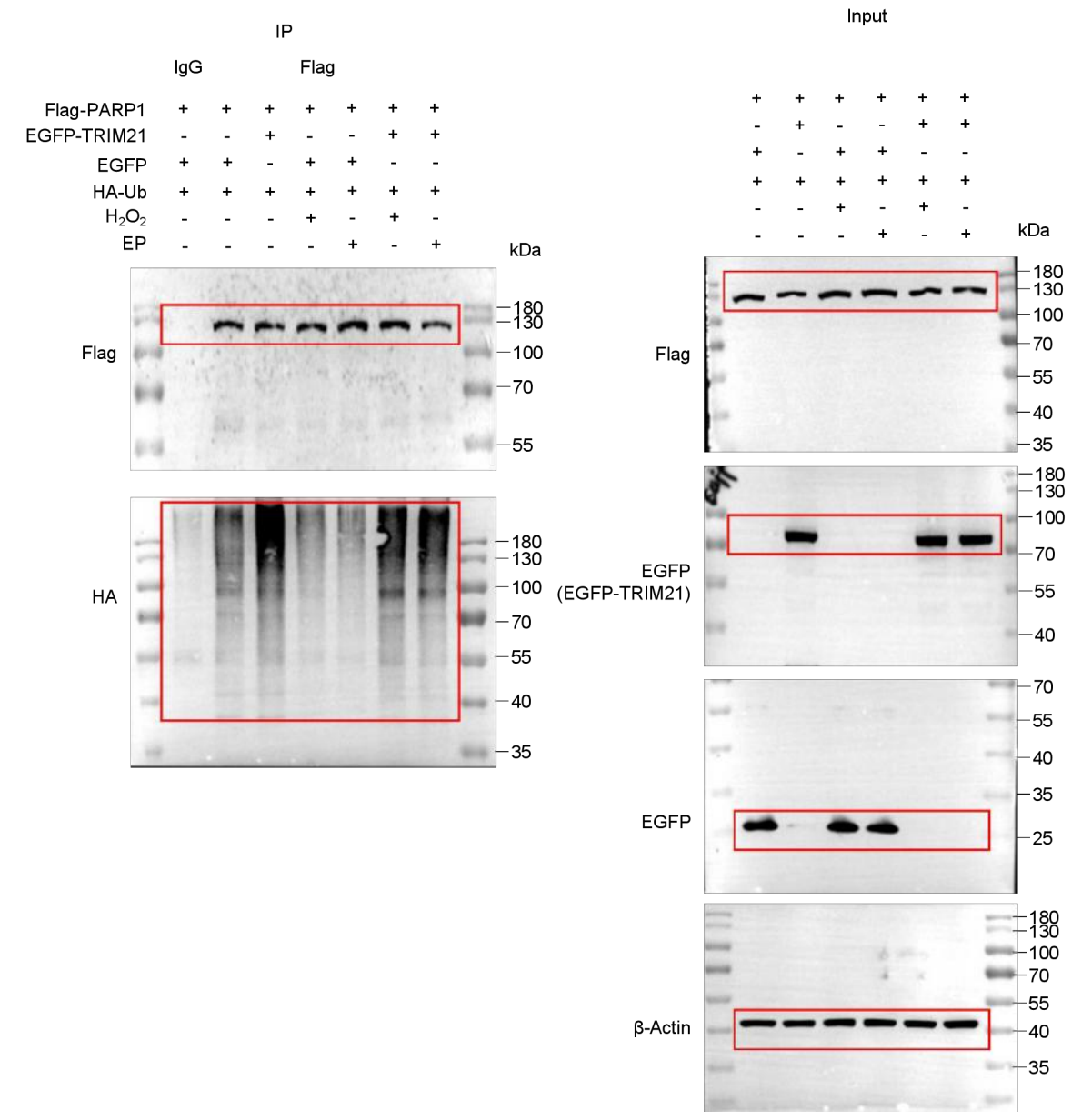

Supplementary Fig. 2i

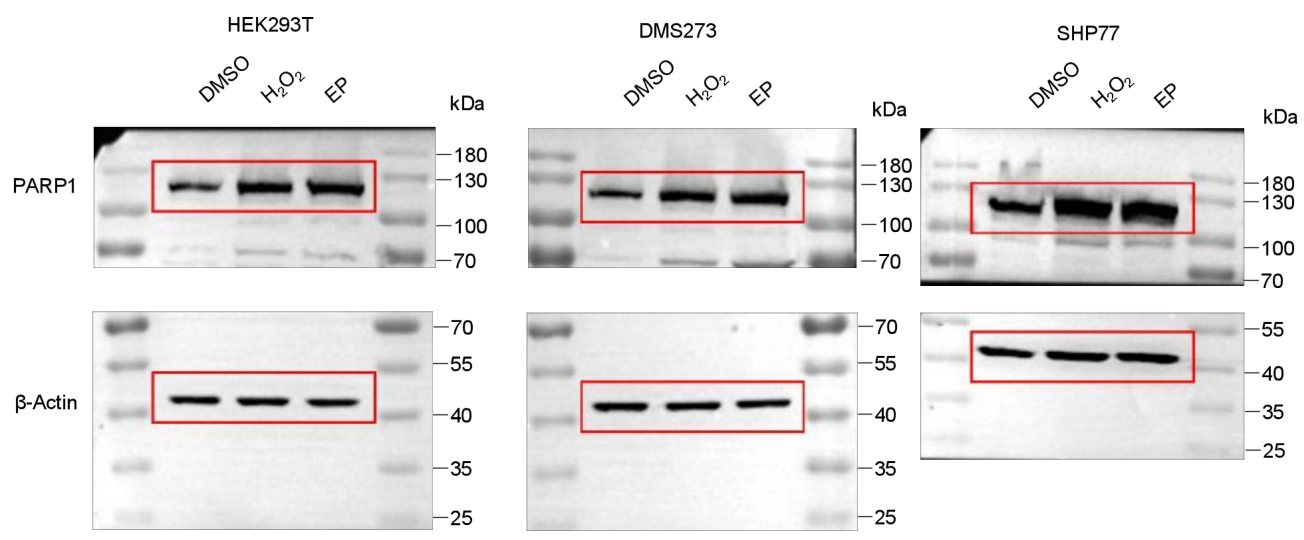

Uncropped scans of Supplementary Figure 4

Supplementary Fig. 4a

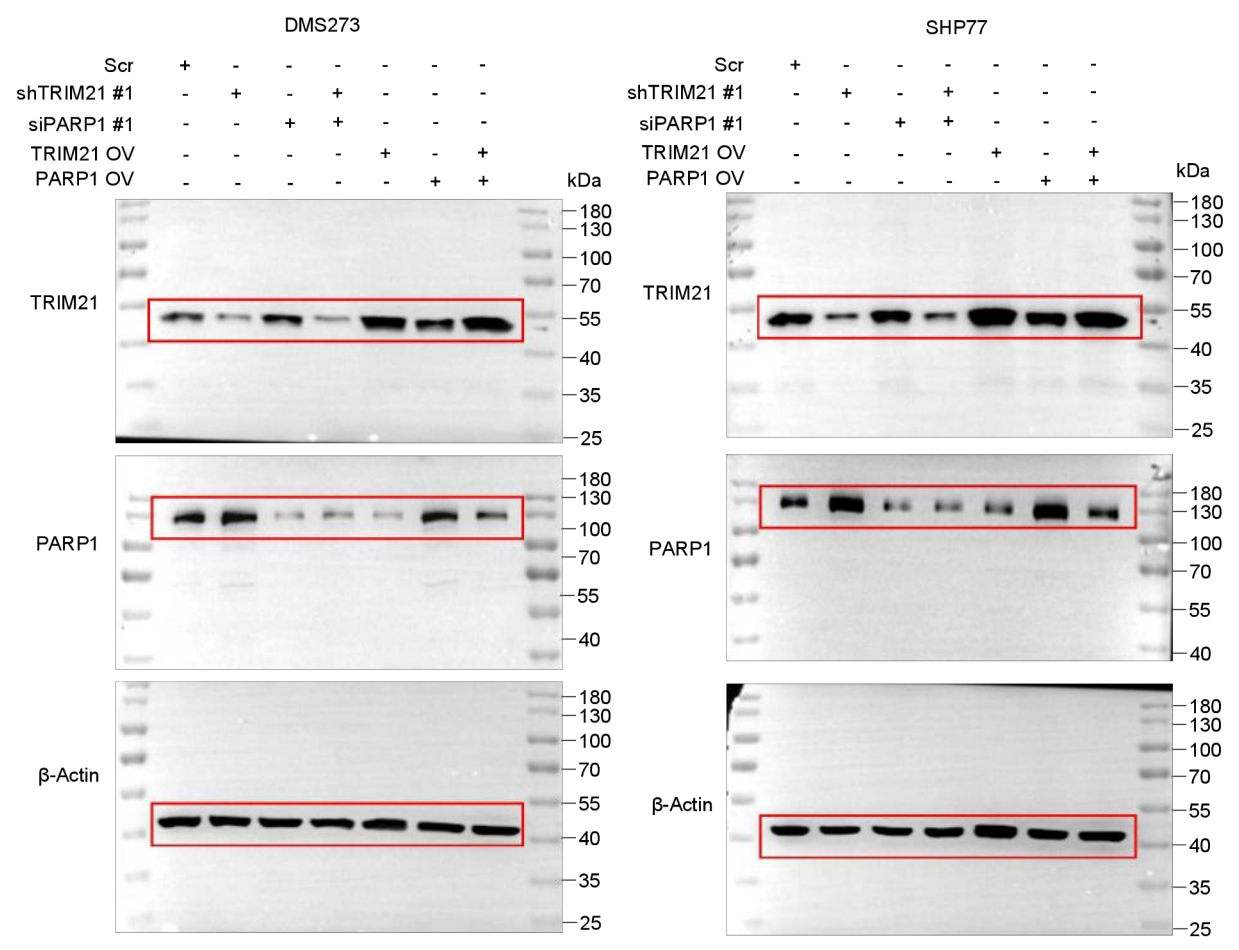

Supplementary Fig. 4c

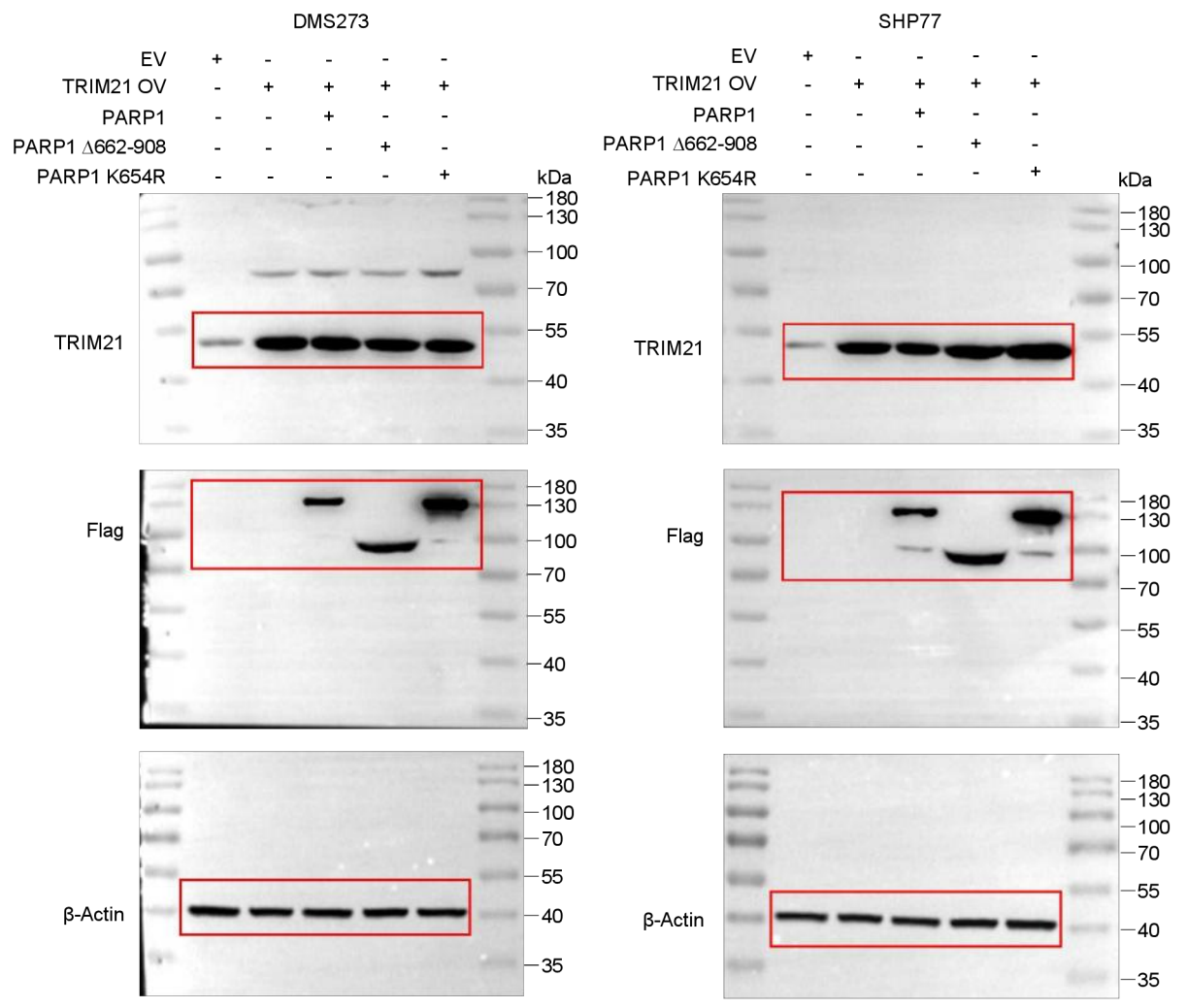

Uncropped scans of Supplementary Figure 6

Supplementary Fig. 6e

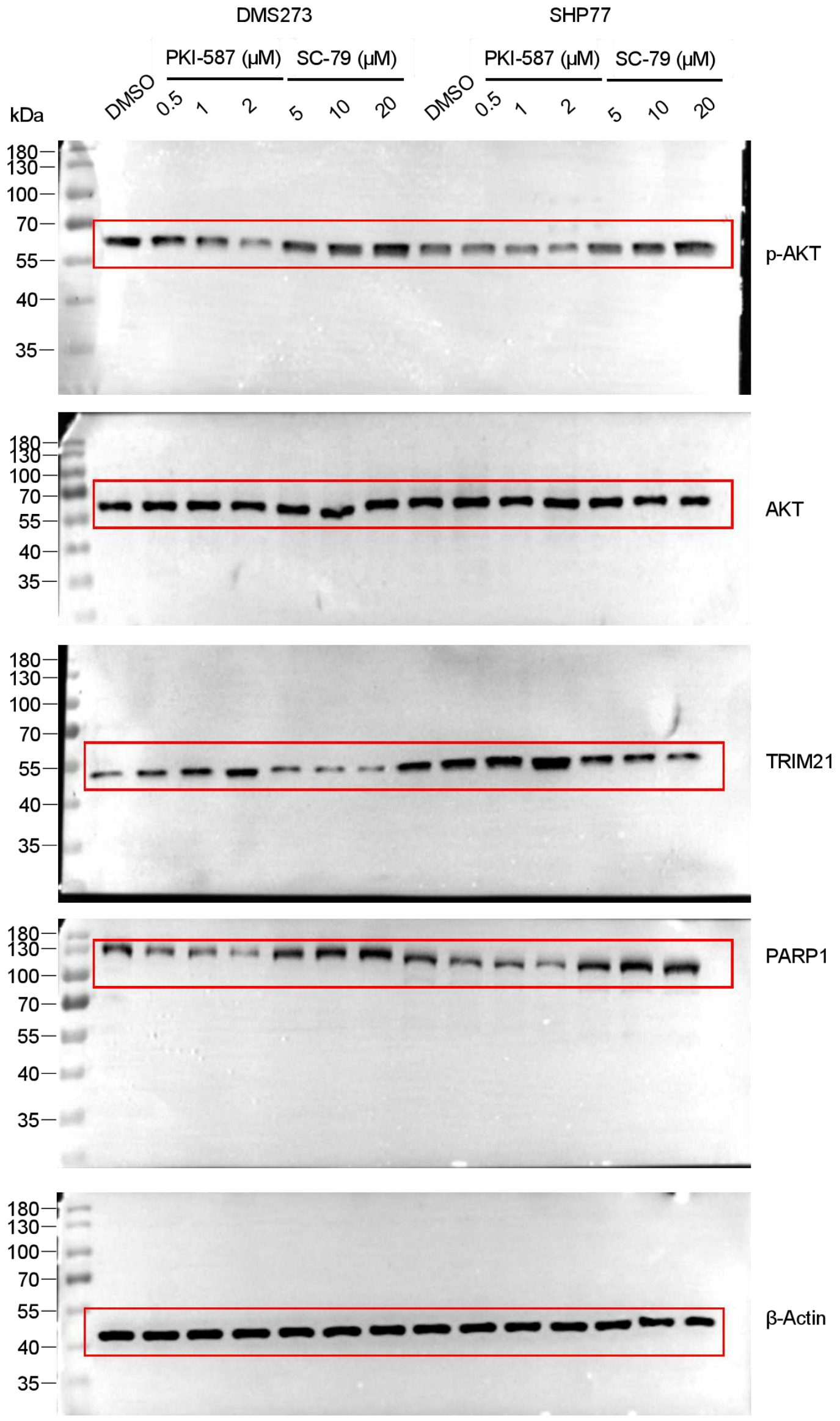

Uncropped scans of Supplementary Figure 7

Supplementary Fig. 7k

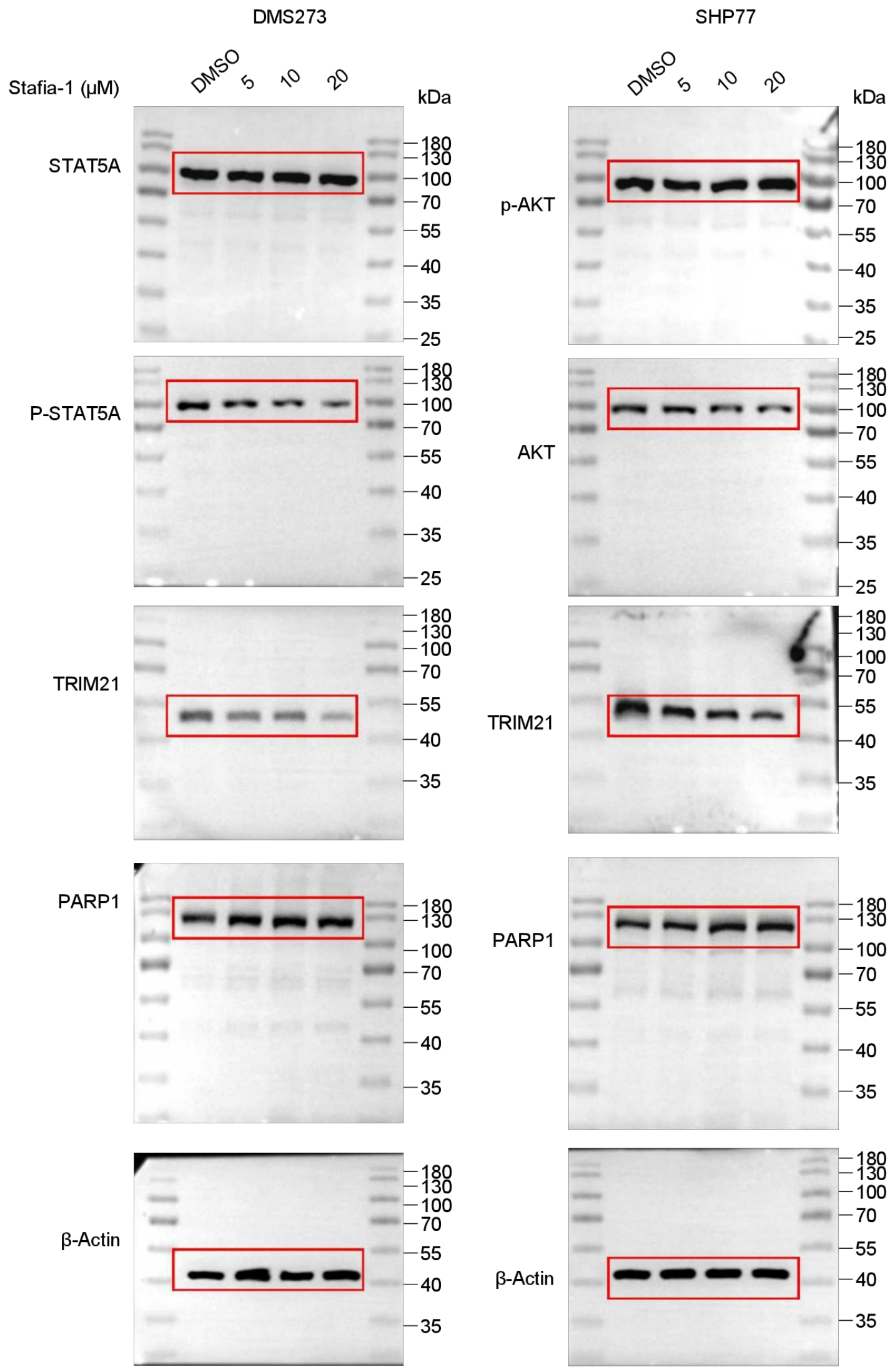

Uncropped scans of Supplementary Figure 8

Supplementary Fig. 8b

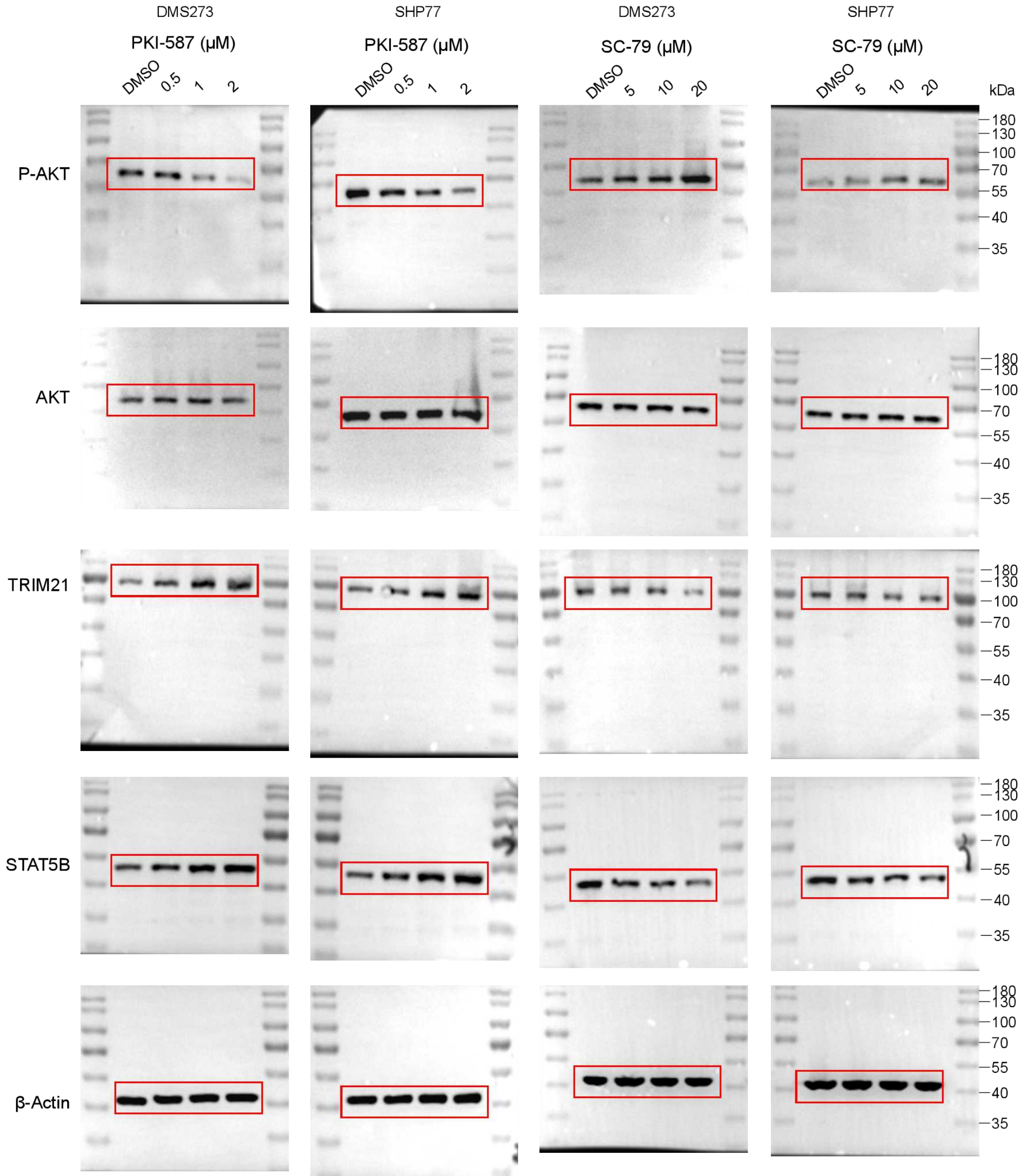

Supplementary Fig. 8f

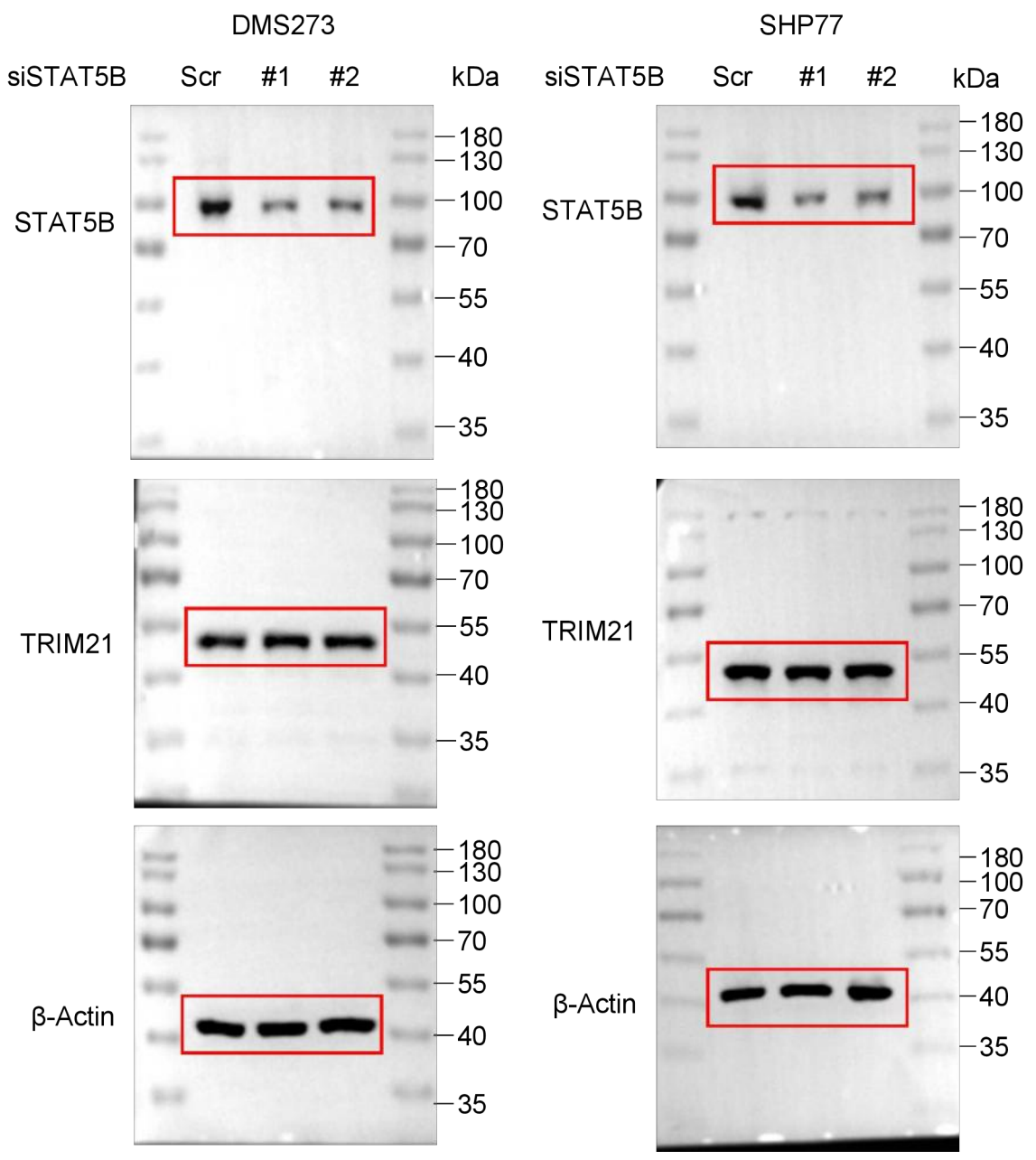

Supplement: Supplementary file 1 [file 41467_2026_73271_MOESM1_ESM.pdf]
