# Supplementary material for: TRIM21-mediated ubiquitination of PARP1 regulated by the PI3K/AKT-STAT5A axis suppresses small cell lung cancer
Source: Nat Commun. 2026 Jun 18;17:7704. doi: 10.1038/s41467-026-73271-1 (PMC13434705; doi:10.1038/s41467-026-73271-1)
Supplement: Supplementary file 2 [file 41467_2026_73271_MOESM2_ESM.pdf]

TRIM21-mediated ubiquitination of PARP1 regulated by the PI3K/AKT-STAT5A axis suppresses small cell lung cancer

Guozhen Cao[1, 2, 3, #], Gongfeng Li[1, 2, 3, #], Xiaolin Wang[2], Tengfei Zhang[4, 5], Xinhuang Yao[1], Peng Hou[1, 2, 3], Jiahui Zhang[1, 2, 3], Xiaopeng Guo[6], Jiarong Wang[3], Li Xiang[1], Hongbin Ji [4, 7], Chenchen Guo[4, 8, 9, *], Wenchu Lin[1, *]

[1] The Second Affiliated Hospital, School of Medicine, The Chinese University of Hong Kong, Shenzhen & Longgang District People's Hospital of Shenzhen, Shenzhen 518172, China

[2] University of Science and Technology of China, Hefei 230036, Anhui, China

[3] High Magnetic Field Laboratory, Chinese Academy of Sciences, Hefei 230031, Anhui, China

[4] Key Laboratory of Multi-Cell Systems, Shanghai Institute of Biochemistry and Cell Biology, Center for Excellence in Molecular Cell Science, Chinese Academy of Sciences, Shanghai 200031, China

[5] University of Chinese Academy of Sciences, Beijing 100049, China

[6] School of Life Science and Engineering, Lanzhou University of Technology, Lanzhou 730050, Gansu, China

[7] Westlake University School of Medicine, Hangzhou 310030, Zhejiang, China

[8] Shanghai Institute of Thoracic Oncology, Shanghai Chest Hospital, Shanghai Jiao Tong University School of Medicine, Shanghai 200030, China

[9] Shanghai Key Laboratory of Thoracic Tumor Biotherapy, Shanghai Chest Hospital, Shanghai Jiao Tong University School of Medicine, Shanghai 200030, China

Description of Additional Supplementary

Supplementary Data 1. PARP1-binding proteins profiled by label-free quantitative mass spectrometry in HEK293T cells. All proteomics data generated in this study have been deposited to the ProteomeXchange Consortium via the iProX partner repository (dataset identifier: PXD068168; accessible at http://proteomecentral.proteomexchange.org). The candidate PARP1-interacting proteins are provided in Supplementary Data 1.

Supplementary Data 2. This Excel file lists all antibodies used in this study (including source, catalog number, application and RRID) as well as all compounds and drugs (including supplier and catalog number).

Supplementary Data 3. This Excel file lists all oligonucleotides used in this study, including shRNA, siRNA, and primers.
