# Supplementary material for: TRIM21-mediated ubiquitination of PARP1 regulated by the PI3K/AKT-STAT5A axis suppresses small cell lung cancer
Source: Nat Commun. 2026 Jun 18;17:7704. doi: 10.1038/s41467-026-73271-1 (PMC13434705; doi:10.1038/s41467-026-73271-1)
Supplement: Supplementary file 3 [file 41467_2026_73271_MOESM6_ESM.pdf]

Corresponding author(s): Wenchu Lin

Last updated by author(s): Jan 30, 2026

Reporting Summary

Nature Portfolio wishes to improve the reproducibility of the work that we publish. This form provides structure for consistency and transparency in reporting. For further information on Nature Portfolio policies, see our Editorial Policies and the Editorial Policy Checklist.

Statistics

For all statistical analyses, confirm that the following items are present in the figure legend, table legend, main text, or Methods section.

n/a	Confirmed	
☐	☒	The exact sample size (n) for each experimental group/condition, given as a discrete number and unit of measurement
☐	☒	A statement on whether measurements were taken from distinct samples or whether the same sample was measured repeatedly
☐	☒	The statistical test(s) used AND whether they are one- or two-sided<br>*Only common tests should be described solely by name; describe more complex techniques in the Methods section.*
☒	☐	A description of all covariates tested
☐	☒	A description of any assumptions or corrections, such as tests of normality and adjustment for multiple comparisons
☐	☒	A full description of the statistical parameters including central tendency (e.g. means) or other basic estimates (e.g. regression coefficient) AND variation (e.g. standard deviation) or associated estimates of uncertainty (e.g. confidence intervals)
☐	☒	For null hypothesis testing, the test statistic (e.g. F, t, r) with confidence intervals, effect sizes, degrees of freedom and P value noted<br>*Give P values as exact values whenever suitable.*
☒	☐	For Bayesian analysis, information on the choice of priors and Markov chain Monte Carlo settings
☒	☐	For hierarchical and complex designs, identification of the appropriate level for tests and full reporting of outcomes
☐	☒	Estimates of effect sizes (e.g. Cohen's d, Pearson's r), indicating how they were calculated

Our web collection on statistics for biologists contains articles on many of the points above.

Software and code

Policy information about availability of computer code

Data collection	Q-Exactive HF-X mass spectrometer interfaced with an Easy nLC1200 system was used to identify proteins interacting with PARP1 at Shanghai Bioprofile Technology Co., Ltd.<br>Real-time quantitative PCR (qPCR) analysis was conducted on a QuantStudio 3 Real-Time PCR System (Thermo Fisher Scientific).<br>Images of IHC and H&E staining were captured using an Olympus IX73 inverted microscope (Olympus).<br>Fluorescent images of comet assays were acquired with a Leica DMi8 inverted fluorescent microscope (Leica).<br>Immunofluorescence (IF) and proximity ligation assay (PLA) images were obtained using a Leica STELLARIS 5 laser scanning confocal microscope (Leica).<br>Immunoblotting images were visualized using a Tanon 5200Multi chemiluminescent imaging system (Tanon).
Data analysis	GraphPad Prism v 8.0.2<br>image Pro plus v 6.0<br>Comet Assay Software Project (CASP) (Free edition)<br>Fiji (ImageJ) v1.53c<br>MaxQuant 1.6.17.0

For manuscripts utilizing custom algorithms or software that are central to the research but not yet described in published literature, software must be made available to editors and reviewers. We strongly encourage code deposition in a community repository (e.g. GitHub). See the Nature Portfolio guidelines for submitting code & software for further information.

Data

Policy information about availability of data

All manuscripts must include a data availability statement. This statement should provide the following information, where applicable:

- Accession codes, unique identifiers, or web links for publicly available datasets
- A description of any restrictions on data availability
- For clinical datasets or third party data, please ensure that the statement adheres to our policy

All the mass spectrometry proteomics data have been deposited to the ProteomeXchange Consortium (http://proteomecentral.proteomexchange.org) via the iProX partner repository with the dataset identifier PXD068168, and the potential PARP1-associated proteins were listed in Supplementary Data 1. The RNA-seq data for 1,019 cancer cell lines and the proteomic profiles of 375 cancer cell lines were retrieved from the Cancer Cell Line Encyclopedia (CCLE) via https://portals.broadinstitute.org/ccle/data. The RNA-seq datasets were downloaded from the NCBI Gene Expression Omnibus under accessions GSE40564 (https://www.ncbi.nlm.nih.gov/geo/query/acc.cgi?acc=GSE40564), GSE60052 (https://www.ncbi.nlm.nih.gov/geo/query/acc.cgi?acc=GSE60052), GSE30219 (https://www.ncbi.nlm.nih.gov/geo/query/acc.cgi?acc=GSE30219), GDS4794 (https://www.ncbi.nlm.nih.gov/geo/query/acc.cgi?acc=GDS4794), GSE149507 (https://www.ncbi.nlm.nih.gov/geo/query/acc.cgi?acc=GSE149507), and GSE40564 (https://www.ncbi.nlm.nih.gov/geo/query/acc.cgi?acc=GSE40564); obtained from the Genome Sequence Archive (http://bigd.big.ac.cn/gsa-human) under accession HRA003419 (https://ngdc.cncb.ac.cn/gsa-human/browse/HRA003419); and obtained from the European Genome-phenome Archive under accession EGAS00001000925 (https://ega-archive.org/studies/EGAS00001000925). The proteomic data were secured from the OMIX database under accession OMIX002489 (https://ngdc.cncb.ac.cn/omix/release/OMIX002489). All ChIP-seq data were sourced from the ChIP-Atlas database under accessions SRX831872 (https://chip-atlas.org/view?id=SRX831872), SRX041292 (https://chip-atlas.org/view?id=SRX041292), SRX18444036 (https://chip-atlas.org/view?id=SRX18444036), SRX9681372 (https://chip-atlas.org/view?id=SRX9681372), SRX9932045 (https://chip-atlas.org/view?id=SRX9932045), and SRX190177 (https://chip-atlas.org/view?id=SRX190177). The information on compounds and antibodies is provided in Supplementary Table 1. All the sequences of oligos used in this study are presented in Supplementary Table 2. The original photographs of immunoblots are provided in the source data file. The remaining data are available within the Article, Supplementary Information, or Source Data file. Source data are provided with this paper.

Research involving human participants, their data, or biological material

Policy information about studies with human participants or human data. See also policy information about sex, gender (identity/presentation), and sexual orientation and race, ethnicity and racism.

Reporting on sex and gender	This study did not apply to just one gender, and we did not take gender into account in our study.
Reporting on race, ethnicity, or other socially relevant groupings	This study did not involve socially relevant categorical variable.
Population characteristics	The SCLC PDX models were established using transbronchial lung biopsy tissues collected from 13 SCLC patients. Age range was 55-69 years. 11male, 2 female.
Recruitment	Written informed consent was obtained from all patients (ethical approval nos. K19-159 and K21-019).
Ethics oversight	All protocols approved by the Medical Ethics Committee of Shanghai Pulmonary Hospital, Tongji University Medical School Cancer Institute.

Note that full information on the approval of the study protocol must also be provided in the manuscript.

Field-specific reporting

Please select the one below that is the best fit for your research. If you are not sure, read the appropriate sections before making your selection.

☒ Life sciences ☐ Behavioural & social sciences ☐ Ecological, evolutionary & environmental sciences

For a reference copy of the document with all sections, see nature.com/documents/nr-reporting-summary-flat.pdf

Life sciences study design

All studies must disclose on these points even when the disclosure is negative.

Sample size	Sample size was determined based on previous studies in the field to generate reproducible results. In animal experiments, at least six animal samples were used in one group, the exact number of mice was indicated in the figure legends.
Data exclusions	No data was excluded from the study.
Replication	All experiments, including IP, WB, PT-qPCR, IF, PLA, CTG, colony formation, comet assays, ChIP-PCR, luciferase reporter assays, and drug treatments, were performed with at least 3 biological replicates. All animal experiments had at least six replicates. These replicate numbers were indicated in the figures and figure legends. All attempts at replication were successful.
Randomization	Randomization for sample and organism allocation is not applicable in this study because all experiments were performed with identifiable groups based on experimental conditions (e.g., WT vs. shTRIM21 SCLC cell lines). Within both control and experimental groups, samples were randomly assigned. For comet assays, IHC and IF signal quantifications in different areas, the regions to be analyzed were chosen randomly.
Blinding	The experiments were not performed blindly due to the researchers' need to verify the genotype of the cell lines (e.g., WT vs. shTRIM21 SCLC

cell lines). However, all comparisons were conducted automatically using statistical software, which is not influenced by the investigator.

Reporting for specific materials, systems and methods

We require information from authors about some types of materials, experimental systems and methods used in many studies. Here, indicate whether each material, system or method listed is relevant to your study. If you are not sure if a list item applies to your research, read the appropriate section before selecting a response.

Materials & experimental systems

n/a	Involved in the study
☐	☒ Antibodies
☐	☒ Eukaryotic cell lines
☒	☐ Palaeontology and archaeology
☐	☒ Animals and other organisms
☒	☐ Clinical data
☒	☐ Dual use research of concern
☒	☐ Plants

Methods

n/a	Involved in the study
☒	☐ ChIP-seq
☒	☐ Flow cytometry
☒	☐ MRI-based neuroimaging

Antibodies

Antibodies used

Antibody for Immunoprecipitation
Anti-PARP1 (Active Motif, Cat# 39559, RRID: AB_2793257, 2 µg for IP)
Anti-FLAG-Tag (Cell Signaling, Cat# 14793S, RRID: AB_2572291, 2 µg for IP)
Anti-GFP-Tag (Abcam, Cat# ab290; RRID: AB_567807, 2 µg for IP)
Goat Anti-Rabbit IgG (GeneTex, Cat# GTX26702, RRID: AB_378876, 2 µg for IP)

Antibody for Immunofluorescence and PLA
Anti-PARP1 (GeneTex, Cat# GTX632388, RRID: AB_2888311, 1:200 for IF, 1:200 for PLA)
Anti-TRIM21 (Cell Signaling, Cat#: 92043, RRID: AB_2800177, 1:200 for PLA)
Anti-TRIM21 (Abcam, Cat#: ab4369, RRID: AB_304444, 1:200 for IF)
Anti-Phospho-Histone H2A.X (Cell Signaling, Cat# 2577S, RRID: AB_2118010, 1:300 for IF)

Antibody for Immunohistochemistry
Anti-PARP1 (Active Motif, Cat# 39559, RRID: AB_2793257, 1:200)
Anti-TRIM21 (Cell Signaling, Cat# 92043, RRID: AB_2800177, 1:200)
Anti-p-AKT (Ser473) (Cell Signaling, Cat# 4060, RRID: AB_330715, 1:200)
Anti-p-STAT5A (Tyr694) (Abcam, Cat# ab106095, RRID: AB_1773752, 1:200)
Anti-Phospho-Histone H2A.X (Cell Signaling, Cat# 80312, RRID: AB_2799949, 1:200)
Anti-Ki67 (Cell Signaling, Cat# 12202S, RRID: AB_2620142, 1:200)

Antibody for ChIP
Anti-STAT5A (Abcam, Cat# ab32043, RRID: AB_1704186, 2µg for ChIP)
Anti-STAT5B (Abcam, Cat#: ab178941; RRID:AB_2885102, 2 µg for ChIP)

Primary Antibody for Western Blot
Anti-PARP1 (Active Motif, Cat# 39559, RRID: AB_2793257, 1:2000)
Anti-TRIM21 ((Abcam, Cat#: ab207728, RRID: AB_2927717, 1:1000)
Anti-AKT (Cell Signaling, Cat# 9272, RRID: AB_2084695, 1:1000)
Anti-p-AKT (Ser473) (Cell Signaling, Cat# 4060, RRID: AB_330715, 1:1000)
Anti-STAT5A (Abcam, Cat# ab32043, RRID: AB_1704186, 1:1000)
Anti-p-STAT5A (Tyr694) (Abcam, Cat# ab106095, RRID: AB_1773752, 1:1000)
Anti-GFP-Tag (Abcam, Cat# ab290, RRID: AB_567807, 1:1000)
Anti-Myc-Tag (Cell Signaling, Cat# 2276S, RRID: AB_331783, 1:1000)
Anti-GST-Tag (Affinity, Cat# T0007, RRID: AB_2839414, 1:1000)
Anti-β-Actin (Affinity, Cat# T0022, RRID: AB_2839417, 1:2000)
Anti-HA-Tag (Affinity, Cat# T0008, RRID: AB_2839415, 1:1000)
Anti-His-Tag (Affinity, Cat# T0009, RRID: AB_2839416, 1:1000)
Anti-Ubiquitin (Cell Signaling, Cat# 3936S, RRID: AB_331292, 1:1000)
Anti-RPA32 (Cell Signaling, Cat# 35869S, RRID: AB_2799086, 1:1000)
Anti-p-RPA32 (Cell Signaling, Cat# 83745S, RRID: AB_2800029, 1:1000)
Anti-ChK1 (Cell Signaling, Cat# 2360S, RRID: AB_2080320, 1:1000)
Anti-p-ChK1 (Cell Signaling, Cat# 12302S, RRID: AB_2783865, 1:1000)
Anti-STAT5B (Abcam, Cat#: ab178941; RRID:AB_2885102, 1:1000)

Secondary Antibodies
Goat Anti-Mouse IgG H&L (Alexa Fluor® 647) (Abcam, Cat#: ab150115, RRID:AB_2687948, 1:1000 for IF)
Goat Anti-Rabbit IgG H&L (Alexa Fluor® 488) (Abcam, Cat# ab150077, RRID: AB_2630356, 1:1000 for IF)
Goat Anti-Mouse IgG (H+L) (peroxidase/HRP conjugated) (Elabscience, Cat# E-AB-1001, RRID: AB_558691, 1:3000 for WB)
Goat Anti-Rabbit IgG (H+L) (peroxidase/HRP conjugated) (Elabscience, Cat#: E-AB-1003, RRID: AB_2921220, 1:3000 for WB)
IPKine™ HRP, Goat Anti-Mouse IgG LCS (Abbkine, Cat#: A25012, RRID:AB_2737290, 1:3000 for WB)
IPKine™ HRP, Mouse Anti-Rabbit IgG LCS (Abbkine, Cat# A25022, RRID: AB_2893334, 1:3000 for WB)

Validation

Antibody for Immunoprecipitation
Anti-PARP1:
https://www.activemotif.com.cn/antibodies/chromatin-immunoprecipitation-antibodies/poly-adp-ribose-polymerase-1-antibody-39559
Anti-FLAG-Tag:
https://www.cellsignal.cn/products/primary-antibodies/d-flag-antibody/14793
Anti-GFP-Tag:
https://www.abcam.cn/gfp-antibody-polyclonal-ab290.html
Goat Anti-Rabbit IgG:
https://www.genetex.com/Goat-Anti-Rabbit-IgG/p/GTX26702

Antibody for Immunofluorescence and PLA
Anti-PARP1:
https://www.genetex.com/PARP1-antibody/p/GTX632388
Anti-TRIM21:
https://www.cellsignal.cn/products/primary-antibodies/trim21-d1o1d-rabbit-monoclonal-antibody/92043
https://www.abcam.com/en-us/products/primary-antibodies/trim21-ss-a-antibody-ab4369

Anti-Phospho-Histone H2A.X:
https://www.cellsignal.cn/products/primary-antibodies/phospho-histone-h2a-x-ser139-antibody/2577

Antibody for Immunohistochemistry
Anti-PARP1:
https://www.activemotif.com.cn/antibodies/chromatin-immunoprecipitation-antibodies/poly-adp-ribose-polymerase-1-antibody-39559
Anti-TRIM21:
https://www.cellsignal.cn/products/primary-antibodies/trim21-antibody/92043
Anti-p-AKT (Ser473):
https://www.cellsignal.cn/products/primary-antibodies/phospho-akt-ser473-antibody/4060
Anti-p-STAT5A (Tyr694):
https://www.abcam.cn/anti-phospho-stat5a-tyr694-antibody-ab106095.html
Anti-Phospho-Histone H2A.X: https://www.cellsignal.cn/products/primary-antibodies/phospho-histone-h2a-x-ser139-antibody/80312
Anti-Ki67:
https://www.cellsignal.cn/products/primary-antibodies/ki-67-antibody/12202

Antibody for ChIP
Anti-STAT5A:
https://www.abcam.cn/anti-stat5a-antibody-chip-grade-ab32043.html
Anti-STAT5B:
https://www.abcam.com/en-us/products/primary-antibodies/stat5b-antibody-epr16671-ab178941

Primary Antibody for Western Blot
Anti-PARP1:
https://www.activemotif.com.cn/antibodies/chromatin-immunoprecipitation-antibodies/poly-adp-ribose-polymerase-1-antibody-39559
Anti-TRIM21:
https://www.abcam.com/en-us/products/primary-antibodies/trim21-ss-a-antibody-epr20290-ab207728
Anti-AKT:
https://www.cellsignal.cn/products/primary-antibodies/akt-antibody/9272
Anti-p-AKT (Ser473):
https://www.cellsignal.cn/products/primary-antibodies/phospho-akt-ser473-antibody/4060
Anti-STAT5A:
https://www.abcam.cn/anti-stat5a-antibody-chip-grade-ab32043.html
Anti-p-STAT5A (Tyr694):
https://www.abcam.cn/anti-phospho-stat5a-tyr694-antibody-ab106095.html
Anti-GFP-Tag:
https://www.abcam.cn/gfp-antibody-polyclonal-ab290.html
Anti-Myc-Tag:
https://www.cellsignal.cn/products/primary-antibodies/myc-antibody/2276
Anti-GST-Tag:

https://www.affinibody.com/GST-Tag-Antibody/index.html
Anti-β-Actin:
https://www.affinibody.com/Beta-Actin-Antibody/index.html
Anti-HA-Tag:
https://www.affinibody.com/HA-Tag-Antibody/index.html
Anti-His-Tag:
https://www.affinibody.com/His-Tag-Antibody/index.html
Anti-Ubiquitin:
https://www.cellsignal.cn/products/primary-antibodies/ubiquitin-antibody/3936
Anti-RPA32 :
https://www.cellsignal.cn/products/primary-antibodies/rpa32-antibody/35869
Anti-p-RPA32 :
https://www.cellsignal.cn/products/primary-antibodies/phospho-rpa32-phospho-32-residue-antibody/83745
Anti-ChK1:
https://www.cellsignal.cn/products/primary-antibodies/chk1-antibody/2360
Anti-p-ChK1:
https://www.cellsignal.cn/products/primary-antibodies/phospho-chk1-ser345-antibody/12302
Anti-STAT5B:
https://www.abcam.com/en-us/products/primary-antibodies/stat5b-antibody-epr16671-ab178941

Secondary Antibodies
Goat Anti-Mouse IgG H&L (Alexa Fluor® 647):
https://www.abcam.cn/goat-anti-mouse-igg-h-l-alexa-fluor-647-ab150115.html
Goat Anti-Rabbit IgG H&L (Alexa Fluor® 488):
https://www.abcam.cn/goat-anti-rabbit-igg-h-l-alexa-fluor-488-ab150077.html
Goat Anti-Rabbit IgG (H+L) (peroxidase/HRP conjugated):
https://www.elabscience.cn/products/10128-1-AP
Goat Anti-Mouse IgG (H+L) (peroxidase/HRP conjugated):
https://www.elabscience.cn/products/10170-1-AP
IPKine™ HRP, Mouse Anti-Rabbit IgG LCS :
https://www.abbkine.com/product/ipkine-hrp-mouse-anti-rabbit-igg-lcs-a25022/
IPKine™ HRP, Goat Anti-Mouse IgG LCS
https://www.abbkine.com/product/ipkine-hrp-goat-anti-mouse-igg-lcs-a25012/

Eukaryotic cell lines

Policy information about cell lines and Sex and Gender in Research

Cell line source(s)	The SCLC cell lines DMS273 and SHP77 were used as described previously. The SCLC cell lines H196, H526, and DMS114 were kindly provided by Dr. Hongbin Ji (Key Laboratory of Multi-Cell Systems, Shanghai Institute of Biochemistry and Cell Biology, Center for Excellence in Molecular Cell Science, Chinese Academy of Sciences). HeLa and HEK293T cells were generously provided by Dr. Ge Shan (University of Science and Technology of China). All other cell lines, including A549, PC9, AGS, and MKN45, were obtained from and maintained in our laboratory repository.
Authentication	All cell lines were authenticated by short-tandem-repeat (STR) profiling.
Mycoplasma contamination	All cell lines were regularly tested for mycoplasma contamination using a mycoplasma detection kit (Beyotime, #C0301S) .
Commonly misidentified lines (See ICLAC register)	No commonly misidentified line was used in this study.

Animals and other research organisms

Policy information about studies involving animals; ARRIVE guidelines recommended for reporting animal research, and Sex and Gender in Research

Laboratory animals	For the establishment of SCLC cell line xenografts, female nu/nu athymic mice (BALB/c background), aged 4–6 weeks, were purchased from the Guangdong Medical Laboratory Animal Center (GDMLAC) (Guangzhou, China).<br>For the establishment of SCLC PDX models, male C.B-17 severe combined immunodeficiency (SCID) mice, aged 4–6 weeks, were obtained from Shanghai SLAC Laboratory Animal Co., Ltd. (SLAC Co.) (Shanghai, China).<br>All mice were kept in an enriched environment under the standard conditions (23–25 °C temperature, 40–60% humidity) with a 12-hour light/dark cycle (lights on from 08:00 to 20:00) at the Specific-Pathogen-Free (SPF) facility. All animal protocols were approved by the Institutional Animal Care and Use Committee of Longgang District People's Hospital of Shenzhen.
Wild animals	This study did not use wild animals.
Reporting on sex	No sex- and gender-based analyses have been performed.<br>Our findings presumably apply to both genders, because there is no biological regulation known to be related to sex in this study, except for the convenience of making mice model.
Field-collected samples	This study does not involve field-collected samples.

Ethics oversight	All animal protocols were approved by the Institutional Animal Care and Use Committee of Longgang District People's Hospital of Shenzhen (2024003DW).

Note that full information on the approval of the study protocol must also be provided in the manuscript.

Plants

Seed stocks	N.A.
Novel plant genotypes	N.A.
Authentication	N.A.
