# Supplementary material for: TRIM21-mediated ubiquitination of PARP1 regulated by the PI3K/AKT-STAT5A axis suppresses small cell lung cancer
Source: Nat Commun. 2026 Jun 18;17:7704. doi: 10.1038/s41467-026-73271-1 (PMC13434705; doi:10.1038/s41467-026-73271-1)
Supplement: Supplementary file 4 [file 41467_2026_73271_MOESM7_ESM.pdf]

nature portfolio

Peer Review File

TRIM21-mediated ubiquitination of PARP1 regulated by the PI3K/AKT-STAT5A axis suppresses small cell lung cancer

Corresponding Author: Dr Wenchu Lin

This file contains all reviewer reports in order by version, followed by all author rebuttals in order by version.

Version 0:

Reviewer comments:

Reviewer #1

(Remarks to the Author)
In this manuscript, Cao et. al. report that TRIM21 as an E3 ubiquitin ligase promotes PARP1 degradation through polyubiquitination, with its downregulation in SCLC contributing to tumorigenesis. The PI3K/AKT signaling suppresses TRIM21 expression via STAT5A, stabilizing PARP1 and enhancing DNA repair. Therefore, combining PI3K/AKT and PARP inhibitors shows synergistic antitumor effects, highlighting the therapeutic potential of targeting the “PI3K/AKT-STAT5A-TRIM21-PARP1” axis in SCLC. The study presents a comprehensive investigation into the "PI3K/AKT-STAT5A-TRIM21-PARP1" axis in SCLC. However, there are concerns regarding certain aspects of the innovation, experimental design, logical flow of the manuscript, and result analysis, which could benefit from further clarification and refinement.

Major concerns:

1. A bunch of E3 ligases have been reported to regulate the degradation of PARP1. Are the functions of those E3 ligases redundant?

2. The role of TRIM21 in cancer, innate immunity, autoimmunity, and systemic lupus erythematosus is well-studied. The role of PARP1 has also been explored in tumors. The manuscript discusses a treatment strategy to enhance the efficacy of PARP inhibitors via TRIM21, which has already been introduced (PMID: 37864041). Although no prior studies have examined how TRIM21 regulates PARP1 in SCLC specifically, this aspect alone does not significantly enhance its innovation.

3. Missing details of the In Vivo Mouse Model Using shRNA/siRNA/siPARP1#1 (Figure 4E-I): the methods section (lines 723-735) lacks sufficient details. Theoretically, silencing target proteins in vivo, particularly in SCLC cells which are epithelia cells, is extremely challenging. Even if successful, the tumor microenvironment involves multiple cell types, raising concerns about whether the silencing occurs exclusively in SCLC cells. For instance, the target proteins in macrophages or dendritic cells could also been silenced, potentially influencing the results. Additionally, how were these sh/siRNAs delivered to the mice? Were they administered alone, or in combination with chemicals or viruses? Furthermore, these molecules were injected intratumorally. How was their efficacy validated? Were the target proteins significantly silenced?

4. In Vivo Model with shRNA, Empty Vector, and TRIM21 Overexpression (Figure 3K-N): this model requires further clarification in the results, figure legend, and methods (lines 728-731). Specifically, were the mice injected with wild-type cells and later treated with shRNA, empty vector, and TRIM21 overexpression intratumorally, or were pre-transfected cells used? Both approaches require in vivo evidence to demonstrate the silencing and overexpression efficiency. If the first approach was used, similar concerns about target specificity arise, as mentioned in Major Revision #2.

5. Clarification of the PI3K/AKT-STAT5A-TRIM21-PARP1 Axis in Title: the manuscript reports the role of the "PI3K/AKT-STAT5A-TRIM21-PARP1" axis in SCLC; however, the title, “TRIM21-mediated ubiquitination of PARP1 suppresses SCLC progression through the PI3K/AKT/STAT5A axis”, implies that TRIM21-induced ubiquitination of PARP1 activates the PI3K/AKT/STAT5A axis to suppress SCLC progression. This interpretation does not align with the data presented in the manuscript. The title should be clarified and consistency with the findings.

6. "Destabilizing PARP1" in Result 4: The result 4 title, TRIM21 inhibits SCLC cell survival by destabilizing PARP1 (line 207), could involve two aspects: reduced expression levels and decreased activation of PARP1. However, the data in this section may not fully support this claim. There is no examination of PARP1 expression levels, and cell viability alone cannot indicate PARP1 activation. Control groups with PARP1 overexpression or silencing alone are necessary to substantiate this conclusion.

7. Conflicting Results Regarding γH2AX Expression: Basal γH2AX expression is known to be high in cancers (PMID: 21586614). The manuscript concludes that TRIM21 suppresses SCLC progression (line179), yet Figure 3N shows higher γH2AX levels in the TRIM21 overexpression group compared to controls. Additionally, in Result 5, TRIM21 loss is stated to enhance the DNA damage response by restricting PARP1 degradation (line 233), Figures 5C-D show lower γH2AX expression in the shTRIM21#1 group than in controls. This interpretation may conflict with the manuscript's findings. These discrepancies need to be clarified to align the results, conclusion, and title more cohesively.

8. BMN673 and PKI-587 in Result 8: The combination treatment discussed in this section may not solely target the PI3K/AKT-STAT5A-TRIM21-PARP1 axis, as neither inhibitor is specific to PARP1 or TRIM21. Other mechanisms may contribute to the observed anti-tumor effects. Recent studies (PMID: 37925433) on TRIM21 and specific substrate-targeted therapies may offer insights for developing TRIM21-PARP1-specific treatments.

Minor concerns:

1. Provide evidence to support the effects of all shRNA, siRNA, and overexpression constructs (TRIM21 and PARP1) in both in vivo and in vitro experiments.

2. Figures 4A-B do not adequately support the conclusion in lines 212-214, as no PARP1 overexpression group was included. According to the figure legend, only TRIM21 knockdown or overexpression cells were used, not PARP1 overexpression cells.

Reviewer #2

(Remarks to the Author)
Summary
The manuscript titled “TRIM21-mediated ubiquitination of PARP1 suppresses small cell lung cancer progression through the PI3K/AKT/STAT5A axis” investigates the molecular mechanisms of PARP1 upregulation in SCLC and identifies that PARP1 as a novel substrate of E3 ubiquitin ligase TRIM21 which is regulated via the PI3K/AKT-STAT5A axis.
The authors conducted a comprehensive assessment of PARP1 as a putative ubiquitination and degradation substrate of TRIM21. In addition, they showed that loss of TRIM21 promotes SCLC tumor growth and genomic stability. The authors identified that PI3K/AKT pathway downregulates TRIM21 expression by inhibiting STAT5A transcription factor. Finally, the authors demonstrated the translational potential of the study by showing the synergistic effects of PARP inhibitor and PI3K/AKT inhibitor combination in SCLC.
This work presents a novel and intricate mechanism of PARP1 upregulation in SCLC, offering valuable insights into the mechanisms of SCLC tumor progression and potential therapeutic targets. While the concept of synergistic effects from the combination of a PI3K/AKT inhibitor with a PARP inhibitor has been previously reported (Cardnell et al., doi:10.1371/journal.pone.0152584), this study provides additional understanding of why this combination is effective in the context of SCLC.

Per Figure summary below:
Fig 1 PARP1-TRIM21 interaction assays. IP, co-IP, PLA. Structure function analysis. PP domain of TRIM21 required for PARP1 interaction. HD domain of PARP1 required for TRIM21 interaction his His pull-down assays. DMS273, SHP77 SCLC cell lines. And HEK 293T used in various combinations for the above assays. 293 used only for the domain domain interaction analyses.
Fig 2 – CCLE proteomics correlation and westerns of cell-lines for TRIM21 and PARP1, in sum negative correlation between TRIM21 and PARP1 protein levels. siTRIM21 in 293, SHP77 and DMS273 cells led to increased PARP1 protein levels. Cycloheximide chase shoed TRIM21 affects PARP1 protein half-life in DMS273 and SHP77 cells. HA-Ub K48R mutant showed less PARP1-Ub. IN vitro Ub assays showing Ub-PARP1 with 293 cells.
Fig 3- TRIM21 mRNA lower in SCLC cell lines and tumors vs normal. shTRIM21 increased DMS273 and SHP77 clonogenicity whereas TRIM21-OE decreased clonogenicity. Similar in xenograft analysis of DMS273 by shTRIM21 and TRIM21-OE
Fig 4 – Partial rescue with PARP1-OE in DMS273 and SHP77 cells after TRIM21-OE. PARP1-K654R increased clonogenicity in DMS273 and SHP77. In in vivo experiments with shTRIM21 and shPARP1. Adding siPARP1 reverses shTRIM21 alone protumorgenic effects.
Fig 5 – Less DNA damage in TRIM21 knockdown, less gH2AX and comet tails, and increased viability in DMS273 cells. Some assays with SHP77 as well.
Fig 6- PI3K/AKT pathway correlation analysis with TRIM21 mRNA. PI3Ki increased TRIM21 mRNA. AKT-OE inhibited TRIM21 exp and increased PARP1 levels.
Fig 7 – Transcriptional factor analysis identified 6 candidate transcription factors that bind to TRIM21 promoter, correlation analysis surfaced STAT5A, TCF21 and SIX5. STAT5A knockdown reduced TRIM21 mRNA but not PARP1 mRNA.

PI3K/AKT inhibition enhanced STAT5A binding to TRIM21 promoter.
Fig 8 – In vitro and in vivo analysis of PARPi (talazoparib) and PI3K inhibition. SHP77 and DMS273 clonogenic drug experiments with PI3Ki and PARPi. DMS273 xenografts same treatments.

The following Major Revisions are recommended:
1. Additional experiments using SCLC cell lines of POU2F3 (SCLC-P) and YAP/Inflamed (SCLC/Y/I) subtypes are recommended to prove the effects of TRIM21 are universal across SCLC subtypes. Experiments such as knocking down TRIM21 or overexpressing WT/mutant TRIM21 following examination of cell viability, clonogenicity, and genome stability should be done in cell lines of SCLC-P/Y/I subtypes.
2. The authors should replicate the cycloheximide chase assay (figure 2F) to include the level of TRIM21 over time.
3. DMS273 and SHP-77 SCLC cell lines are moderately sensitive to PARPi monotherapy. Additional experiments using PARPi-resistant cell lines to examine the efficacy of PI3K and PARPi combination is needed.
4. Talazoparib is a potent PARP trapper and enzymatic inhibitor of PARP function. Is the synergistic effect with PI3K inhibition related to PARP trapping vs the enzymatic inhibition? Veliparib as an enzymatic inhibitor with minimal PARP trapping could be an approach to deconvolute this. (Fig 8)
5. Additional SCLC molecular subtypes for PI3Ki and PARPi in vitro synergy analyses would be valuable (Fig 8)
6. Additional in vivo model(s) would be valuable and confirm translational impact, esp in other molecular subtypes or with any available patient-derived xenograft models. (Fig 8)

The following minor revisions are recommended:
1. Quantification of PLA data in figure 1F
2. Could the authors confirm PARP is proteasomal degraded via proteasomal inhibitor (MG132) experiments? Related to Fig 2F/G, etc.
3. For figure 2E, please explain why shTRIM21 #1 barely caused any PARP1 accumulation compared to shTRIM21 #2 despite similar TRIM21 knockdown efficiency.
2. An additional experiment can be done to examine global PARylation level changes in TRIM21 knockdown/overexpression cells.
3. I am not sure if interpretation of figure S2H is correct. The claim that 'TRIM21 promotes PARP1 ubiquitination under oxidative stress' is not shown properly by the data. The data is rather suggesting that oxidative stress decreases TRIM21-mediated PARP1 ubiquitination. To prove the above claim, additional controls are needed whereby cells are treated with H_2O_2 or EP without overexpression of EGFP/TRIM21. Additionally, H_2O_2 or EP treatment increases PARP1 level compared to DMSO treatment (figure S2I) rather than a relative decrease.
4. Throughout the article, the authors mentioned that the PI3K/AKT-STAT5A-TRIM21-PARP1 signaling axis promotes 'tumorigenesis' of SCLC (see line 350 as an example). I would change the wording to 'tumor growth'. Tumorigenesis refers to when a normal cell become cancerous, which is not shown by this study.
5. Consider doing a correlation analysis of TRIM21 expression and chemotherapy or PARPi response in SCLC cell lines. How well can TRIM21 expression predict treatment response?

Reviewer #3

(Remarks to the Author)
I co-reviewed this manuscript with one of the reviewers who provided the listed reports. This is part of the Nature Communications initiative to facilitate training in peer review and to provide appropriate recognition for Early Career Researchers who co-review manuscripts.

Reviewer #4

(Remarks to the Author)
The paper by Guozhen Cao and colleagues entitled "TRIM21-mediated ubiquitination of PARP1 suppresses small cell lung cancer (SCLC) progression through the PI3K/AKT/STAT5A axis" explores the connection between STAT5A repressing directly the TRIM21 locus, via PI3K-AKT-mTOR signalling. The authors show mainly with cell line work that STAT5A binding to the promoter of TRIM21 promotes PARP1 stabilization through downregulation of TRIM21 via STAT5A transcriptional repression action. The paper overall is focused on SCLC, but several angles used to demonstrate mechanistic questions, e.g. questionable model cell systems for this cancer, no genetic appropriate controls, or shortcomings in the mechanism of STAT5A or STAT5B or both acting repressive or why a sole repressor function of STAT5A downstream of the PI3K-AKT-mTOR pathway should exist leave space for improvement or study design work flow?!
It was e.g. described in the literature that STAT5 interacts with the p85 subunit of PI3K and it can transcribe AKT isoforms, as well as it is phosphorylated by mTOR, ignored by authors.
Here, authors describe a negative action via the PI3K-AKT-mTOR pathway using STAT5A only to repress TRIM21, all data presented are a bit selectively due to different model character. One can view the study design to particularly build a case in a neuroendocrine progenitor-driven tumor, that represents a bit more then 10% of all lung cancers. However, as the story is presented, literature incorporated, or mechanism explored, this paper leaves too many questions and holes and it is immature in its current format for publication in Nature Communications. Main points with multiple subpoints emphasis this as follows:

1) The team used a limited and selected set of cell lines for their study that was not appropriately introduced for cancer genome landscape information what are the key drivers in these cell lines and their origin. A closer look reveals that only

one cell line represents the study focus SCLC and five of six are quite different, leaving a selected presentation of data, not explained why these different cancer cell origins should resemble a conclusive set of patient derived model cell systems. No primary lung cancer purified cell samples were extracted to back up data or the lack of PDX or alternatively organoid models of SCLC which exist are further limitations. The selected cell lines used display even mutations affecting the axis to be described distinctly, not introduced or discussed. Thus, the selection and representation of work related to these models can be regarded as inappropriate. Conclusions drawn can lead to false interpretations of results. The authors need to validate in a genetic model of SCLC their key findings and PDX models exist or other relevant samples such as primary specimens, which were all not incorporated, representing a problematic model work. Why the representation of the cell lines were done and why they were not introduced and described is far from optimal and too selected. The authors need to do a better work to demonstrate that their models represent a valid genetic model of SCLC, e.g. establishing paired model cell line systems of human origin. Here, also CRISPR-cas9 paired cell line deletion or after deletion add back was not performed to e.g. assemble three cell lines of one small cell lung cancer patient. Instead, authors did use knockdown, but omitted add back of the gene of interest to control for off-target effects of any sh- or si-RNA interference work. All that leaves room for improvements. A small survey on the cell lines reveals the following:
From the six tumour cell lines used for the study DMS273 which is also called DMS 273 and was isolated in 1978 from a patient with SCLC, is the closest model. However, it was isolated via the pleural fluid specimen from a 50-year-old female patient decades ago and it can drift away from the study subject tumour. The patient was in relapse after chemotherapy and radiotherapy, where cells were reported in Cellosaurs to carry a TP53 and NPM1 mutation, which is relevant here, e.g. both interact with STAT proteins and NPM1 steers import and export into nucleus of STATs hanging together with BRCA1/2/BARD complex at the nuclear pore.

PC9 which is also called in Cellosaurus PC-9 represents a human adenocarcinoma from lung tissue which remains differentiated, but it is not a SCLC model. It carryies TP53 mutation and two EGFR mutations, the later phosphorylates STAT5 at multiple alternative tyrosine phosphorylation sites, of unknown biological consequence, or pYSTAT1/3 as well......

SHP77 which is also called SHP-77 are epithelial cells are derived from a non-encapsulated primary lung tumor from the apical portion of the upper lobe of the left lung of a 54-year-old, again not SCLC model. It was isolated from a Caucasian male in 1977. Cellosaurus documents KRAS, TP53, RAC1 and ABL1 mutations. Again, ABL-mediated tyrosine phosphorylation and nuclear import of STAT5 complexing with RAC1 for nuclear import is affected as well as mitochondrial function of STAT3/5 via KRAS, all not discussed or introduced, etc.

A549 cells are adenocarcinoma human alveolar basal epithelial cells, again not SCLC as a model and they constitute a cell line developed in 1972 through the removal and culturing of cancerous lung tissue in the explanted tumor of a 58-year-old Caucasian male. Cellosaurus documents KRAS, TP53 and STK11 mutations. See comments above and also below to cellular mutations.

AGS is a cell line exhibiting epithelial morphology that was isolated in 1979 from the stomach tissue of a 54-year-old, White, female patient with gastric adenocarcinoma. Cellosaurus documents KRAS, TP53, CTNNB1 and two PI3KCA mutations. It is questionable if persistent PI3K-AKT-mTOR action would be a good model or not and why should gastric cancer be relevant for studying and concluding on SCLC, also true for next cell line.

MKN45 which is also called MKN-45 cell line is a human gastric cancer cell line, derived from the poorly differentiated adenocarcinoma of the stomach. Cellosaurus documents MET amplification and TP53 mutation. MET growth factor receptor probably also phosphorylates STAT5 at alternative tyrosines similar like EGFR, all not clear.

The use of HEK293T cells for mechanistic insight is another distance to SCLC….

Thus, in conclusion the model cell systems used in this study and how authors deal with their introduction and discussion of results are in most cases far away from the tumour in focus or can be called a selected assembly. That is not acceptable to serve as a basis for claims of mechanistic nature in small cell lung cancer studies, where driver mutation status is key to be taken into consideration in multiple model cell character with similar mutation setups of SCLC origin.

2) STAT5 is encoded by two different gene products, STAT5A and STAT5B, both binding to an inverted palindromic sequence. The question if STAT5B follows the same trait and rules is important and both STAT5 proteins make a heterodimer, but STAT5B is more oncogenic. STAT5A is a balancer with shorter half life over STAT5B action, authors ignored expression or activity of STAT5B in SCLC. Both bind palindromic sequences, but STAT5B is in particular more versatile for their spacing for 2 up to 4 bps within the palindromic sequence that authors displayed in Sup. Fug. 7, but this might be a comprehensive bioinformatics way of showing potential binding sites. A reporter construct with these sites and identification of corepressor complex with NCOR or BCOR components being described to bind to STAT5 is feasible. However, if true more sequences could be expected in the large loci used for analysis, promoter or enhancer as well as exom intron structure of the TRIM21 locus have to be illustrated better. The whole transcriptional relevant elements of the TRIM21 locus need to be displayed and marked for potential binding sites of the STAT family and it might not only be STAT5 that then is relevant. E.g. in a simple sequence file with marked binding site analysis also validated by ChIP for both STAT5A or STAT5B and for control purposes also for STAT3 ChIP or STAT1 ChIP and monitoring of their mRNA and protein expression is key here. Authors should show these sites as single sites, since that allows judgment in a more meaningful manner. Interspecies conservation should be displayed e.g. is it conserved in mice, rat, canine and e.g. chimpanzee? Binding site conservation if existent in a more general manner manifests the transcriptional mode of action for different species as model system, e.g. is there a species that is more vulnerable to SCLC then how about that

conservation? These binding sequences can also be bound by BCL6, a STAT5 or STAT3 locus repressor in a YIN/YANG fashion. Here, BCL6 expression monitoring would be important. STAT5A can be expressed via TP53 action, but not STAT5B, whereas both interact with TP53. Most model cell systems are TP53 mutated making mechanistic work here in that regard unclear due to the many functions that mutated TP53 still has, genetic Null mutation for TP53 and a better separation into driver mutation clusters would be key here. Biallelic inactivation of TP53 and RB1 is nearly universal in SCLC cases and indeed, some 20 years ago also a GEMM was made with both their deletions genetically. Normally, wt TP53 can repress STAT5 mediated transcription as was shown in MPN and AML model cell systems, but mutated TP53 can boost STAT5-mediated transcription. Last, STAT3 and STAT1 action need to be controlled and taken into consideration, since they are interwoven on chromatin action or transcriptional regulation in cancer cells with STAT5 mode of action. Pharmacologic intervention with selective STAT5 inhibitors would also be appropriate and such tools exist. No genetic model of STAT5 deletion with SCLC was made as further limitation of the study backing up claims.

3) One study main claim is that the PI3K/AKT pathway negatively regulates STAT5A activation, but that is so far not reported in the literature, despite many studies on both JAK-STAT5 or PI3K-AKT-mTOR action. A known scaffold function for interaction with STAT5 and PI3K was shown to promote cytoplasmic localisation of STAT5 in cancer cells as a result of p85-STAT5 interaction, influencing e.g. nuclear shuttling. mTOR can phosphorylate STAT5A and STAT5B within the coiled coil domain at position serine 193, but the biological consequence is underexplored, which might be relevant here, phosphoproteomic data exists that should be incorporated for SCLC. STAT5 was also shown to directly boost the PI3K-AKT-mTOR pathway in breast cancer cells e.g. through transcribing AKT1 isoform. These studies were incompletely taking into consideration, particularly for the SCLC angle.

Version 1:

Reviewer comments:

Reviewer #1

(Remarks to the Author)
The authors have tried to address all my comments.

Reviewer #2

(Remarks to the Author)
Authors have been able to address much of the requested revisions.

Some key ones that were stated were the additional SCLC subtypes (P, and Y/I) which shows greater generalizability of their results.

The authors used patient-derived organoid models for part of the revisions (rather then in vivo patient-derived xenograft models) – this lessens the translational assessment, however this may be adequate at this juncture for this type of manuscript (as cell line xenografts were studied).

However, there are some minor revisions recommended to be clarified or incorporated please:

Figure 3D - Please clarify why low TRIM21 expression is associated with shorter overall survival (manuscript figure 3D), yet no significant correlation is observed between TRIM21 expression and chemotherapy or PARPi response. This seems inconsistent with the authors' claim that low TRIM21 expression leads to increased PARP1 levels and genomic stability.

Figure 4B, clonogenic assay – The images do not appear cleanly quantifiable. In particular, the second and second last wells for DMS273 are difficult to interpret. Please clarify how these were quantified. I note in methods pg 23 lines 638-639 states that "Relative cell densities were 638 analyzed using ImageJ software. The quantification of clonogenic survival was expressed as the ratio of absorbance values relative to the control group." However, absorbance would quantify the surface area covered by cells – and is NOT a quantification of clonogenic potential (i.e. 1 cell being able to form a colony --- see Fig 2A in PMID: 34697469). Could these assays In Fig 4B be re-quantified? Or methodologically infeasible, the definition of this assay as displayed in revised manuscript be then corrected?

Minor revision that could be mentioned as limitations in the results and discussion:

Figure 4A, 4B – Could alternative substrates of TRIM21 explain the observed phenotypes, rather than solely its effects on PARP1? Given that PARP1 knockdown/overexpression only partially rescues the phenotype, this possibility should be considered and discussed a bit more as a potential limitation in the Discussion section. (I note p19 lines 530 does begin to address this but a handful of add'l context sentences would be appreciated)

Reviewer #3

(Remarks to the Author)
I co-reviewed this manuscript with one of the reviewers who provided the listed reports. This is part of the Nature Communications initiative to facilitate training in peer review and to provide appropriate recognition for Early Career Researchers who co-review manuscripts.

Reviewer #5

(Remarks to the Author)
The authors have satisfactorily fulfilled the requests of the reviewer.

Open Access This Peer Review File is licensed under a Creative Commons Attribution 4.0 International License, which permits use, sharing, adaptation, distribution and reproduction in any medium or format, as long as you give appropriate credit to the original author(s) and the source, provide a link to the Creative Commons license, and indicate if changes were made.
In cases where reviewers are anonymous, credit should be given to 'Anonymous Referee' and the source.
The images or other third party material in this Peer Review File are included in the article's Creative Commons license, unless indicated otherwise in a credit line to the material. If material is not included in the article's Creative Commons license and your intended use is not permitted by statutory regulation or exceeds the permitted use, you will need to obtain permission directly from the copyright holder.
To view a copy of this license, visit https://creativecommons.org/licenses/by/4.0/

Title: "Regulation of TRIM21-mediated ubiquitination of PARP1 by the PI3K/AKT/STAT5A axis suppresses the progression of small cell lung cancer"

Tracking #: **NCOMMS-24-73313**

Authors: Cao *et al*.

Dear Editor and Reviewers,

We sincerely appreciate the time and effort you have dedicated to reviewing our manuscript, "*Regulation of TRIM21-mediated ubiquitination of PARP1 by the PI3K/AKT/STAT5A axis suppresses the progression of small cell lung cancer*" (**NCOMMS-24-73313**). Your insightful comments and constructive critiques have significantly strengthened our study, and we are deeply grateful for the opportunity to refine our work.

In this revision, we have thoroughly addressed all reviewers' concerns through extensive additional experiments, in-depth analyses, and comprehensive clarifications, with key advancements including: 1) validated *in vivo* proteins modulation approaches with improved controls and specificity analyses (Response Figure 1, 2, 5); 2) resolved γH2AX discrepancies through mechanistic studies linking TRIM21-PARP1 to DNA damage responses (Response Figure 4); 3) demonstrated similar effects across SCLC subtypes using additional cell lines and patient-derived organoid models (Response Figure 6, 10, 11); 4) incorporated PDX models to strengthen clinical relevance (Response Figure 18); 5) characterized STAT5A, but not STAT5B, specifically regulates *TRIM21* transcription (Response Figure 21, 22); 6) accounted for genetic backgrounds through nuclear localization studies (Response Figure 20). We have exhaustively responded to all reviewers' comments with point-by-point responses and rigorous revisions, including but not limited to the key points mentioned above (Response Figure 3, 7-9, 12-17, 19, 23-28).

We have incorporated the new or revised data as Figure 2E, 2F, 4A, 4B, 7E, 7I and 8A-I, and also in Supplementary Figure S2H, S3G-I, S5C-F, S7J, S7K, S8A-J, S9B, S9K, S9L, S9O, S9P, S10A-D. Due to the incorporation of new data and reorganization of the manuscript, the previous Figure 7B, 7E, 8A-C have been moved to Supplementary Figure S7A, S7E, S9E-H, S9M, S9N; the former Supplementary Figure S5C-K is now presented as Figure S5G-O; the previous Supplementary Figure S7A-G has been reassigned to Figure S7B-D and S7F-I; and the original Supplementary Figure S8A,

S8B, S8C, S8E, S8G, S8H, and S8I are now shown as Supplementary Figure S9A, S9C, S9D, S9I, S9J, S10E, and S10F, respectively. More analyses and discussions have been included in the revised manuscript, and major changes are highlighted for your convenience.

Collectively, these revisions resolve all concerns while providing novel insights into this regulatory axis in SCLC, significantly strengthening the manuscript's impact, and we believe the work now makes an important and timely contribution to understanding SCLC biology and treatment strategies.

Thank you once again for your constructive comments, which have greatly improved the quality of our work.

Point-by-Point Reply to Reviewers' Comments:

(*The original comments of reviewers are shown in italics*)

Reviewer #1 (Comments to the Author):

In this manuscript, Cao et. al. report that TRIM21 as an E3 ubiquitin ligase promotes PARP1 degradation through polyubiquitination, with its downregulation in SCLC contributing to tumorigenesis. The PI3K/AKT signaling suppresses TRIM21 expression via STAT5A, stabilizing PARP1 and enhancing DNA repair. Therefore, combining PI3K/AKT and PARP inhibitors shows synergistic antitumor effects, highlighting the therapeutic potential of targeting the "PI3K/AKT-STAT5A-TRIM21-PARP1" axis in SCLC. The study presents a comprehensive investigation into the "PI3K/AKT-STAT5A-TRIM21-PARP1" axis in SCLC. However, there are concerns regarding certain aspects of the innovation, experimental design, logical flow of the manuscript, and result analysis, which could benefit from further clarification and refinement.

Response: Thanks for your interest in our study. In response to your constructive suggestions, we have systematically addressed each of the specific concerns raised regarding innovation, experimental design, logical flow, and result analysis through comprehensive revisions and additional experiments. Our responses to every point have been carefully documented, and we believe that these revisions have successfully resolved all concerns while further strengthening both the novelty and scientific rigor of our study.

Major concerns:

1. A bunch of E3 ligases have been reported to regulate the degradation of PARP1. Are the functions of those E3 ligases redundant?

Response: We appreciate the reviewer's insightful concerns regarding potential functional redundancy among E3 ligases involved in PARP1 degradation. As noted in our introduction, multiple E3 ligases have indeed been reported to modulate PARP1 stability across various conditions and tissue contexts. In this study, we identify TRIM21 as a novel E3 ligase that promotes PARP1 degradation under physiological conditions through K48-linked polyubiquitination at residue K654, which is a site not previously associated with other known E3 ubiquitin ligases (Figure 1B, 1C, 2I, 2L, and 2M in revision).

Furthermore, unlike previously reported E3 ligases, TRIM21 mediates PARP1 degradation not only under physiological conditions but also in response to oxidative stress or DNA damage (Supplementary Figure S2H in revision). This broad regulatory capacity underscores the unique role of TRIM21 as an E3 ligase for PARP1.

The distinct ubiquitination sites and context-dependent degradation mechanisms suggest that these E3 ligases are not functionally redundant but rather act cooperatively or under specific conditions to finely regulate PARP1 stability. This diversity in regulation likely reflects the multifaceted roles of PARP1 and highlights the complexity of its biological functions. Our findings thus expand the mechanistic understanding of PARP1 regulation and emphasize the importance of investigating context-specific contributions of E3 ligases in future studies.

2. The role of TRIM21 in cancer, innate immunity, autoimmunity, and systemic lupus erythematosus is well-studied. The role of PARP1 has also been explored in tumors. The manuscript discusses a treatment strategy to enhance the efficacy of PARP inhibitors via TRIM21, which has already been introduced (PMID: 37864041). Although no prior studies have examined how TRIM21 regulates PARP1 in SCLC specifically, this aspect alone does not significantly enhance its innovation.

Response: We fully acknowledge the reviewer's point regarding the established roles of TRIM21 and PARP1 in cancer biology. We apologize for our oversight in not initially citing the important work

(PMID: 37864041), which demonstrates that TRIM21 enhances PARPi sensitivity through regulation of BRCA1 in breast cancer. We have now discussed and cited this work as reference 68 in the revised manuscript (Lines 519-522).

Beyond these previously reported functions, our study uncovers distinct and novel mechanistic insights:

1) Novel mechanistic basis for combination therapy

Prior studies have shown that co-inhibition of PI3K/AKT and PARP generates synergistic anti-tumor effects. However, the underlying mechanism is still incompletely understood. In this study, we elucidate a novel molecular mechanism whereby PI3K/AKT inhibition potentiates PARPi efficacy: PI3K/AKT inhibition restores TRIM21 expression, destabilizing PARP1 and amplifying DNA damage, thereby sensitizing cancer cells to PARPi.

2) First identification of TRIM21-mediated PARP1 ubiquitination

We reveal, for the first time, that TRIM21 directly targets PARP1 for K48-linked polyubiquitination and degradation. This mechanism is spatially and functionally distinct, as TRIM21 binds PARP1's HD domain and operates under physiological conditions.

3) Discovery of the PI3K/AKT-STAT5A-TRIM21 regulatory axis

Our work identifies STAT5A as a transcriptional regulator of TRIM21 that is suppressed by the PI3K/AKT pathway, revealing a novel regulatory axis for TRIM21 and PARP1. This upstream axis explains why TRIM21 is downregulated in SCLC, driving PARP1 stabilization and tumor progression.

These discoveries advance the field by delineating an SCLC-specific regulatory axis and offering a translatable strategy to overcome primary resistance to PARPi.

3. Missing details of the In Vivo Mouse Model Using shRNA/siRNA/siPARP1#1 (Figure 4E-I): the methods section (lines 723-735) lacks sufficient details. Theoretically, silencing target proteins in vivo, particularly in SCLC cells which are epithelia cells, is extremely challenging. Even if successful, the tumor microenvironment involves multiple cell types, raising concerns about whether the silencing occurs exclusively in SCLC cells. For instance, the target

proteins in macrophages or dendritic cells could also been silenced, potentially influencing the results. Additionally, how were these sh/siRNAs delivered to the mice? Were they administered alone, or in combination with chemicals or viruses? Furthermore, these molecules were injected intratumorally. How was their efficacy validated? Were the target proteins significantly silenced?

Response: Sorry for the confusion caused by the lack of detail and precision in this section. Below, we have provided a thorough clarification, and corresponding revisions have also been made in revision.

1) Experimental workflow

Stable DMS273 cell lines with *TRIM21* knockdown (sh*TRIM21* #1) or *PARP1* knockdown (sh*PARP1* #1) were similarly implanted. Once tumors reached approximately 100~150 mm^3, the sh*TRIM21* #1 group was randomized into two subgroups: one continued without further treatment, while the other received intratumoral injections of si*PARP1* #1 to establish the "sh*TRIM21* #1 + si*PARP1* #1" cohort. We revised the experimental workflow to explicitly illustrate this timeline and treatment regimen (Response Figure 1A, designated as Figure 4E in revision).

2) Delivery and specificity

shRNA: Stable knockdown was achieved via lentiviral transduction *in vitro* prior to implantation, ensuring tumor cell-specific silencing. siRNA: si*PARP1* #1 was delivered intratumorally using in vivo-jetPEI® (Polyplus), a non-viral transfection reagent optimized for localized uptake in tumor cells. Critically, in designing si*PARP1* #1, we specifically targeted a highly unique sequence (CCGAGAAATCTCTTACCTCAA) located exclusively within the coding DNA sequence (CDS) region of human *PARP1*. This sequence shows no homology with the murine *PARP1* CDS, thereby ensuring that our si*PARP1* #1 selectively knocks down human *PARP1* in implanted SCLC cells while leaving mouse-derived cells in the tumor microenvironment (*e.g.*, macrophages or dendritic cells) unaffected.

3) Validation of silencing efficiency

IHC analysis demonstrated a significant reduction in TRIM21 and PARP1 protein levels within tumor tissues, confirming the efficient silencing of the target proteins (Response Figure 1B, designated as Figure 4I in revision). Additionally, newly supplemented immunofluorescence (IF) analysis of

corresponding tumor tissues further validated these findings: in the sh*TRIM21* #1 group, TRIM21 was significantly decreased while PARP1 exhibited high expression; in the sh*PARP1* #1 group, PARP1 was markedly reduced with TRIM21 levels comparable to the control group; in the combined sh*TRIM21* #1 + si*PARP1* #1 group, both TRIM21 and PARP1 showed low expression, results fully consistent with the IHC data (Response Figure 1C).

4) Technical details added

The revised Methods section specifies the siRNA formulation as follows: "The intratumoral injection of siRNAs was performed using in vivo-jetPEI® according to the manufacturer's guidelines. Briefly, 10 μg siPARP1 #1 diluted in sterile 5% glucose solution was complexed with in vivo-jetPEI® at a ratio of 0.16 μL reagent per 1 μg siRNA. The formulated siRNA was administered intratumorally every other day. siRNA sequences are detailed in Supplementary Table S3." These experimental details have been incorporated into the revised manuscript with highlighted details (Lines 846-855).

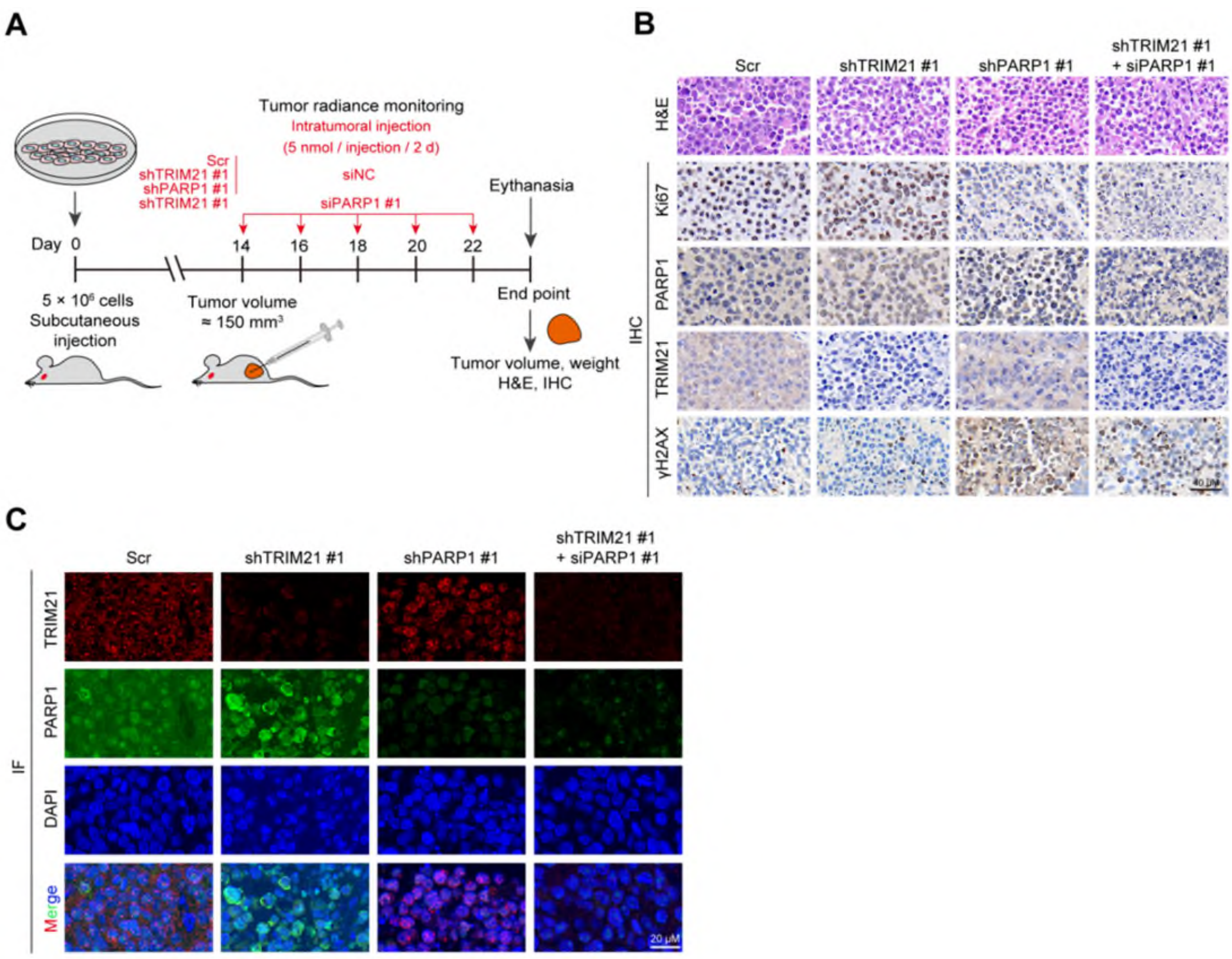

Response Figure 1

Response Figure 1. **A**) Schematic representation of the experimental workflow of the xenograft study. Colored arrows indicate the times when different events occurred. **B**) Representative images of HE staining and immunohistochemistry for Ki67, PARP1, TRIM21, and γH2AX within tumor sections. Scale bars, 40 μm. **C**) Representative immunofluorescence images of PARP1 and TRIM21 in tumor sections. Scale bars, 20 μm.

4. In Vivo Model with shRNA, Empty Vector, and TRIM21 Overexpression (Figure 3K-N): this model requires further clarification in the results, figure legend, and methods (lines 728-731). Specifically, were the mice injected with wild-type cells and later treated with shRNA, empty vector, and TRIM21 overexpression intratumorally, or were pre-transfected cells used? Both approaches require in vivo evidence to demonstrate the silencing and overexpression efficiency. If the first approach was used, similar concerns about target specificity arise, as mentioned in Major Revision #2.

Response: The reviewer's rigorous evaluation of our methodology has been most helpful to us. To clarify: in the part of the study, stable DMS273 cell lines with *TRIM21* knockdown (sh*TRIM21* #1) or overexpression (*TRIM21* OV), along with their respective controls (shControl/Scr and Empty Vector/EV), were generated *in vitro* via lentiviral transduction and validated for efficiency using Western blot (Response Figure 2A, designated as Figure 2E in revision). These pre-transfected cells were then subcutaneously implanted into nude mice to assess TRIM21's role in SCLC tumorigenicity. No post-implantation intratumoral interventions were performed. To confirm *in vivo* silencing and overexpression efficacy, we conducted IHC analysis on harvested tumors (Response Figure 2B, designated as Figure 3N in revision), which demonstrated significant TRIM21 reduction in sh*TRIM21* #1 tumors and marked overexpression in *TRIM21* OV tumors. To provide clearer documentation of our experimental process and results, we have refined and supplemented the relevant experimental methods in the Results, figure legends, and Methods sections in revision with highlighted details (Lines 213–219, 844–846, and 1261–1266).

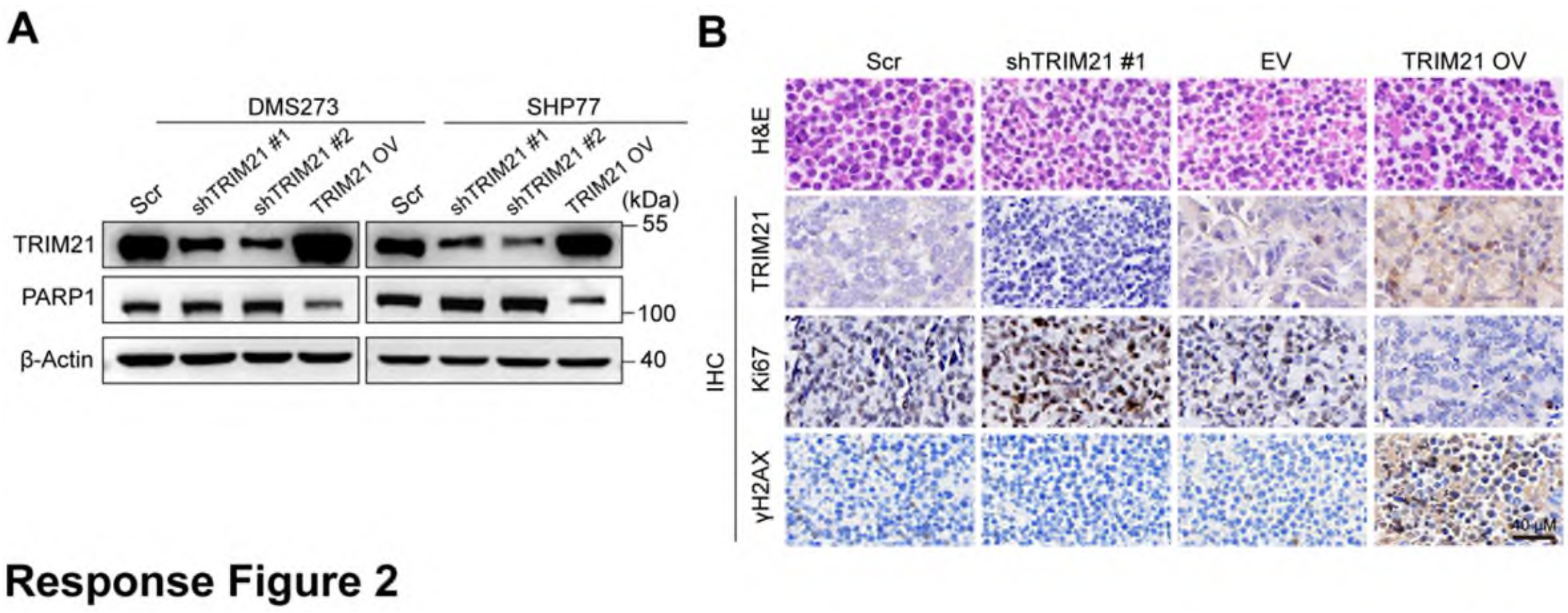

Response Figure 2. **A**) Assessment of PARP1 expression following *TRIM21* modulation. Western blot analyses were performed to assess PARP1 expression after knockdown and overexpression of *TRIM21* in DMS273 and SHP77 cells. **B**) Representative images of HE staining and immunohistochemistry for TRIM21, Ki67, and γH2AX within tumor sections. Scale bars, 40 μm.

5. Clarification of the PI3K/AKT-STAT5A-TRIM21-PARP1 Axis in Title: the manuscript reports the role of the "PI3K/AKT-STAT5A-TRIM21-PARP1" axis in SCLC; however, the title, "TRIM21-mediated ubiquitination of PARP1 suppresses SCLC progression through the PI3K/AKT/STAT5A axis", implies that TRIM21-induced ubiquitination of PARP1 activates the PI3K/AKT/STAT5A axis to suppress SCLC progression. This interpretation does not align with the data presented in the manuscript. The title should be clarified and consistency with the findings.

Response: We fully agree with the reviewer's suggestion. To accurately reflect our findings, which the PI3K/AKT/STAT5A axis regulates TRIM21-mediated PARP1 ubiquitination to suppress SCLC progression, we have revised the title to: "Regulation of TRIM21-mediated ubiquitination of PARP1 by the PI3K/AKT/STAT5A axis suppresses the progression of small cell lung cancer". This revision clarifies the mechanistic hierarchy, demonstrating that PI3K/AKT signaling regulates STAT5A activity, which in turn controls TRIM21 expression, ultimately leading to PARP1 destabilization. This interpretation aligns precisely with the mechanistic evidence presented in our study.

6. "Destabilizing PARP1" in Result 4: The result 4 title, TRIM21 inhibits SCLC cell survival by destabilizing PARP1 (line 207), could involve two aspects: reduced expression levels and decreased activation of PARP1. However, the data in this section may not fully support this claim. There is no examination of PARP1 expression levels, and cell viability alone cannot indicate PARP1 activation. Control groups with PARP1 overexpression or silencing alone are necessary to substantiate this conclusion.

Response: Thank you very much for your suggestions. As recommended, we have performed additional experiments including control groups with *PARP1* overexpression or silencing alone, while systematically examining PARP1 accumulation levels and cell viability under these genetic manipulations (Response Figure 3A, D, designated as Supplementary Figure S4A, B in revision; Response Figure 3B, C, designated as Figure 4A, B in revision). Our new results demonstrated that the promotion of SCLC cell viability induced by *TRIM21* knockdown is significantly reversed by concurrent *PARP1* silencing; conversely, the suppressive effect on SCLC cell viability caused by *TRIM21* overexpression is partially rescued by *PARP1* overexpression (Response Figure 3B-D). Throughout these experiments, PARP1 protein levels consistently exhibited corresponding changes

(*e.g.*, reduced accumulation upon *TRIM21* overexpression, increased stability upon *TRIM21* knockdown) (Response Figure 3A). These findings collectively establish that TRIM21 inhibits SCLC cell survival specifically through regulating PARP1 protein stability, thereby providing the conclusion "TRIM21 inhibits SCLC cell survival by destabilizing PARP1" in Result 4. The relevant data and revised interpretation have been incorporated into the manuscript with highlighted details (Lines 224–229 and 1271-1274).

Additionally, we further investigated the impact of *TRIM21* expression levels on PARP1 activation by assessing global PARylation and found that *TRIM21* knockdown significantly enhanced overall PARylation levels, while *TRIM21* overexpression markedly reduced PARylation (Response Figure 3E). These changes were associated with corresponding alterations in PARP1 protein abundance, which accumulated following *TRIM21* depletion and reduction after *TRIM21* overexpression. These findings establish that TRIM21 regulates global PARylation through controlling PARP1 protein stability, thereby modulating PARP1 catalytic activity.

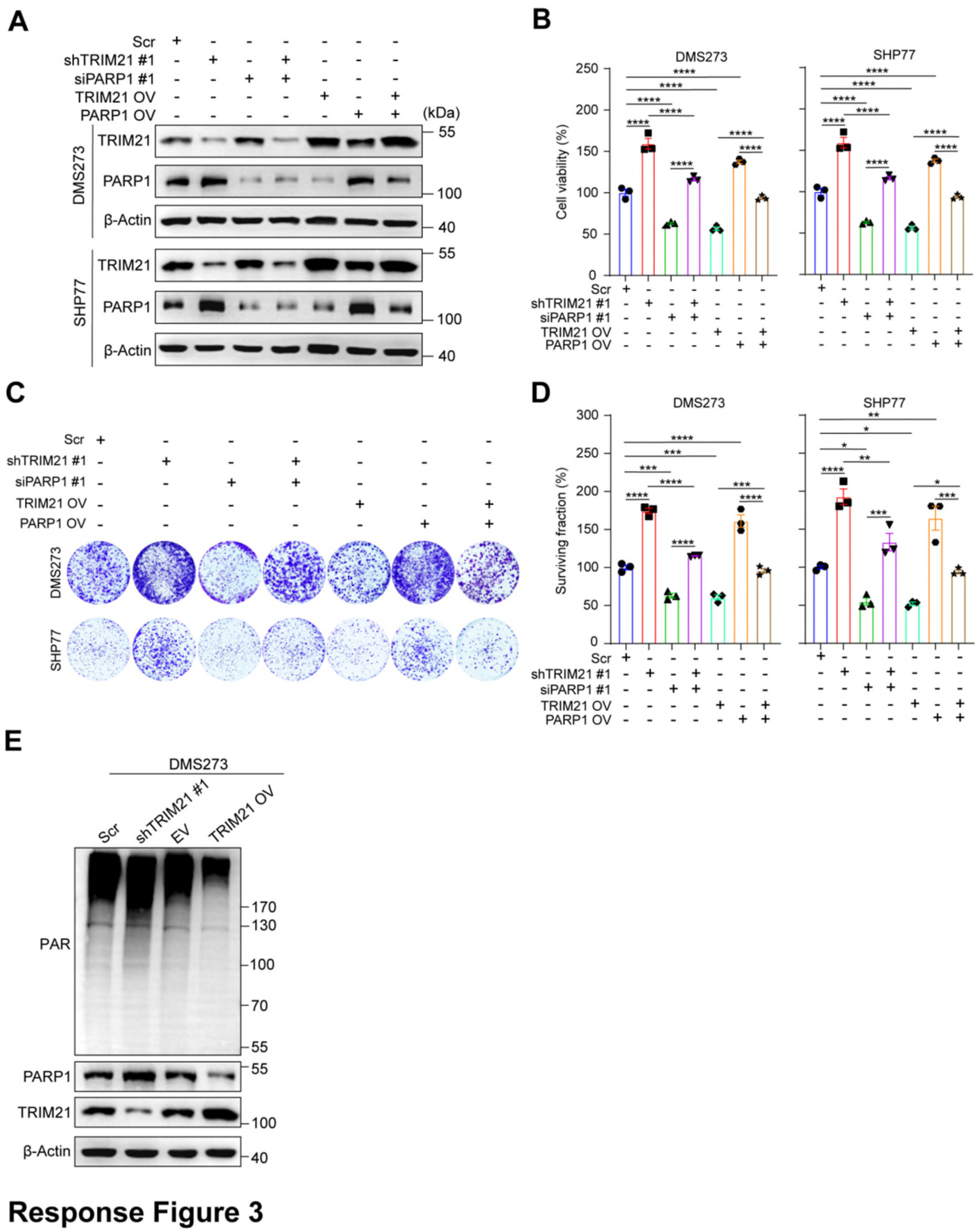

Response Figure 3. **A**) Western blot analysis investigating the compensatory effects of PARP1 on altered *TRIM21* expression. **B**) Cell viability assessment using the CellTiter-Glo luminescent assay showing the compensatory effect of PARP1 on cell survival in *TRIM21* knockdown or overexpressed cells. **C**) Colony formation assays illustrating PARP1-mediated rescue of capacity in *TRIM21*-knockdown or -overexpressing cells. **D**) Quantitative analysis of colony formation shown in (**C**). **E**) Global PARylation levels assessed by western blot using whole-cell lysates from DMS273 cells with *TRIM21* knockdown or overexpression. Data are represented as mean ± SEM, and were statistically analyzed using a one-way ANOVA. Significance is denoted as follows: *P < 0.05; **P < 0.01; ***P < 0.001; ****P < 0.0001.

7. Conflicting Results Regarding γH2AX Expression: Basal γH2AX expression is known to be high in cancers (PMID: 21586614). The manuscript concludes that TRIM21 suppresses SCLC progression (line179), yet Figure 3N shows higher γH2AX levels in the TRIM21 overexpression group compared to controls. Additionally, in Result 5, TRIM21 loss is stated to enhance the DNA damage response by restricting PARP1 degradation (line 233), Figures 5C-D show lower γH2AX expression in the shTRIM21#1 group than in controls. This interpretation may conflict with the manuscript's findings. These discrepancies need to be clarified to align the results, conclusion, and title more cohesively.

Response: We are grateful for the reviewer's insightful observation regarding γH2AX expression. As rightly noted, elevated basal γH2AX levels are a well-established feature in many cancers compared to normal tissues[1-2].

To rigorously address the relationship between TRIM21 expression and γH2AX, we performed additional detailed experiments. Our new data confirmed that *TRIM21* knockdown cells exhibit lower γH2AX levels, while *TRIM21* overexpression results in higher γH2AX expression, similar to *PARP1* depletion (Response Figure 4A-C). Importantly, these findings were further validated in tumor tissues using immunofluorescence assays (Response Figure 4D), which reproduced the same trends observed at the cellular level. These new experimental results fully align with and reinforce our original data presented in Figure 3N, 4I, 5C, 5D, and Supplementary Figure S5A-F.

It has been established that PARP1 plays a role in DNA damage repair, with PARP1 defects resulting in substantial γH2AX buildup in cells[3]. We propose that this pattern arises because TRIM21 expression directly modulates PARP1 protein levels: *TRIM21* overexpression promotes ubiquitination-mediated PARP1 degradation, and since *PARP1* deficiency impairs DNA damage repair, this leads to significant γH2AX accumulation. Conversely, *TRIM21* loss stabilizes PARP1, enhancing DNA repair capacity and reducing γH2AX. This mechanism aligns with our conclusion that TRIM21 suppresses SCLC progression by regulating PARP1-dependent DNA damage responses.

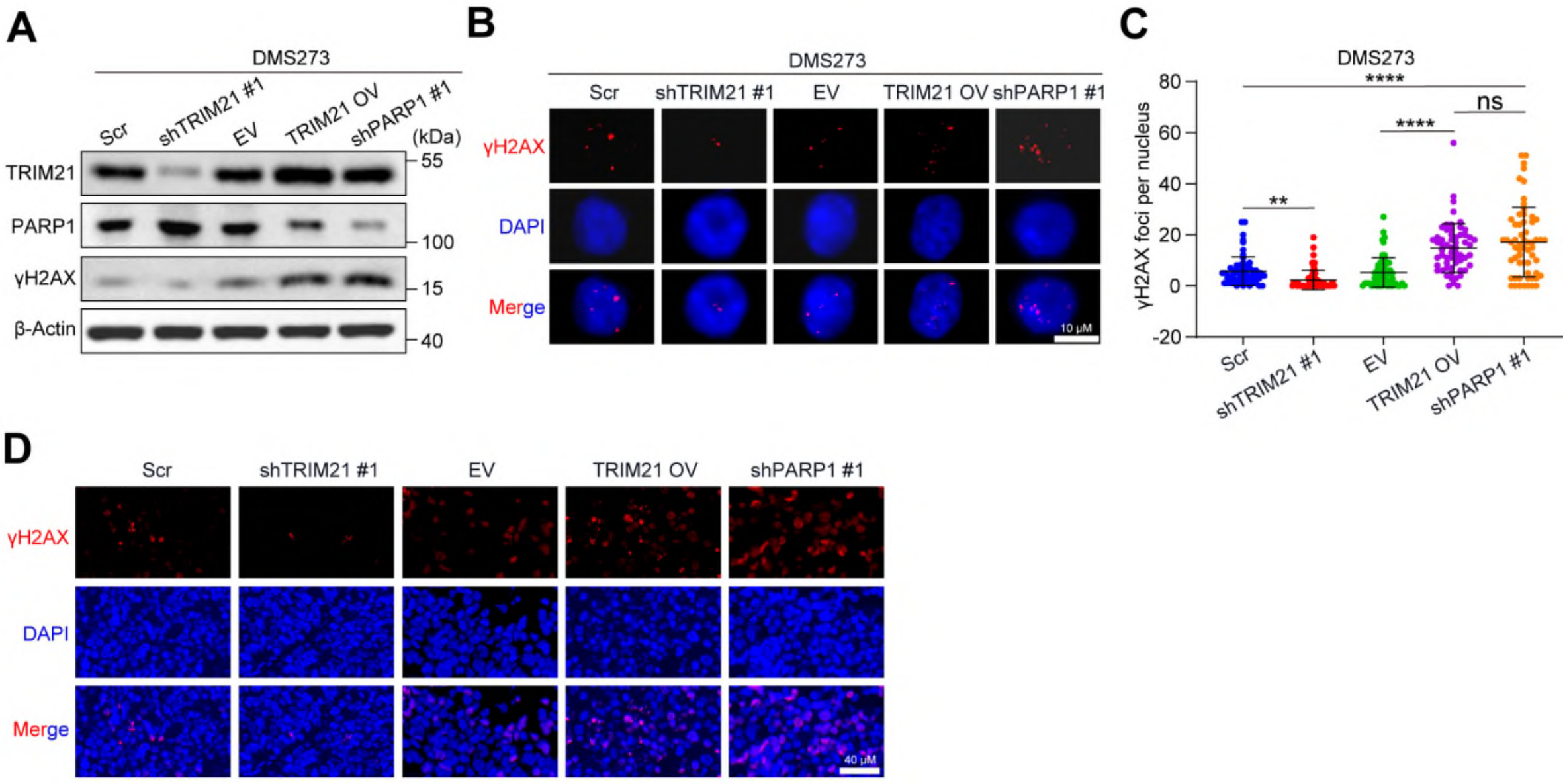

Response Figure 4

Response Figure 4. **A**) Western blot analyses showing the expression level of PARP1 and TRIM21 following *TRIM21* knockdown/overexpression or *PARP1* knockdown in DMS273 cells. **B**) Immunofluorescent staining depicting the change of γH2AX foci in DMS273 cells upon *TRIM21* knockdown/overexpression or *PARP1* knockdown in DMS273 cells. Scale bars, 10 μm. **C**) Quantification of the γH2AX foci in (**B**). n = 50 cells. **D**) Representative immunofluorescence images of γH2AX in tumor sections. Scale bars, 40 μm. Data show mean ± SD. Statistical analysis was performed using two-tailed unpaired t-tests. Statistical significance is indicated as **P < 0.01, ****P < 0.0001; 'ns' indicates no significant difference.

References

1. Martin OA, Bonner WM. γ-H2AX in Cancer Cells: A Potential Biomarker for Cancer Diagnostics, Prediction and Recurrence. Cell Cycle **2006**;5:2909-13

2. Cornelissen B, Kersemans V, Darbar S, Thompson J, Shah K, Sleeth K, *et al.* Imaging DNA Damage In Vivo Using γH2AX-Targeted Immunoconjugates. Cancer Research **2011**;71:4539-49

3. Maresca C, Dello Stritto A, D'Angelo C, Petti E, Rizzo A, Vertecchi E, *et al.* PARP1 allows proper telomere replication through TRF1 poly (ADP-ribosyl)ation and helicase recruitment. Communications Biology **2023**;6

8. BMN673 and PKI-587 in Result 8: The combination treatment discussed in this section may not solely target the PI3K/AKT-STAT5A-TRIM21-PARP1 axis, as neither inhibitor is specific to PARP1 or TRIM21. Other mechanisms may contribute to the observed anti-tumor effects. Recent studies (PMID: 37925433) on TRIM21 and specific substrate-targeted therapies may offer insights for developing TRIM21-PARP1-specific treatments.

Response: We agree with the reviewer's insightful critique regarding the specificity of BMN673 and PKI-587 in targeting the PI3K/AKT-STAT5A-TRIM21-PARP1 axis. We acknowledge that our original phrasing may have been imprecise in suggesting these drugs directly "target" this axis.

Instead, BMN673 (a PARP inhibitor) and PKI-587 (a PI3K/AKT inhibitor) likely exert synergistic anti-tumor effects by collectively modulating the activation status of this signaling cascade.

As noted by the reviewer, other mechanisms beyond this axis contribute to the drug synergy. Substantial evidence indicates that PI3K/AKT inhibitors induce significant DNA damage, while PARP inhibitors compromise DNA damage repair pathways. Their combination thus exacerbates genomic instability, leading to synthetic lethality—a well-established rationale for the observed synergy[1-3].

Furthermore, the reviewer highlighted recent studies (PMID: 37925433) demonstrating that TRIM21-mediated degradation of the RNA-binding protein HuR serves as a potent anti-tumor strategy. This finding inspired an additional hypothesis: PKI-587 may transcriptionally upregulate *TRIM21*, thereby promoting PARP1 degradation to suppress its oncogenic functions. So, we propose that this TRIM21-PARP1 regulatory axis could also contribute to the observed synergy between BMN673 and PKI-587. We have incorporated these insights into our revised discussion and have specifically cited the referenced study (PMID: 37925433, as reference 69 in revision), with key details highlighted in Lines 522-526.

References

1. Cardnell RJ*, et al.* Proteomic markers of DNA repair and PI3K pathway activation predict response to the PARP inhibitor BMN 673 in small cell lung cancer. *Clin Cancer Res* **19**, 6322-6328 (2013).

2. Ibrahim YH*, et al.* PI3K Inhibition Impairs BRCA1/2 Expression and Sensitizes BRCA-Proficient Triple-Negative Breast Cancer to PARP Inhibition. *Cancer Discovery* **2**, 1036-1047 (2012).

3. Bian X*, et al.* PTEN deficiency sensitizes endometrioid endometrial cancer to compound PARP-PI3K inhibition but not PARP inhibition as monotherapy. *Oncogene* **37**, 341-351 (2018).

Minor concerns:

1. Provide evidence to support the effects of all shRNA, siRNA, and overexpression constructs (TRIM21 and PARP1) in both in vivo and in vitro experiments.

Response: Thank you for your suggestion. All genetic tools used in this study were rigorously validated: For *TRIM21* manipulation, we confirmed the knockdown efficiency of two distinct shRNAs (sh*TRIM21* #1 and #2) and two siRNAs (si*TRIM21* #1 and #2), as well as the overexpression

efficacy of two constructs (EGFP-*TRIM21* and CHD-*TRIM21*) *in vitro* (Response Figure 5A, B, designated as Figure 2D, E in revision). Similarly, all corresponding *PARP1*-targeting constructs (shRNA, siRNA, and overexpression vector) used throughout our experiments have been systematically validated, with newly added data demonstrating their functional efficacy in relevant experimental systems (Response Figure 5C). The efficacy of all shRNA, siRNA, and overexpression constructs targeting *TRIM21* and *PARP1* used in the *in vivo* experiments of this study was primarily determined through IHC analysis (Response Figure 5D, E, designated as Figure 3N, 4I in revision). These comprehensive validations ensure the reliability of our genetic manipulations across all reported findings.

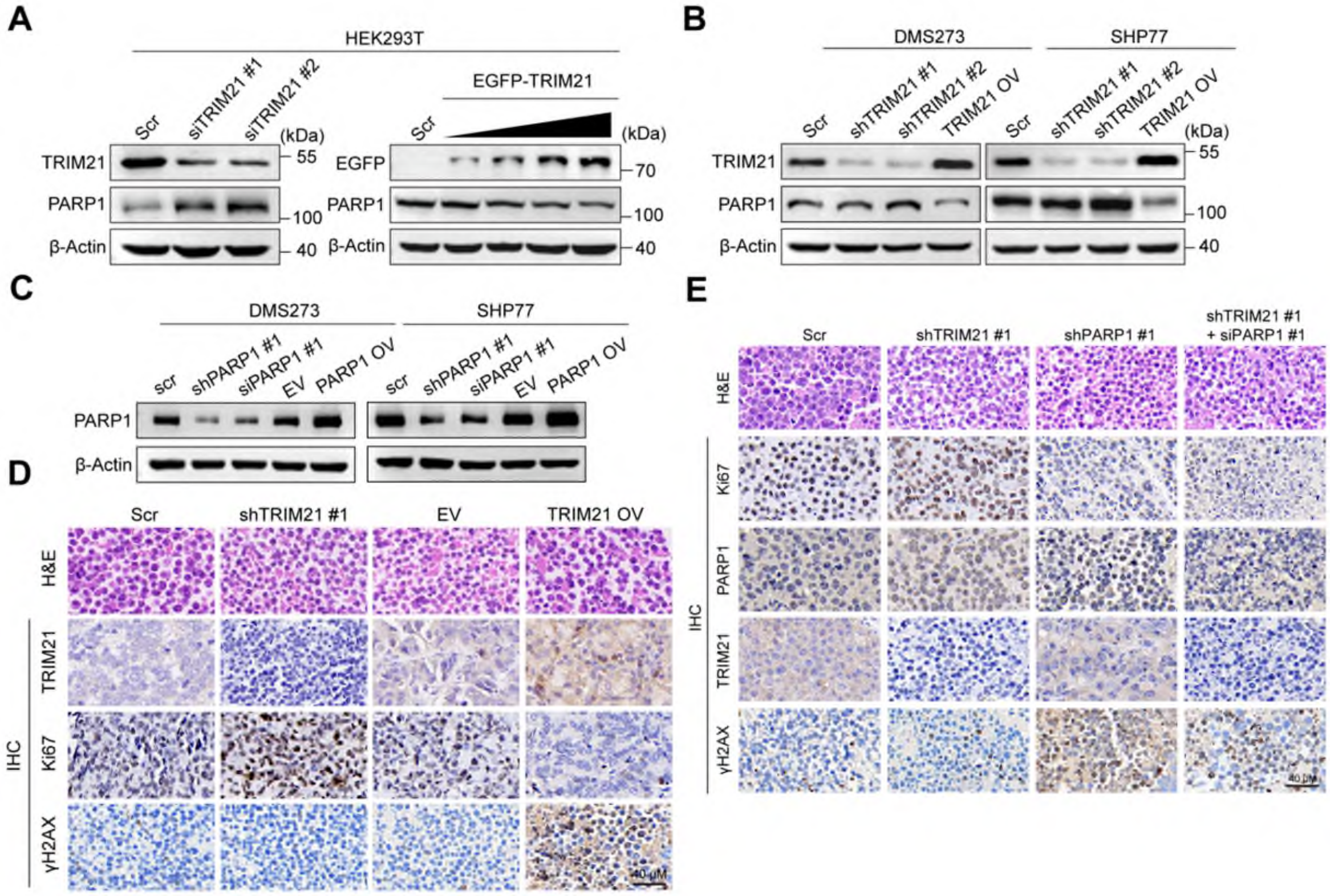

Response Figure 5

Response Figure 5. **A**) Influence of *TRIM21* levels on PARP1 expression. HEK293T cells were modulated to express varying levels of *TRIM21*. The levels of TRIM21 and PARP1 were determined by Western blot. **B**) Assessment of PARP1 expression following *TRIM21* modulation. Western blot analyses were performed to assess PARP1 expression after knockdown and overexpression of *TRIM21* in DMS273 and SHP77 cells. **C**) Validation of *PARP1* knockdown (shRNA/siRNA) and overexpression in DMS273 and SHP77 cells. **D**) Representative images of HE staining and immunohistochemistry for TRIM21, Ki67, and γH2AX within tumor sections. Scale bars, 40 μm. **E**) Representative images of HE staining and immunohistochemistry for Ki67, PARP1, TRIM21, and γH2AX within tumor sections. Scale bars, 40 μm.

2. Figures 4A-B do not adequately support the conclusion in lines 212-214, as no PARP1 overexpression group was included. According to the figure legend, only TRIM21 knockdown or overexpression cells were used, not PARP1 overexpression cells.

Response: We fully endorse the reviewer's critical observation. As addressed in our response to your Major concern 6, we have repeated these experiments with the following critical additions: 1) *PARP1*-knockdown alone groups; 2) *PARP1*-overexpression alone groups; 3) concurrent measurement of PARP1 and TRIM21 protein levels across all conditions. These newly added data—integrated into revised Supplementary Figure S4A, B (Response Figure 3A, B) and Figure 4A, B (Response Figure 3C, D)—demonstrated that TRIM21's effects on SCLC cell survival are specifically mediated through PARP1 regulation, as evidenced by reversal of *TRIM21*-knockdown phenotypes upon *PARP1* ablation and partial rescue of *TRIM21*-overexpression effects by PARP1 restoration, with corresponding changes in PARP1 protein abundance confirming the destabilization mechanism. This experimental validation solidly supports our conclusion that TRIM21 inhibits SCLC cell survival through PARP1 destabilization.

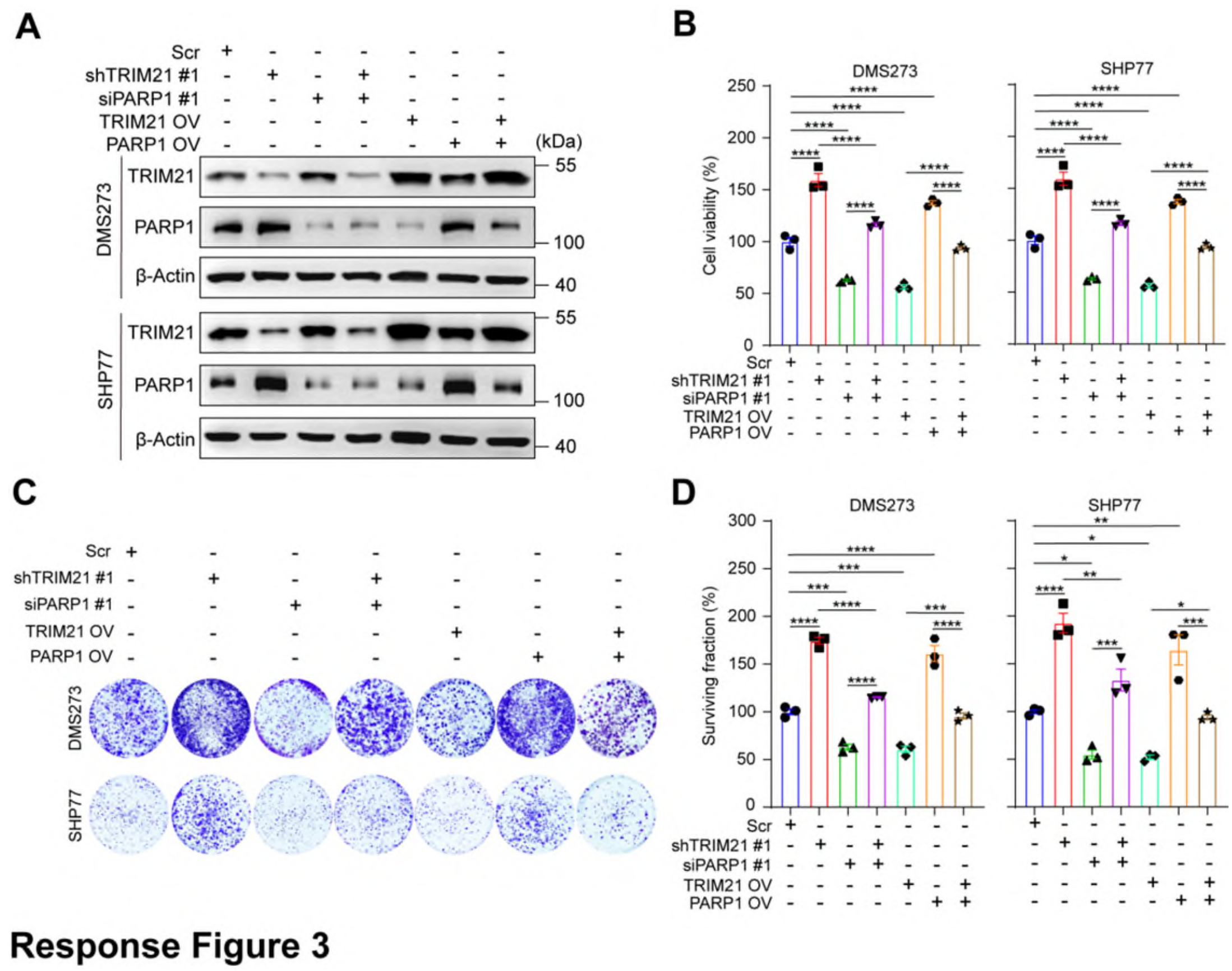

Response Figure 3. **A**) Western blot analysis investigating the compensatory effects of PARP1 on altered *TRIM21* expression. **B**) Cell viability assessment using the CellTiter-Glo luminescent assay showing the compensatory effect

of *PARP1* on cell survival in *TRIM21* knockdown or overexpressed cells. **C**) Colony formation assays illustrating PARP1-mediated rescue of capacity in *TRIM21*-knockdown or -overexpressing cells. **D**) Quantitative analysis of colony formation shown in (**C**). Data are represented as mean ± SEM, and were statistically analyzed using a one-way ANOVA. Significance is denoted as follows: *$P < 0.05$; **$P < 0.01$; ***$P < 0.001$; ****$P < 0.0001$.

Reviewer #2 (Remarks to the Author):

Summary

The manuscript titled "TRIM21-mediated ubiquitination of PARP1 suppresses small cell lung cancer progression through the PI3K/AKT/STAT5A axis" investigates the molecular mechanisms of PARP1 upregulation in SCLC and identifies that PARP1 as a novel substrate of E3 ubiquitin ligase TRIM21 which is regulated via the PI3K/AKT-STAT5A axis.

The authors conducted a comprehensive assessment of PARP1 as a putative ubiquitination and degradation substrate of TRIM21. In addition, they showed that loss of TRIM21 promotes SCLC tumor growth and genomic stability. The authors identified that PI3K/AKT pathway downregulates TRIM21 expression by inhibiting STAT5A transcription factor. Finally, the authors demonstrated the translational potential of the study by showing the synergistic effects of PARP inhibitor and PI3K/AKT inhibitor combination in SCLC.

This work presents a novel and intricate mechanism of PARP1 upregulation in SCLC, offering valuable insights into the mechanisms of SCLC tumor progression and potential therapeutic targets. While the concept of synergistic effects from the combination of a PI3K/AKT inhibitor with a PARP inhibitor has been previously reported (Cardnell et al., doi:10.1371/journal.pone.0152584), this study provides additional understanding of why this combination is effective in the context of SCLC.

Response: Thanks for the general positivity to our study. We fully agree with the reviewer's observation that the synergistic efficacy of combining PI3K/AKT inhibitors with PARP inhibitors has been previously explored, and we deeply value the opportunity to clarify how our work builds upon existing knowledge. While prior studies, such as the report by Cardnell *et al*. (now cited as reference 67 in the revised discussion), established the therapeutic promise of this combination, our work uniquely identifies the "PI3K/AKT-STAT5A-TRIM21-PARP1" axis as a critical mechanism driving PARP1 stabilization in SCLC, providing mechanistic clarity to prior observations. By defining TRIM21's role in ubiquitinating PARP1 and linking STAT5A-mediated transcriptional regulation to PI3K/AKT signaling, we offer a rational framework for optimizing this therapeutic combination in SCLC. We appreciate the opportunity to emphasize these mechanistic advances and their translational implications in this study.

Per Figure summary below:

Fig 1 PARP1-TRIM21 interaction assays. IP, co-IP, PLA. Structure function analysis. PP domain of TRIM21 required for PARP1 interaction. HD domain of PARP1 required for TRIM21 interaction his His pull-down assays. DMS273, SHP77 SCLC cell lines. And HEK 293T used in various combinations for the above assays. 293 used only for the domain domain interaction analyses.

Fig 2 – CCLE proteomics correlation and westerns of cell-lines for TRIM21 and PARP1, in sum negative correlation between TRIM21 and PARP1 protein levels. siTRIM21 in 293, SHP77 and DMS273 cells led to increased PARP1 protein levels. Cycloheximide chase shoed TRIM21 affects PARP1 protein half-life in DMS273 and SHP77 cells. HA-Ub K48R mutant showed less PARP1-Ub. IN vitro Ub assays showing Ub-PARP1 with 293 cells.

Fig 3- TRIM21 mRNA lower in SCLC cell lines and tumors vs normal. shTRIM21 increased DMS273 and SHP77 clonogenicity whereas TRIM21-OE decreased clonogenicity. Similar in xenograft analysis of DMS273 by shTRIM21 and TRIM21-OE

Fig 4 – Partial rescue with PARP1-OE in DMS273 and SHP77 cells after TRIM21-OE. PARP1-K654R increased clonogenicity in DMS273 and SHP77. In in vivo experiments with shTRIM21 and shPARP1. Adding siPARP1 reverses shTRIM21 alone protumorgenic effects.

Fig 5 – Less DNA damage in TRIM21 knockdown, less gH2AX and comet tails, and increased viability in DMS273 cells. Some assays with SHP77 as well.

Fig 6- PI3K/AKT pathway correlation analysis with TRIM21 mRNA. PI3Ki increased TRIM21 mRNA. AKT-OE inhibited TRIM21 exp and increased PARP1 levels.

Fig 7 – Transcriptional factor analysis identified 6 candidate transcription factors that bind to TRIM21 promoter, correlation analysis surfaced STAT5A, TCF21 and SIX5. STAT5A knockdown reduced TRIM21 mRNA but not PARP1 mRNA. PI3K/AKT inhibition enhanced STAT5A binding to TRIM21 promoter.

Fig 8 – In vitro and in vivo analysis of PARPi (talazoparib) and PI3K inhibition. SHP77 and DMS273 clonogenic drug experiments with PI3Ki and PARPi. DMS273 xenografts same treatments.

Response: We are profoundly grateful to the reviewer for their exceptionally thorough and meticulous summary of each figure in our manuscript.

The following Major Revisions are recommended:

1. Additional experiments using SCLC cell lines of POU2F3 (SCLC-P) and YAP/Inflamed (SCLC/Y/I) subtypes are recommended to prove the effects of TRIM21 are universal across SCLC subtypes. Experiments such as knocking

down TRIM21 or overexpressing WT/mutant TRIM21 following examination of cell viability, clonogenicity, and genome stability should be done in cell lines of SCLC-P/Y/I subtypes.

Response: Thanks for your valuable suggestion. We have conducted additional experiments using representative cell lines of the SCLC-*P* (H526) and SCLC-*Y* (H196) subtypes by either knocking down *TRIM21* or overexpressing wild-type/mutant *TRIM21* (including the ligase-deficient mutant, LD). Our results demonstrated that *TRIM21* knockdown significantly enhanced cell viability, clonogenic potential, and DNA damage response capability in both H526 and H196 cells, while *TRIM21* overexpression showed opposite effects (Response Figure 6A-E, designated as Supplementary Figure S3G-I and Figure S5C-F in revision). Importantly, the *TRIM21*-LD mutant failed to significantly alter these cellular phenotypes in either subtype (Response Figure 6A-E). These findings exhibit remarkable consistency with our initial observations regarding TRIM21's function in SCLC-*N* (DMS273) and SCLC-*A* (SHP77) subtypes, suggesting that TRIM21's tumor-suppressive function is not subtype-specific but rather represents a common regulatory mechanism in SCLC pathogenesis. These contents have been incorporated into the revised manuscript (Lines 209-212 and 262-266). We believe these comprehensive results have significantly strengthened the generalizability of our conclusions regarding TRIM21's role in SCLC progression.

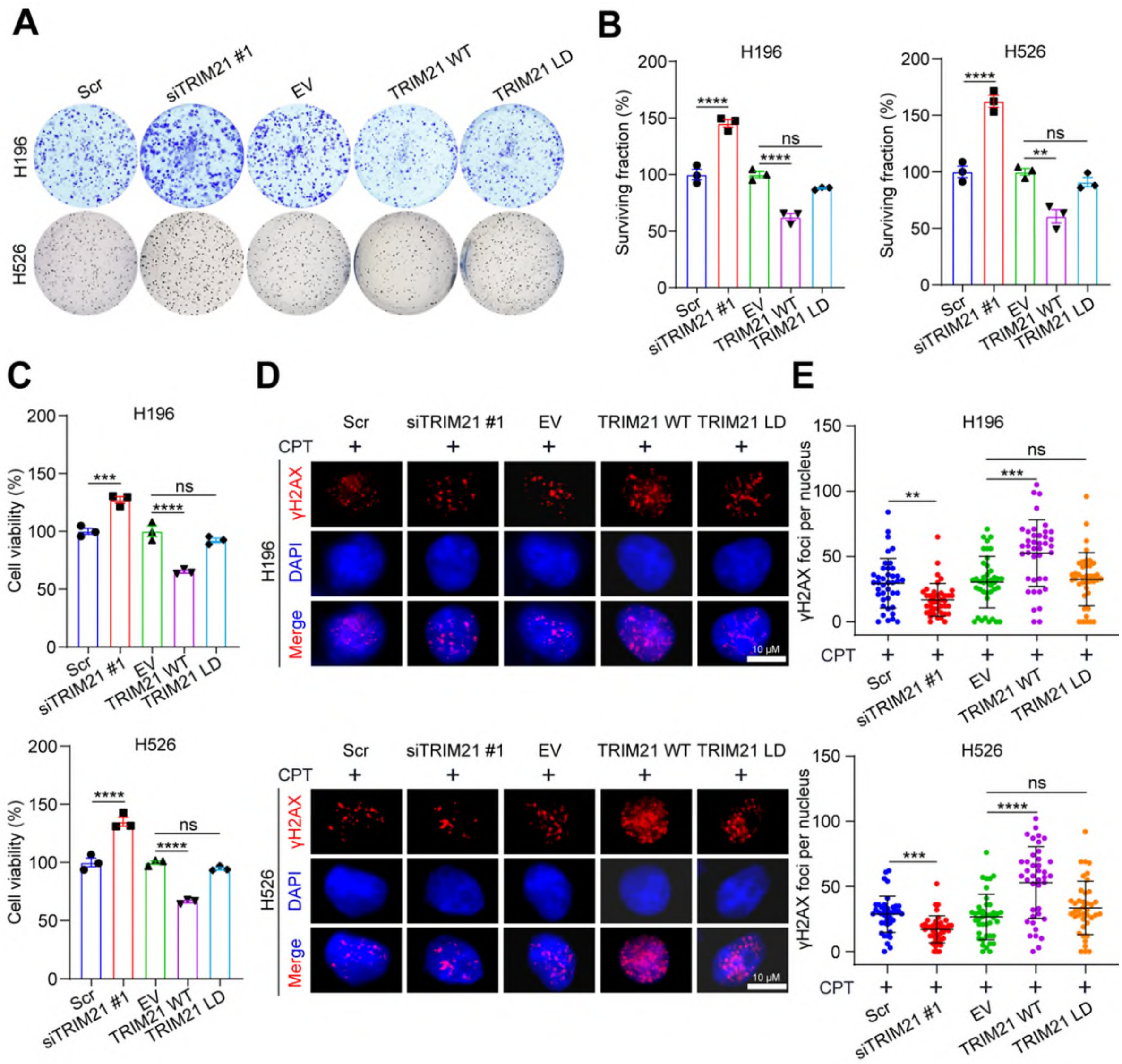

Response Figure 6

Response Figure 6. **A**) Colony formation assays in H526 and H196 cells following either *TRIM21* knockdown (si*TRIM21*) or overexpression of wild-type (WT) or ligase-deficient mutant (LD) *TRIM21*. For the suspension cell line H526 in soft agar colony formation assays. **B**) Quantification of clonogenic survival from (**A**) using ImageJ software. **C**) Cell viability measured by CellTiter-Glo assay in H526/H196 cells with si*TRIM21*, WT-*TRIM21*, or LD-*TRIM21* expression. **D**) Immunofluorescence analysis of γH2AX foci in SCLC cells treated with 2 μM camptothecin (1 hour) followed by 4 hours recovery under si*TRIM21*, WT-*TRIM21*, or LD-*TRIM21* conditions. Scale bars, 10 μm. **E**) γH2AX foci quantification from (**C**); n = 50 cells/group. Data are mean ± SEM in (**B**) and (**C**). Data show mean ± SD in (**E**). Statistical analysis was performed using two-tailed unpaired t-tests. Statistical significance is indicated as 'ns' indicates no significant difference; $^{**}P < 0.01$; $^{***}P < 0.001$; $^{****}P < 0.0001$.

2. The authors should replicate the cycloheximide chase assay (figure 2F) to include the level of TRIM21 over time.

Response: Thank you for raising this critical point. We have supplemented the cycloheximide chase

assay in Figure 2F to include TRIM21 protein levels over time, with the updated experimental results now integrated into the revision (Response Figure 7A, designated as Figure 2F in revision).

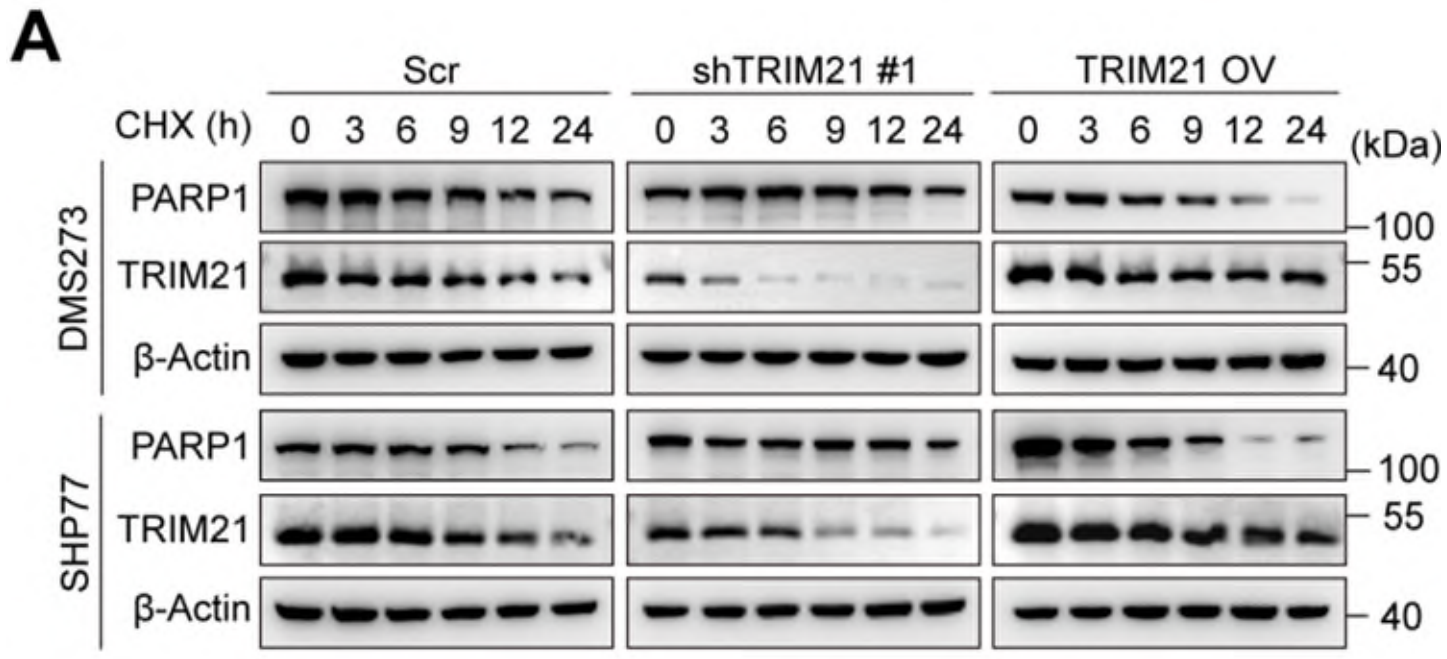

Response Figure 7

Response Figure 7. **A**) The effect of altered TRIM21 expression levels on PARP1 stability. The DMS273 and SHP77 cells with *TRIM21* knockdown or overexpression were treated with CHX (100 μg/ml) for different durations, and PARP1 and TRIM21 protein levels were assessed at various time points by Western blot.

3. DMS273 and SHP-77 SCLC cell lines are moderately sensitive to PARPi monotherapy. Additional experiments using PARPi-resistant cell lines to examine the efficacy of PI3K and PARPi combination is needed.

Response: We thank the reviewer for giving us this valuable suggestion. We have conducted experiments using PARPi-resistant cell lines to evaluate the efficacy of the PI3K/PARPi combination. We first analyzed BMN673 sensitivity across SCLC lines and confirmed the reviewer's observation: DMS273 shows moderate sensitivity, while H196 exhibits marked resistance to BMN673 (Response Figure 8A). Quantitative IC_{50} analysis revealed that H196 has an IC_{50} of ~20.23 μM, approximately 100-fold higher than DMS273 (IC_{50} = 0.2095 μM) (Response Figure 8B), definitively establishing H196 as a PARPi-resistant model. When assessing sensitivity to the PI3K/AKT inhibitor PKI-587, both lines showed broadly comparable responses despite a modest trend toward greater sensitivity in DMS273 (Response Figure 8C). Crucially, combination therapy with BMN673 and PKI-587 at optimized doses induced significant synergistic antitumor effects even in H196 cells (Response Figure 8D, E). These results elucidate that PI3K/AKT inhibition via PKI-587 effectively sensitizes PARPi-resistant SCLC to PARPi.

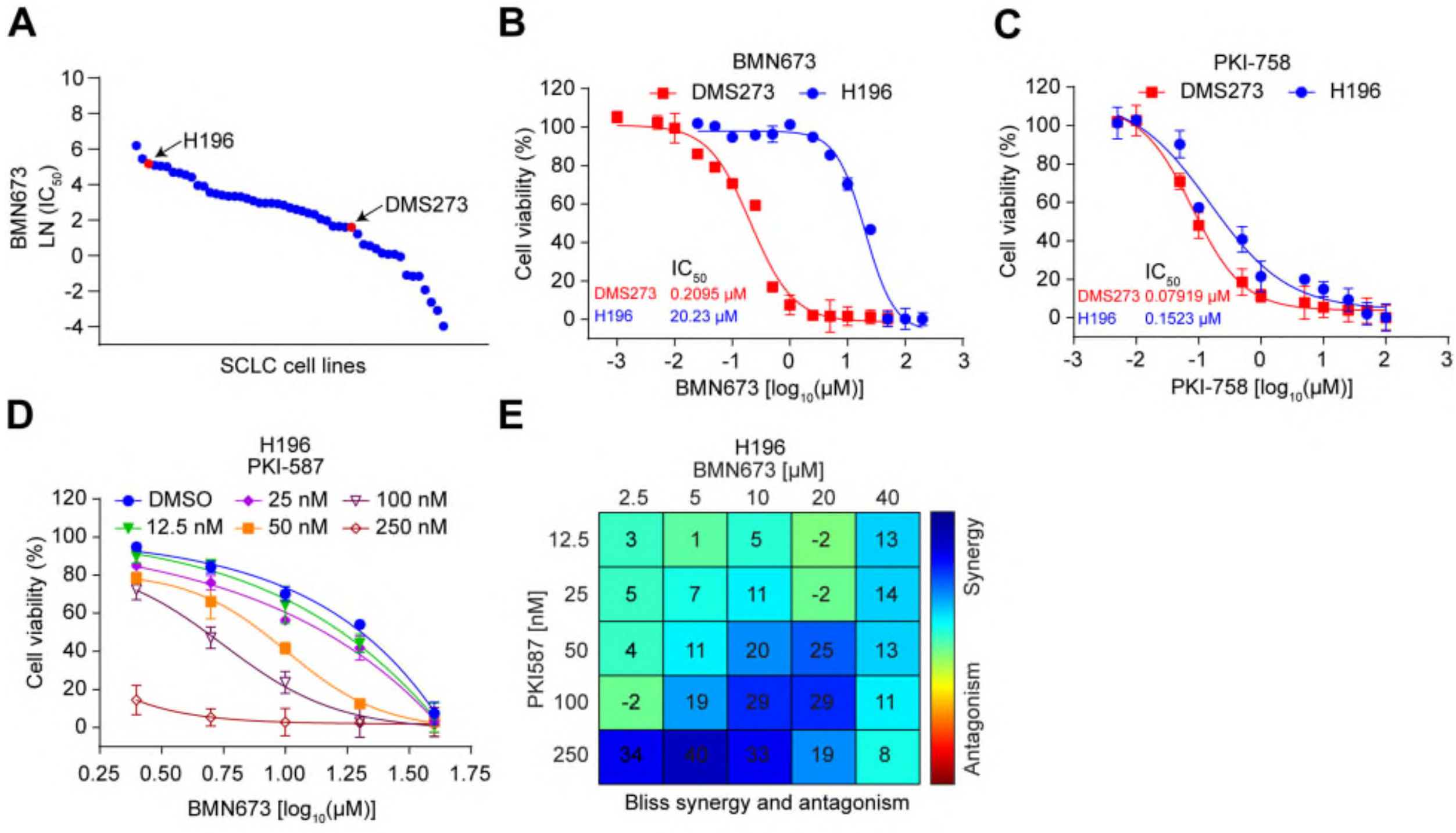

Response Figure 8

Response Figure 8. **A**) Differential sensitivity of SCLC cell lines to BMN673. The Half-lethal inhibitory concentrations (IC_{50}) of BMN673 across 51 SCLC cell lines from the GDSC2 database. **B**) The IC_{50} values of BMN673 were determined from the sigmoidal dose-response curves in DMS273 and H196 cell lines. **C**) The IC_{50} values of PKI-587 were determined from the sigmoidal dose-response curves in DMS273 and H196 cell lines. **D**) Dose-response curves of BMN673 and PKI-587 in H196 cells. Cells were treated with increasing concentrations of BMN673 and PKI-587 for 72 hours, followed by cell viability measurement using CellTiter-Glo luminescent assay. **E**) Bliss synergy analysis of the BMN673-PKI-587 combination. Synergy indices were calculated using Combenefit software (CRUK Cambridge Institute) based on the dose-response data from (**D**).

4. Talazoparib is a potent PARP trapper and enzymatic inhibitor of PARP function. Is the synergistic effect with PI3K inhibition related to PARP trapping vs the enzymatic inhibition? Veliparib as an enzymatic inhibitor with minimal PARP trapping could be an approach to deconvolute this. (Fig 8)

Response: We concur with the reviewer's astute observation regarding the distinct mechanistic contributions of PARP trapping versus catalytic inhibition in mediating the synergistic effects with PI3K/AKT pathway blockade. To address this, we selected Veliparib, an enzymatic inhibitor with minimal PARP trapping activity, and evaluated its single-agent efficacy against SCLC cells (DMS273) and its combination effects with the PI3K/AKT inhibitor PKI-587. Our data demonstrated that Veliparib exhibits an IC_{50} of ~62.22 μM in DMS273 cells (Response Figure 9A), significantly higher than talazoparib (BMN673; IC_{50} = 0.2416 μM), likely attributable to talazoparib's dual

mechanism (enzymatic inhibition + PARP trapping) versus Veliparib's predominant enzymatic inhibition. Crucially, although Veliparib monotherapy showed modest activity, its combination with PKI-587 at appropriate doses still produced marked synergistic antitumor effects (Response Figure 9B, C). These results indicate that the synergistic mechanism between PARP inhibitors and PI3K/AKT inhibition is not exclusively dependent on PARP trapping activity.

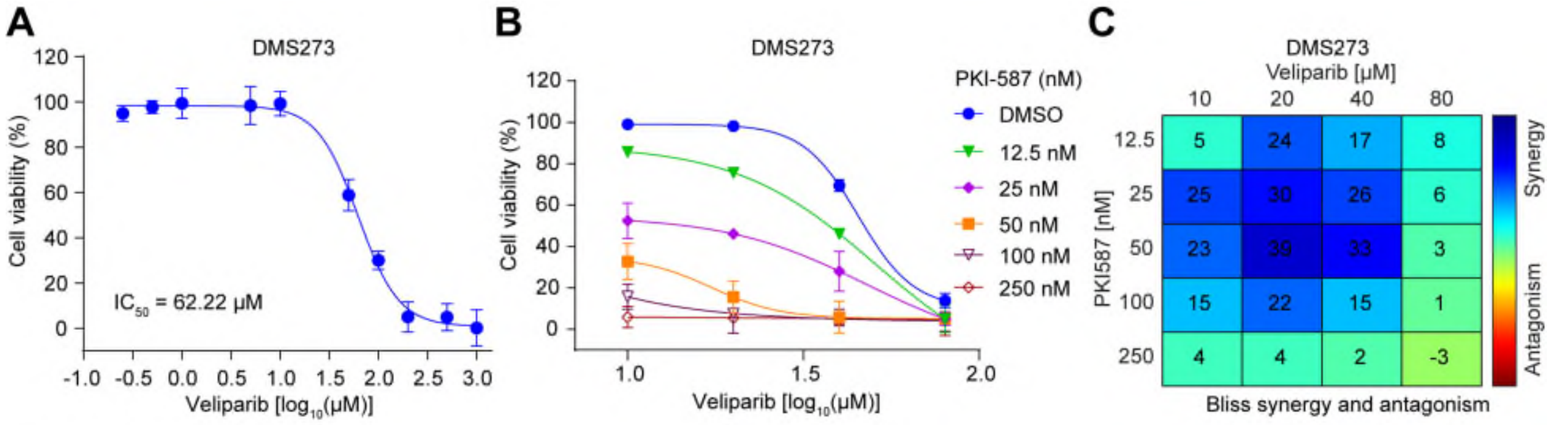

Response Figure 9

Response Figure 9. **A**) The IC_{50} value of Veliparib was determined from the sigmoidal dose-response curves in the DMS273 cell line. **B**) Dose-response curves of Veliparib and PKI-587 in DMS273 cells. Cells were treated with increasing concentrations of Veliparib and PKI-587 for 72 hours, followed by cell viability measurement using CellTiter-Glo luminescent assay. **C**) Bliss synergy analysis of Veliparib-PKI-587 combination. Synergy indices were calculated using Combenefit software (CRUK Cambridge Institute) based on the dose-response data from (**B**).

5. Additional SCLC molecular subtypes for PI3Ki and PARPi in vitro synergy analyses would be valuable (Fig 8).

Response: In accordance with the reviewer's valuable suggestion, we have accordingly extended our *in vitro* synergy analyses to additional SCLC molecular subtypes, specifically evaluating *P*-subtype (H526) and *Y*-subtype (DMS114) cell lines, to assess PI3K/AKT inhibitor (PKI-587) and PARP inhibitor (BMN673) monotherapy efficacy and combination effects. The results demonstrated that BMN673 monotherapy exhibits differential sensitivity across subtypes with an IC_{50} of 1.196 μM in H526 (*P*-subtype) versus 13.52 μM in DMS114 (*Y*-subtype) (Response Figure 10A, designated as Supplementary Figure S9B in revision), whereas PKI-587 shows comparable potency in both lines (IC_{50} = 0.1205 μM in H526 vs. 0.1964 μM in DMS114) (Response Figure 10B, designated as Supplementary Figure S9B in revision). Crucially, combination therapy at optimized doses induced significant synergistic effects in both H526 and DMS114 cells (Response Figure 10C-F, designated as Supplementary Figure S9K, S9L, S9O, and S9P in revision). These findings indicate that while

PARPi sensitivity varies across SCLC molecular subtypes, PI3K/AKT inhibition maintains consistent activity. Increasingly, the synergistic effects between these drug classes occur independently of SCLC subtypes when appropriately dosed. These results have been incorporated into the revised manuscript (Lines 377-388).

Additionally, we also evaluated the combined efficacy of these two drugs across SCLC organoid models representing different molecular subtypes (Response Figure 11). The detailed results are provided in our response to your next question.

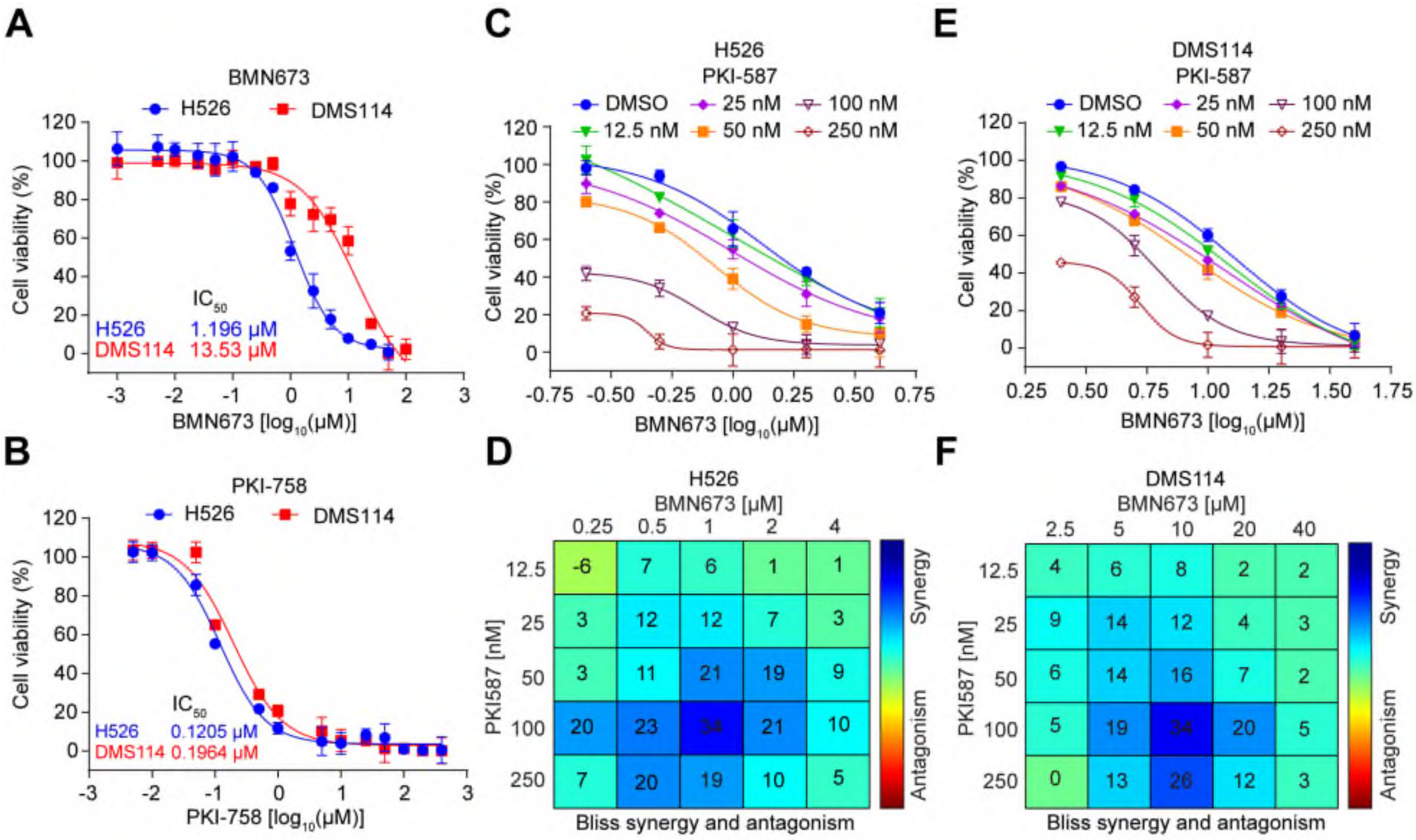

Response Figure 10

Response Figure 10. **A**) BMN673 IC_{50} values derived from sigmoidal dose-response curves in H526 and DMS114 cell lines. **B**) PKI-587 IC_{50} values derived from sigmoidal dose-response curves in H526 and DMS114 cell lines. **C**, **E**) Dose-response profiles of BMN673 combined with PKI-587 in H526 (**C**) and DMS114 (**E**) cells. Cells were treated with increasing concentrations of BMN673 and PKI-587 for 72 hours, followed by cell viability measurement using CellTiter-Glo luminescent assay. **D**, **F**) Bliss synergy analysis of the combination of BMN673 and PKI-587 in H526 (**D**) and DMS114 (**F**) cells. Synergy indices were calculated using Combenefit software (CRUK Cambridge Institute) based on the dose-response data from (**C**, **E**).

6. Additional in vivo model(s) would be valuable and confirm translational impact, esp in other molecular subtypes or with any available patient-derived xenograft models. (Fig 8)

Response: We greatly appreciate the reviewer's insightful suggestion regarding additional validation across SCLC molecular subtypes *in vivo*. In response, we have conducted comprehensive analyses using patient-derived organoid models representing distinct SCLC subtypes (*A*: SC049, *A* + *N*: SC081, *P*: SC080, and *Y*: SC030).

Our results demonstrated that PARP1 expression is significantly higher in *A* and *A* + *N* subtypes compared to the *P* subtype, with the lowest expression observed in the *Y* subtype (Response Figure 11A, designated as Figure 8B in revision). Correspondingly, PARP inhibitor (BMN673) sensitivity was greatest in *A* and *A* + *N* subtypes, intermediate in *P* subtype, and lowest in *Y* subtype (Response Figure 11B, designated as Figure 8C in revision), establishing a clear correlation between PARP1 expression levels and therapeutic response to BMN673 across different SCLC molecular subtypes.

Concomitantly, our results also demonstrated that PI3K signaling activity remains remarkably consistent across all molecular subtypes, with no significant differences observed in either basal pathway activation levels or therapeutic response to PI3K/AKT inhibitor (PKI-587) (Response Figure 11C, D, designated as Figure 8D, E in revision), suggesting that PKI-587 sensitivity may be independent of molecular subtypes in SCLC.

We further evaluated the combination effects of BMN673 and PKI-587 across SCLC subtypes using patient-derived organoid models (*A*: SC049, *A* + *N*: SC081, *P*: SC080, and *Y*: SC030). The results demonstrated synergistic effects in all subtypes (Response Figure 11E-L, designated as Figure 8F-I and Supplementary Figure S10A-D in revision), suggesting that the antitumor synergy between BMN673 and PKI-587 occurs independently of SCLC subtypes.

Collectively, these data indicate that: 1) BMN673 sensitivity correlates with PARP1 expression levels rather than molecular subtypes; 2) PKI-587 efficacy is similarly subtype-independent; 3) Most importantly, the therapeutic synergy between BMN673 and PKI-587 is consistently observed across all SCLC subtypes, suggesting broad clinical applicability of this combination strategy regardless of tumor classification. These contents have been incorporated into the revised manuscript (Lines 389-401 and 1357-1365).

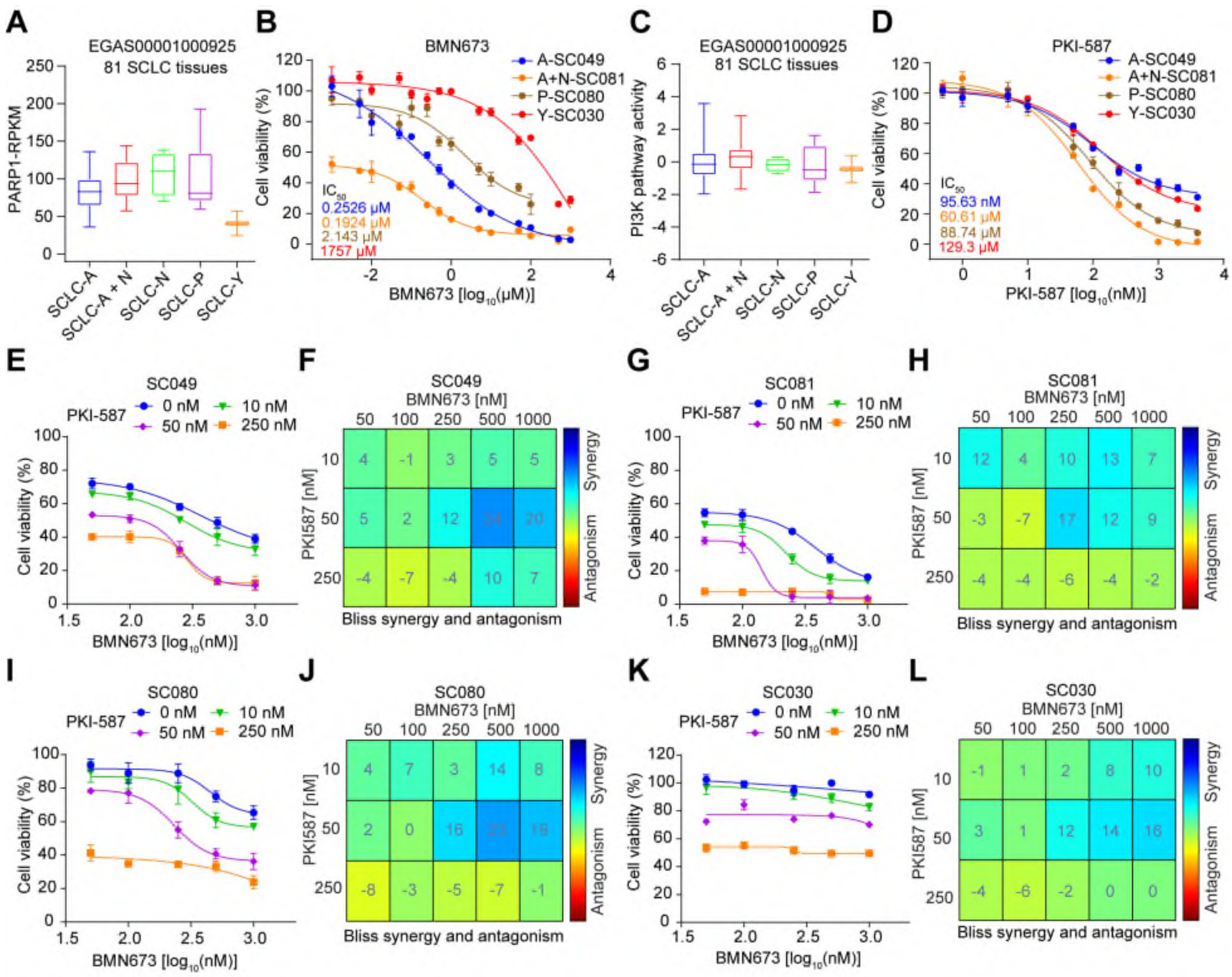

Response Figure 11

Response Figure 11. A) Systematic evaluation of PARP1 expression levels across molecularly defined SCLC subtypes (*A*, *A* + *N*, *N*, *P*, and *Y*) using the EGAS00001000925 dataset. **B**) Quantitative determination of BMN673 IC_{50} values through sigmoidal curve fitting of dose-response relationships in subtype-representative SCLC organoid models (*A*: SC049; *A* + *N*: SC081; *P*: SC080; *Y*: SC030). **C**) Comparative analysis of PI3K pathway activation scores across SCLC subtypes derived from the EGAS00001000925 dataset. **D**) The IC_{50} values of PKI-587 were determined from the sigmoidal dose-response curves in distinct subtypes of SCLC organoid (subtype designations identical to panel **B**). **E**, **G**, **I**, **K**) Combinatorial dose-response profiles illustrating the effects of BMN673 and PKI-587 co-treatment across all four subtypes (*A*: SC049, **E**; *A* + *N*: SC081, **G**; *P*: SC080, **I**; *Y*: SC030, **K**) following 72 hours of exposure, measured by CellTiter-Glo viability assays. **F**, **H**, **J**, **L**) Bliss synergy analysis of the BMN673 and PKI-587 combination treatment in four molecularly defined SCLC subtypes (*A*: SC049, **F**; *A* + *N*: SC081, **H**; *P*: SC080, **J**; *Y*: SC030, **L**). Quantitative synergy indices were computed using the Bliss independence model implemented in Combenefit software (CRUK Cambridge Institute), with calculations derived from the corresponding dose-response matrices shown in panels **E**, **G**, **I**, and **K**.

The following minor revisions are recommended:

1. Quantification of PLA data in figure 1F.

Response: Following your suggestion, we have now conducted rigorous quantitative analysis of the PLA data in Figure 1F, and the results demonstrated significant PLA signals in both DMS273 and SHP77 cells (Response Figure 12A), confirming robust endogenous PARP1-TRIM21 interactions in these SCLC cell lines.

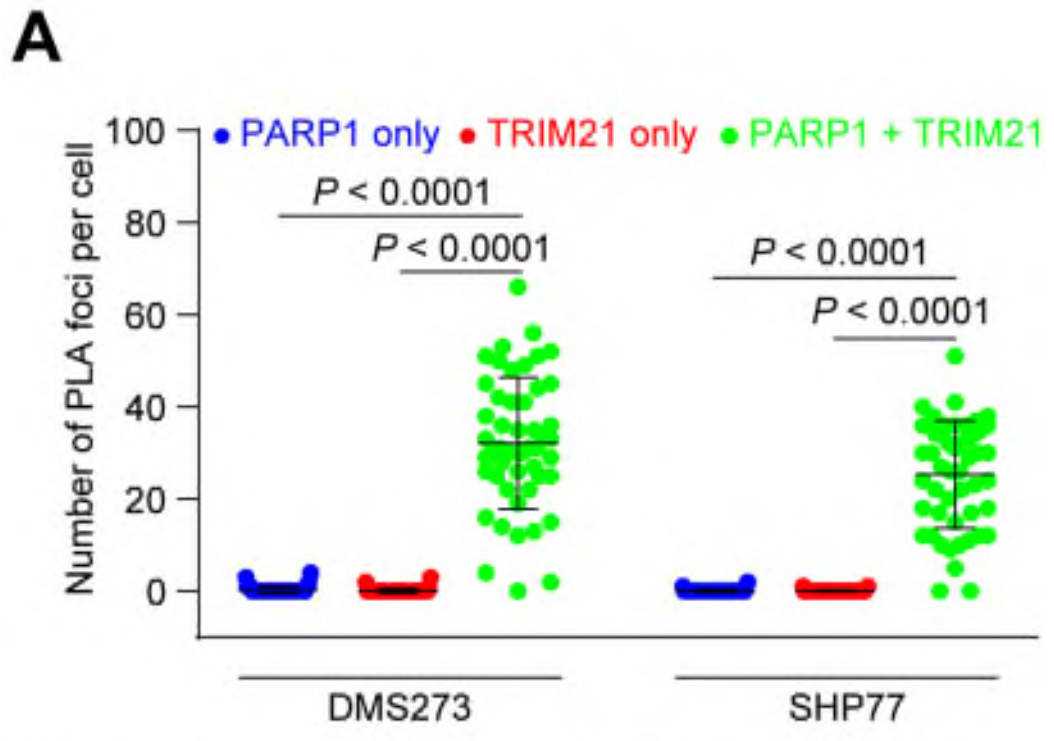

Response Figure 12

Response Figure 12. A) Proximity ligation assay (PLA) was employed to quantitatively assess PARP1-TRIM21 protein interactions in DMS273 and SHP77 cells. Data are represented as mean ± SD, and were statistically analyzed using a one-way ANOVA. $n = 50$, $****P < 0.0001$.

2. Could the authors confirm PARP is proteasomal degraded via proteasomal inhibitor (MG132) experiments? Related to Fig 2F/G, etc.

Response: As suggested, we have performed additional experiments treating DMS273 and SHP77 cells with the proteasome inhibitor MG132. The results demonstrated significant accumulation of PARP1 protein upon MG132 treatment (Response Figure 13A), confirming that PARP1 undergoes proteasomal degradation via the ubiquitin-proteasome pathway.

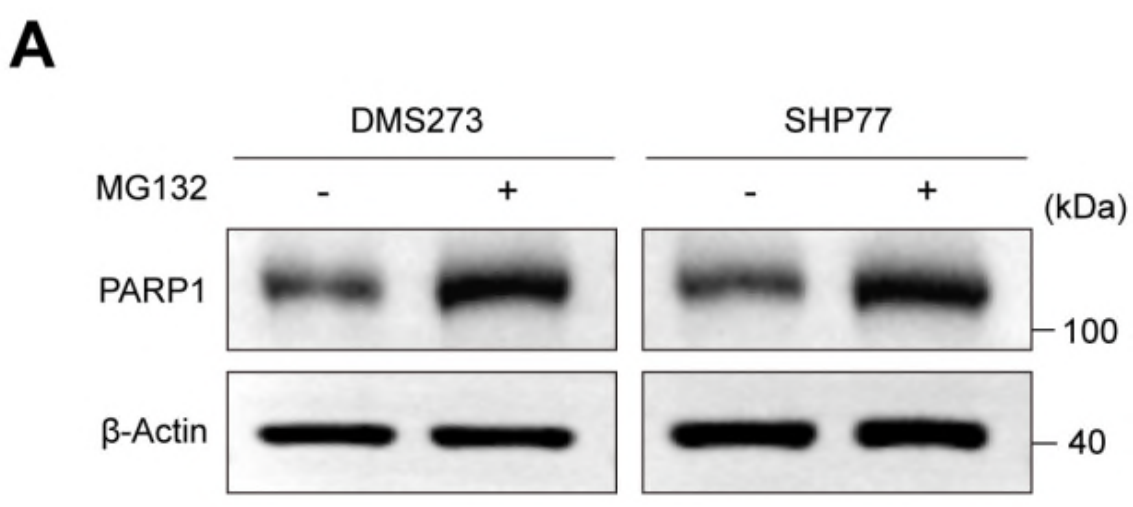

Response Figure 13

Response Figure 13. A) Stabilization of PARP1 protein following proteasome inhibition. DMS273 and SHP77 cells treated with 40 μM MG132 for 12 hours were analyzed by Western blot.

3. For figure 2E, please explain why shTRIM21 #1 barely caused any PARP1 accumulation compared to shTRIM21 #2 despite similar TRIM21 knockdown efficiency.

Response: Sorry for the confusion caused. We have repeated the experiments with overexposed Western blots and performed quantitative analysis. The results demonstrated that both sh*TRIM21* #1 and sh*TRIM21* #2 achieved highly significant *TRIM21* knockdown compared to the control. While the overexposed blots suggested sh*TRIM21* #2 might have slightly stronger knockdown efficiency than sh*TRIM21* #1 (Response Figure 14A, designated as Figure 2E in revision), quantitative analysis revealed no statistically significant difference between them (Response Figure 14B). Similarly, though sh*TRIM21* #2 appeared to cause marginally greater PARP1 accumulation than sh*TRIM21* #1, quantitative analysis showed this difference was not statistically significant, with both shRNAs causing significant PARP1 accumulation compared to the scramble control (Response Figure 14A, B). We have now replaced the original data in Figure 2E with these overexposed blots to more accurately represent the experimental outcomes.

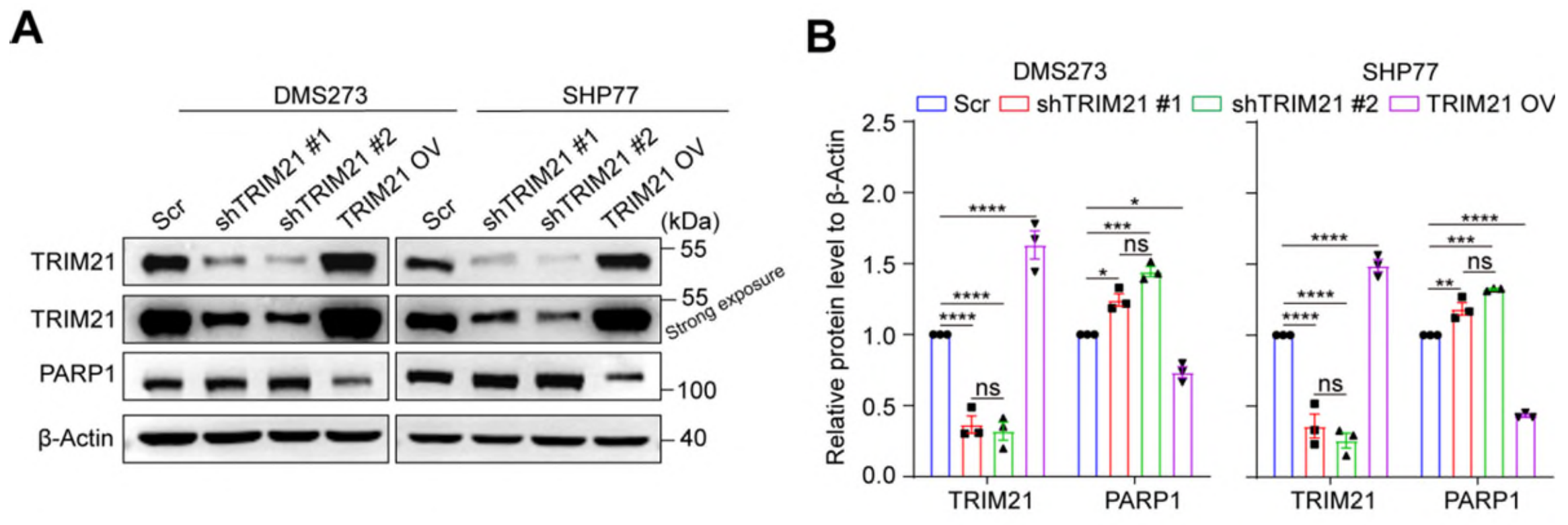

Response Figure 14

Response Figure 14. A) Assessment of PARP1 expression following *TRIM21* modulation. Western blot analyses were performed to assess PARP1 expression after knockdown and overexpression of *TRIM21* in DMS273 and SHP77 cells. **B**) Quantitative densitometric analysis of TRIM21 and PARP1 protein levels from (**A**), normalized to β-Actin loading control and calculated using ImageJ software. Data are represented as mean ± SEM, and were statistically analyzed using a one-way ANOVA. ns, no significance; *$P < 0.05$, **$P < 0.01$, ***$P < 0.001$, ****$P < 0.0001$.

4. An additional experiment can be done to examine global PARylation level changes in TRIM21 knockdown/overexpression cells.

Response: We thank the reviewer for giving us a valuable suggestion and have performed additional experiments examining global PARylation levels in *TRIM21*-modulated cells. Our results demonstrated that *TRIM21* knockdown significantly increased global PARylation levels, whereas

TRIM21 overexpression substantially decreased PARylation (Response Figure 15A). These alterations correlate with PARP1 protein accumulation upon *TRIM21* depletion and reduction after *TRIM21* overexpression, indicating that TRIM21 regulates global PARylation by controlling PARP1 protein stability and consequently modulating PARP1-mediated catalytic activity.

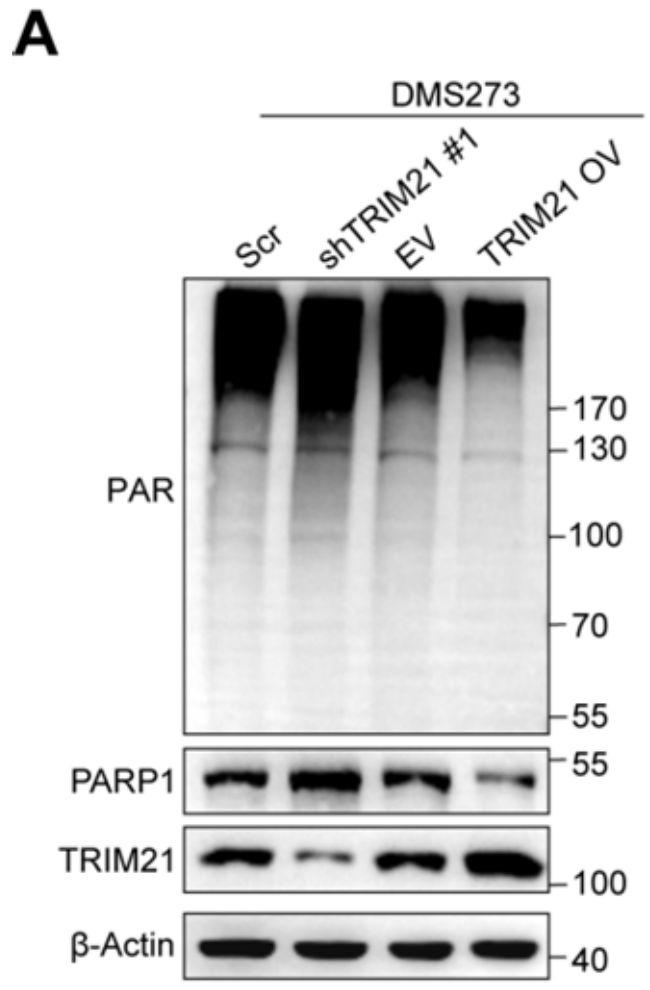

Response Figure 15

Response Figure 15. **A**) Global PARylation levels assessed by western blot using whole-cell lysates from DMS273 cells with *TRIM21* knockdown or overexpression.

5. I am not sure if interpretation of figure S2H is correct. The claim that 'TRIM21 promotes PARP1 ubiquitination under oxidative stress' is not shown properly by the data. The data is rather suggesting that oxidative stress decreases TRIM21-mediated PARP1 ubiquitination. To prove the above claim, additional controls are needed whereby cells are treated with H2O2 or EP without overexpression of EGFP/TRIM21. Additionally, H2O2 or EP treatment increases PARP1 level compared to DMSO treatment (figure S2I) rather than a relative decrease.

Response: We apologize for the imprecise interpretation and experimental limitations in our original submission. As suggested, we have performed additional control experiments treating cells with H_2O_2 or EP without EGFP-*TRIM21* overexpression. The new data demonstrated that oxidative stress (H_2O_2 or EP) alone suppresses PARP1 ubiquitination (lane 2 *vs.* 4, 5) and increases PARP1 accumulation (Response Figure 16A, designated as Supplementary Figure S2H in revision), suggesting cells may attenuate PARP1 degradation to maintain DNA repair capacity under stress. Importantly, while oxidative stress moderately reduces TRIM21-mediated PARP1 ubiquitination (lane 3 *vs.* 6, 7), TRIM21 consistently promotes PARP1 degradation under both normal and stress conditions

(Response Figure 16A). We have replaced Figure S2H with these comprehensive results and revised the text (highlighted in Lines 181-189) to more accurately reflect these findings.

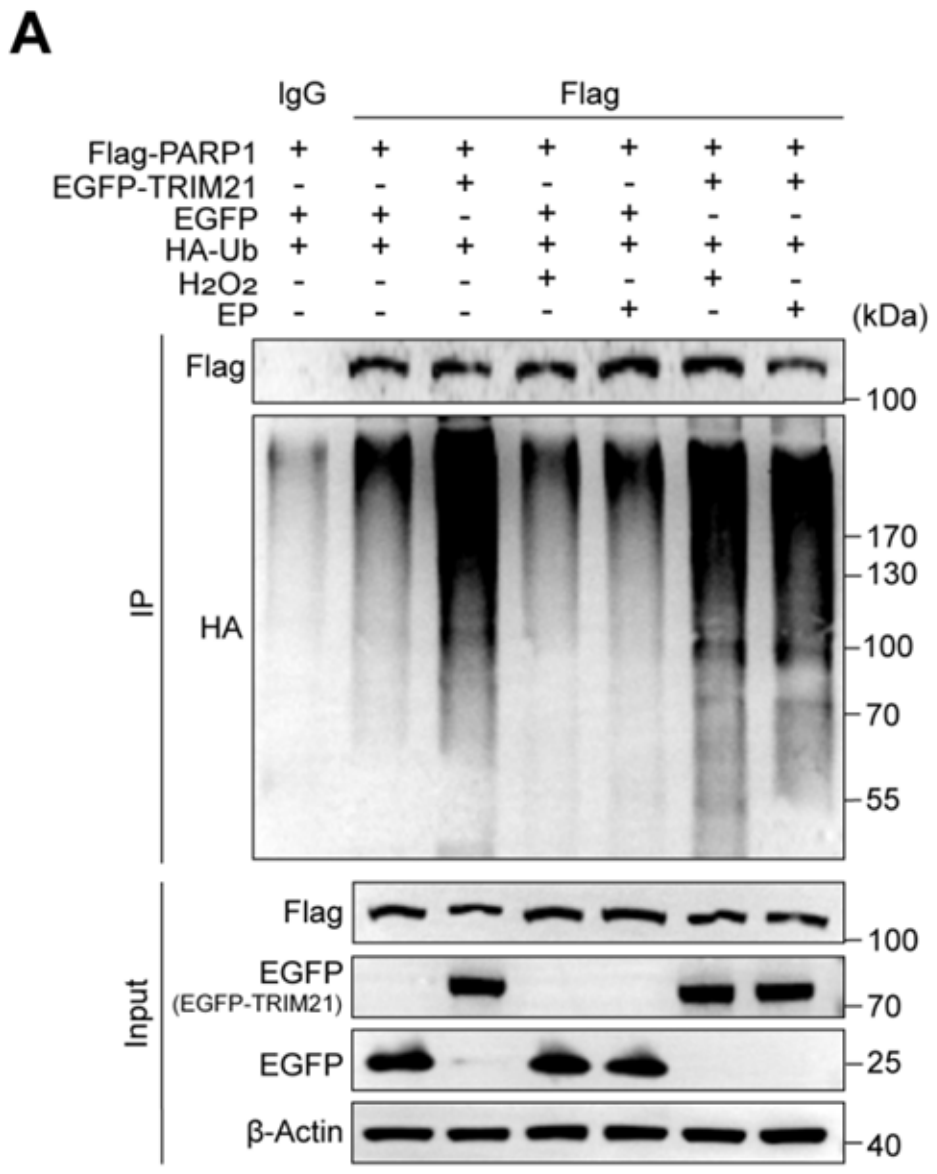

Response Figure 16

Response Figure 16. **A**) Analysis of PARP1 ubiquitination and protein accumulation under DNA damage conditions. HEK293T cells were co-transfected with Flag-*PARP1*, HA-Ub, and either EGFP-***TRIM21*** or EGFP-empty vector. Following treatment with etoposide (EP, 40 μM, 24 h) or H_2O_2 (2 μM, 5 min), PARP1 ubiquitination and protein levels were evaluated by immunoprecipitation-western blotting.

6. Throughout the article, the authors mentioned that the PI3K/AKT-STAT5A-TRIM21-PARP1 signaling axis promotes 'tumorigenesis' of SCLC (see line 350 as an example). I would change the wording to 'tumor growth'. Tumorigenesis refers to when a normal cell become cancerous, which is not shown by this study.

Response: Thanks for the comment. We have carefully revised all relevant instances throughout the manuscript, replacing "tumorigenesis" with the more accurate term "tumor growth" to properly reflect our experimental findings regarding the PI3K/AKT-STAT5A-TRIM21-PARP1 axis's role in promoting established tumor progression rather than initial malignant transformation. This modification significantly improves the precision of our biological interpretations.

7. Consider doing a correlation analysis of TRIM21 expression and chemotherapy or PARPi response in SCLC cell lines. How well can TRIM21 expression predict treatment response?

Response: We fully endorse the reviewer's intriguing suggestion. In response, we have conducted

comprehensive correlation analyses between *TRIM21* expression levels and drug sensitivity across multiple chemotherapeutic classes (alkylating agents, antimetabolites, mitotic inhibitors, antitumor antibiotics, and topoisomerase inhibitors) as well as PARP inhibitors in SCLC cell lines. The results showed no significant correlation between *TRIM21* expression levels and IC_{50} values for any of these drug classes (Response Figure 17A). Similarly, our analysis also revealed no significant correlation between *TRIM21* expression levels and IC_{50} values for multiple PARP inhibitors (Response Figure 17B). These results suggest that *TRIM21* mRNA level may not serve as a reliable biomarker for predicting chemotherapy or PARPi sensitivity in SCLC cells.

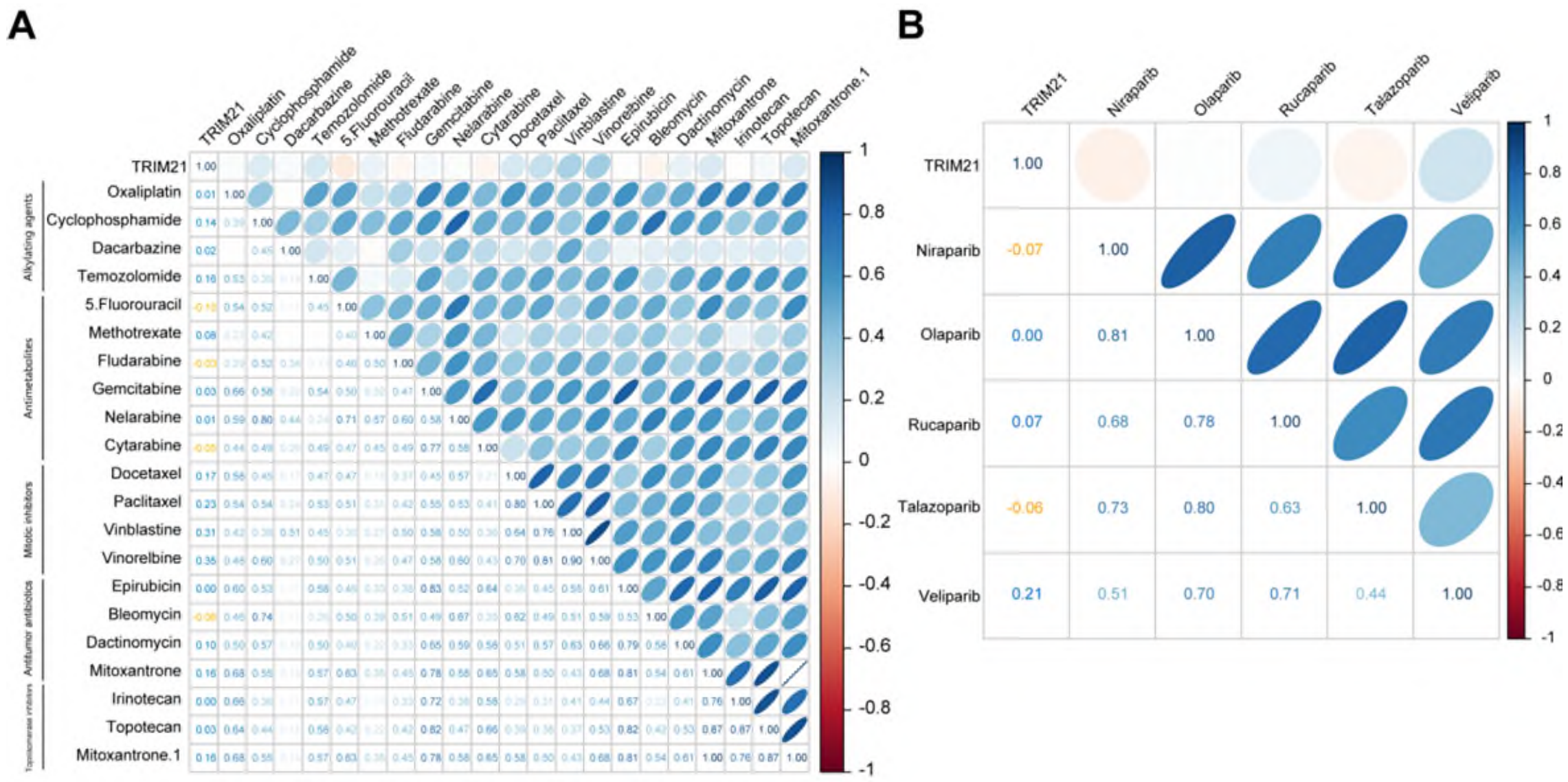

Response Figure 17

Response Figure 17. **A**) Correlation analysis between *TRIM21* mRNA levels and IC_{50} values of various chemotherapeutic agents (alkylating agents, antimetabolites, mitotic inhibitors, antitumor antibiotics, and topoisomerase inhibitors) using the GDSC2 dataset. **B**) Similar correlation analysis evaluating the relationship between *TRIM21* mRNA expression and IC_{50} values of multiple PARP inhibitors.

Reviewer #3 (Remarks to the Author):

I co-reviewed this manuscript with one of the reviewers who provided the listed reports. This is part of the Nature Communications initiative to facilitate training in peer review and to provide appropriate recognition for Early Career Researchers who co-review manuscripts.

Response: We appreciate Reviewer #3's thoughtful participation in evaluating our manuscript.

Reviewer #4 (Remarks to the Author):

The paper by Guozhen Cao and colleagues entitled "TRIM21-mediated ubiquitination of PARP1 suppresses small cell lung cancer (SCLC) progression through the PI3K/AKT/STAT5A axis" explores the connection between STAT5A repressing directly the TRIM21 locus, via PI3K-AKT-mTOR signalling. The authors show mainly with cell line work that STAT5A binding to the promoter of TRIM21 promotes PARP1 stabilization through downregulation of TRIM21 via STAT5A transcriptional repression action. The paper overall is focused on SCLC, but several angles used to demonstrate mechanistic questions, e.g. questionable model cell systems for this cancer, no genetic appropriate controls, or shortcomings in the mechanism of STAT5A or STAT5B or both acting repressive or why a sole repressor function of STAT5A downstream of the PI3K-AKT-mTOR pathway should exist leave space for improvement or study design work flow?!

Response: We appreciate your interest in our study. We have addressed your concerns by performing comprehensive authentication and verification of the SCLC cell lines (DMS273 and SHP77) used in this study, including STR profiling, to ensure their reliability and validity for all reported experiments. Additionally, we conducted a series of new experiments that demonstrated that while STAT5B is transcriptionally regulated by the PI3K-AKT signaling pathway, it does not function as a transcriptional factor for *TRIM21*. Please refer to our point-by-point responses below for detailed addressing of each concern.

It was e.g. described in the literature that STAT5 interacts with the p85 subunit of PI3K and it can transcribe AKT isoforms, as well as it is phosphorylated by mTOR, ignored by authors.

Response: We fully acknowledge the reviewer's important point regarding the well-documented interactions between STAT5 and the PI3K-AKT-mTOR pathway, including STAT5's binding to the p85 subunit of PI3K, its role in transcribing AKT isoforms, and its phosphorylation by mTOR. While these established mechanisms were not the primary focus of our current study, our experimental data demonstrated that inhibition of PI3K-AKT signaling (PKI-587 treatment) significantly increases both STAT5A transcription and protein levels, whereas pathway activation (SC79 treatment) decreases them (Figure 7G and S7I). Similarly, overexpression of AKT, particularly its constitutively active form, but not the inactive variant, suppressed STAT5A, leading to subsequent downregulation of TRIM21 expression (Figure 7K). Furthermore, our newly added data from previous studies confirm

that treatment with a PI3K/AKT pathway inhibitor upregulates STAT5A transcript levels in SCLC (Figure S8C). These results position STAT5A as a downstream effector whose transcription is negatively regulated by the PI3K-AKT signaling in our system.

The integration of our findings with the literature cited by the reviewer suggests that the existence of a sophisticated and potentially bidirectional regulatory crosstalk between STAT5 and PI3K-AKT signaling, which may involve previously unrecognized feedback mechanisms. Furthermore, the interplay between STAT5 and PI3K-AKT pathways may exhibit tumor-specific characteristics. We agree that this represents a fascinating biological phenomenon worthy of future investigation, and we have accordingly expanded our discussion of this point in the revised manuscript (Lines 479-488) to better contextualize our findings within the broader understanding of the crosstalk between PI3K-AKT and STAT5A suppression. We have also cited the references suggested by the reviewer (refs: 53, 54).

Here, authors describe a negative action via the PI3K-AKT-mTOR pathway using STAT5A only to repress TRIM21, all data presented are a bit selectively due to different model character. One can view the study design to particularly build a case in a neuroendocrine progenitor-driven tumor, that represents a bit more then 10% of all lung cancers. However, as the story is presented, literature incorporated, or mechanism explored, this paper leaves too many questions and holes and it is immature in its current format for publication in Nature Communications. Main points with multiple subpoints emphasis this as follows:

Response: We are deeply grateful for the reviewer's thorough evaluation of our manuscript and their constructive suggestions. We have performed comprehensive additional experiments and provided substantial new evidence to validate our conclusions. These include: 1) definitive clarification that STAT5A (but not STAT5B) is transcriptionally regulated by the PI3K-AKT signaling to control TRIM21 expression (Response Figure 21 and 22); 2) experimental confirmation of STAT5A nuclear localization in our SCLC model systems (DMS273 and SHP77 cells) (Response Figure 20); and 3) validation of our proposed molecular mechanism using multiple independent SCLC patient-derived xenograft (PDX) models (Response Figure 18). The new data have not only strengthened our central findings but also provided deeper mechanistic insights that address the reviewer's concerns about

pathway specificity and biological relevance. Detailed experimental results addressing these specific concerns are presented in our point-by-point responses below.

We believe that our comprehensive responses and substantially revised manuscript have significantly enhanced the quality of this work. The reviewer's expert insights have been invaluable in helping us develop a more rigorous and compelling story that we expect will make a meaningful contribution to the SCLC research field.

1) The team used a limited and selected set of cell lines for their study that was not appropriately introduced for cancer genome landscape information what are the key drivers in these cell lines and their origin. A closer look reveals that only one cell line represents the study focus SCLC and five of six are quite different, leaving a selected presentation of data, not explained why these different cancer cell origins should resemble a conclusive set of patient derived model cell systems. No primary lung cancer purified cell samples were extracted to back up data or the lack of PDX or alternatively organoid models of SCLC which exist are further limitations. The selected cell lines used display even mutations affecting the axis to be described distinctly, not introduced or discussed. Thus, the selection and representation of work related to these models can be regarded as inappropriate. Conclusions drawn can lead to false interpretations of results. The authors need to validate in a genetic model of SCLC their key findings and PDX models exist or other relevant samples such as primary specimens, which were all not incorporated, representing a problematic model work. Why the representation of the cell lines were done and why they were not introduced and described is far from optimal and too selected. The authors need to do a better work to demonstrate that their models represent a valid genetic model of SCLC, e.g. establishing paired model cell line systems of human origin. Here, also CRISPR-cas9 paired cell line deletion or after deletion add back was not performed to e.g. assemble three cell lines of one small cell lung cancer patient. Instead, authors did use knockdown, but omitted add back of the gene of interest to control for off-target effects of any sh- or si-RNA interference work. All that leaves room for improvements. A small survey on the cell lines reveals the following:

From the six tumour cell lines used for the study DMS273 which is also called DMS 273 and was isolated in 1978 from a patient with SCLC, is the closest model. However, it was isolated via the pleural fluid specimen from a 50-year-old female patient decades ago and it can drift away from the study subject tumour. The patient was in relapse after chemotherapy and radiotherapy, where cells were reported in Cellosaurs to carry a TP53 and NPM1 mutation, which is relevant here, e.g. both interact with STAT proteins and NPM1 steers import and export into nucleus of STATs hanging together with BRCA1/2/BARD complex at the nuclear pore.

PC9 which is also called in Cellosaurus PC-9 represents a human adenocarcinoma from lung tissue which remains differentiated, but it is not a SCLC model. It carryies TP53 mutation and two EGFR mutations, the later

phosphorylates STAT5 at multiple alternative tyrosine phosphorylation sites, of unknown biological consequence, or pYSTAT1/3 as well......

SHP77 which is also called SHP-77 are epithelial cells are derived from a non-encapsulated primary lung tumor from the apical portion of the upper lobe of the left lung of a 54-year-old, again not SCLC model. It was isolated from a Caucasian male in 1977. Cellosaurus documents KRAS, TP53, RAC1 and ABL1 mutations. Again, ABL-mediated tyrosine phosphorylation and nuclear import of STAT5 complexing with RAC1 for nuclear import is affected as well as mitochondrial function of STAT3/5 via KRAS, all not discussed or introduced, etc.

A549 cells are adenocarcinoma human alveolar basal epithelial cells, again not SCLC as a model and they constitute a cell line developed in 1972 through the removal and culturing of cancerous lung tissue in the explanted tumor of a 58-year-old Caucasian male. Cellosaurus documents KRAS, TP53 and STK11 mutations. See comments above and also below to cellular mutations.

AGS is a cell line exhibiting epithelial morphology that was isolated in 1979 from the stomach tissue of a 54-year-old, White, female patient with gastric adenocarcinoma. Cellosaurus documents KRAS, TP53, CTNNB1 and two PI3KCA mutations. It is questionable if persistent PI3K-AKT-mTOR action would be a good model or not and why should gastric cancer be relevant for studying and concluding on SCLC, also true for next cell line.

MKN45 which is also called MKN-45 cell line is a human gastric cancer cell line, derived from the poorly differentiated adenocarcinoma of the stomach. Cellosaurus documents MET amplification and TP53 mutation. MET growth factor receptor probably also phosphorylates STAT5 at alternative tyrosines similar like EGFR, all not clear. The use of HEK293T cells for mechanistic insight is another distance to SCLC….

Thus, in conclusion the model cell systems used in this study and how authors deal with their introduction and discussion of results are in most cases far away from the tumour in focus or can be called a selected assembly. That is not acceptable to serve as a basis for claims of mechanistic nature in small cell lung cancer studies, where driver mutation status is key to be taken into consideration in multiple model cell character with similar mutation setups of SCLC origin.

Response: Thanks for your comments. In response, we have conducted extensive additional experiments to address each point raised. The specific details are as follows:

1) Validation and characterization of SCLC cell lines

We have re-examined and verified the origins and genetic characteristics of all cell lines used in our study. Our primary SCLC investigations were conducted using two well-characterized SCLC cell lines: DMS273 (RRID: CVCL_1176) and SHP77 (RRID: CVCL_1693), both of which are authenticated as bona fide SCLC models by Cellosaurus (https://www.cellosaurus.org) and ATCC (http://www.atcc.org). These two cell lines have been extensively utilized and validated as reliable

SCLC models in multiple studies[1-5]. We have performed additional cell line authentication (documentation provided in the attachment of revision), and the results perfectly match the STR profiling provided by Cellosaurus, definitively confirming the identity of these cell lines as canonical SCLC models. Furthermore, we characterized the genetic profiles of these two cell lines, with the following key information: DMS273 carries NPM1 (p.Asp173del) and TP53 (p.Gly245Cys) mutations, while SHP77 harbors ABL1 (p.Val1128Glu), KRAS (p.Gly12Val), RAC1 (p.Tyr32Cys), and TP53 (p.Cys176Trp) mutations.

Regarding other lung adenocarcinoma cells (PC9 and A549), gastric cancer cells (AGS and MKN45), and cervical cancer cells (HeLa), we clarify that these were used solely in Original Figure 2C to illustrate the pan-cancer inverse correlation between TRIM21 and PARP1 expression levels. These cells were not subjected to further mechanistic investigations in this study. Additionally, HEK293 cells were only utilized for studying the molecular details of TRIM21-mediated PARP1 ubiquitination. We believe this clarification adequately addresses the reviewer's concerns about model system selection and provides full transparency regarding the cellular contexts in evaluating the mechanism by which TRIM21-mediated ubiquitination of PARP1 by the PI3K-AKT-STAT5A axis.

References

1. Ghandi M, et al. Next-generation characterization of the Cancer Cell Line Encyclopedia. Nature 569, 503-508 (2019).
2. Goncalves E, et al. Pan-cancer proteomic map of 949 human cell lines. Cancer Cell 40, 835-849 e838 (2022).
3. Nguyen A, et al. Jumonji histone demethylases are therapeutic targets in small cell lung cancer. Oncogene 43, 2885-2899 (2024).
4. Nusinow DP, et al. Quantitative Proteomics of the Cancer Cell Line Encyclopedia. Cell 180, 387-402.e316 (2020).
5. Polley E, et al. Small Cell Lung Cancer Screen of Oncology Drugs, Investigational Agents, and Gene and microRNA Expression. J Natl Cancer Inst 108, (2016).

2) Validation of key findings using SCLC PDX models

We have performed additional immunohistochemical analyses of 13 SCLC PDX models for the expression levels of phosphorylated AKT (p-AKT), phosphorylated STAT5A (p-STAT5A), TRIM21, PARP1, and γH2AX. The results demonstrated that p-AKT and PARP1 consistently show high expression levels, while p-STAT5A, TRIM21, and γH2AX generally exhibit low expression patterns in these PDX models (Response Figure 20A, designated as Supplementary Figure S8J in revision).

Furthermore, negative correlations were observed between p-AKT and p-STAT5A, as well as between p-AKT and TRIM21. In contrast, a positive correlation was found between p-STAT5A and TRIM21, whereas TRIM21 and PARP1 were negatively correlated (Response Figure 20A, designated as Figure 8A in revision). These contents have been incorporated into the revised manuscript (Lines 365-374 and 1355-1357). These findings robustly validate our proposed mechanistic model that "PI3K/AKT signaling suppresses TRIM21 transcription through the transcription factor STAT5A, thereby reducing TRIM21-mediated PARP1 ubiquitination and degradation. This leads to PARP1 accumulation and subsequent maintenance of genomic stability."

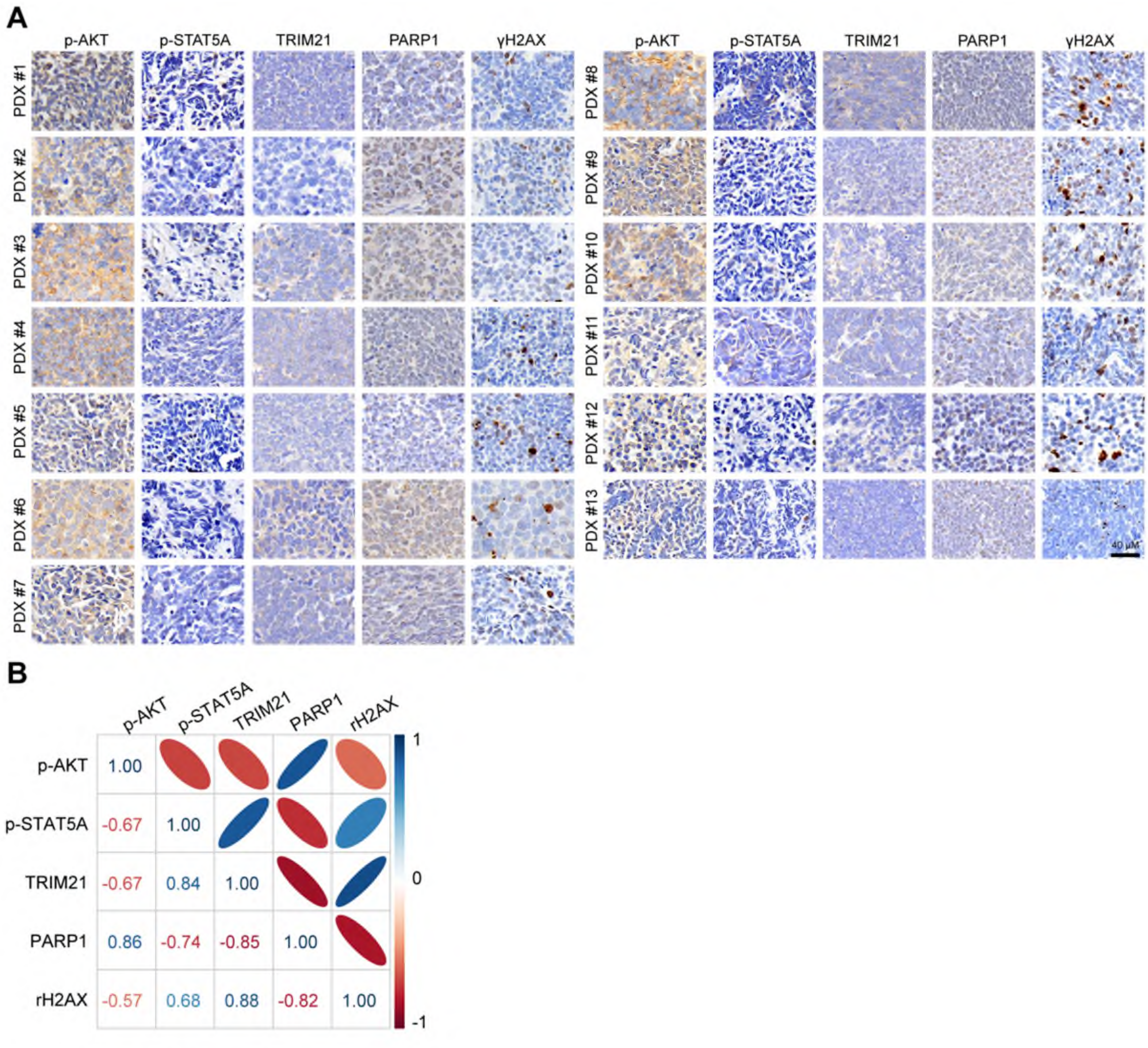

Response Figure 18

Response Figure 18. **A**) IHC profiling of SCLC PDX models. 13 patient-derived xenograft (PDX) models (PDX #1-#13) were analyzed by IHC for key biomarkers: p-AKT, p-STAT5A, TRIM21, PARP1, and γH2AX. Scale bars, 40 μm. **B**) Correlation analysis of expression levels of p-AKT, p-STAT5A, TRIM21, PARP1, and γH2AX in SCLC PDX tissues.

3) Rescue experiments following *STAT5A* knockdown in SCLC cells

We fully agree with the reviewer's insightful suggestion about the critical need for *STAT5A* rescue experiments in *STAT5A*-knockdown cells. Following this recommendation, we have conducted stable *STAT5A* knockdown followed by *STAT5A* reconstitution in SCLC cells to exclude potential off-target effects. The results showed that *STAT5A* knockdown significantly reduces TRIM21 mRNA and protein expression levels, consequently weakening TRIM21-mediated PARP1 ubiquitination and degradation, which leads to increased PARP1 accumulation. Importantly, reintroduction of *STAT5A* in the knockdown cells effectively reverses these changes. Notably, the alterations in *STAT5A* expression levels do not affect *PARP1* transcriptional levels (Response Figure 19A, B, designated as Supplementary Figure S7J and Figure 7I in revision). These contents have been incorporated into the revised manuscript (Lines 335-338 and 1342-1345). These comprehensive validation experiments provide robust support for our central findings and the mechanistic model.

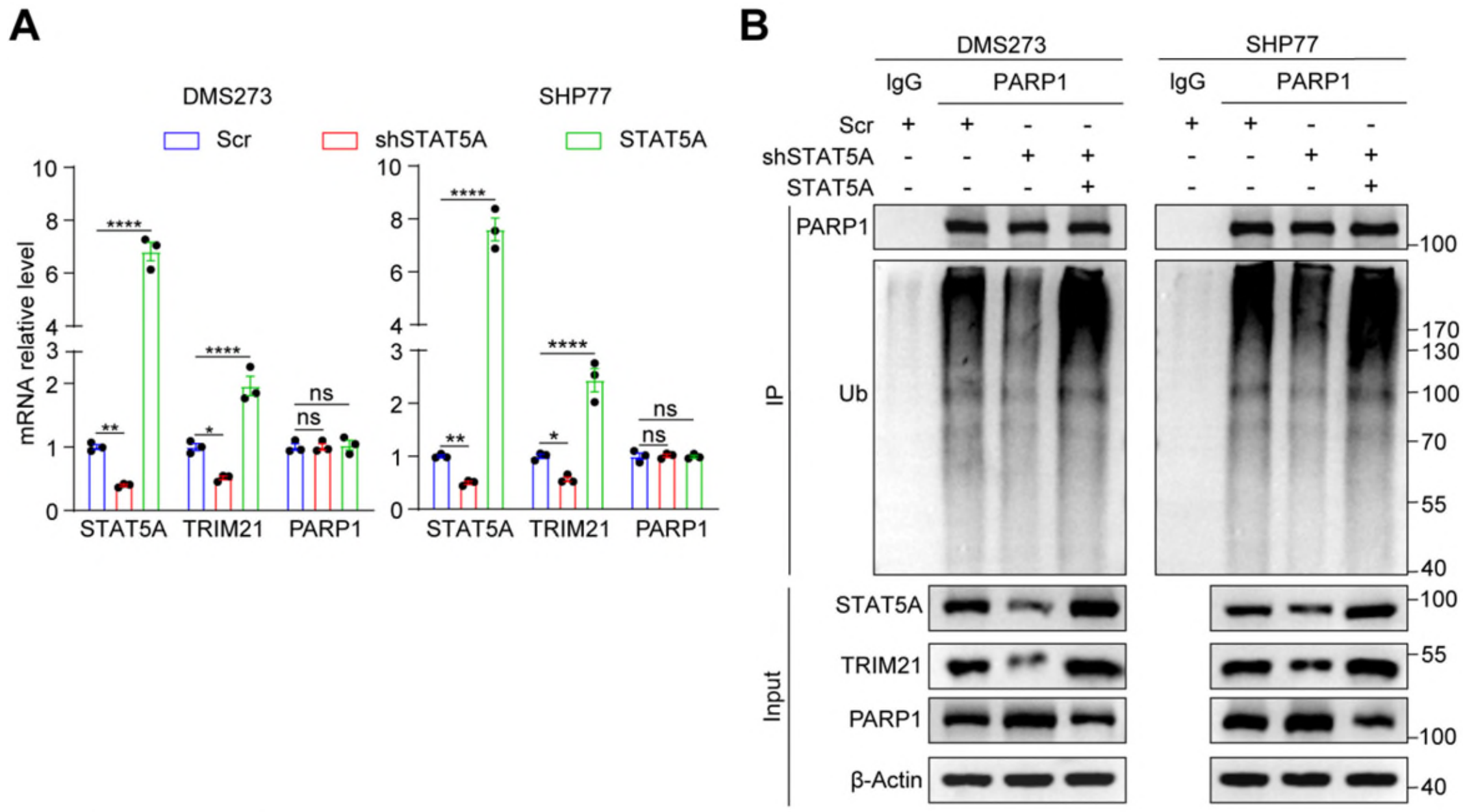

Response Figure 19

Response Figure 19. **A**) Analysis of *STAT5A*, *TRIM21*, and *PARP1* mRNA expression levels. DMS273 and SHP77 cells were stably transfected with *STAT5A* knockdown (sh*STAT5A*), followed by *STAT5A* overexpression rescue (STAT5A). The transcriptional levels of *STAT5A*, *TRIM21*, and *PARP1* were then measured by RT-qPCR using β-Actin as the internal control. Data are represented as mean ± SEM, and were statistically analyzed using the two-tailed unpaired Student's *t*-test. ns, no significance; *P < 0.05, **P < 0.01, ****P < 0.0001. **B**) IP-Western blot analysis of key proteins indicated and the ubiquitination level of PARP1. Cells were treated as described in (**A**). PARP1 was immunoprecipitated and analyzed by Western blot to detect the levels of accumulation and polyubiquitination.

4) Validation of STAT5A nuclear localization

As noted by the reviewer, the TP53 and NPM1 mutations in DMS273, as well as the KRAS, TP53, RAC1, and ABL1 mutations in SHP77, could potentially affect STAT protein nuclear import in these SCLC cells. To directly assess whether STAT5A localizes to the nucleus in these models, we performed comprehensive analyses using immunofluorescence assays and nuclear-cytoplasmic fractionation experiments under both PI3K/AKT pathway inhibition and activation conditions. Our results demonstrated that under basal conditions (without drug treatment), STAT5A is distributed in both the nucleus and cytoplasm, but predominantly localized in the nucleus of both DMS273 and SHP77 cells (Response Figure 20A, B), confirming that STAT5A nuclear import remains functionally intact in these cell lines despite their distinct mutational backgrounds.

Importantly, inhibition of the PI3K/AKT pathway by PKI-587 increased STAT5A levels much more in the nuclear fractions than the cytoplasmic fractions, while PI3K/AKT pathway activation (using the activator SC79) reduced STAT5A expression in both compartments (Response Figure 20A, B). These findings provide compelling evidence that the PI3K/AKT pathway regulates STAT5A nuclear localization in DMS273 and SHP77 cell lines.

In summary, our supplemental experiments robustly demonstrate that despite the presence of various mutations in SCLC cells, the specific mutational profiles of DMS273 and SHP77 do not compromise our core finding that the PI3K/AKT signaling suppresses TRIM21 transcription through regulation of STAT5A.

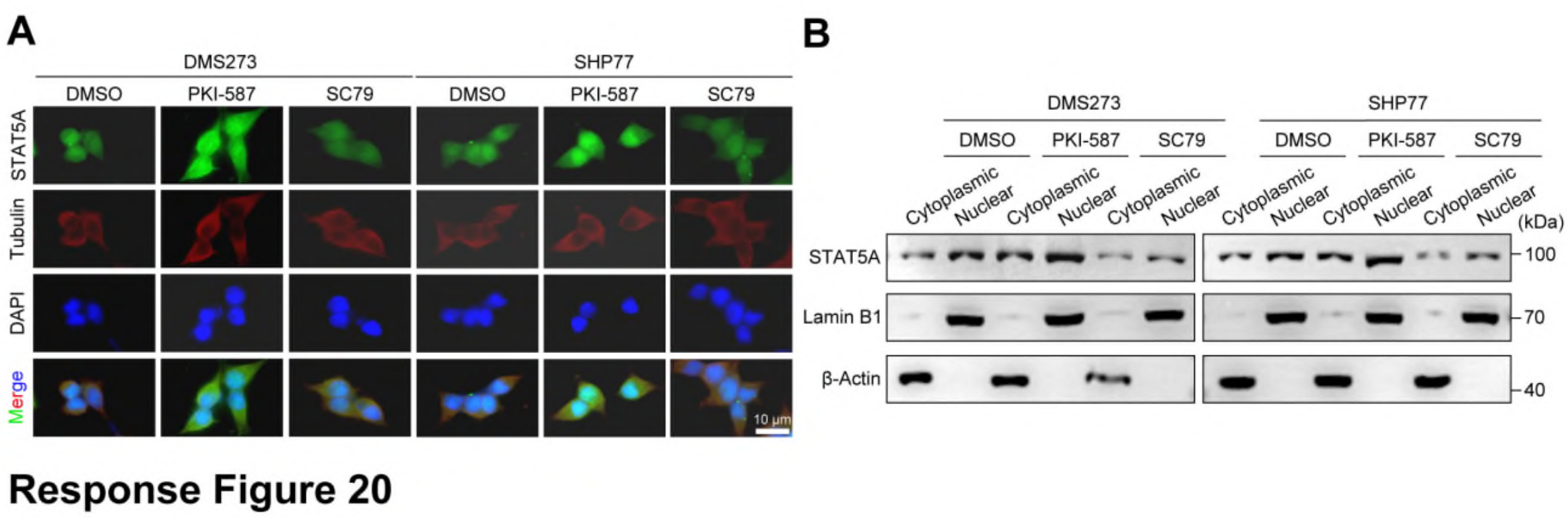

Response Figure 20. **A**) Nuclear translocation of STAT5A in DMS273 and SHP77 cells treated with 1 μM PKI-587 (24 h) or 5 μM SC79 (24 h) was analyzed by immunofluorescence. Scale bar, 30 μm. **B**) Subcellular localization analysis of STAT5A. Cells were treated as in (**A**), followed by nuclear/cytoplasmic fractionation and Western blot detection of STAT5A levels in each fraction.

2) STAT5 is encoded by two different gene products, STAT5A and STAT5B, both binding to an inverted palindromic sequence. The question if STAT5B follows the same trait and rules is important and both STAT5 proteins make a heterodimer, but STAT5B is more oncogenic. STAT5A is a balancer with shorter half life over STAT5B action, authors ignored expression or activity of STAT5B in SCLC. Both bind palindromic sequences, but STAT5B is in particular more versatile for their spacing for 2 up to 4 bps within the palindromic sequence that authors displayed in Sup. Fug. 7, but this might be a comprehensive bioinformatics way of showing potential binding sites. A reporter construct with these sites and identification of corepressor complex with NCOR or BCOR components being described to bind to STAT5 is feasible. However, if true more sequences could be expected in the large loci used for analysis, promoter or enhancer as well as exom intron structure of the TRIM21 locus have to be illustrated better. The whole transcriptional relevant elements of the TRIM21 locus need to be displayed and marked for potential binding sites of the STAT family and it might not only be STAT5 that then is relevant. E.g. in a simple sequence file with marked binding site analysis also validated by ChIP for both STAT5A or STAT5B and for control purposes also for STAT3 ChIP or STAT1 ChIP and monitoring of their mRNA and protein expression is key here. Authors should show these sites as single sites, since that allows judgment in a more meaningful manner. Interspecies conservation should be displayed e.g. is it conserved in mice, rat, canine and e.g. chimpanzee? Binding site conservation if existent in a more general manner manifests the transcriptional mode of action for different species as model system, e.g. is there a species that is more vulnerable to SCLC then how about that conservation? These binding sequences can also be bound by BCL6, a STAT5 or STAT3 locus repressor in a YIN/YANG fashion. Here, BCL6 expression monitoring would be important. STAT5A can be expressed via TP53 action, but not STAT5B, whereas both interact with TP53. Most model cell systems are TP53 mutated making mechanistic work here in that regard unclear due to the many functions that mutated TP53 still has, genetic Null mutation for TP53 and a better separation into driver mutation clusters would be key here. Biallelic inactivation of TP53 and RB1 is nearly universal in SCLC cases and indeed, some 20 years ago also a GEMM was made with both their deletions genetically. Normally, wt TP53 can repress STAT5 mediated transcription as was shown in MPN and AML model cell systems, but mutated TP53 can boost STAT5-mediated transcription. Last, STAT3 and STAT1 action need to be controlled and taken into consideration, since they are interwoven on chromatin action or transcriptional regulation in cancer cells with STAT5 mode of action. Pharmacologic intervention with selective STAT5 inhibitors would also be appropriate and such tools exist. No genetic model of STAT5 deletion with SCLC was made as further limitation of the study backing up claims.

Response: We are grateful for the reviewer's valuable and thoughtful suggestions. Below, we provide point-by-point responses with new experimental evidence addressing these important concerns.

1) The PI3K-AKT pathway also inhibits the expression level of *STAT5B*

The reviewer inquired whether PI3K-AKT signaling specifically represses *STAT5A* or exerts similar effects on *STAT5B*. Our additional experiments demonstrated that PI3K-AKT pathway inhibition (PKI-587 treatment) significantly increases STAT5B transcription and protein levels, while PI3K-AKT pathway activation (SC79 treatment) decreases them (Response Figure 21A, B, designated as Supplementary Figure S8A, B in revision), mirroring our previous findings with STAT5A. Interestingly, analysis of previously published data on STAT family members' expression following PI3K/AKT inhibitor PIK-75 treatment revealed that PI3K/AKT inhibition significantly upregulates the expression levels of *STAT5A* and *STAT5B* in SCLC (Response Figure 21C, designated as Supplementary Figure S8C in revision), which is consistent with our findings. Moreover, clinical correlation analysis further supports this relationship, showing a significant negative association between PI3K-AKT pathway activity and *STAT5B* expression in SCLC tissues (Response Figure 21D, designated as Supplementary Figure S8D in revision). These results have been incorporated into the revised manuscript (Lines 347-354). Collectively, these data elucidate that PI3K-AKT transcriptionally suppresses both *STAT5A* and *STAT5B*.

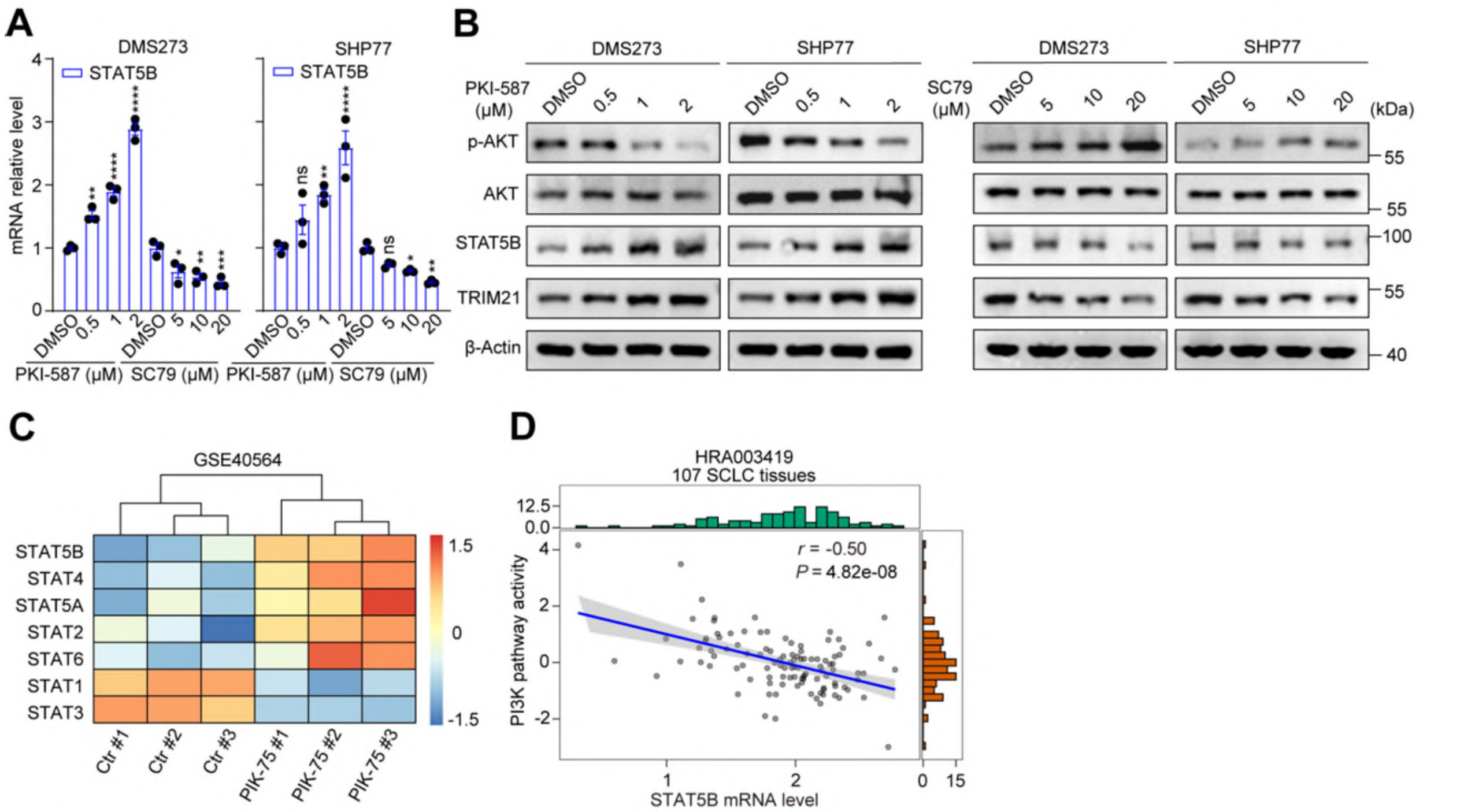

Response Figure 21

Response Figure 21. **A**) RT-qPCR analysis of *STAT5B* expression in DMS273 and SHP77 cells treated with a concentration gradient of PKI-587 or SC-79 for 24 h. **B**) Western blot analysis of p-AKT, AKT, STAT5B, and TRIM21 in DMS273 and SHP77 cells following treatment with PKI-587 or SC-79 for 24 h. **C**) Heatmap of STAT family members expression (*STAT1*, *STAT2*, *STAT3*, *STAT4*, *STAT5A/B*, and *STAT6*) from RNA-seq data (GSE40564 dataset) in cells treated with the PI3K/AKT inhibitor PIK-75. **D**) Correlation analysis between PI3K/AKT pathway

activity scores and *STAT5B* mRNA levels in 107 SCLC tissues (HRA003419 database). Data are represented as mean ± SEM and were statistically analyzed using the two-tailed unpaired Student's *t*-test. ns, no significance, **P* < 0.05, ***P* < 0.01, ****P* < 0.001, *****P* < 0.0001.

2) STAT5B is not a transcriptional regulator of *TRIM21*

We have conducted a systematic investigation into whether STAT5B functions as a transcriptional regulator of *TRIM21*. Our new results showed that *STAT5B* knockdown does not affect TRIM21 expression at either mRNA or protein levels (Response Figure 22A, B, designated as Supplementary Figure S8E, F in revision), and no correlation exists between *STAT5B* and *TRIM21* expression levels in SCLC clinical samples (Response Figure 22C, D, designated as Supplementary Figure S8G in revision). Most conclusively, interrogation of ChIP-seq data and subsequent ChIP-PCR analysis demonstrated that STAT5B does not bind the *TRIM21* promoter region (Response Figure 22E, F, designated as Supplementary Figure S8H, I in revision). These results have been incorporated into the revised manuscript (Lines 354-363). Thus, integrating our initial findings on STAT5A-mediated regulation of *TRIM21* transcription with the results presented above in section "1)", we conclude that while PI3K-AKT suppresses the transcriptional level of both *STAT5A* and *STAT5B*, only STAT5A functions as a transcription factor of *TRIM21*.

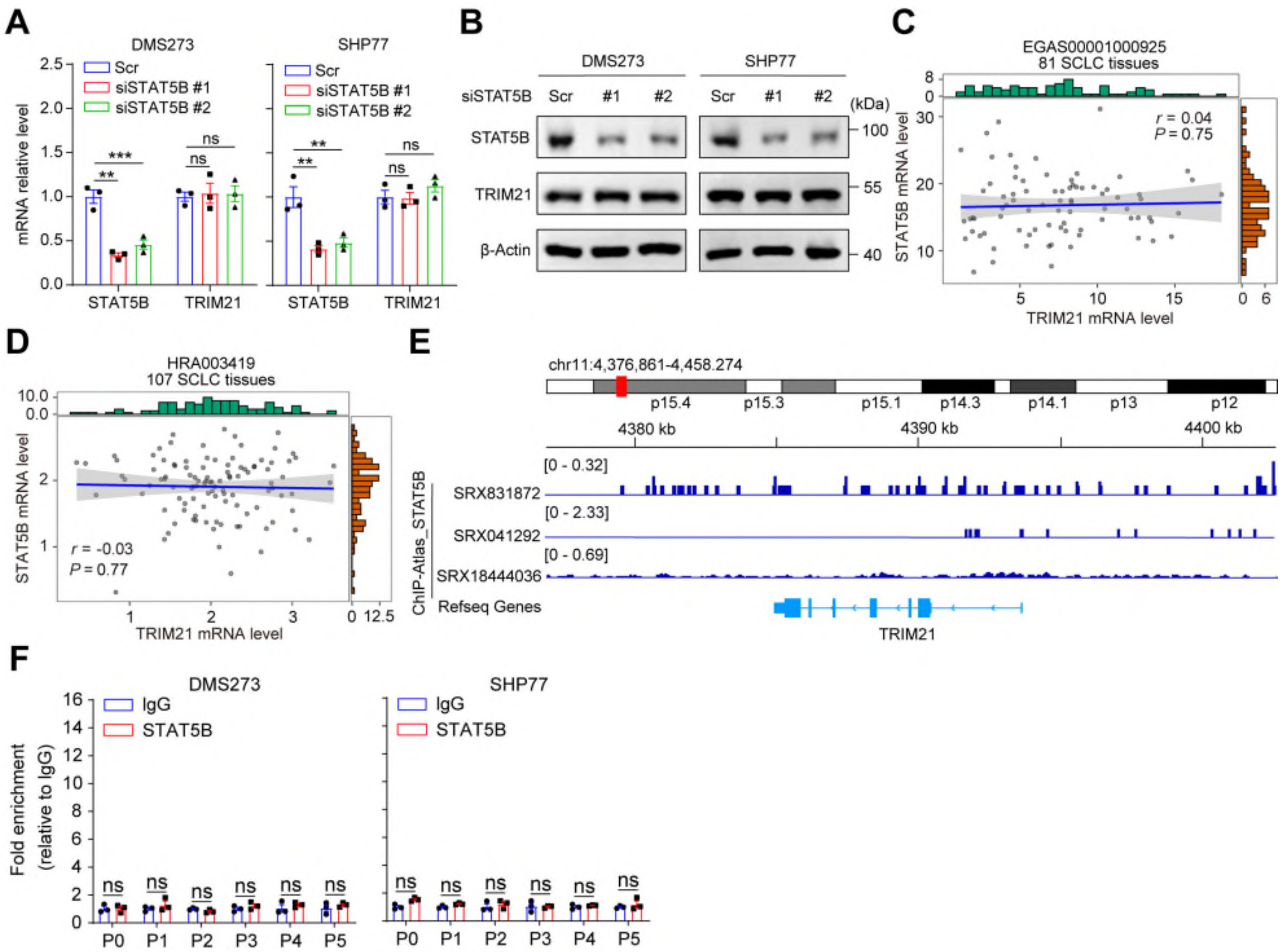

Response Figure 22

Response Figure 22. **A**, **B**) RT-qPCR and Western blot analyses of STAT5B and TRIM21 expression following *STAT5B* knockdown (si*STAT5B*). The mRNA (**A**) and protein levels (**B**) of STAT5B and TRIM21 were measured. **C**, **D**) Correlation analysis between PI3K/AKT pathway activity scores and *STAT5B* mRNA levels in 81 SCLC tissues (EGAS00001000925 database) and 107 SCLC tissues (HRA003419 database). **E**) Identification of STAT5B peaks in the promoter of the *TRIM21* gene from the ENCODE (Encyclopedia of DNA Elements) database. **F**) STAT5B did not bind to the *TRIM21* promoter assessed by ChIP-qPCR in DMS273 and SHP77 cells. Data represent mean ± SEM and were statistically analyzed using the two-tailed unpaired Student's *t*-test. ns, no significance, $^{**}P < 0.01$, $^{***}P < 0.001$.

3) Luciferase reporter validation of STAT5A binding

Following the reviewer's suggestion, we developed a dual-luciferase reporter system containing the *TRIM21* promoter. Co-transfection with the *STAT5A* overexpression vector in HEK293T cells significantly increased luciferase activity (Response Figure 23A, designated as Figure 7E in revision), directly confirming that STAT5A binds to the *TRIM21* promoter and activates *TRIM21* transcriptional activity. This result has been incorporated into the revised manuscript (Lines 320-324).

Furthermore, we examined the potential roles of corepressor complex NCOR and BCOR components in modulating STAT5A binding to the *TRIM21* promoter, revealing similar functions for these factors. Genetic depletion experiments demonstrated that neither *BCOR* nor *NCOR* knockdown affected STAT5A's binding affinity for the *TRIM21* promoter (Response Figure 23B). These findings suggest that NCOR and BCOR do not normally function to suppress STAT5A-mediated transcriptional activation of *TRIM21* in SCLC.

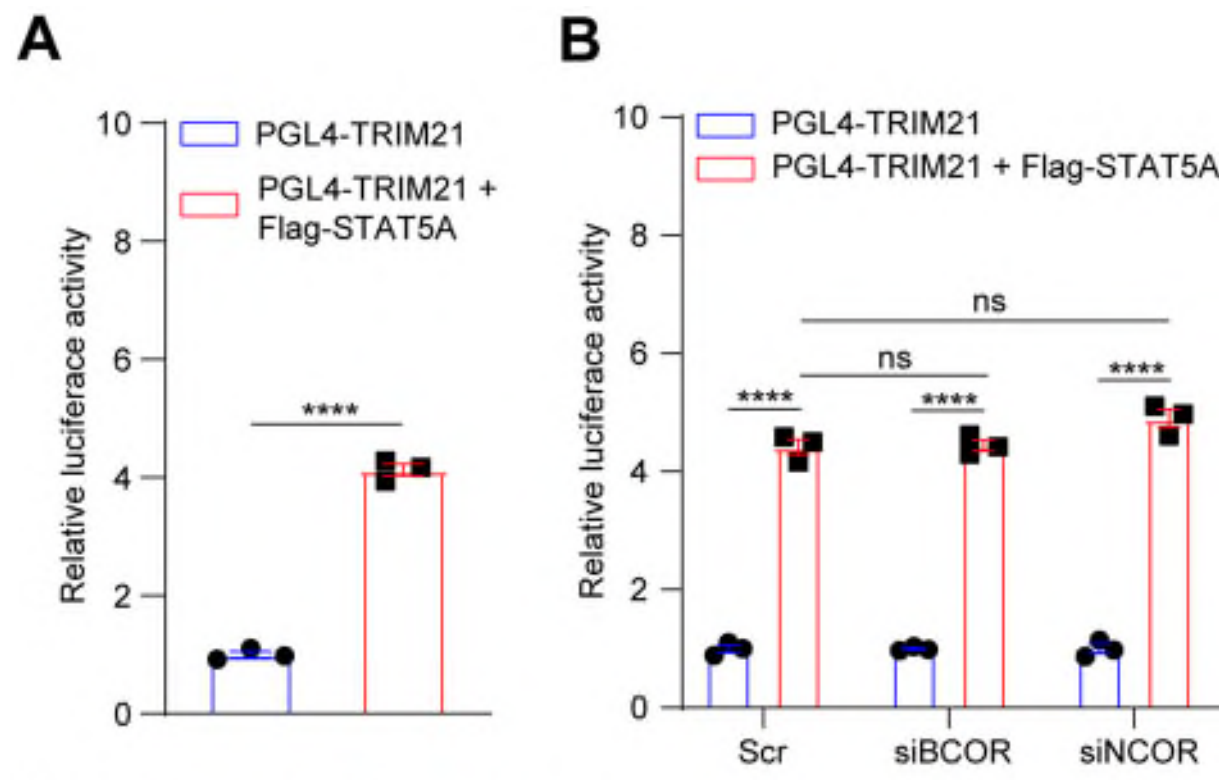

Response Figure 23

Response Figure 23. **A**) Dual-luciferase reporter assay for STAT5A binding to the *TRIM21* promoter. The *TRIM21* promoter sequence was cloned into the PGL4 vector (PGL4-*TRIM21*) and co-transfected with the *STAT5A* overexpression vector (Flag-*STAT5A*) into HEK293T cells. Relative luciferase activity was measured 48 hours post-transfection, with Renilla luciferase (rLuc) as internal control. **B**) Dual-luciferase reporter assay evaluating NCOR and BCOR effects on STAT5A binding *TRIM21* promoter. HEK293T cells were co-transfected with si*NCOR* or si*BCOR*, PGL4-*TRIM21*, and Flag-*STAT5A*. Relative luciferase activity was measured after 48 hours. Data represent mean ± SEM and were statistically analyzed using the two-tailed unpaired Student's *t*-test. ns, no significance, ****$P < 0.0001$.

4) STAT family binding landscape at the *TRIM21* locus

Our analysis of publicly available ENCODE ChIP-seq data revealed differential binding patterns of STAT family members at the *TRIM21* promoter region. While STAT5A demonstrated strong binding, we also detected significant occupancy by STAT1, STAT2, and STAT3 near the transcription start site, suggesting potential functional redundancy among these STAT members in *TRIM21* regulation (Response Figure 24A). In contrast, STAT4, STAT5B, and STAT6 showed minimal binding affinity, mirroring the negative results we observed for STAT5B by ChIP-PCR (Response Figure 22F). These findings elucidate that multiple STAT family members can bind to the *TRIM21* promoter region and potentially regulate its transcriptional activity. While our current study has established STAT5A as a key regulator of *TRIM21* in SCLC, the functional significance of other STAT family transcription

factors (particularly STAT1, STAT2, and STAT3) in modulating *TRIM21* expression under different cellular contexts or tumor types presents an intriguing biological question that warrants further investigation.

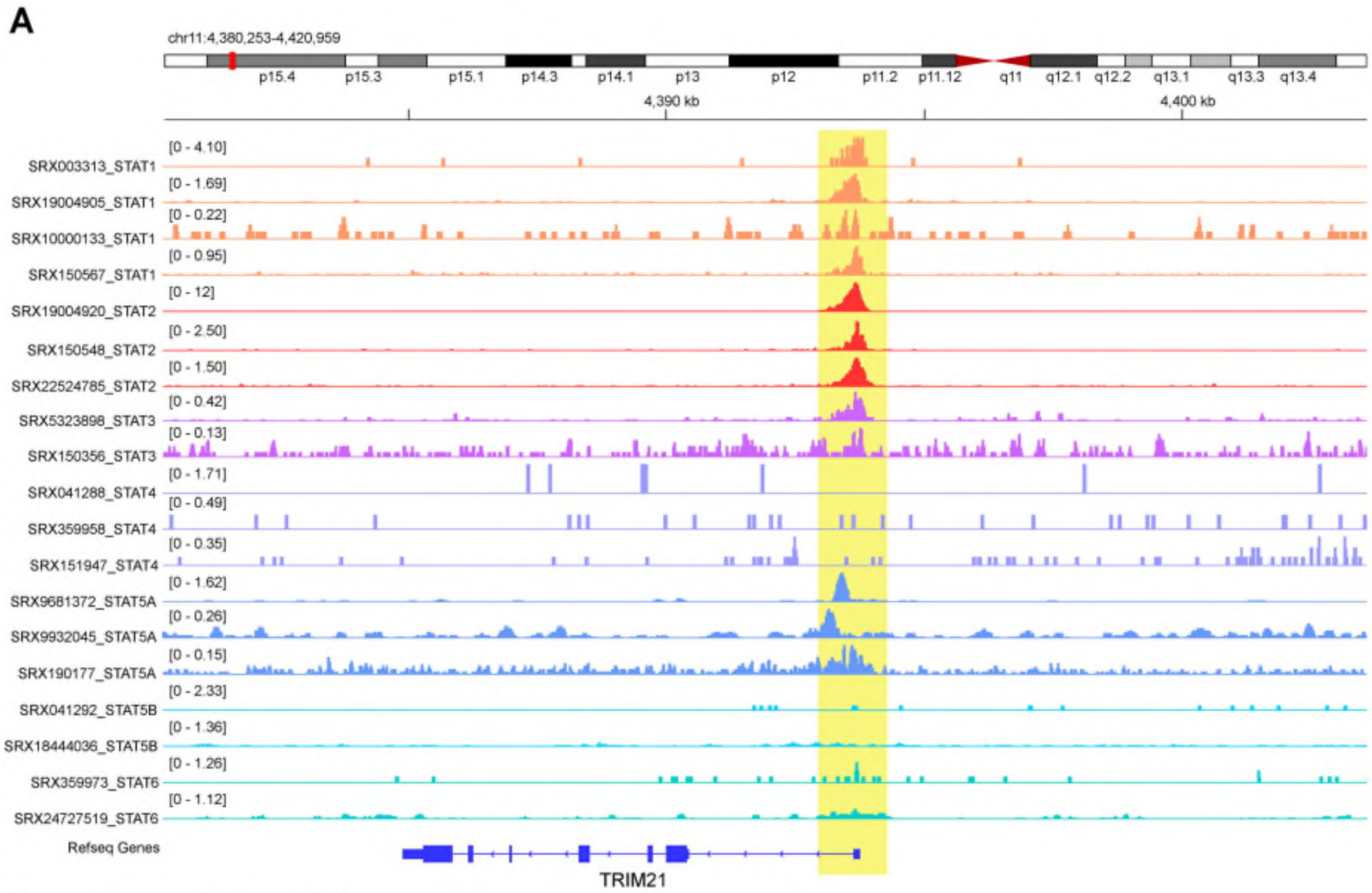

Response Figure 24

Response Figure 24. **A**) Binding profiles of STAT family proteins at the *TRIM21* gene locus. ChIP-seq data from the ENCODE (Encyclopedia of DNA Elements) database were analyzed to determine the binding patterns of STAT1, STAT2, STAT3, STAT4, STAT5A/B, and STAT6 at the *TRIM21* genomic region.

5) Evolutionary conservation of STAT5A binding sites

Our experimental data (Figure 7D and Supplementary Figure S7E) elucidated that STAT5A predominantly binds to a 300bp regulatory region flanking the *TRIM21* transcription start site (TSS), specifically spanning from -200 bp upstream to +100 bp downstream of the TSS. Within this region, bioinformatic analysis using the Eukaryotic Promoter Database identified a high-confidence STAT5A binding motif (STAT5A-motif). Notably, cross-species conservation analysis revealed strong evolutionary preservation of STAT5A-motif (Response Figure 25A). This conserved binding pattern suggests that STAT5A-mediated regulation of *TRIM21* transcription likely represents a fundamental and widely conserved regulatory mechanism across species, rather than being restricted to specific cellular contexts or organisms. The evolutionary conservation of these regulatory elements

also underscores their probable functional importance in maintaining proper *TRIM21* expression levels.

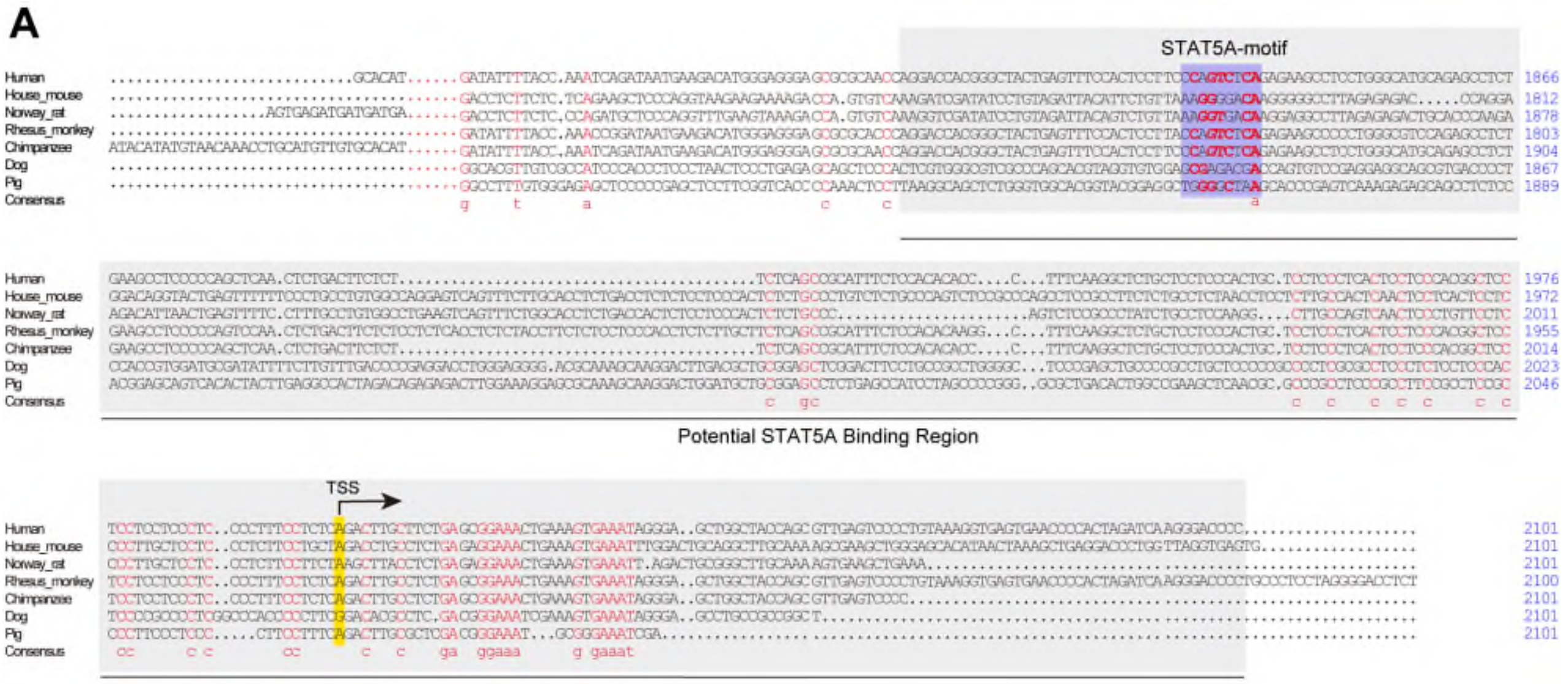

Response Figure 25

Response Figure 25. **A**) Conservation analysis of potential STAT5A binding regions at the *TRIM21* promoter. The STAT5A binding motif (STAT5A-motif) identified through the Eukaryotic Promoter Database represents a conserved sequence for STAT5A binding at the *TRIM21* promoter across multiple species.

6) Function of BCL6

Our investigation into BCL6 function revealed an inverse relationship between PI3K/AKT pathway activity and BCL6 expression, where PI3K/AKT inhibition (PKI-587 treatment) upregulated *BCL6* transcription while pathway activation (SC79 treatment) downregulated it (Response Figure 26A), mirroring the pathway's effect on *STAT5A*. BCL6 deficiency promoted *TRIM21* transcriptional level (Response Figure 26B), suggesting BCL6 may function as a transcriptional repressor of *TRIM21*. Importantly, luciferase reporter assays demonstrated that *BCL6* knockdown significantly enhanced STAT5A binding at the *TRIM21* promoter (Response Figure 26C), implying that BCL6 may suppress STAT5A-mediated transcriptional activation of *TRIM21* in SCLC. These findings reveal a complex regulatory interplay between BCL6 and STAT5A in controlling *TRIM21* expression, with BCL6 potentially serving as a negative modulator of STAT5A-mediated *TRIM21* transcription. The reciprocal regulation between these factors, coupled with their shared responsiveness to the PI3K/AKT signaling, points to a sophisticated transcriptional network that warrants further exploration to fully understand its implications for SCLC biology.

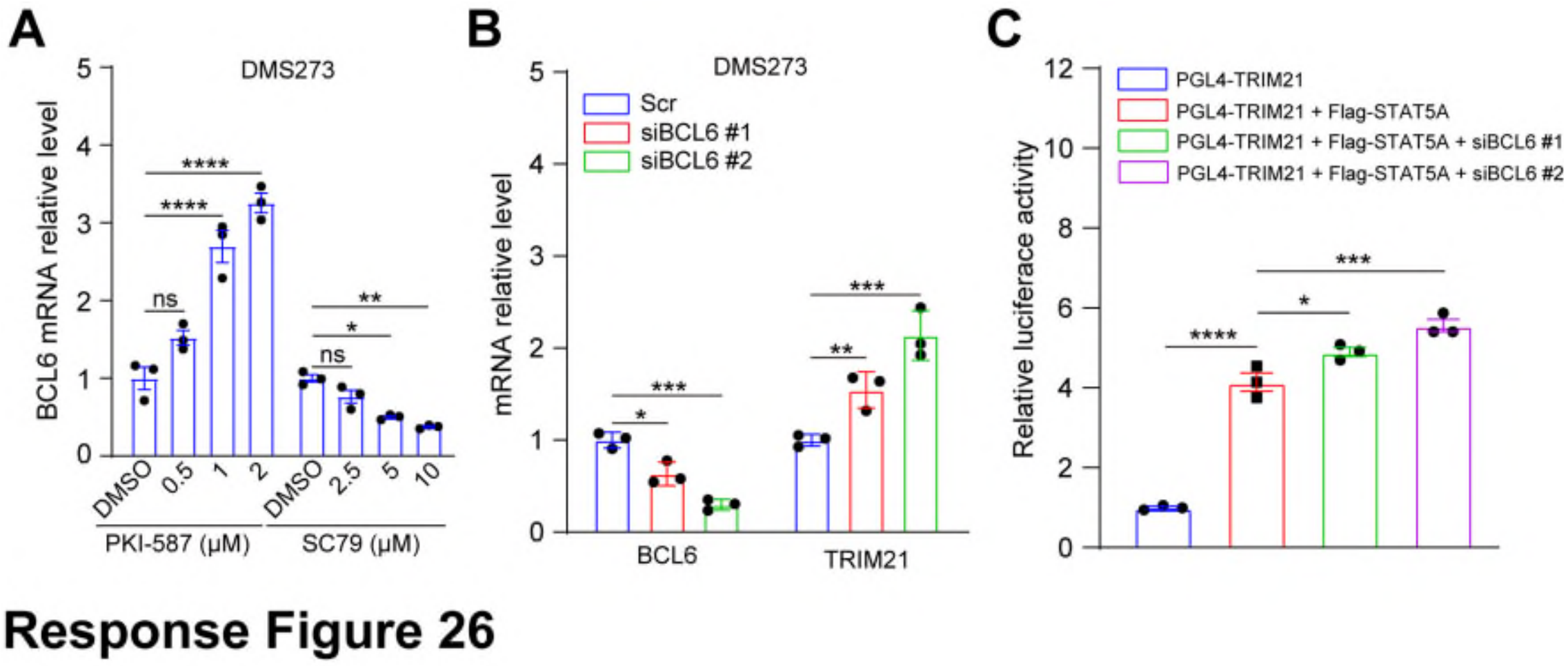

Response Figure 26. **A**) RT-qPCR analysis of *BCL6* expression in DMS273 cells treated with a concentration gradient of PKI-587 or SC-79 for 24 h. **B**) RT-qPCR analysis of *BCL6* and *TRIM21* expression following *BCL6* knockdown (si*BCL6*) in DMS273 cells. **C**) Dual-luciferase reporter assay for STAT5A binding to the *TRIM21* promoter under BCL6-deficient conditions. HEK293T cells were co-transfected with PGL4-*TRIM21*, Flag-*STAT5A*, or si*BCL6*. Relative luciferase activity was measured 48 h post-transfection using Renilla luciferase (rLuc) as an internal control. Data represent mean ± SEM and were statistically analyzed using the two-tailed unpaired Student's *t*-test. ns, no significance, **P* < 0.05; ***P* < 0.01; ****P* < 0.001; *****P* < 0.0001.

7）Effects of STAT5A-targeted inhibitor

Following the reviewer's suggestion, we employed Stafia-1, a selective inhibitor of STAT5A phosphorylation, to assess its impact on STAT5A-mediated transcriptional regulation of *TRIM21*. Our experiments demonstrated that Stafia-1 treatment specifically suppressed STAT5A phosphorylation without affecting its basal expression levels. Importantly, this inhibition of STAT5A activation led to a corresponding decrease in TRIM21 expression and a significant accumulation of PARP1 protein (Response Figure 27A, designated as Supplementary Figure S7K in revision). This result has been incorporated into the revised manuscript (Lines 342-346). These findings provide direct pharmacological evidence supporting our central hypothesis that STAT5A-mediated regulation of TRIM21 governs PARP1 stability in SCLC. The results not only validate our mechanistic model but also suggest potential therapeutic applications of STAT5A inhibitors in SCLC treatment. Through exhaustive experimental validation, we have strengthened these mechanistic conclusions while opening new avenues for investigation.

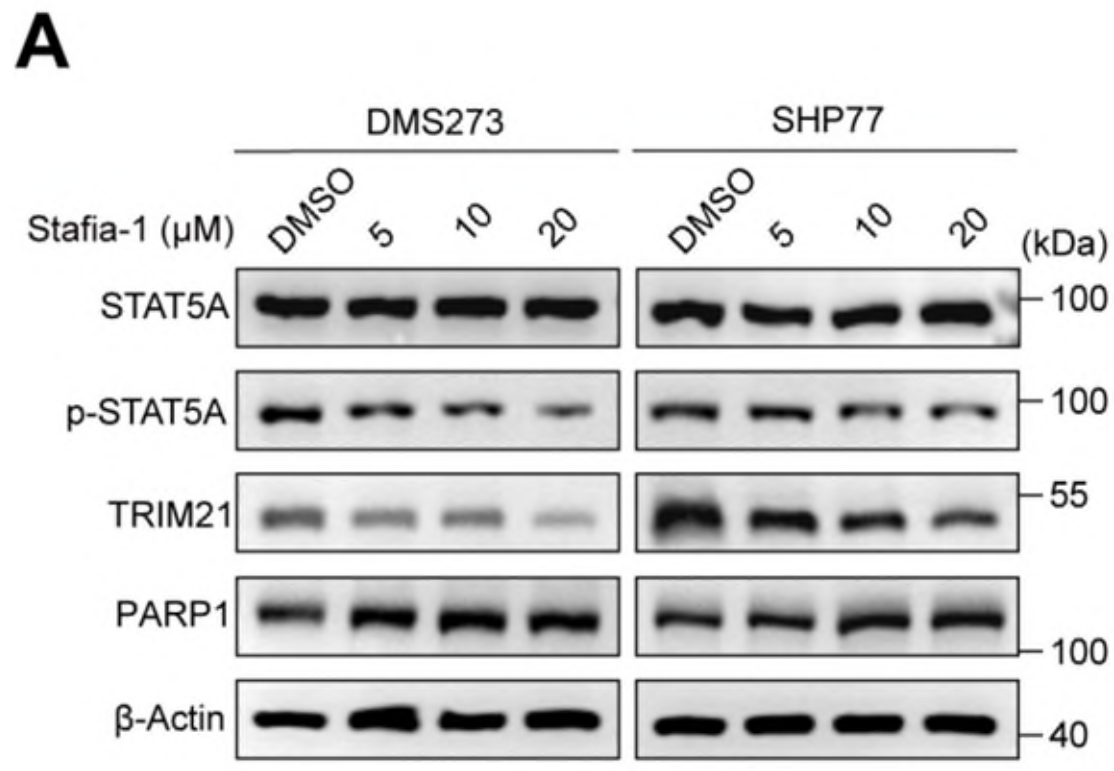

Response Figure 27. **A**) Western blot analysis of STAT5A, p-STAT5A, TRIM21, and PARP1 in DMS273 and SHP77 cells treated with increasing concentrations of Stafia-1 for 24 hours.

We are once more grateful for the reviewer's thoughtful and constructive comments, which have significantly improved our study and broadened our perspective on this research. We believe these additional experimental results adequately address the reviewer's concerns while substantially improving the quality of our manuscript.

3) One study main claim is that the PI3K/AKT pathway negatively regulates STAT5A activation, but that is so far not reported in the literature, despite many studies on both JAK-STAT5 or PI3K-AKT-mTOR action. A known scaffold function for interaction with STAT5 and PI3K was shown to promote cytoplasmic localisation of STAT5 in cancer cells as a result of p85-STAT5 interaction, influencing e.g. nuclear shuttling. mTOR can phosphorylate STAT5A and STAT5B within the coiled coil domain at position serine 193, but the biological consequence is underexplored, which might be relevant here, phosphoproteomic data exists that should be incorporated for SCLC. STAT5 was also shown to directly boost the PI3K-AKT-mTOR pathway in breast cancer cells e.g. through transcribing AKT1 isoform. These studies were incompletely taking into consideration, particularly for the SCLC angle.

Response: Thanks for your valuable suggestions. First, we have analyzed the status of STAT5A serine 193 phosphorylation (p-STAT5A (S193)) in SCLC tissues and revealed a significant decrease in p-STAT5A (S193) levels in SCLC compared to adjacent normal tissues (Response Figure 28A), suggesting attenuated mTOR-mediated phosphorylation of STAT5A in SCLC. Additionally, We have investigated the effects of altered STAT5A expression levels on *AKT1* isoform transcription in SCLC. The results demonstrated that in SCLC cells (DMS273), *STAT5A* knockdown did not

significantly alter *AKT1* mRNA levels, and *STAT5A* reconstitution in knockdown cells similarly showed no effect on *AKT1* transcription (Response Figure 28B). These results suggest that STAT5A may not regulate *AKT1* transcription in SCLC, a mechanism that appears to differ from previously reported regulatory interactions in breast cancer. This indicates that the crosstalk between STAT5A and the PI3K/AKT pathway may exhibit significant tissue or cancer type specificity.

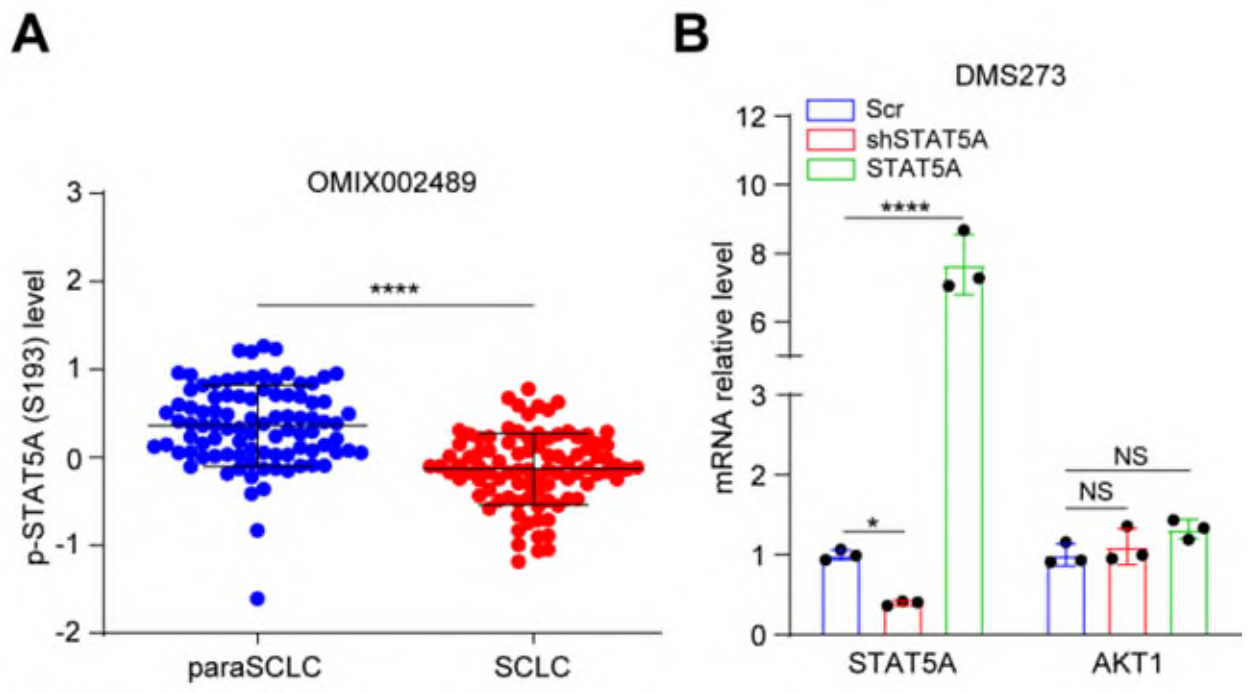

Response Figure 28

Response Figure 28. **A**) The level of STAT5A serine 193 phosphorylation in SCLC and adjacent normal tissues was analyzed using data retrieved from the OMIX database (OMIX002489, n = 93, P < 0.0001). **B**) RT-qPCR Analysis of *STAT5A* and *AKT1* mRNA expression levels. DMS273 cell was stably transfected with *STAT5A* knockdown (sh*STAT5A*), followed by *STAT5A* overexpression rescue (STAT5A). The transcriptional levels of *STAT5A* and *AKT1* were then measured by RT-qPCR using β-Actin as the internal control. Data are represented as mean ± SEM, and were statistically analyzed using the two-tailed unpaired Student's *t*-test. ns, no significance; *P < 0.05, ****P < 0.0001.

On one hand, the well-established literatures (as noted by the reviewer) demonstrate multiple interactions between these pathways, including the p85-STAT5 scaffolding function promoting cytoplasmic localization and mTOR-mediated STAT5 phosphorylation. On the other hand, our data reveal a novel regulatory mechanism in SCLC where PI3K/AKT pathway activity negatively regulates STAT5A at the transcriptional level. These findings suggest a more complex and potentially tumor-specific relationship between STAT5 and the PI3K/AKT pathway than previously recognized, possibly involving uncharacterized feedback mechanisms. This important open question warrants further investigation. We have incorporated this perspective into the revised discussion section (Lines 479-488) to better contextualize our findings within the existing literature while highlighting the novel aspects of our discovery.

We appreciate all referees for improving the quality of the paper. We have now made revisions according to editorial requests, and track changes have been made for the final edits. We hope that the revision is now suitable for publication in *Nature Communications*.

Title: "TRIM21-mediated ubiquitination of PARP1 regulated by the PI3K/AKT/STAT5A axis suppresses the progression of small cell lung cancer"

Tracking #: **NCOMMS-24-73313A**

Authors: Cao *et al*.

Point-by-Point Reply to Reviewers' Comments:

(*The original comments of reviewers are shown in italics.*)

Reviewer #1 (Remarks to the Author):

The authors have tried to address all my comments.

Response: Thanks for the comment.

Reviewer #2 (Remarks to the Author):

Authors have been able to address much of the requested revisions.

Response: I appreciate you taking the time to review and comment.

Some key ones that were stated were the additional SCLC subtypes (P, and Y/I) which shows greater generalizability of their results.

The authors used patient-derived organoid models for part of the revisions (rather then in vivo patient-derived xenograft models) – this lessens the translational assessment, however this may be adequate at this juncture for this type of manuscript (as cell line xenografts were studied).

Response: Thank you for your positive feedback on our inclusion of additional SCLC subtypes (*P* and *Y*/*I*), which strengthens the generalizability of our findings.

1. However, there are some minor revisions recommended to be clarified or incorporated please:

Figure 3D - Please clarify why low TRIM21 expression is associated with shorter overall survival (manuscript figure 3D), yet no significant correlation is observed between TRIM21 expression and chemotherapy or PARPi response. This seems inconsistent with the authors' claim that low TRIM21 expression leads to increased PARP1

levels and genomic stability.

Response: We sincerely appreciate the reviewer's valuable suggestion regarding the relationship between TRIM21 expression and drug response. To further clarify this point, we conducted a comprehensive analysis of the correlation between TRIM21 expression levels and drug sensitivity (IC_{50}) across all compounds in the GDSC2 database for SCLC cell lines. The results revealed that only three compounds exhibited a weak negative correlation between their IC_{50} values and TRIM21 expression ($R < -0.3$) (Response Figure 1). These drugs include two PI3K/mTOR signaling pathway targeted inhibitors (AZD6482 and PF-4708671) and a histone acetyltransferase inhibitor (Remodelin) (Response Figure 1).

Higher TRIM21 expression was associated with greater sensitivity (*i.e.*, lower IC_{50}) to these specific drugs. We speculate that inhibition of the PI3K signaling pathway may relieve its suppressive effect on TRIM21 transcription, leading to upregulated TRIM21 expression, which in turn could enhance TRIM21-mediated PARP1 ubiquitination and degradation, consequently elevating genomic instability. This may represent one plausible mechanism underlying the relative sensitivity to PI3K pathway inhibitors. However, consistent with our prior findings, we observed no significant correlation between TRIM21 expression and sensitivity to PARP inhibitors, suggesting that while TRIM21 targets PARP1 for degradation, it is not an ideal predictive biomarker for PARPi sensitivity.

In summary, within SCLC, TRIM21 expression levels may not serve as a robust biomarker for predicting sensitivity to most targeted therapies or conventional chemotherapeutic agents. This is likely attributable to the intrinsic complexity of TRIM21 as a multi-substrate E3 ligase, which mediates diverse types of ubiquitination on various downstream targets, thereby generating complex and multifaceted biological outcomes that extend beyond PARP1 regulation.

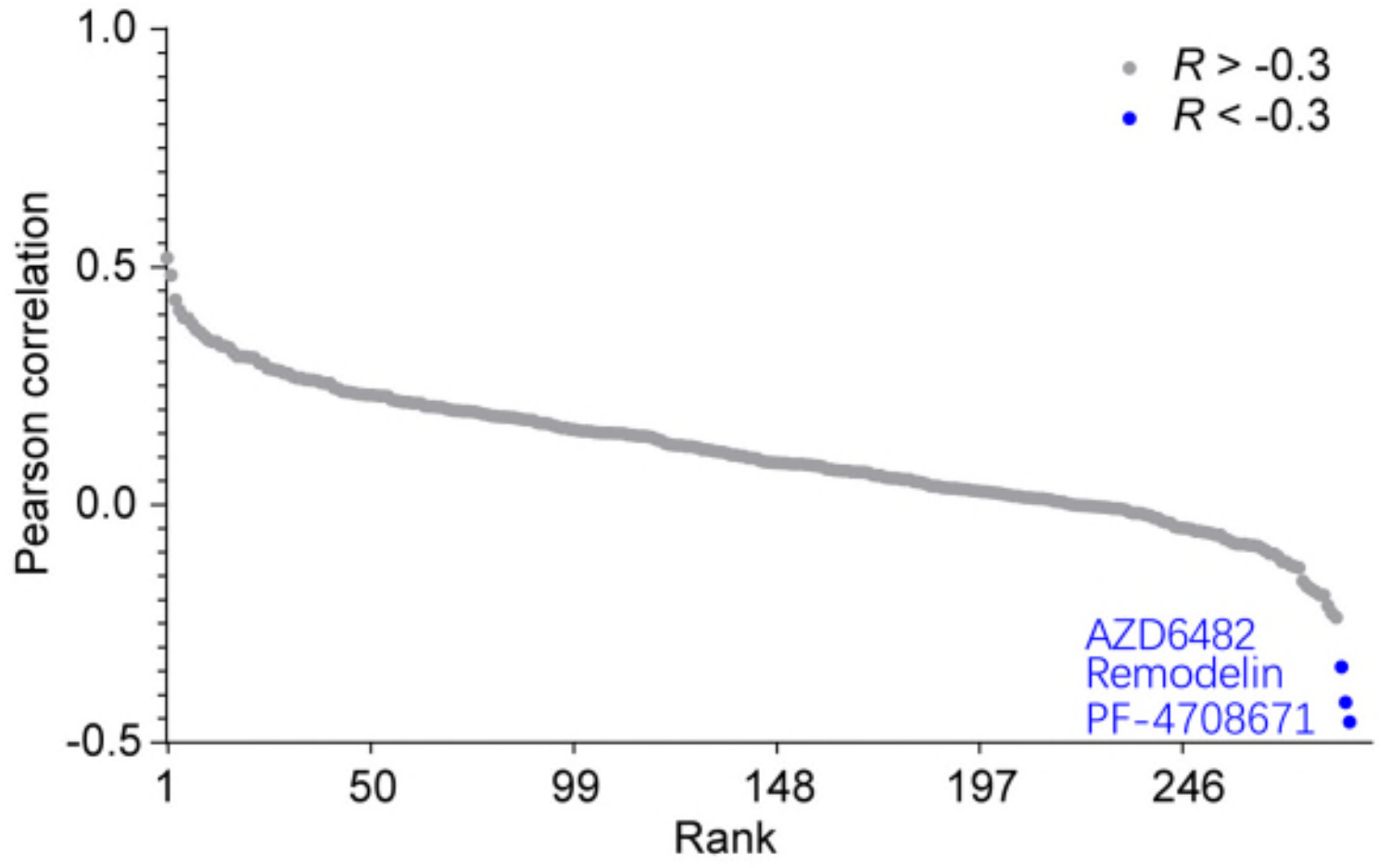

Response Figure 1

Response Figure 1. Correlation between *TRIM21* mRNA expression and the IC_{50} values of 286 anticancer drugs in the GDSC2 dataset.

Figure 4B, clonogenic assay–The images do not appear cleanly quantifiable. In particular, the second and second last wells for DMS273 are difficult to interpret. Please clarify how these were quantified. I note in methods pg 23 lines 638-639 states that "Relative cell densities were 638 analyzed using ImageJ software. The quantification of clonogenic survival was expressed as the ratio of absorbance values relative to the control group." However, absorbance would quantify the surface area covered by cells–and is NOT a quantification of clonogenic potential (i.e. 1 cell being able to form a colony --- see Fig 2A in PMID: 34697469). Could these assays In Fig 4B be re-quantified? Or methodologically infeasible, the definition of this assay as displayed in revised manuscript be then corrected?

Response: Thanks for your critical suggestions. We agree that the methodology should accurately reflect the assay's intention. In response, we have performed the following:

1) Re-evaluation of the clonogenic assay in Figure 4B:

To address the concern regarding the clarity and interpretability of the original images, we have carefully repeated the clonogenic assay for DMS273 under conditions ensuring even cell distribution (Response Figure 2, designated as Figure 4B in revision). The new results show well-defined, distinct colonies that are unequivocally quantifiable, and the quantitative results are consistent with our original findings.

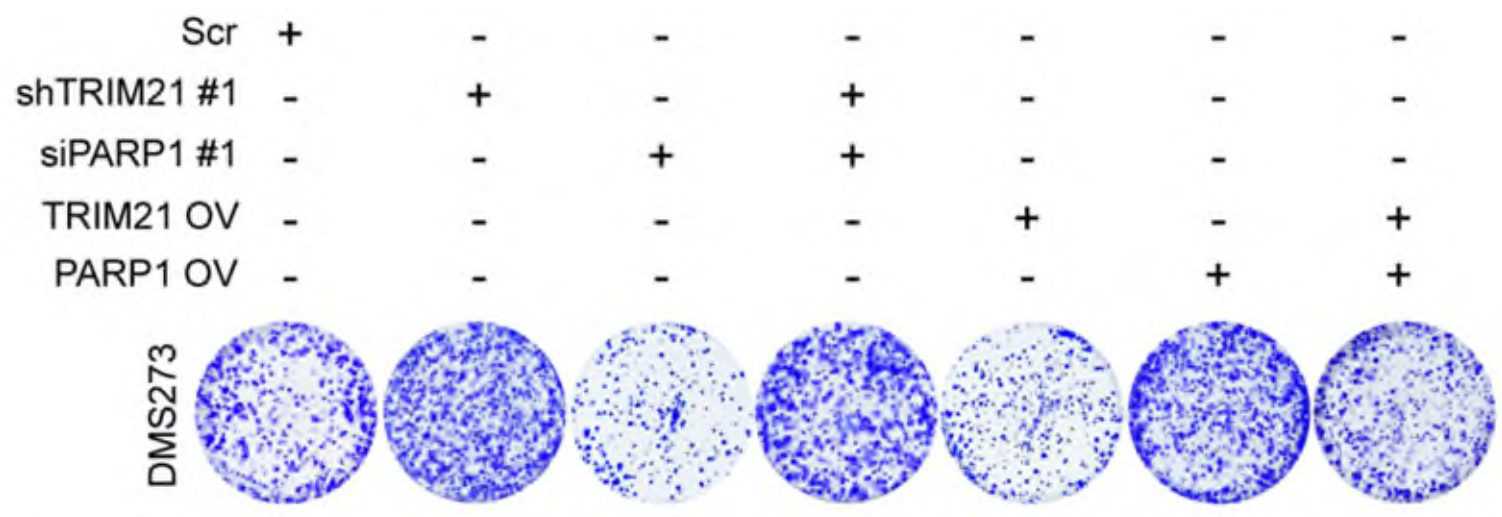

Response Figure 2. Colony formation assays illustrating PARP1-mediated rescue of capacity in *TRIM21*-knockdown or -overexpressing DMS273 cells.

2) Revised quantification method and text:

As suggested by the reviewer, we have re-quantified all clonogenic assays based on the method described in the recommended reference (PMID: 34697469, now cited as reference 71 in revision). Specifically, clonogenic capacity under different genetic manipulations is now expressed as the relative colony formation rate compared to the control, whereas cell survival after drug treatments is presented as clonogenic survival relative to the control. The corresponding methodological description has been incorporated into the manuscript with highlighted details (Lines 635–641). We are pleased to confirm that this revised analysis consistently supports our original conclusions.

Minor revision that could be mentioned as limitations in the results and discussion:

Figure 4A, 4B – Could alternative substrates of TRIM21 explain the observed phenotypes, rather than solely its effects on PARP1? Given that PARP1 knockdown/overexpression only partially rescues the phenotype, this possibility should be considered and discussed a bit more as a potential limitation in the Discussion section. (I note p19 lines 530 does begin to address this but a handful of add'l context sentences would be appreciated)

Response: We fully agree with the reviewer's insightful suggestion regarding the possibility that additional substrates of TRIM21 could contribute to the observed phenotypes, beyond its effect on PARP1 alone. Indeed, the partial rescue upon PARP1 modulation strongly supports that PARP1 is not the sole substrate of TRIM21. Following your recommendation, we have now expanded the discussion of this point both in the results description related to Figures 4A, B (Lines 228-229) and in the "Potential Limitations" section of the Discussion (Lines 528-535). Specifically, we have included relevant background and literature on other known ubiquitination substrates of TRIM21 to further reinforce the conclusion that PARP1 is not its only target. These additions strengthen the rigor and completeness of our study.

Reviewer #3 (Remarks to the Author):

I co-reviewed this manuscript with one of the reviewers who provided the listed reports. This is part of the Nature Communications initiative to facilitate training in peer review and to provide appropriate recognition for Early Career Researchers who co-review manuscripts.

Response: Thanks for the comment.

Reviewer #5 (Replacing Reviewer #4, Remarks to the Author):

The authors have satisfactorily fulfilled the requests of the reviewer.

Response: We thank the reviewer for acknowledging our revisions.

We thank the referees for their positive feedback on our previous revisions. All new editorial requests have been addressed in the revised manuscript, with corresponding track changes provided. We believe the manuscript now meets the standards for publication in *Nature Communications*.
